# Deciphering the universal role of gut microbiota in pollutant transformation

**DOI:** 10.1093/ismejo/wraf215

**Published:** 2025-09-30

**Authors:** Rui Hou, Xiaowei Jin, Jingchun Feng, Jingchuan Xue, Chengzhi Chen, Yuanqiang Zou, Xiangrong Xu, Kefu Yu, Pei-Yuan Qian, Wei Zhang, Jizhong Zhou, Si Zhang, Zhifeng Yang

**Affiliations:** Guangdong Provincial Key Laboratory of Water Quality Improvement and Ecological Restoration for Watersheds, School of Ecology, Environment and Resources, Guangdong University of Technology, Guangzhou, Guangdong 510006, China; China National Environmental Monitoring Centre, Beijing 100012, China; Guangdong Provincial Key Laboratory of Water Quality Improvement and Ecological Restoration for Watersheds, School of Ecology, Environment and Resources, Guangdong University of Technology, Guangzhou, Guangdong 510006, China; Southern Marine Science and Engineering Guangdong Laboratory (Guangzhou), Guangzhou, Guangdong 511458, China; Key Laboratory of City Cluster Environmental Safety and Green Development, Institute of Environmental and Ecological Engineering, Guangdong University of Technology, Guangzhou, Guangdong 510006, China; Guangdong Provincial Key Laboratory of Water Quality Improvement and Ecological Restoration for Watersheds, School of Ecology, Environment and Resources, Guangdong University of Technology, Guangzhou, Guangdong 510006, China; Key Laboratory of City Cluster Environmental Safety and Green Development, Institute of Environmental and Ecological Engineering, Guangdong University of Technology, Guangzhou, Guangdong 510006, China; Department of Occupational and Environmental Health, School of Public Health, Chongqing Medical University, Chongqing 400016, China; State Key Laboratory of Genome and Multi-omics Technologies, BGI Research, Shenzhen, Guangdong 518083, China; Guangxi Laboratory on the Study of Coral Reefs in the South China Sea, Coral Reef Research Centre of China, School of Marine Sciences, Guangxi University, Nanning, Guangxi 530004, China; Southern Marine Science and Engineering Guangdong Laboratory (Guangzhou), Guangzhou, Guangdong 511458, China; Guangxi Laboratory on the Study of Coral Reefs in the South China Sea, Coral Reef Research Centre of China, School of Marine Sciences, Guangxi University, Nanning, Guangxi 530004, China; Southern Marine Science and Engineering Guangdong Laboratory (Guangzhou), Guangzhou, Guangdong 511458, China; Department of Ocean Science, The Hong Kong University of Science and Technology, Hong Kong 999077, China; Centre for Marine Bioproducts Development, College of Medicine and Public Health, Flinders University, Adelaide, South Australia 5001, Australia; Institute for Environmental Genomics, University of Oklahoma, Norman, OK 74078, United States; Southern Marine Science and Engineering Guangdong Laboratory (Guangzhou), Guangzhou, Guangdong 511458, China; Guangdong Provincial Key Laboratory of Water Quality Improvement and Ecological Restoration for Watersheds, School of Ecology, Environment and Resources, Guangdong University of Technology, Guangzhou, Guangdong 510006, China; Southern Marine Science and Engineering Guangdong Laboratory (Guangzhou), Guangzhou, Guangdong 511458, China; Key Laboratory of City Cluster Environmental Safety and Green Development, Institute of Environmental and Ecological Engineering, Guangdong University of Technology, Guangzhou, Guangdong 510006, China

**Keywords:** gut microbiota, pollutant transformation, toxicological outcomes, “pollutant–gut microbiota–host” interactions, interdisciplinary research framework

## Abstract

The gut microbiota represents a critical yet underexplored “second genome” in the host that functions as a key driver of pollutant transformation across Earth’s ecosystems. This review synthesizes the current understanding of over 490 pollutants across a wide range of species, highlighting the universal role of gut microbial communities in modifying pollutant exposure. We have demonstrated that gut microbial communities transform a broad spectrum of environmental pollutants through evolutionarily conserved pathways, fundamentally altering their bioavailability, fate, and toxicity potential within the host. Transformation reactions are elucidated with connections among the metabolic enzymes that are developed by specific gut microbes, emphasizing the markedly specific and complementary signatures of microbial biotransformation compared to the host process. By integrating multidisciplinary studies, the complex and dynamic interplay between the gut microbiota, host physiology, and environmental pollutants has been elucidated, and drivers involved in the biotransformation processes have been proposed. Furthermore, current methodologies are critically evaluated, and next-generation approaches to reveal the underlying mechanisms of gut microbiota-driven pollutant transformation are outlined. This review underscores the urgent need to systematize research on “pollutant–gut microbiota–host” interactions and advocates the integration of gut microbial perspectives into interdisciplinary research paradigms of toxicology, microbiology, and ecology.

## Graphical Abstract

**Graphical Abstract:**
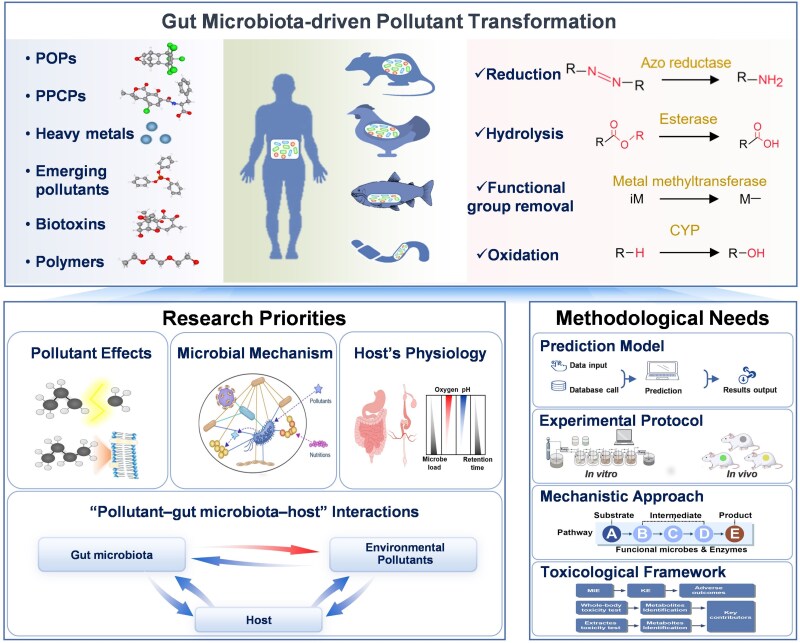

## Introduction

Environmental pollutants pose unprecedented challenges to ecosystems and human health in the Anthropocene [1]. While traditional toxicology has focused primarily on direct host–pollutant interactions, emerging evidence reveals a critical yet underexplored driver in environmental toxicology: the gut microbiota. The gut microbiota, which can be found in virtually any metazoan, from invertebrates to vertebrates, serves as a crucial interface between environmental exposure and biological responses [2]. This complex microbial ecosystem, which comprises trillions of microbes with > 150-fold more genes than their host [3, 4], performs functions including resistance to pathogens, regulation of the immune system, fermentation of nondigestible dietary fibers, anaerobic metabolism of peptides and proteins, interaction with the circadian rhythm, and biotransformation of xenobiotics [5, 6]. There is now growing recognition that the gut microbiota functions as an “external” organ for the host [7, 8].

In recent years, the “game-changing potential” of the gut microbiota with respect to its effects on the mode of action (MOA) and the absorption, distribution, metabolism, and excretion (ADME) of pollutants have since shaped the ecological and environmental toxicology. The gut microbiota acts as the first physical and biological barrier against environmental pollutants from the diet as it resides at the site of exposure. Environmental pollutants, such as antibiotics [9], heavy metals [10, 11], persistent organic pollutants (POPs) [12, 13], pesticides [14–16], nanomaterials [17, 18], and other emerging pollutants [19], strongly influence the structure and activity of the gut microbiome. The gut-“X” axis of the gut is considered an important target of pollutant toxicity, such as intestinal injury [20], hepatic diseases [21], metabolic disorders [22], immune perturbations [23], and behavioral and neurochemical alterations [24, 25]. In addition, the gut microbiota harbors diverse enzyme families with xenobiotic-transformation potential, which function as modifiers of pollutant exposure in organisms. Many recent cutting-edge and innovative efforts in toxicology have made great strides toward gut microbiota-driven pollutant transformation in organisms ranging from invertebrates to humans [5, 26–28]. The gut microbiota has been revealed to play an important role in the biotransformation of pollutants, and different chemical transformation modes and toxicity modifications associated with the hepatic process have been described [29]. Overall, research on the gut microbiota has spurred a paradigm shift from focusing solely on host-level consequences to adopting a more integrative toxicological approach that emphasizes host–microbiota symbiosis as a key determinant of pollutant-induced adverse outcomes.

Over the past few years, the impact of environmental pollutants on the gut microbiota, together with induced host toxicology, has been described in various recent reviews [7, 19, 28, 30, 31]. Although some reviews have summarized the available information on pollutant biotransformation by the gut microbiota [5, 26–28, 32–35], most have focused primarily on the human gut microbiota as a major target. Globally, mechanistic and critical reviews of gut microbiota-driven pollutant transformation grounded in the “One Planet, One Health” concept (encompassing the multidisciplinary links among planet, human health, and animal health) are still lacking. Many recent cutting-edge and innovative efforts in toxicology have made great strides toward gut microbiota-driven pollutant transformation in organisms ranging from invertebrates to humans [5, 26–28], covering: (i) potential candidate microbes and their transformation activities; (ii) transformation kinetics, pathways, and mechanisms of target pollutants; and (iii) changes in pollutant bioavailability and toxicity during microbial transformation. Pollutant transformation by the gut microbiota may be far more diverse and complicated than previously believed because of the complex interplay of several factors. The gut microbiota encompasses diverse domains of life, including archaea, bacteria, fungi, and viruses, all of which could contribute to pollutant transformation. Differences in the amount and composition of the gut microbiota among species directly affect enzyme activities, resulting in significant variation in the effects on pollutant transformation; in contrast, pollutant structures and properties can also determine the intrinsic recalcitrance of pollutants to microbial transformation [36]. Moreover, various host-related factors, such as host genetics, lifestyle, diet, and environmental stress, can also dramatically modulate microbial capacity and efficiency in pollutant transformation [30, 37, 38]. To date, the “gut microbiota-driven transformation” is a research gap in which specialization has been neglected and research frequently overlooks, where the associated direct transformation modulations and the indirect modulations under “pollutant–microbiota–host” interactions are the key issues to be addressed.

By synthesizing insights from toxicological, microbiological, and ecological research, this review aims to provide a comprehensive framework for understanding how gut microbiota-driven biotransformation shapes the impact and fate of environmental pollutants. First, a panoramic overview of the role of the gut microbiota in the ADME processes of pollutants is provided, and scenarios where the gut microbiota participates in the pollutant transformation are proposed. Second, specific pathways (the enzymes involved), functional microbes, and their corresponding effects on toxicity in gut microbiota-driven transformation are summarized on the basis of the latest knowledge of pollutant transformation. Third, the drivers of this gut microbial transformation distribution are analyzed from the basic perspective of “pollutant–gut microbiota–host” interactions. Finally, methodological advances in the study of gut microbial transformation are presented, and key issues that should be addressed in the future are suggested. This timely and essential review of gut microbiota-driven transformation will not only advance scientific understanding but also facilitate the development of more accurate risk assessment models and innovative strategies to mitigate the ecological and human health impacts of environmental pollution.

## A brief overview of gut microbiota-driven transformation and its influence on pollutant absorption, distribution, metabolism, and excretion (ADME) processes

The ADME processes determine the internal exposure and toxic action of pollutants within the body. Among these factors, host-gut microbiota cooperative transformation is recognized as both the most influential and the least understood factor (Box 1A). Due to their distinct physiological and enzymatic properties, the host (particularly the liver, a primary site of chemical transformation) and the gut microbiota often exhibit divergent, or even opposing, capabilities in metabolizing pollutants. As a highly vascularized organ with a constant oxygen supply, the liver predominantly facilitates detoxifying transformation through the action of cytochrome P450 monooxygenases (CYPs), hydrolases, and conjugating enzymes [39, 40]. In contrast, much of the gut of large animals are located in reducing environments, where the gut microbiota cannot rely on oxygen as the terminal electron acceptor for respiration [41]. In general, hepatic transformation tends to exhibit broad substrate specificity, whereas the gut microbiota is capable of mediating a wide array of metabolic reactions, including reductive and hydrolytic processes [42].

**Box 1:** Overview of the complex metabolic network of pollutants in the host-gut microbiota cooperative process (A) and the four proposed scenarios of pollutant transformation with respect to the gut microbiota (B).

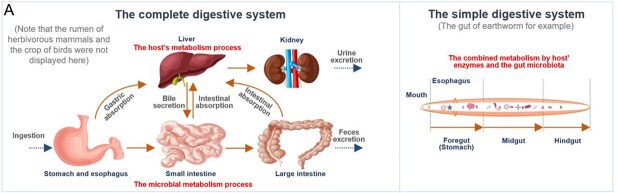
There are different ADME processes for xenobiotics in the digestive system of invertebrates and high-trophic level organisms. In the complete digestive system, orally ingested chemicals first experience the extremely acidic environment of the stomach (pH 1.5) [43], where various pH-sensitive compounds can undergo abiotic hydrolysis and reduction reactions. Subsequently, these compounds can be jointly modified by digestive enzymes and the gut microbiota in the small intestine and colon. They are then absorbed by the intestinal tissues and enter into circulation. Compounds in the circulatory system can be processed by hepatic transformation and excreted via the kidneys. Bile excretion provides another opportunity for metabolism by the gut microbiota, in which the phase II metabolites can be transformed to the conjugated form then reabsorbed during the “enterohepatic cycle” [4, 26]. Eventually, chemicals and metabolites that are not absorbed by the large intestine can be excreted via feces. In the simple digestive system, host and microbial transformation processes are more likely to occur simultaneously and function together [44].
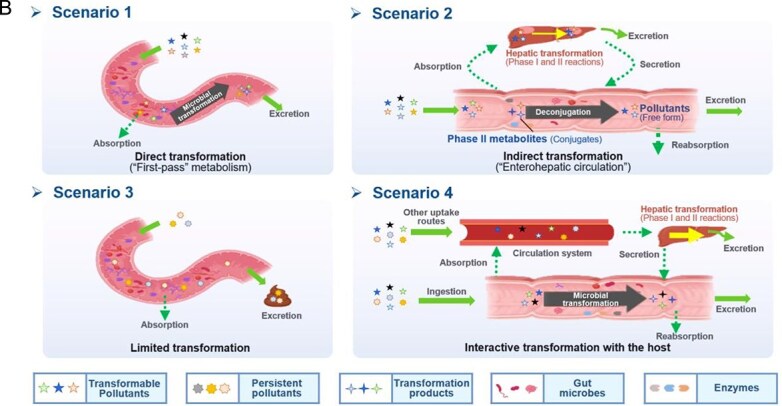
There are four scenarios in which the gut microbiota participates in pollutant transformation: (1) the “first-pass metabolism” of ingested pollutants by the gut microbiota; (2) the hepatic metabolites (mainly the phase II metabolites) of some pollutants in the circulatory system may also be excreted via the bile back to the gut lumen, after which they are reprocessed by the gut microbiota; (3) some persistent pollutants cannot be degraded due to their persistence and limited residence time in the GI tract; and (4) the gut microbiota participates in a complex and interactive process with the host.

In humans and other vertebrate animals with a complete digestive system, pollutants can encounter the gut microbiota through various pathways. Once absorbed by the gastrointestinal (GI) tract, the ingested pollutants often undergo “first-pass metabolism (presystemic metabolism)” by the host’s enzymes and microbial enzymes before they reach the systemic circulatory system. Hepatic metabolites in the circulation may also be excreted via the bile back to the gut lumen and reprocessed by the gut microbiota [4, 26], where the subsequent deconjugation of the excreted phase II metabolites can result in “enterohepatic circulation” of chemicals in the body. Invertebrates possess a simple digestive system (a simple tube of enterocyte cells) harboring the gut microbiota [44], often resulting in a relatively simple host–gut microbiota transformation process. Overall, the host- and gut microbiota-driven transformations form a complex, complementary, and interactive metabolic network.

The role of the gut microbiota in pollutant transformation and the alteration of toxicokinetic and toxicodynamic properties are closely tied to the structure and exposure route of the pollutant. Briefly, gut microbiota-driven pollutant transformation in humans and other vertebrate animals can be categorized into four distinct scenarios (Box 1B). With respect to the susceptibility of pollutants to microbial transformation, “first-pass metabolism” by the gut microbiota can be considered the first barrier for the entry of pollutants into the blood circulation system (i.e. direct transformation). The easily absorbed and readily hepatically conjugated pollutants are more likely to be reproposed by the gut microbiota, thereby spending long periods in the systemic circulation through this process of “enterohepatic circulation” (i.e. indirect transformation) [4, 26]. In contrast to the previous two scenarios, the gut microbiota can hardly modify rapidly absorbed or microbe-resistant chemicals (the third scenario). In addition, the gut microbiota mainly exerts direct effects on dietary compounds. Because the exposed pollutants bypass this “first-pass metabolism” to enter systemic circulation via another route (i.e. inhalation, dermal intake, or intravenous injection), only the bile-excreted proportion can be processed by the gut microbiota [26]. In real-world scenarios involving mixed pollutant exposure, host–gut microbiota cooperative biotransformation is the most common occurrence (the fourth scenario). Consequently, ADME studies must meticulously distinguish between these distinct transformation processes and account for exposure- and structure-specific mechanisms when evaluating the role of gut microbiota–pollutant interactions.

## Universality of gut microbiota-driven pollutant transformation

**Figure 1:**
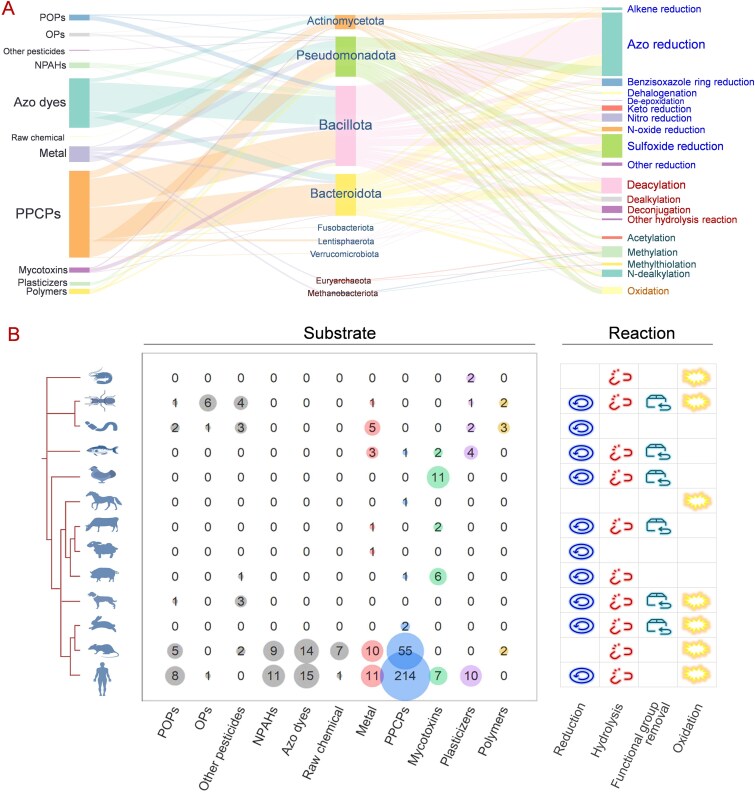
Distribution of pollutant biotransformation by gut microbes from mammals and non-mammal fauna. (A) Consensus evidence of pollutant–microbe–reaction triad can be found in Supplementary Tables S1–S6. (B) Diversity of transformable pollutants and reaction types across 13 taxonomic groups. The size of the circles represents in the left the number of transformable pollutants for a particular category on the left. The symbols on the right indicate whether a certain reaction was reported for the gut microbial transformation within a certain taxonomic group. Organisms categorized as follows (top to bottom): other invertebrates, diptera insects, worms, fish, chickens, horses, cattle, sheep, pigs, dogs, rabbits, rats, and humans. Abbreviations: OPs, organophosphate pesticides (OPs); NPAHs, nitrated polycyclic aromatic hydrocarbons; POPs, persistent organic pollutants; PPCPs, pharmaceuticals and personal care products.

The capacity of gut microbiota to transform environmental pollutants demonstrates remarkable universality, as shown by a comprehensive analysis of the biotransformation of over 490 structurally diverse pollutants by animal species across 13 taxonomic groups, spanning invertebrates, fish, birds, non-human mammals, and human. Supporting data on transformation pathways, functional microbes, and potential toxicity modulation are summarized from the environmental toxicology and ecotoxicology studies (Fig. 1; Table 1; Supplementary Tables S1–S6).

**Table 1:** General mechanisms and toxicological consequences of gut microbiota-driven transformation.

**Category**	**Reaction types**	**General reaction mechanism**	**Representative chemical targets**	**Toxicology consequences**
Reduction	Azo reduction	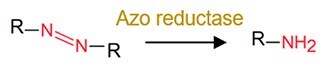	Azo-dyes and azo-antibacterial predrugs (i.e. balsalazide analogs and prontosil)	Generate carcinogenic aromatic amines from azo-dyes; Release products with efficacy (toxicity).
	Nitro reduction	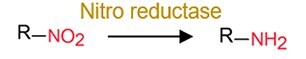	Nitro-PAHs, benzodiazepines drugs (i.e. nitrazepam, clonazepam, and bromazepam), and metronidazole	Decrease toxicity of nitro-PAHs; Increase carcinogenicity of benzodiazepines.
	Alkene reduction	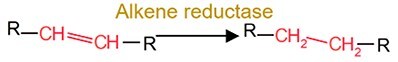	Deleobuvir and digoxin (the drugs)	Decrease efficacy (toxicity).
	Sulfoxide reduction	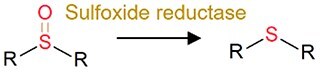	Sulfur-containing drugs (i.e. omeprazole and sulfinpyrazone)	Release products with efficacy (toxicity).
	N-oxide reduction	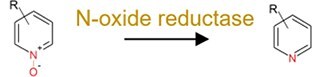	N-oxide prodrug (i.e. loperamide oxide) and H2 receptor antagonists (i.e. ranitidine and nitazidine)	Release products with efficacy (toxicity) for the prodrug; Decrease the efficacy of the antagonists.
	Keto reduction	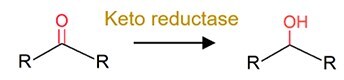	Hydrocortisone (the endocrine drug) and zearalenone (the mycotoxin)	Increase efficacy (toxicity) of zearalenone. Increase toxicity of zearalenone.
	De-epoxidation	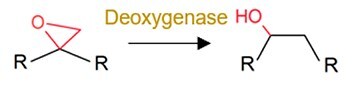	Mycotoxin (i.e. deoxynivalenol and nivalenol)	Decrease toxicity.
	Dehalogenation	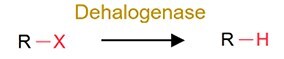	DDT, PCBs, and other organochlorine pesticides	Decrease toxicity.
	Metal reduction	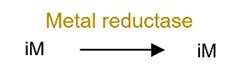	iAs^V^	Increase toxicity of iAs.
	Metal demethylation	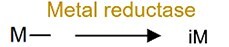	CH_3_Hg^I^	Decrease toxicity and bioavailability.
Hydrolysis	Dealkylation	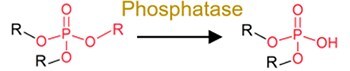 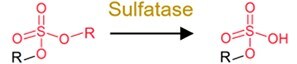 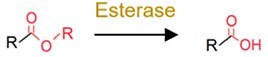	Ester-containing drugs (i.e. carbetapentane citrate, ketorolac, and L-Dopa), organophosphate pesticides, organophosphate flame retardants and parabens	Decrease toxicity and bioavailability in most cases.
	Deacetylation	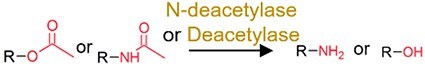	N- or O-acetyl chemicals and drugs (i.e. acetaminophen, formanilide, and irinotecan) and acetyl mycotoxins (i.e. T-2 toxin)	Increase bioavailability in most cases; Sometimes increase toxicity (i.e. diltiazem, irinotecan, and spironolactone).
	Deglycosylation	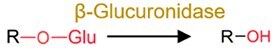	Conjugates of drugs, personal care products, mycotoxins, pesticides, nirto-PAHs and plastic additive	Increase bioavailability.
	Other deconjugation	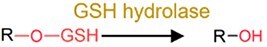 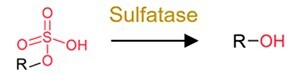	Conjugates of drugs, personal care products, nirto-PAHs, and pesticides	Increase bioavailability.

*(continued)*

### Substrate and reaction universality of the gut microbial transformation

#### Legacy organic pollutants

Even though legacy organic pollutants including POPs possess relatively stable chemical structures, substantial evidences have confirmed their biotransformation by gut microbiota of humans and rodents (Supplementary Fig. S1). For instance, dechlorination has been identified as a general reductive metabolic pathway for a wide range of chlorinated compounds [45, 46], including dichlorodiphenyl trichloroethane (DDT), organochlorine pesticides (OCPs) such as dieldrin, lindane, and methoxychlor, as well as polychlorinated biphenyls (PCBs). Similarly, the ester bonds in organophosphate pesticides (OPs) and other ester-containing raw chemicals render these compounds susceptible to hydrolysis by enzymes (e.g. phosphoesterases) derived from human gut microbiota [47, 48]. Furthermore, the nitro-containing pollutants, such as nitro-polycyclic aromatic hydrocarbons (nitro-PAHs), can be reduced by gut microorganisms to amino-polycyclic aromatic hydrocarbons (amino-PAHs), thereby enhancing their mutagenicity and carcinogenicity; for example, certain bacteria such as *Klebsiella* sp. C1, which are isolated from humans may be involved in this process [49, 50]. Similarly, azo dyes are susceptible to biotransformation by azo reductases produced by specific gut bacteria, including *Clostridium perfringens*, *Bacteroides ovatus*, *Enterococcus faecalis*, and *Ruminococcus obeum*, as well as the broader human gut microbiota [51–53], though which generates carcinogenic aniline derivatives. PAHs have also been reported to undergo hydroxylation by CYPs from the gut microbiota [54, 55], yielding products with elevated estrogenicity. Furthermore, the gut microbiota-mediated biotransformation of melamine has attracted much attention due to the significant increase in nephrotoxicity caused by the formation of cyanuric acid [56].

**Table 1:** Continued.

**Category**	**Reaction types**	**General reaction mechanism**	**Representative chemical targets**	**Toxicology consequences**
Functional group removal	Acetylation	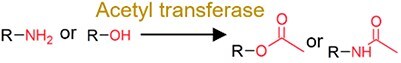	Some drugs (i.e. 5-aminosalicylate, sulfapyridine and sulfasalazine)	Decrease bioavailability in most cases.
	Methylthiolation	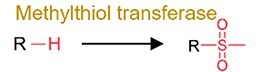	Organochlorine pesticides (i.e. PCB29 and chlorothalonil)	Increase bioavailability in most cases.
	N-dealkylation	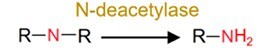	Some drugs and neonicotinoid pesticides	Decrease toxicity
	Metal methylation	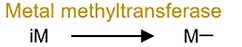	As, Bi, Hg, Sb, Se, and Te	Decrease toxicity and increase bioavailability.
	Metal thiolation	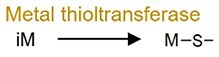	As and Se	Decrease toxicity and increase bioavailability.
Oxidation	Hydroxylation	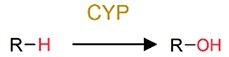	PAHs and phenol	Increase toxicity (estrogenicity) and bioavailability.
	Alkyl cleavage	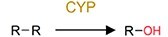	Plastic additives	Change toxicity (estrogenicity) and bioavailability.
	Oxidative depolymerization	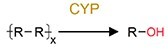	Plastic and nanomaterials	Decrease toxicity.

#### Metals

Microbial transformation of metals often involves complex networks of redox, methylation, and thiolation reactions, profoundly influencing their mobility and toxicity. Arsenic (As) metabolism provides a seminal example of this metabolic versatility, which display complex transformation networks: iAs^V^ can be reduced to iAs^III^ by sulfate-reducing bacteria (e.g. *Desulfovibrio*), subsequently methylated to monomethyl- (MMA^V^) and dimethylarsinic acid (DMA^V^), and thiolated to generate products such as monomethylmonothio- (MMMTA^V^), dimethylmonothio- (DMMTA^V^), dimethyldithioarsinic acid (MMDTA^V^), and dimethyldiothio-arsonic acid (DMDTA^V^) [57–59]. Human gut microbiota can also oxidize iAs^III^ to iAs^V^ [60]. Meanwhile, organic arsenic compounds (e.g. MMA^V^, DMA^V^, AsB, and arsenic sugars) can also be transformed by the gut microbiota through hydrolysis to enhance their host absorption [61–63]. Critically, this capacity for biotransformation is not unique to As; similar bacterial methylation pathways have been confirmed for other metals including Hg, Bi, Sb, Se, and Te (Supplementary Fig. S2), highlighting a conserved functional role across metal substrates.

#### Pharmaceutical and personal care products (PPCPs)

The role of the gut microbiota in pharmaceutical transformation has long been recognized and considered in the field of pharmaceutical design (Supplementary Fig. S3). The reductive metabolism of pharmaceuticals represents a significant biotransformation process, particularly for structurally distinct groups such as azo (–N=N–), nitro (–NO₂), alkenes (–C=C–), ketones (–C=O), N-oxides (–N–O), and sulfoxides (–S=O). For example, the azo drugs (mainly the digestion-related drugs) of balsalazide, ipsalazide, neoprontosil, olsalazine, prontosil, and sulfasalazine are intentionally designed to avoid rapid adsorption to ensure their effective delivery to the colonic region [64]. These prodrugs rely on azo reductases secreted by the gut microbiota for azo bond cleavage to release active ingredients [4, 65]. Nitrazepam can be efficiently reduced to 7-aminonitrazepam by *Clostridium leptum* from human gut [66], whereas the cardiac drug digoxin can undergo alkene cleavage by the human gut microbiota to produce dihydrodigoxin [67]. Hydrolysis is another critical metabolic pathway for pharmaceuticals by the gut microbiota. For instance, *Helicobacter pylori*, a bacterium found in human gut, can initially inactivate L-DOPA by decarboxylating it to generate *m*-tyramine and *m*-hydroxyphenyl-acetic acid via hydrolase activity [68]. Similarly, antibiotics including benzylpenicillin and chloramphenicol can also be dealkylated by the gut microbiota to counteract their adverse effects [69]. In addition, deconjugation mediated by microbial β-glucuronidases and other hydrolases can transform the detoxified pharmaceuticals (the conjugates), such as itirican (SN38) [70], acetaminophen [71], diclofenac [72], indomethacin [73], and triclosan [74], extending their pharmacological or toxicological effects.

#### Mycotoxins

Although mycotoxins are natural fungal metabolites, they have attracted global attention as emerging pollutants that adversely affect human health [75]. The transformation of deoxynivalenol (DON) into deepoxy-deoxynivalenol by the gut microbiota is an efficient detoxifying transformation that occurs by the gut microbiota (Supplementary Fig. S4) [76]. In addition, hydrolysis can also be conducted by the gut microbiota to detoxify mycotoxins, such as ochratoxin A [77]. The sulfate and glucuronide conjugates of mycotoxins (e.g. DON [75, 76] and zearalenone [78]) can also be rapidly deconjugated by the gut microbiota, extending their half-lives in the body.

#### Polymers and nanomaterials

A key universal mechanism for polymer biotransformation by the gut microbiota involves enzymatic oxidative fragmentation initiated by microbial oxidases (e.g. cytochrome P450 and laccases), followed by depolymerization and assimilation. This pathway has been demonstrated for a range of synthetic polymers, such as polyethylene [79, 80], low-density polyethylene [81], polystyrene [82–84], polyvinyl chloride [85], and polylactic acid [86]. These processes ultimately metabolize polymers into monomers, low-molecular-weight organic compounds, and the subsequent CO_2_ and H_2_O. In addition, carbon nanomaterials have been confirmed to be transformed into endogenous organic metabolites via anaerobic fermentation in the gut, incorporating inorganic carbon into organic butyrate [87].

#### Other emerging pollutants

Bisphenol A and disinfection byproducts (e.g. chloroacetonitrile, dibromoacetic acid, and tetrabromopyrrole) are transformed in the simulator of human intestinal microbial ecosystem (SHIME), mitigating oxidative DNA damage [88, 89]. Furthermore, tetrabromobisphenol A and its analogs (e.g. tetrachlorobisphenol A and tetrabromobisphenol S) can suffer debromination and glycosylation by the human gut microbiota [90, 91], whereas microbial deglycosylation of the conjugates of these compounds is also observed [91]. Esters can increase the susceptibility of organophosphate flame retardants and phthalate esters to hydrolysis by the gut microbiota [92, 93]. In addition, our previous study on crucian carp has demonstrated that gut microbiota-mediated alkyl-cleavage significantly alters the estrogenic effects of bisphenol A and its analogs [94].

Overall, the gut microbiota-driven pollutant metabolic reactions generally include the following: (i) reduction (e.g. alkene reduction, azo reduction, de-epoxidation, dihydroxylation, dehalogenation, enoate reduction, hydrazine cleavage, keto reduction, nitro reduction, N-oxide reduction, and sulfoxide reduction); (ii) hydrolysis (e.g. deacylation, dealkylation, deamidation, decarboxylation, deconjugation, proteolysis, and thiazole ring-opening); (iii) functional group removal (e.g. acetylation, methylthiolation, N-dealkylation, and transamination); and (iv) oxidation. The extensive transformation of diverse environmental pollutants demonstrates the broad substrate diversity inherent to the gut microbiota metabolic capabilities.

### Cross-species universality of the gut microbial transformation

Reduction reactions are widely prevalent microbial-mediated transformation processes for pollutants across 13 taxonomic groups (Fig. 1B). The metabolism mediated by the gut microbiota is widely regarded as a response-modifying process that reflects the energy demand of gut microbes [95]. Most of the gut parts of large animals are in reducing environments, and as a result the gut microbiota in the intestine cannot utilize oxygen as the terminal electron acceptor for respiration [41]. Consequently, in contrast to oxidation, which ranks first in host metabolic processes, the redox potential in the intestine is suitable for gut microbes because it transfers hydride equivalents or electrons (H^+^ and 2e^−^) to substrates [5, 26, 27]. For example, dehalogenation reactions of halogenated organic compounds (primarily classified as POPs) have been widely observed in the guts of various species, including humans [46], rodents [45], earthworms [96], and apple maggots [97]. Parallel transformation conservation occurs for metal reduction. In studies of fish, rodents and humans, both the reduction and methylation could be mediated by the gut microbiota to inorganic and organic arsenic (iAs and oAs), encompassing the reduction, methylation, and thiolation pathways [61–63, 98, 99]. This metabolic versatility extends to the invertebrates’ gut microbiota, where the *Escherichia coli*. From earthworm gut (*Eisenia foetida*) can reduce and methylate As [100]. Mycotoxin’s detoxifying transformation further demonstrates cross-species conservation: reductive pathways efficiently transform DON, HT-2, nivalenol, verrucarol, and T-2 toxins in fish [101], chickens [102, 103], pigs [104], and humans [76].

Hydrolysis and functional group removal reactions are important mechanisms through which the gut microbiota participates in host energy metabolism; these two reactions can produce simple molecules that can be used for energetic purposes without relying on oxidants [26, 105]. For endosulfan, a special OCP with a sulfate group, endosulfan diol has been confirmed as a microbial triphenyl produced through hydrolysis by *Rhodococcus* found in earthworms [106]. The plasticizers with esters (e.g. di(2-ethylhexyl) phthalate and tri(2-butoxyethyl) phosphate) undergo hydrolysis by specific gut microbes from earthworms [92, 93] and fish [94], and the hydrolysis of OPs occurs exclusively in various mammals and invertebrates [107–109]. Deconjugation is also a widespread and highly efficient hydrolysis reaction that occurs across species (including humans [4, 70, 91, 110], other mammals [111, 112], fish [113], prawns [113], and mussels [113]) and across diverse xenobiotic classes, such as drugs, POPs, mycotoxins, and plasticizers. These reactions, which typically proceed rapidly, highlight the microbiota’s inherent and host-independent capacity to transform conjugated compounds, regardless of specific host-microbiota interactions. In addition, N-dealkylation (the functional group removal reaction) can be rapidly conducted for pharmaceutical neonicotinoid pesticides or by the gut microbiota of worms and mammals [3, 114].

Evidence of pollutant oxidation reactions that are driven by the gut microbiota has also been reported from invertebrates to vertebrates, although oxidation reactions are typically oxygen-sensitive and require a significant amount of energy. In invertebrates, the relatively simple gut structure of invertebrates allows higher oxygen availability in their gut environment, thereby facilitating oxidative reactions [115, 116]; in contrast, vertebrate gut systems contain more anoxic gut environments, but CYP enzymes are highly abundant in several facultative anaerobic bacteria [117, 118]. For example, the ability of the gut microbiota to transform various plastics was discovered in studies based on earthworms [81], mealworms [83], superworms [84, 116], other invertebrates [119–121], and the mammals [122]. The facultatively anaerobic and strictly aerobic bacteria are ubiquitous in the gut may promote oxidation under both anoxic and oxic conditions [115, 116].

Although the gut microbiota across species shares a universality for the general pollutant transformations, species-specific differences in final outcomes often arise due to the simultaneous operation of diverse and intricate microbial transformation processes. For example, in earthworms, the gut microbiota primarily mediates the methylation of inorganic mercury (iHg) into methylmercury (MeHg), with sulfate-reducing bacteria (e.g. *Desulfovibrio*) serving as the key methylators [123]. In contrast, studies on fish have shown that while their gut microbiota can simultaneously drive both the methylation and demethylation of Hg [124, 125], where the demethylation process appears to be more efficient. Collectively, these cross-species examples illustrate conserved functional roles of the gut microbiota in pollutant transformation, serving as a critical modifier against environmental pollution.

### General toxicological consequences of gut microbial transformation

Transformation processes driven by the gut microbiota have attracted much attention because they can significantly alter the bioavailability (pharmacokinetic parameters) of certain pollutants and induce changes in their toxicological effects (Table 1) [5, 7, 28]. The reduction reactions generally convert chemicals into more water-soluble metabolites and facilitate their excretion from microbial cells, but the toxicological outcomes are dependent on substrate specificity [26]. For example, nitro reduction generates amine metabolites with weak toxicity, and azo reduction generally induces compound carcinogenicity. The hydrolysis of the pollutant itself markedly reduces host exposure, whereas the deconjugation of the pollutant metabolites results in the reactivation of pollutants and increases their bioavailability in the gut (Table 1). In functional removal reactions, the installation or removal of specific groups can also affect the bioavailability and toxicity of pollutants. For example, the acetylation of pollutants (e.g. 5-aminosalicylate and sulfapyridine) has been shown to reduce toxicity (or efficacy) [126], whereas the methylation of heavy metals may increase their bioavailability and toxicity [127].

To date, few toxicokinetic studies have assessed the role of the gut microbiota in pollutant transformation and its combined impact on host metabolism. A bioavailability study of disinfection byproducts via SHIME revealed that ~ 60% of chloroacetonitrile, 45% of dibromoacetic acid, and 80% of tetrabromopyrrole underwent abiotic transformation in the stomach and small intestine. Subsequent transformation of the remaining compounds occurs in the colon, facilitated by the gut microbiota, underscoring the pivotal role of gastrointestinal transformation in the toxicology of these orally ingested pollutants [89]. Research has confirmed that the gut microbiota’s efficiency in reducing nitrazepam to 7-aminonitrazepam is seven times greater than that of the liver [66]. A recent study utilizing a GF rat model for the global analysis of the physiological toxicokinetics of brivudine (a pharmaceutical) reported that 70% of the compound’s transformation could be attributed to microbial processes [29]. In addition, a toxicokinetic model demonstrated that the gut microbiota contributed 44.0% and 18.4% to the metabolism of total and inorganic arsenic, respectively, in zebrafish [99]. However, current toxicokinetic studies remain insufficient in fully characterizing the contribution of the gut microbiota to pollutant ADME, thereby limiting the toxicological understanding of the microbiota’s role in the context of pollutant exposure. Given the variable metabolic activity of the gut microbiota toward different pollutants and their inherent variability, this contribution is influenced by multiple factors, necessitating careful interpretation of existing data or context-specific application. Advanced models that integrate environmental complexity are imperative for evaluating the long-term toxicological impacts of gut microbiota-driven pollutant biotransformation in real-world exposure scenarios.

## Major factors driving the gut microbial biotransformation

The intricate interplay among pollutants, the gut microbiota, and host physiology underscores the complexity of gut microbiota-driven pollutant transformation. To systematically deconstruct this complexity, our analysis progresses across three tiers: (i) pollutant structural and toxicological determinants of microbial susceptibility, (ii) the enzymatic machinery across bacterial phyla and their metabolic interplay with hosts, and (iii) host-specific modulators shaping microbial catalysis. This tripartite framework offers a unified perspective on how microbial pollutant transformation emerges from the confluence of chemical properties, microbial ecology, and host biology.

### Pollutant effects on microbial transformation

The biotransformation potential of a chemical largely depends on its intrinsic structure and properties, which determine its resistance to transformation and ability to interact with specific enzymes [32, 36]. Multidimensional scaling clustering was employed to categorize the chemicals into groups of structurally similar compounds (SI-1; *n* = 125; Supplementary Table S7). This similarity-based clustering yielded 63 distinct clusters. To streamline the subsequent analysis, all clusters containing fewer than three molecules were consolidated into a single cluster, designated Cluster 14 (Supplementary Fig. S5A). Consequently, certain clusters could be associated with specific transformation pathways (Supplementary Fig. S5B). Clusters 8, 10, and 12 are readily distinguishable by the presence of an azo group. The azo group is a key structural feature conducive to specific azo reduction reactions. For the compounds in Clusters 1 and 4, which encompass majority of nitro-PAHs, nitro reduction emerges as the most prevalent transformation reaction. In Cluster 1, chemicals lacking specialized functional groups are more likely to undergo oxidation or the removal of functional groups. Epoxy-containing compounds in Cluster 2 trigger de-epoxidation reaction, whereas halogenated compounds in Cluster 3 undergo dehalogenation. The Phase II conjugates in Clusters 9 and 13 primarily undergo glycosyl or glucuronosyl cleavage. Chemicals in Clusters 5, 6, and 11, which possess C-O, C-N, C-S, or ester bonds, are prone to hydrolysis reactions, including dealkylation and deacylation, as well as N-dealkylation. Overall, this analysis indicates that pollutant transformation by the gut microbiota is highly structure specific. Certain substructures, such as glycosidases-related groups, esters, nitro, azo, alkene, keto, N-oxide, sulfoxide, and epoxy substructures, increase the susceptibility of pollutants to microbial transformation.

Pollutants can exert dose-dependent constraints on biotransformation via direct toxicity to the gut microbiota. Antibiotics, as the most disruptive pollutants, can induce acute microbial suppression, compositional shifts, and metabolic capacity changes [128, 129]. Similarly, exposure to non-antibiotic, including pharmaceuticals and environmental chemicals, may disrupt enzymatic pathways (e.g. azoreductases and β-glucuronidases) critical for pollutant transformation, leading to the accumulation of toxic intermediates and compromised detoxification [130]. Polybrominated biphenyls (BDE-209) and perfluorooctane sulfonate reduce microbial diversity and short-chain fatty acid production in both carp and infants [131, 132], whereas digoxin enhances reductase activity and pollutant transporter expression [130]. Furthermore, exposure to dioxins and heavy metals has been linked to increased microbial transformation (and/or resistance) genes, as demonstrated by shotgun metagenomic analysis of 359 Italian subjects across contamination gradients [133]. This transformation capacity erosion illustrates a cascading effect within “One Health” frameworks, wherein antibiotics and other microbiologically toxic pollutants diminish the gut microbiota-driven transformation, thereby magnifying the adverse impacts of other pollutants. Within this framework, understanding how antibiotic and non-antibiotic pollutants alter the gut microbial function is vital not only for elucidating the gut microbiota-driven transformation to mixed pollutants but also for revealing the bidirectional interplay between pollutants and the gut microbiota.

### Microbial mechanisms of transformation

**Figure 2:**
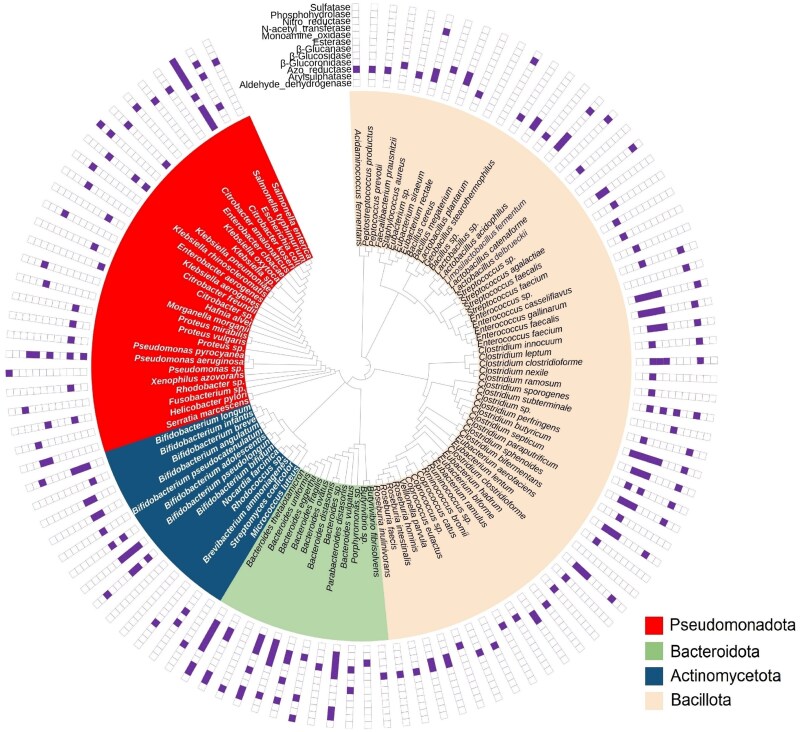
Phylogenetic tree showing the isolated gut bacterial strains with xenobiotic-metabolizing enzyme (XME) activities. The phylogenetic relationships among the reported bacterial strains were extracted from the National Center for Biotechnology Information (NCBI) taxonomic database classification system. The data points plotted outside the tree represent the xenobiotic metabolism ability of each species.

One of the goals in gaining a mechanistic understanding of microbial transformation is identifying and classifying the functions of key microbes. We compiled information for 109 isolated gut microbes known to secrete core XME families: hydrolases, oxidoreductases, transferases, and lyases (Fig. 2; Supplementary Table S8). Overall, the xenobiotic-metabolizing microbes appeared to be dispersed across four major bacterial phyla (Bacillota, Pseudomonadota, Actinomycetota, and Bacteroidota). The phylum Bacillota was the most frequently observed phylum of XME microbes in these datasets, consisting of the genera *Clostridium* (25.4%), *Eubacterium* (14.5%), *Lactobacillus* (9.1%), and *Enterococcus* (9.1%). *Bifidobacterium* (69.2%) is the most common genus in the phylum Actinomycetota and is characterized by the activities of azo reductase, nitro reductase, β-glucuronidase, and arylsulfatase. The genera *Bifidobacterium* (in Actinomycetota), *Bacteroides* (in Bacteroidota), *Citrobacter* (in Pseudomonadota), *Klebsiella* (in Pseudomonadota), and *Escherichia* (in Pseudomonadota) are also possible sources of additional xenobiotic-metabolizing bacteria. *E. coli*, the gut species with the highest XME activity, is capable of catalyzing the hydrolysis, reduction, oxidation, and transfer reactions of xenobiotics [107, 134, 135]. However, the “bacteria-centric” perspective presented herein reflects the current state of the literature rather than disregarding the potential significance of other microbiota components, and the roles of archaea, fungi, and viruses in pollutant transformation should not be overlooked. For example, the archaeal genus *Methanobrevibacter* represents a primary source of cellulases in the gastrointestinal tract of ruminants [136, 137], whereas the human gut archaea such as *Methanosphaera stadtmanae* and *Methanobrevibacter smithii* have demonstrated methyltransferase activity toward arsenic (As) [58, 59].

Although the isolation method provides direct insights into the gut strains involved in pollutant transformation, it clearly overlooks those uncultured gut microbes and the microbial interactions. In a previous study of XMEs in human gut microbiota (397 gut metagenomes) using metagenomic approaches, 850 bacterial genera were found to encode at least one XME (including CYP, monoamine oxidase, epoxide hydrolase, alcohol and aldehyde dehydrogenase, thiopurine methyltransferase, *N*-acetyl transferase, and glutathione *S*-transferase) [136]. In many cases, the diversity of the gut microbiota leads to a greater variety of microbial enzymes, and the combination of synergistic, beneficial, and antagonistic interactions among members of the gut microbiota may have a significant effect on the microbial transformation of pollutants in the digestive system [137]. Horizontal gene transfer events among the gut microbiota can also expand their substrate utilization range, enhance fermentation capacity, and provide new electron transfer pathways, thereby improving adaptation to anaerobic environments and increasing pollutant transformation efficiency [138–140]. Metagenomics has enabled the discovery of previously unrecognized enzymes, reactions, and organism-specific pathways by systematically expanding the catalog of xenobiotic-metabolizing pathways beyond culturable species [133]. Therefore, this approach is preferable for clarifying the diverse genes and pathways expressed by poorly represented microbes, the interactions of transformation-associated microbes with other microbes, and their adaptive evolution to pollutants on the basis of microbial co-occurrence in future studies.

### Host effects on the microbial transformation

The gut morphology, physiology, and function exert a combined influence on the colonization of the gut microbiota (Box 2), which reflects adaptations to ecological niches and environmental conditions (e.g. pH, lumen flux, and peristalsis). There is little microbial colonization in the stomach due to the extremely acidic environment (pH 1.5), whereas the density of the gut microbiota increases significantly in the distal small intestine (also known as the ileum) and colon [5, 141]. Another gradient of microbial distribution can be found along the tissue–lumen axis, wherein few bacteria adhere to the tissue or mucus but many bacteria are found in the lumen [142, 143]. Physiological characteristics of the host’s digestive system are also important determinants of the pollutant retention time and their opportunities in contact with the gut microbiota. The slow dietary transport and abundant microbial colonization make the distal intestine an ideal site for pollutant transformation [5, 144, 145]. For example, the extended digesta retention time in ruminants also allows for more extensive microbial transformation of complex and persistent pollutants [146]. In addition, vertebrate microbial stability enables predictable pollutant transformation efficiency, whereas the structural simplicity and environmental sensitivity of invertebrate guts may lead to high variability in transformation pathways and outcomes across individuals [147].

**Box 2:** Distribution characteristics of the microbiota in the gut systems of humans and other animals.

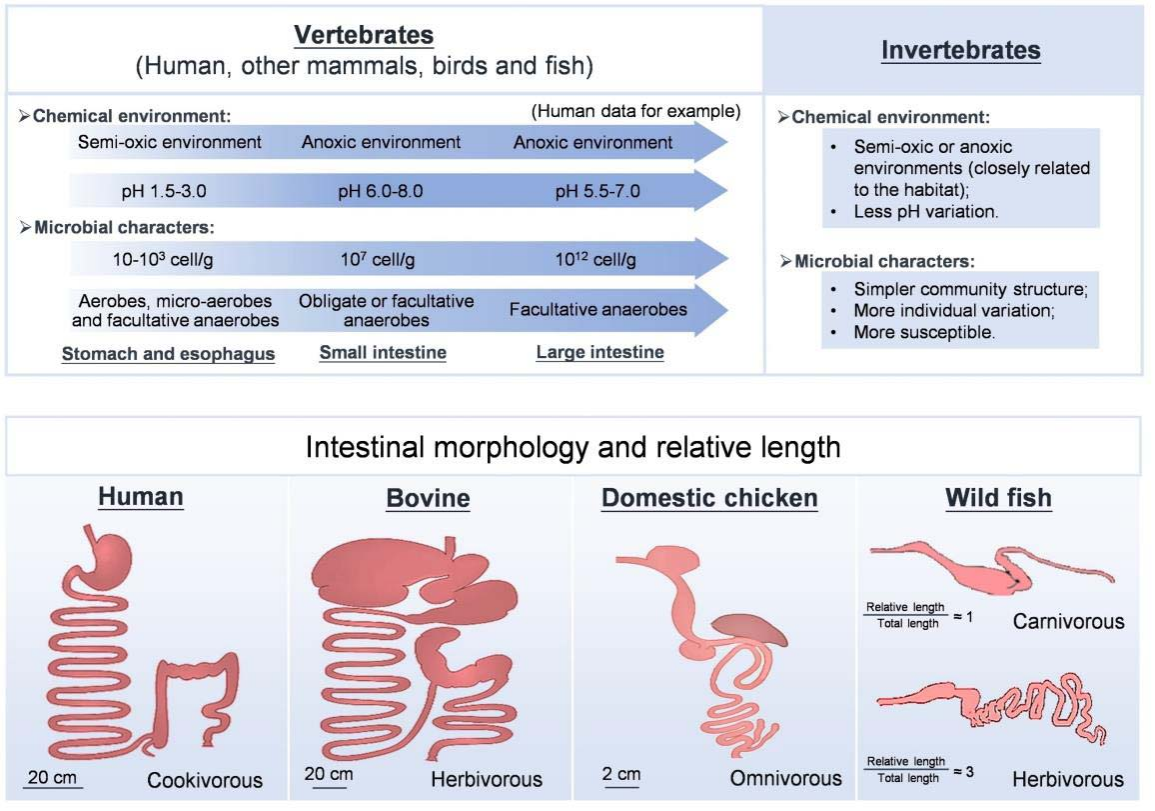
Spatial heterogeneity of both chemical conditions and microbial signatures varies along the digestive tract [148]. In humans, the mucosal surface area of the gastrointestinal (GI) tract is ~ 200–300 m^2^, harboring various microbes, including 10^13^–10^14^ cells of 400 different microbial species and subspecies [149]. Anaerobic bacteria are the main components of the stable intestinal microbiota, and their numbers are 100 to 1000 times higher than those of aerobic bacteria and facultative anaerobes [150]. The general ranking for bacterial number in human tissues from high to low is as follows: large intestine > small intestine > duodenum and stomach [5, 141]. In addition, the core gut microbes across vertebrate species are relatively stable including bacterial phyla of Actinomycetota, Bacillota, Bacteroidota, Pseudomonadota, Fibrobacterota, Fusobacteriota, and Verrucomicrobiota, and other microbes of fungi, archaea, protozoa, and viruses [151, 152], while > 98% of isolated genetic sequences present in the gut come from bacteria in vertebrates [38, 153]. In invertebrates, the tube-like gut provides a semi-oxic or anoxic environment with stable moisture conditions and nutrient pools from the foregut to the hindgut, which facilitates colonization through facultatively anaerobic bacteria [44]. In contrast, the simple invertebrate digestive system induces environmentally susceptible gut microbiota with high interindividual variability and no consistent core microbiome [44]. All vertebrates, including fish, mammals, and humans, have relatively complex but different gut systems [154–156], which equipping them with distinct digestive capacities (the comparative gut structures illustrated were redrawn based on anatomical data from the cited literatures). The average total intestinal transit time for healthy adults is 70 h (in the range of 23–168 h), with a majority (~80%) of the time spent in the large intestine (colon) rather than other sections [5, 144, 145]. Ruminant mammals (e.g. bovine) have developed a downstream stomach or hindgut, thereby extending the digesta retention time in the hindgut by as long as 96 h [157]. In fish, the gut evacuation time measured *in situ* generally ranges from 6 to 86 h [158–160], with herbivorous species exhibiting extended digestion periods.

The composition and activity of the gut microbiota are influenced by a combination of various internal factors (such as the host’s sex, lifestyle, and genetic characteristics) and external factors (such as diet) [30, 37, 38]. Thus, the physiological functions and lifestyle habits of the host inevitably affect the gut microbial community and enzyme activity. In a recent large-scale metagenome analysis, community similarity across hosts was determined basis of the initial inoculum and niche-specific factors such as the oxygen level, temperature, pH, and organic carbon availability rather than the phylogenetic relatedness of the hosts [30]. Taking digestive enzymes as an example, there are visible differences in the composition of gut microbes with digestive enzyme activity across host species from humans, mammals, birds, amphibians, and fish to invertebrates (Supplementary Table S9). The activity levels of lipases, proteases, and trypsases in the gut microbiota of carnivorous animals are much higher than those in herbivorous species [161]. In omnivorous animals, the microbiome in the gut tract mainly consists of protein-hydrolyzing bacteria and starch-, lipid-, and cellulose-decomposing bacteria [161]. Feeding ruminants a starch-free diet allows the gut microbiota to require almost no intestinal amylase to function but results in the presence of a series of cellulose degraders in the rumen [162]. Moreover, amylase activity in the digestive tract of omnivorous fishes is generally greater than that in the digestive tract of carnivorous fishes [163]. Previous research revealed differences in the metabolism of both endogenous and xenobiotic substances in the enriched gut microbiota of six marine species caused by differences in their feeding habitats [113, 164]. Therefore, the understanding of the effects of the host’s physiological signatures and feeding habits on the gut microbiota must be further improved to account for variations in the microbiota in response to pollutants, especially in comparisons across species. Such interspecific differences in gut anatomy and microbial function underscore the importance of considering host-specific gut models when predicting the biotransformation and toxicological consequences of pollutants.

## Calling for novel methodologies for elucidating gut microbiota-driven transformation

**Figure 3:**
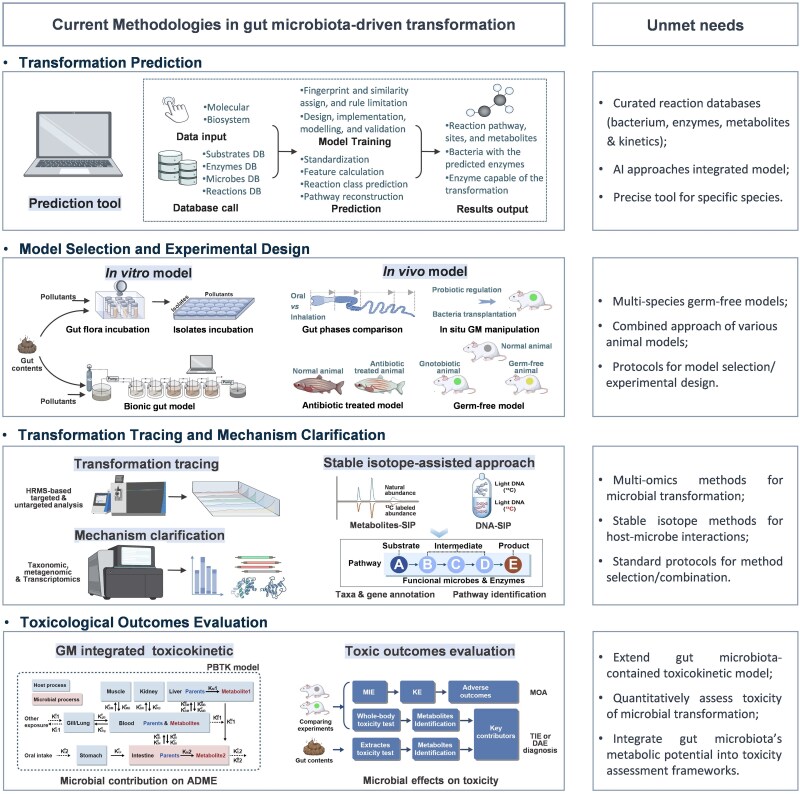
Existing methodologies and development directions for gut microbiota-driven pollutant transformation. Abbreviations: DB, database; AI, artificial intelligence; GM, gut microbiota; HRMS, high-resolution mass spectrometry; SIP, stable isotope probing; PBTK, physiologically based toxicokinetics; ADME, absorption, distribution, metabolism, and excretion; MIE, molecular initiating event; KE, key event; MOA, modes of action; TIE, toxicity identification evaluation; EDA, effect-directed analysis.

Although significant advances in recent years have paved the way for a more comprehensive toxicological perspective of gut microbiota-driven pollutant transformation, the major challenge is that the existing methodologies may no longer be adequate for gaining mechanistic insights into the transformation process under complex “pollutant–gut microbiota–host” interactions (Fig. 3).

### Developing prediction tools for the gut microbiota-driven transformation of pollutants

Given the impracticality of experimentally investigating the gut microbial transformation of every environmental pollutant, computational prediction of transformation potential, scope, and specificity is essential. Reaction mining and machine learning approaches have led to tools such as BioTransformer [165], DrugBug [166], and GutBug [167], which predict potential microbial biotransformation pathways and enzyme classes (EC numbers) based on chemical structure. However, reliable prediction remains challenging due to the complexity and dynamic nature of the gut microbiome and the scarcity of high-quality training data across diverse conditions. Advances in high-throughput technologies are aiding the development of better-curated databases. For example, integrated genetic and metabolomic analyses have been used to identify gut microbes and enzymes involved in drug metabolism [3], whereas microbiome-derived metabolic screening combines culturing, metagenomics, and transformation assays to link genes to functions [4]. Although not specifically designed for gut pollutant transformation studies, numerous databases containing biological and chemical information can be leveraged to develop accurate predictive models through integration or as foundational resources, such as the metabolism pathway databases (e.g. KEGG, MetaCyc, and Reactome), enzyme-specific databases (e.g. CAZy, Pfam, and UniProt), chemical–enzyme interaction databases (e.g. ChEMBL), and protein–protein interaction databases (e.g. STRING). Especially, genomic context prediction methods (i.e. gene co-occurrence, co-expression profiles, and genomic neighborhood) as implemented in tools such as STRING can provide a powerful “guilt-by-association” approach for hypothesizing novel pathway components in non-model organisms [168]. Artificial intelligence and the subdomains machine learning and natural language processing, which integrate pathways, enzymes, metabolites, and kinetics from many biotransformation studies, are recommended for chemoinformatic and bioinformatic analyses. This could enable more precise tools to quickly screen for pollutant candidates prone to microbial transformation, which will support more finely targeted mining and the mechanistic analysis of associations between enzymes and specific reactions.

### Constructing advanced experimental models and selection guidelines for gut microbiota-driven transformation of pollutants

Current experimental models for studying gut microbiota-driven pollutant transformation include both *in vitro* and *in vivo* systems, each with distinct advantages and limitations in replicating the gut physiology and microbial complexity. *In vitro* models, such as batch cultures with fecal samples [169], are accessible and ethically advantageous but are limited by the poor culturability of many gut species [170–172]. Continuous culture simulators (e.g. SHIME [59, 62, 89], the simulation of the mammal intestinal microbial ecosystem [173], and the fish digestion model [158]) can better mimic intestinal conditions and enable direct observation of microbial metabolism, but they still struggle to fully replicate critical gut features such as pH, redox potential, and microbial diversity. *In vivo* gut microbiota manipulation models, including germ-free (GF) animals, antibiotic-treated models, and fecal microbiota transplantation (FMT) models, can provide direct functional insights but face translational and practical challenges [5, 174, 175]. GF rodents are relatively ideal models but still exhibit anatomical and metabolic abnormalities and limited human extrapolation [5]. They also require stringent maintenance and exhibit morphological and functional abnormalities such as significant cecal enlargement and reduced absorption efficiency [5]. Among aquatic species, only GF zebrafish at the larval stage are available and widely used as a model for gut microbiome research [99, 158]. In addition, the FMT model is applicable only for studying specific bacteria upon successful colonization. To enhance reproducibility and biological relevance, future efforts should focus on developing multi-species gnotobiotic models, humanized microbiota mouse models, and integrated systems that combine *in vitro* realism with *in vivo* validity. Novel technologies such as intestinal organoids, 3D cell cultures, or gut-on-a-chip systems offer more accurate biological replication [176], enabling simulation of host responses and supporting reliable *in vitro–in vivo* extrapolation. There is also a growing need to develop standardized selection guidelines that consider research objectives, pollutant properties, and the microbial ecological context to facilitate model comparison and data integration.

### Integrating technologies for the description and tracing of transformation mechanisms

Elucidating the complex interactions among microbial consortia and host cells during gut-driven pollutant transformation requires interdisciplinary strategies integrating microbiology, analytical chemistry, omics technologies, and computational biology. This requires interdisciplinary approaches that combine molecular biology, analytical chemistry, laboratory experiments, and data science [177]. Microbiological techniques, such as conventional culture, single-cell sequencing, metabarcoding, metagenomics, transcriptomics, proteomics, metabolomics, and their combinations, are essential for studying composition and function of the gut microbiota [19, 175]. The study of the enzymes and metabolic pathways of known gut microbes can also provide clues to support microbiome discoveries. Integrating multiomics data provides genetic insights into transformation enzymes, while combining omics with microbial metabolic network analysis helps clarify microbial relationships during metabolism.

Although advanced chemical analysis can detect trace levels of gut metabolites, host-microbe-metabolite interactions remain difficult to characterize. Stable isotopes (e.g. ^13^C, ^15^N, and ^18^O) help trace low-level or cometabolic activities. The preferential breakage of bonds for light isotopes and heavy isotopes (^13^C) results in a gradual increase in the enrichment of heavy isotopes in the parent compound compared with the metabolites [178]. Compound-specific isotope analysis examines isotope ratios to identify unique biotransformation pathways and fractionation-causing reactions [179], thereby distinguishing pollutant transformation from host–microbe interactions. Stable isotope-assisted metabolomics (SIAM) enables sensitive, untargeted detection of isotope-labelled pollutant metabolites, enhancing high-resolution mass spectrometry for unknown metabolite characterization [180, 181]. SIAM can be used to track isotopic labels globally without preconceptions about pollutant fate [182]. Stable isotopes can also link transformation activities with specific microbial populations and assimilation mechanisms. Stable isotope probing (SIP) methods, including DNA-SIP, metagenomic-SIP, transcriptome-SIP, protein-SIP, and single-cell SIP, provide sequence and functional data on potential transformation agents by monitoring labelled atom incorporation into nucleic acids [183, 184]. Time-dependent ^13^C-metabolite flux analysis (^13^C-MFA) traces isotopic ratios in key metabolic pathways, revealing gut transformation processes [185]. For example, ^13^C-MFA successfully elucidated the microbial fermentation of ^13^C-labelled carbon nanomaterials in mice [87], demonstrating its ability to uncover *in situ* gut symbiotic metabolism. These integrative, isotope-enabled approaches allow mechanistic exploration of pollutant transformation at the molecular, cellular, and ecosystem levels, offering a powerful framework for future gut microbiome research.

### Advancing toxicological assessment approaches for gut microbiota-driven pollutant transformation

Despite early hints of its pollutant-transforming role, the gut microbiota’s direct toxicity-related interactions with pollutants, drugs, and xenobiotics may represent just the tip of the iceberg [28, 186]. Beyond these direct impacts (e.g. “first-pass metabolism” and “enterohepatic circulation”), two key non-transformation mechanisms also influence pollutant bioavailability: (i) changes in bioavailability (e.g. gut mucosal barrier alterations or microbiota-mediated pollutant adsorption) [187] and (ii) interference with the host’s detoxification mechanisms [5, 188]. For instance, gut microbes can alter pollutant absorption by affecting mucosal integrity or through direct adsorption. *Lactiplantibacillus plantarum* can reduce PAH uptake via surface binding [189], while some certain bacteria can deplete pharmaceuticals through bioaccumulation without structural modification [190]. Comparative studies in germ-free versus colonized animals have further revealed microbiota-induced changes in host gene expression related to xenobiotic metabolism in the gut and liver [191, 192], highlighting the microbiome’s systemic influence on toxicological responses.

Toxicological assessments must be integrated into gut microbiota transformation studies. For example, product identification coupled with toxicity assessments (i.e. effect-directed analysis and toxicity identification evaluation) can reveal key mechanisms in *in vitro* gut microbiota models. *In silico* tools such as quantitative structure–activity relationships, quantitative cationic–activity relationships, quantitative structure–nanotoxicity relationships, and structural can be employed to predict the toxicity of gut metabolites. In addition, the application of a physiologically based toxicokinetic (PBTK) model incorporating gut microbial transformation can clarify associations between the microbiota and *in vivo* pollutant toxicokinetics. PBTK models can be developed for GF or antibiotic-treated animal comparisons to describe both microbiota- and host-driven transformation processes [29]. These approaches facilitate the elucidation of the microbially mediated toxicological effects of pollutants by providing a quantitative framework for understanding the role of the microbiota in pollutant toxicokinetics. Given the tremendous progress in biochemistry, microbiology, and toxicology, a comprehensive interdisciplinary methodological framework for the more in-depth exploration of the role of the gut microbiota in toxicology is urgently needed.

## Conclusions and prospects for progress

**Figure 4:**
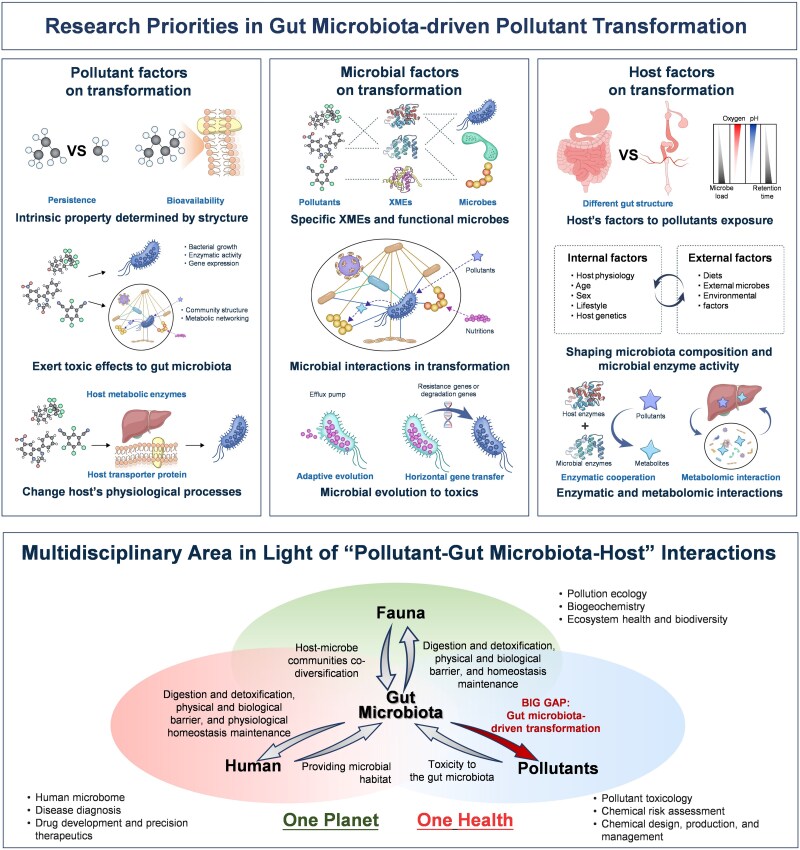
Key goals for investigating gut microbiota-driven transformation and multidisciplinary research in light of “pollutant–gut microbiota–host” interactions.

The transformation of environmental pollutants by the gut microbiota greatly impacts the effects of exposure to and the toxicology of these pollutants in humans and animals. This review summarizes the emerging understanding of the contributions of the gut microbiota to pollutant transformation, covering a wide range of host species and chemical classes. The main transformation reactions that can be driven by the gut microbiota include reduction, hydrolysis, functional group removal, and oxidation. These reactions are connected to the metabolic enzymes that have developed in the gut microbiota, allowing them to directly transform specific pollutants and regulate their MOA and ADME properties in the host. In addition, this review provides an overview of existing methodologies and future development directions to systematically elucidate gut microbiota-driven transformation and its toxicological consequences. The key to the successful investigation of gut microbiota-mediated transformation is to efficiently combine transformation consequences with microbial and compound-specific features, which requires highly multidisciplinary research dedicated to elucidating “pollutant–gut microbiota–host” interactions (Fig. 4). In the opinion of this research group, the following key issues should be addressed in the future:

- Pollutant transformation by gut microbial communities involves both dose effects and mixture effects. Future studies should clearly identify the long-term toxic effects and threshold concentrations for specific compounds and the co-metabolism of complex pollutant mixtures in the assessment of the effects of the host and gut microbiota on environmental pollutants in the real world.
- Owing to the complex structure of gut microbial communities, future studies should systematically explore the functional implications of bacteria and enzymes involved in transformation processes, the mechanistic characterization of interactions among bacteria, and the influence of ecosystem-wide changes in the microbiome on transformation outcomes.
- Considering that pollutant transformation can be recognized as a combined host–microbiota process, a systems biology view of host–microbiota interactions should be established for pollutant transformation mechanisms, including microbial modulation, enzymatic cooperation, and metabolomic interactions.

Recognizing the gut microbiota’s pivotal role as a universal modifier of pollutant exposure underscores the need to integrate “pollutant–gut microbiota–host” interactions into the“One Planet, One Health” concept. However, gut microbiota-driven transformation remains an underexplored research frontier and is often overlooked due to its inherent complexity and interdisciplinary nature. First, insights into gut microbiota-mediated pollutant transformations have led to advancements in chemical design, pollutant management, risk assessment, and toxicology. Beyond this, characterizing population-specific microbial metabolic functions could significantly enhance environmental health research, drug development, precision medicine, and other transdisciplinary fields aligned with the “One Planet, One Health” notion. Ultimately, given the potential relationships between healthy host microbiota relationships at the micro level and ecosystem health and homeostasis at the macro level, understanding gut microbial transformation holds promise as a path for ecological research on ecosystem changes and biodiversity loss across all ecosystems on Earth in the context of the Anthropocene.

## Supplementary Material

ISME-SI-Supplementary_Materials-202409_wraf215

## Data Availability

All data are available in the main text or the supplementary materials.
