# Supplementary material for: Deciphering the universal role of gut microbiota in pollutant transformation
Source: ISME J. 2025 Sep 30;19(1):wraf215. doi: 10.1093/ismejo/wraf215 (PMC12516962; doi:10.1093/ismejo/wraf215)
Supplement: ISME-SI-Supplementary_Materials-202409_wraf215 [file isme-si-supplementary_materials-202409_wraf215.docx]

Supporting Information

**Deciphering the Universal Role of Gut Microbiota in Pollutant Transformation**

Rui Hou,^a,1^ Xiaowei Jin,^b,1^ Jingchun Feng,^a,c,j,*^Jingchuan Xue,^a,j^ Chengzhi Chen,^d^ Yuanqiang Zou,^e^ Xiangrong Xu,^f,*^ Kefu Yu,^c,f^ Pei-Yuan Qian,^c,g^ Wei Zhang,^h^ Jizhong Zhou,^i^ Si Zhang,^c^ Zhifeng Yang^a,c,j^

^a^ Guangdong Provincial Key Laboratory of Water Quality Improvement and Ecological Restoration for Watersheds, School of Ecology, Environment and Resources, Guangdong University of Technology, Guangzhou 510006, China

^b^ China National Environmental Monitoring Centre, Beijing 100012, China

^c^ Southern Marine Science and Engineering Guangdong Laboratory (Guangzhou), Guangzhou 511458, China

^d^ Department of Occupational and Environmental Health, School of Public Health, Chongqing Medical University, Chongqing 400016, China

^e^ BGI Research, Shenzhen 518083, China

^f^ Guangxi Laboratory on the Study of Coral Reefs in the South China Sea, Coral Reef Research Centre of China, School of Marine Sciences, Guangxi University, Nanning 530004, China

^g^ Department of Ocean Science, The Hong Kong University of Science and Technology, Hong Kong, China

^h^ Centre for Marine Bioproducts Development, College of Medicine and Public Health, Flinders University, Adelaide South Australia 5001, Australia

^i^ Institute for Environmental Genomics, University of Oklahoma, Norman, USA

^j^ Key Laboratory of City Cluster Environmental Safety and Green Development, Institute of Environmental and Ecological Engineering, Guangdong University of Technology, Guangzhou 510006, China

^1^ These authors contributed equally to this work.

*Correspondence: [fengjc@gdut.edu.cn](mailto:fengjc@gdut.edu.cn) (Jingchun Feng) and xuxr@gxu.edu.cn (Xiangrong Xu)

**SI-Text S1 Clustering of the selected pollutants based on structure similarity.**

We performed a classification of environmental pollutants with clear transformation pathway based on their chemical structure similarity (n = 125, **Table S7**). The ChemMine Tool provide an online service for analyzing and clustering small molecules.[1] In this service, the maximum common substructure (MCS) algorithm and the Tanimoto coefficient were used to calculate structural similarity between two molecules. The Tanimoto coefficient is defined as the following equation:

$Tanimoto= \frac{c}{a + b - c}$ (1)

where a and b are the total number of unique features of the two molecules and c is the number of common features in their MCS.[2] In ChemMine Tool, the structure data files (SDF) of the selected compounds were used as input values to generate atom pair fingerprints and calculation of Tanimoto coefficient. We used binning clustering algorithm with a similarity cutoff (Tanimoto coefficient) of 0.4 for the analysis. Based on Tanimoto similarity measure between structures, multidimensional scaling (MDS) clustering method was performed for classifying the compounds.

**Figure S1.** Major transformations of persistent organic pollutants (A), pesticides (B) and other legacy organic pollutants (C) mediated by the gut microbiota and their toxicity consequences. The green boxes display representative compounds with similar transformation pathways. The gray boxes show the common toxicity consequences of the transformation type. For a comprehensive list of transformations, see Supplementary Information Table S1.


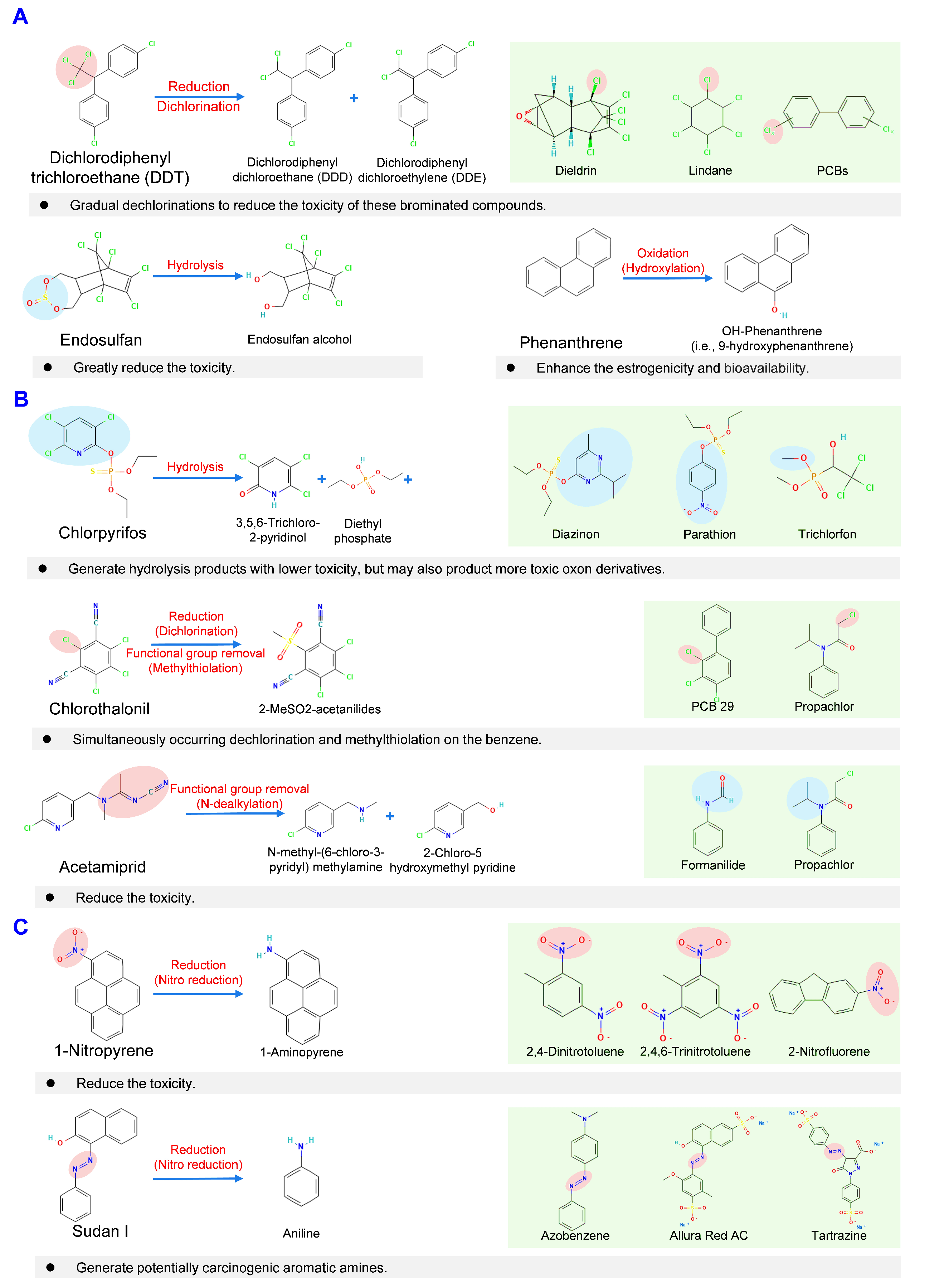


**Figure S2.** Major transformations of As (A) and other metals (B) mediated by the gut microbiota and their toxicity consequences. The gray boxes show the common toxicity consequences of the transformation type. For a comprehensive list of transformations see Supplementary Information Table S2.


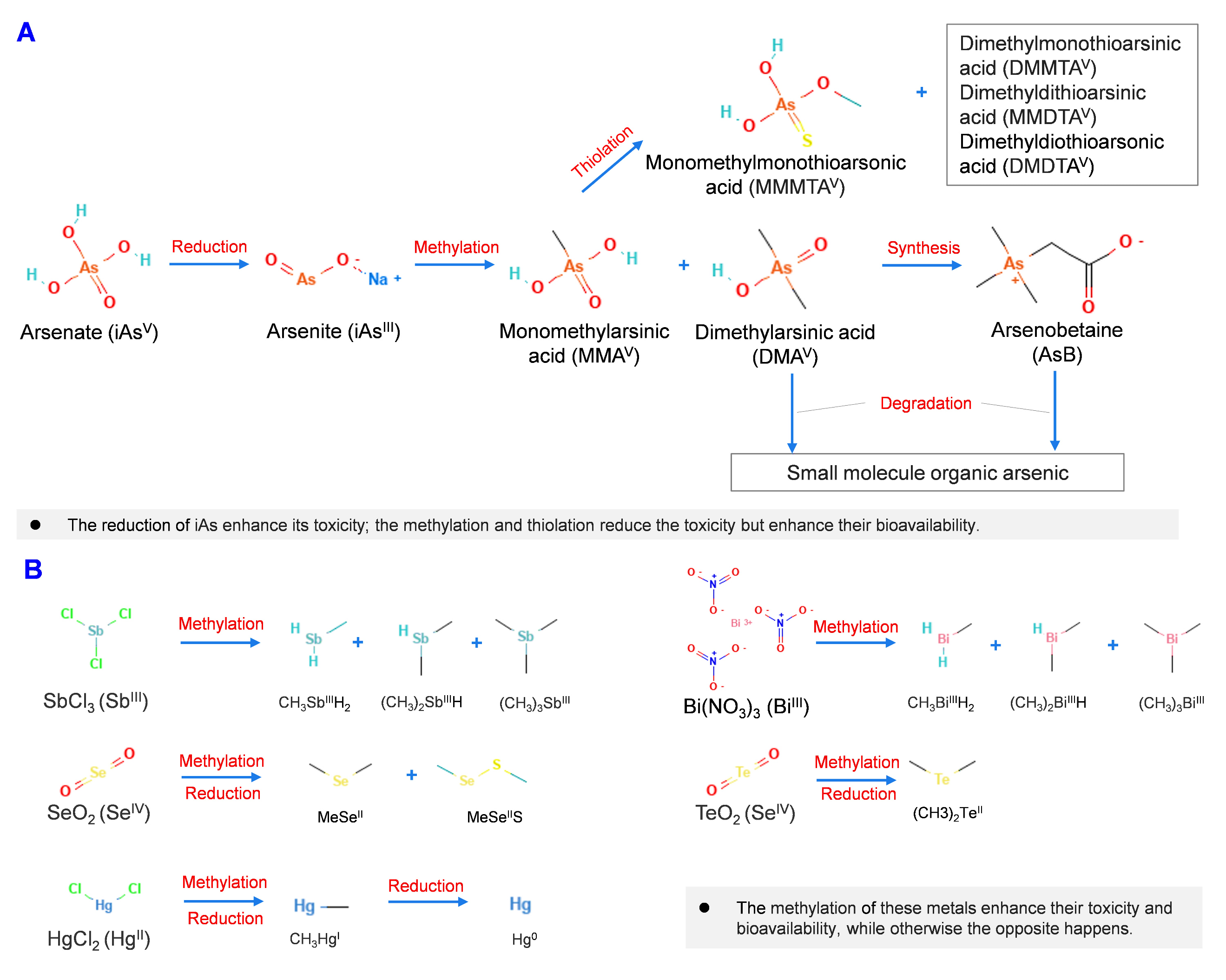


**Figure S3.** Major transformation types of PPCPs are mediated by the gut microbiota and their toxicity consequences. The reaction sites are highlighted on the molecular structures. The green boxes display representative compounds with similar transformation pathways. The gray boxes show the common toxicity consequences of the transformation type. For a comprehensive list of transformations, see Supplementary Information Table S3.


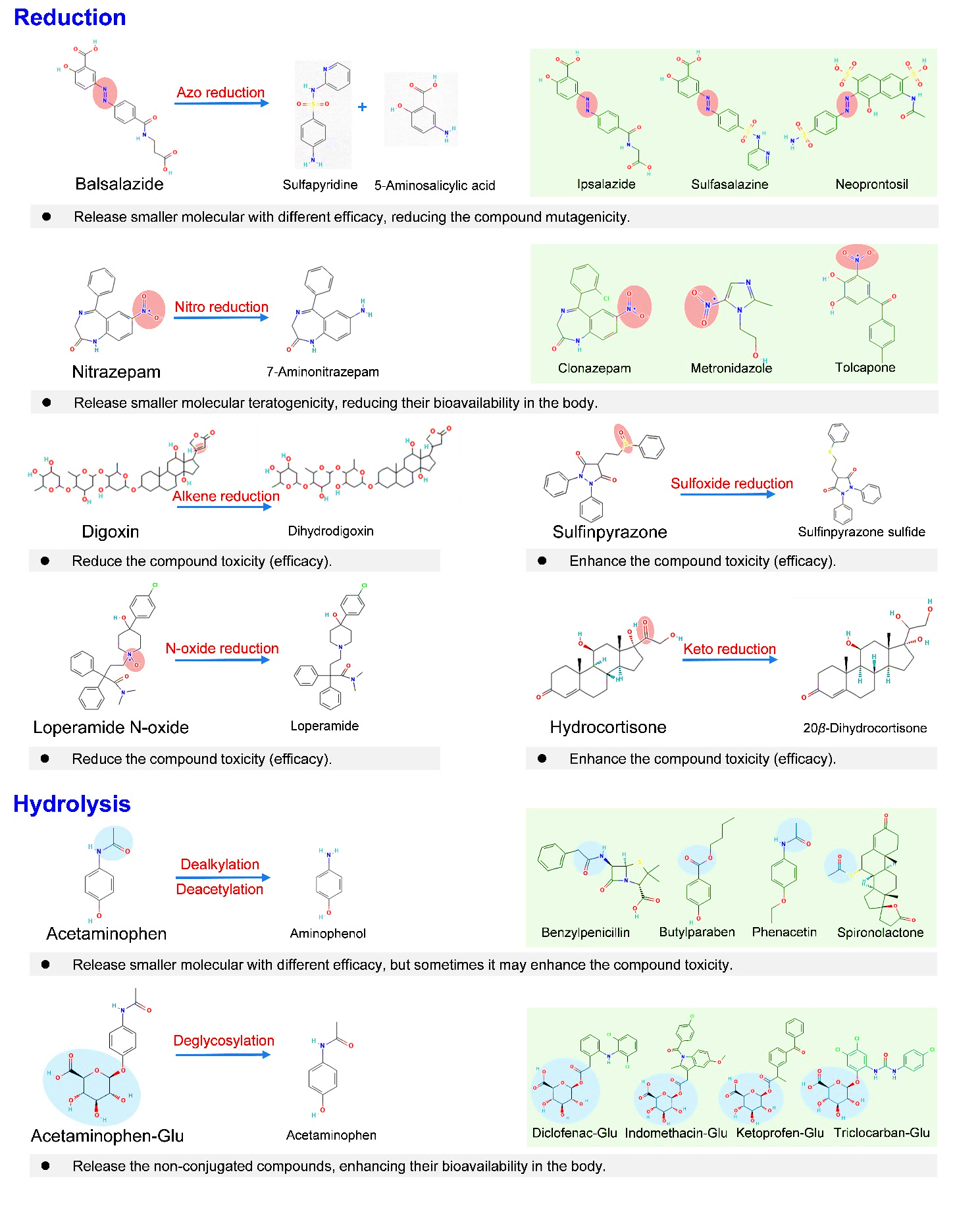


**Figure S4.** Major transformation types of other emerging pollutants (A) and mycotoxins (B) mediated by the gut microbiota and their toxicity consequences. The reaction sites are highlighted on the molecular structures. The green boxes display representative compounds with similar transformation pathways. The gray boxes show the common toxicity consequences of the transformation type. For a comprehensive list of transformations, see Supplementary Information Tables S4 and S5.


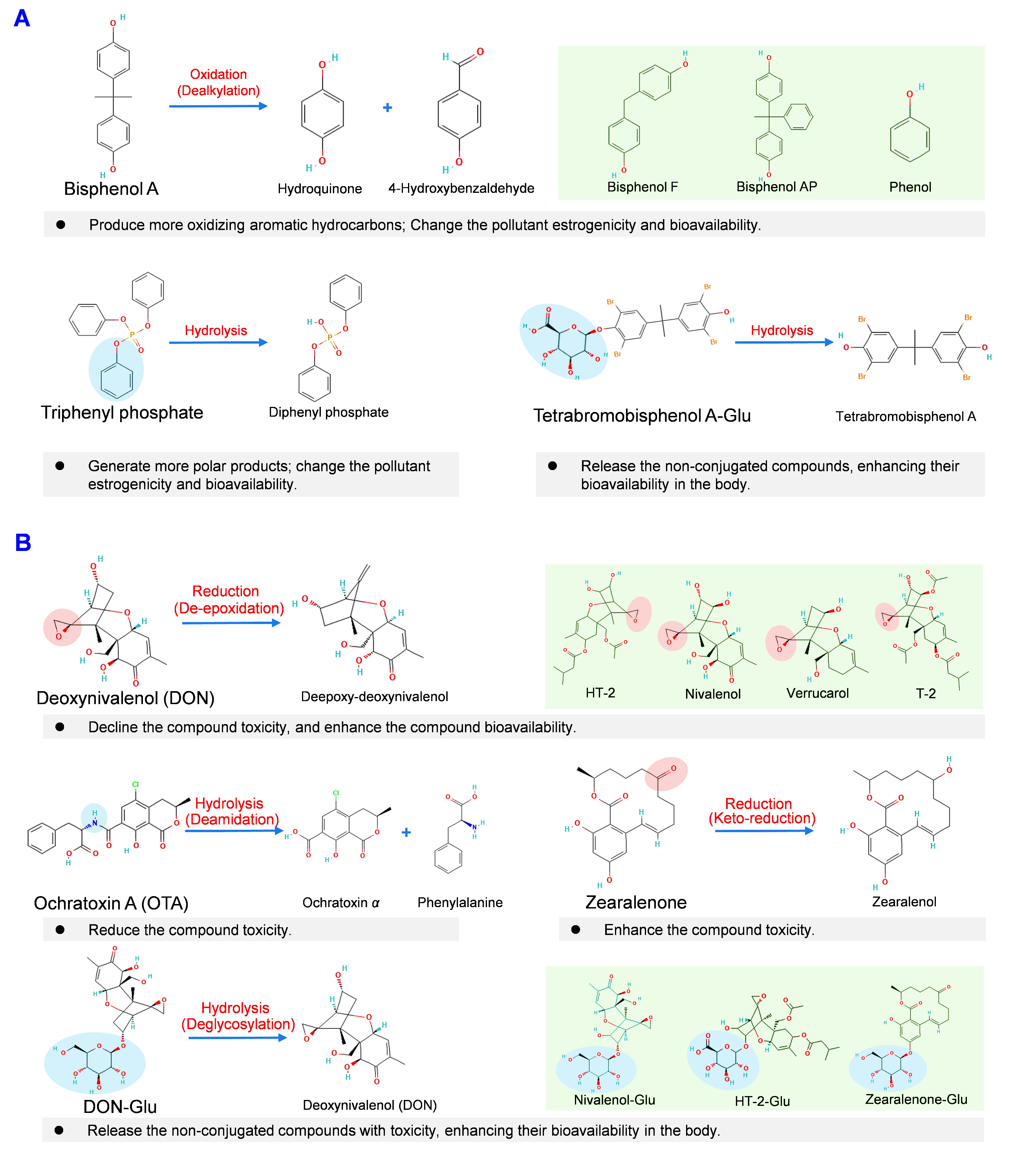


**Figure S5.** Structural similarity analysis of 125 environmental pollutants that can be transformed by the gut microbiota via a clear transformation pathway (A) and the reaction distributions involved in these transformations according to the different clusters (B). The pollutants were clustered using the multidimensional scaling (MDS) clustering method on the ChemMine tool online platform [1].


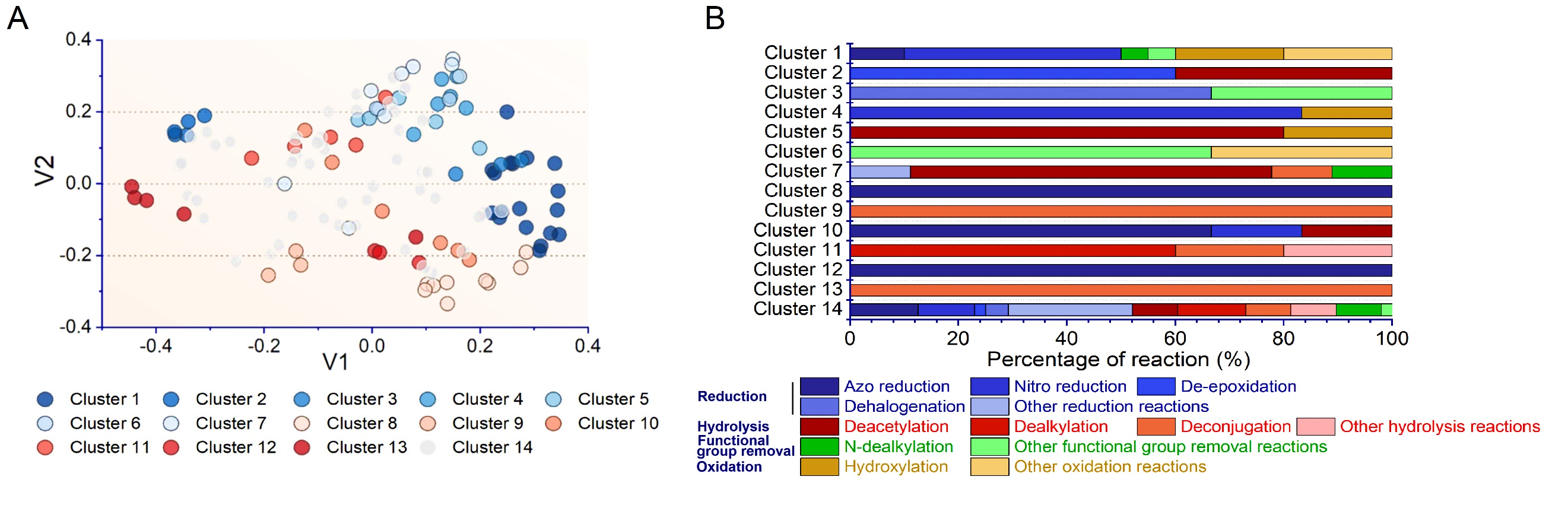


**Table S1.** Summary of transformation of persistent organic pollutants (POPs), non-POP pesticides, and other legacy organic pollutants by gut microbiota from human and fauna.

| **Name** | **Classification** | **Functional microbes** | **Hosts** | **Enzymes** | **Metabolites** | **Potential**  **consequences** | **Mechanisms** | **References** |
| --- | --- | --- | --- | --- | --- | --- | --- | --- |
| 2,4,5-Trichlorobiphenyl (PCB 29) | POPs-polychlorinated biphenyls (PCBs) | *-* | Mice | - | 4-MeSO_2_-trichlorobiphenyl | - | Functional group removal (Methylthiolation) | [3, 4] |
| Benzo[a]pyrene | POPs-polycyclic aromatic hydrocarbons (PAHs) | - | Human | - | 7-Hydroxybenzo(a)pyrene | Increase estrogenicity | Oxidation (Hydroxylation) | [5] |
| Dichlorodiphenyl trichloroethane (DDT) | POPs-DDT | *Proteus vulgaris* | Mice | - | Dichlorodiphenyl dichloroethane (DDD) | Decrease toxicity | Reduction (Dehalogenation) | [6] |
|  |  | *Escherichia coli.* and *Aerobacter aerogenes* | Rat | - | Dichlorodiphenyl dichloroethane (DDD) | Decrease toxicity | Reduction (Dehalogenation) | [7] |
| Hexachlorobiphenyl (PCB 153) | POPs-polychlorinated biphenyls (PCBs) | *Clostridium* beijerinckii | Human | - | Pentachlorobiphenyl | Decrease toxicity | Reduction (Dehalogenation) | [8] |
| Lindane | POPs-organochlorine pesticides (OCPs) | - | Rat | - | 3,4,5,6-Tetrachlorocyclohex-1-ene | - | Reduction (Dehalogenation) | [9] |
| Methoxychlor | Potential POPs-organochlorine pesticides (OCPs) | *Eubacterium limosum* | Human | - | Methoxydichlor | Decrease toxicity | Reduction (Dehalogenation) | [10] |
| Naphthalene | POPs-polycyclic aromatic hydrocarbons (PAHs) | - | Human | - | - | Increase estrogenicity | Oxidation (Hydroxylation) | [5] |
|  |  | - | Rat | - | Naphthol, 1,2-dihydro-1-hydroxy-2-S-cysteinyl naphthalene, 1,2-dihydro-1-hydroxy-2-S-(N-acetyl) cysteinyl naphthalene | - | Oxidation (Hydroxylation) and hydrolysis | [11] |
| Phenanthrene | POPs-polycyclic aromatic hydrocarbons (PAHs) | - | Human | - | - | Increase estrogenicity | - | [5] |
| Pyrene | POPs-polycyclic aromatic hydrocarbons (PAHs) | - | Human | - | 1-Hydroxypyrene | Increase estrogenicity | Oxidation (Hydroxylation) | [5] |
| Tetrachlorobiphenyl (PCB 77) | POPs-polychlorinated biphenyls (PCBs) |  | Human | - | Trichlorobiphenyl | Decrease toxicity | Reduction (Dehalogenation) | [8] |
| Dichlorodiphenyl trichloroethane (DDT) |  | *Rhodococcus Bacillus* | Earthworm | Dehalogenase and dehydrochlorinase | DDD, dichlorodiphenyl  -dichloroethylene (DDE) | Decrease toxicity | Reduction (Dehalogenation) | [12] |
| Dieldrin | POPs-organochlorine pesticides (OCPs) | *Pseudomonas melophthora* | Apple Maggot (*Rhagoletis pomonella*) | - | - | - | - | [13] |
| Endosulfan | POPs-organochlorine pesticides (OCPs) | *Rhodococcus* | Earthworm | - | Endosulfan diol | Decrease toxicity | Hydrolysis | [14, 15] |
| Chlorpyrifos | Pesticides-organophosphates | *Lactobacillus delbrueckii, Limosilactobacillus fermentum, Lactiplantibacillus plantarum, Escherichia coli, Enterococcus faecalis* | Human | Phosphatase | 3,5,6-Trichloro-2-pyridinol, chlorpyrifos-oxon, diethylphosphate | - | Hydrolysis (Dealkylation) | [16] |
| Chlorothalonil | Pesticides-organochlorine pesticides (OCPs) | *-* | Rat, dog, and human | - | 2,5,6-Trichloro-4-methylthioisophthalonitrile, 2,5,6-trichloro-4-thioisophthalonitrile, 3-thia-1-cyano-2,5,6-trichloroisoindolinone, 2,5,6-trichloro-4-hydroxy-isophthalonitrile, and 2,5,6-trichloroisophthalonitrile | - | Functional group removal (Methylthiolation) and reduction (Dehalogenation) | [17] |
| Propachlor | Pesticides-amides | *-* | Rat | - | 2-MeSO_2_-acetanilides | - | Functional group removal (Methylthiolation and N-dealkylation) and reduction (Dehalogenation) | [18-20] |
| Propachlor conjugates (Glutathione, cysteine, N-acetylcysteine, and S-oxide of the N-acetylcysteine) | Pesticides-amides | *-* | Pig and rat | - | Propachlor | Increase bioavailability | Hydrolysis (Deconjugation) | [21-23] |
| Acetamiprid | Pesticides-neonicotinoids | *Brucella intermedia* PDB13 | Earthworm | Nitrile hydratase (NHase) | N-methyl-(6-chloro-3-pyridyl) methylamine, 2-chloro-5 hydroxymethyl pyridine, and 6-Chloronicotinic acid | Decrease toxicity | Oxidation and functional group removal (N-dealkylation) | [24] |
| Carbaryl | Pesticides-carbamates | *Pseudomonas melophthora* | - | - | - | - | - | [13] |
| Chlorpyrifos | Pesticides-organophosphates | *S. maltophilia* | Silkworm | Phosphatase | - | - | Hydrolysis (Dealkylation) | [25] |
|  |  | *-* | Worm (*Nereis succinea*) | - | - | - | - | [26] |
| Chlorpyrifos ethyl | Pesticides-organophosphates | *Arthrobacter nicotinovorans, Delftia lacustris, Enterococcus mundtii, Leclercia adecarboxylata,* and *Enterococcus casseliflavus* | Fifth instars of *Spodoptera frugiperda* | - | - | - | - | [27] |
| Deltamethrin | Pesticides-pyrethroids | *Arthrobacter nicotinovorans,*and *Enterococcus casseliflavus* | Fifth instars of *Spodoptera frugiperda* | - | - | - | - | [27] |
| Diazinon | Pesticides-organophosphates | *Pseudomonas melophthora* | Apple Maggot (*Rhagoletis pomonella*) | - | - | - | - | [13] |
| Dichlorovos | Pesticides-organophosphates | *Pseudomonas melophthora* | Apple Maggot (*Rhagoletis pomonella*) | - | - | - | - | [13] |
| Diisopropyl phosphorofluoridate | Pesticides-organophosphates | *Pseudomonas melophthora* | Apple Maggot (*Rhagoletis pomonella*) | - | - | - | - | [13] |
| Imazalil | Pesticides-bio-pesticides | *Kaistobacter* | Earthworm | - | - | Decrease toxicity | - | [28] |
| Lambda-cyhalothrin | Pesticides-pyrethroids | *Pseudomonas stutzeri, Enterococcus mundtii,* and *Enterococcus casseliflavus* | Fifth instars of *Spodoptera frugiperda* | - | - | - | - | [27] |
| Lufenuron | Pesticides-benzoylureas | *Staphylococcus sciuri, Microbacterium arborescens,* and *Enterococcus mundtii* | Fifth instars of *Spodoptera frugiperda* | - | - | - | - | [27] |
| Parathion | Pesticides-organophosphates | *Pseudomonas melophthora* | Apple Maggot (*Rhagoletis pomonella*) | - | - | - | - | [13] |
| Spinosad | Pesticides-bio-pesticides | *Enterococcus casseliflavus, Enterococcus mundtii,* and *Enterococcus casseliflavus* | Fifth instars of *Spodoptera frugiperda* | - | - | - | - | [27] |
| Trichlorfon | Pesticides-organophosphates | *Citrobacter* sp. | Oriental fruit fly *Bactrocera dorsalis* (Hendel) | Phosphatase | - | Decrease toxicity | Hydrolysis (Dealkylation) | [29] |
| 1,3-Dinitrobenzene | Nitro-polycyclic aromatic hydrocarbons (NPAHs) | - | Rat | Nitro reductase |  | - | Reduction (Nitro reduction) | [30] |
| 1,3- and 1,6- Dinitropyrene | Nitro-polycyclic aromatic hydrocarbons (NPAHs) | *Eubacterium* sp.*, Clostridium leptum, Clostridium paraputrificum* and *Clostridium clostridiiforme* | Human | Nitro reductase | - | Decrease toxicity | Reduction (Nitro reduction) | [31] |
| 1-Nitro-7-ami-nofluorene | Nitro-polycyclic aromatic hydrocarbons (NPAHs) | *Clostridium perfringens* | Human | Nitro reductase | - | - | Reduction (Nitro reduction) | [32] |
| 1-Nitropyrene | Nitro-polycyclic aromatic hydrocarbons (NPAHs) | *Bacteroides fragilis, Eubacterium* sp.*, Clostridium leptum, Clostridium paraputrificum, Clostridium perfringens* and *Clostridium clostridiiforme* | Human | Nitro reductase | 1-Aminopyrene | Decrease toxicity | Reduction (Nitro reduction) | [31-34] |
|  |  | - | Rat | Nitro reductase | 1-Aminopyrene | Decrease toxicity | Reduction (Nitro reduction) | [35-37] |
| 2,4,6-Trinitrotoluene | Nitro-polycyclic aromatic hydrocarbons (NPAHs) | *Klebsiella* sp. *C1* | Human | Nitro reductase | 2-Amino-4,6-dinitrotoluene and 2,2′,6,6′-tetranitro-4,4′-azoxytoluene | - | Reduction (Nitro reduction) | [38] |
| 2,4-Dinitrotoluene | Nitro-polycyclic aromatic hydrocarbons (NPAHs) | - | Rat | Nitro reductase | 4-N-acetyl-2-nitrobenzoic acid and 2-amino-4-  nitrobenzoic acid | - | Reduction (Nitro reduction) | [39, 40] |
|  |  | - | Human | Nitro reductase | 2-amino-4-nitrotoluene, 2-nitro-4-  Aminotoluene and 2,4-diaminotoluene | - | Reduction (Nitro reduction) | [41] |
| 2-Amino-3-methylimidazo [4,5-f] quinoline | Nitro-polycyclic aromatic hydrocarbons (NPAHs) | *Bacteroides thetaiotaomicron, Clostridium perfringens, Clostridium perfringens* and *Escherichia coli* | Human | - | 2-Amino-3,6-dihydro-3-methyl-7H-imidazo[4,5-f] quinolin-7-one | Increase toxicity | Oxidation (Hydroxylation) | [42] |
| 2-Nitrofluorene | Nitro-polycyclic aromatic hydrocarbons (NPAHs) | - | Human | Nitro reductase | - | Decrease mutagenicity | Reduction (Nitro reduction) | [43] |
|  |  | - | Rat | Nitro reductase | 2-Aminofluorene and hydroxylated nitrofluorene | Decrease mutagenicity (2-aminofluorene) or increase mutagenicity (hydroxylated nitrofluorene) | Reduction (Nitro reduction) | [44] |
| 3-Nitrobenzo(a)pyrene | Nitro-polycyclic aromatic hydrocarbons (NPAHs) | - | Rat | Nitro reductase | 3-Aminobenzo(a)pyrene | - | Reduction (Nitro reduction) | [45] |
| 4-Nitrobenzoic acid | Nitro-polycyclic aromatic hydrocarbons (NPAHs) | *Clostridium perfringens, Clostridium* *leptum, Clostridium paraputrificum, Clostridium* sp. and *Eubacterium* sp. | Human | Nitro reductase | 4-Aminobenzoic acid | - | Reduction (Nitro reduction) | [32, 46] |
| 6-Nitrobenzo(a)pyrene | Nitro-polycyclic aromatic hydrocarbons (NPAHs) | - | Human, rat | Nitro reductase | 6-Aminobenzo(a)pyrene and 6-nitrosobenzo[a]pyrene | - | Reduction (Nitro reduction) | [34, 47] |
| 6-Nitrochrysene | Nitro-polycyclic aromatic hydrocarbons (NPAHs) | - | Human, rat and mouse | Nitro reductase | 6-Aminochrysene, N-formyl-6-aminochrysene and 6-nitrosochrysene | Increase carcinogenicity | Reduction (Nitro reduction) | [48] |
| Glutathione conjugate of 1- Nitropyrene oxide | Nitro-polycyclic aromatic hydrocarbons (NPAHs) | *Peptostreptococcus magnus* | Human and rat | Cys β-lyases | 1- Nitropyrene oxide | Decrease toxicity | Hydrolysis (Deconjugation) | [49, 50] |
| Nitrobenzene | Nitro-polycyclic aromatic hydrocarbons (NPAHs) | - | Rat | Nitro reductase | Nitrosobenzene, phenylhydroxylamine and alinine | - | Reduction (Nitro reduction) | [51] |
| Acid yellow (FD&C Yellow 5) | Azo dyes | *Streptococcus* *faecalis,* | Rat | Azo reductase | - | - | Reduction (Azo reduction) | [52] |
| Allura Red AC (FD&C Red 40) | Azo dyes | *Bifidobacterium infantis, Acidaminococcus fermentans, Coprococcus catus, Eubacterium biforme, Peptostreptococcus productus, Bacteroides thetaitaomcron, Citrobacter* sp. and *Fusobacterium* sp. | - | Azo reductase | - | - | Reduction (Azo reduction) | [53] |
|  |  | *Bacteroides ovatus* and *Enterococcus faecalis* | Rat | Azo reductase | 1-amino-2-naphthol-6-sulfonate sodium salt (ANSA-Na) | - | Reduction (Azo reduction) | [54] |
|  |  | - | Rat | Azo reductase | - | - | Reduction (Azo reduction) | [55] |
| Amaranth (FD&C Red 2) | Azo dyes | *Enterococcus faecalis ATCC 19433* | Human | Azo reductase | - | - | Reduction (Azo reduction) | [56] |
|  |  | *Enterococcus faecalis, Enterococcus faecium, Bacillus cereus* and *Escherichia coli* | Human | Azo reductase | - | - | Reduction (Azo reduction) | [57] |
|  |  | *Bifidobacterium infantis, Acidaminococcus fermentans, Coprococcus catus, Eubacterium biforme, Peptostreptococcus productus, Bacteroides thetaitaomcron Citrobacter* sp. and *Fusobacterium* sp. | - | Azo reductase | - | - | Reduction (Azo reduction) | [53] |
|  |  | *Proteus* | Rat | Azo reductase | - | - | Reduction (Azo reduction) | [58, 59] |
| Azobenzene | Azo dyes | - | Rat | Azo reductase | Aniline | Increase carcinogenicity | Reduction (Azo reduction) | [60] |
| Buffalo Black NBR | Azo dyes | *Clostridium perfringens ATCC 3626* | - | Azo reductase | - | - | Reduction (Azo reduction) | [61] |
| Cibacron Brilliant Red 3B-A | Azo dyes | *Clostridium perfringens ATCC 3626* | - | Azo reductase | - | - | Reduction (Azo reduction) | [61] |
| Congo Red | Azo dyes | *Clostridium perfringens ATCC 3626* | - | Azo reductase | - | - | Reduction (Azo reduction) | [61] |
| Direct Blue 15 | Azo dyes | *Clostridium perfringens ATCC 3626* | - | Azo reductase | - | - | Reduction (Azo reduction) | [61] |
|  |  | *Clostridium perfringens, Clostridium* *leptum, Clostridium paraputrificum, Clostridium* sp. and *Eubacterium* sp. | - | Azo reductase | 3,3'-Dimethoxybenzidine | - | Reduction (Azo reduction) | [46] |
|  |  | *Butyrivibrio* sp.*, Clostridium clostridiiforme, Clostridium paraputrificum, Clostridium nexile, Clostridium* sp.*, Eubacterium* sp.*, Eubacterium hadrum* and *Bacteroides* sp. | Human | Azo reductase | 3,3'-Dimethoxybenzidine | - | Reduction (Azo reduction) | [62] |
| Eriochrome Black T | Azo dyes | *Clostridium perfringens ATCC 3626* | - | Azo reductase | - | - | Reduction (Azo reduction) | [61] |
| Fast yellow | Azo dyes | *Proteus* | Rat | Azo reductase | - | - | Reduction (Azo reduction) | [58, 59] |
| Janus Green | Azo dyes | *Clostridium perfringens ATCC 3626* | - | Azo reductase | - | - | Reduction (Azo reduction) | [61] |
| Methyl orange | Azo dyes | *Bifidobacterium infantis, Acidaminococcus fermentans, Coprococcus catus, Eubacterium biforme, Peptostreptococcus productus, Bacteroides thetaitaomcron Citrobacter* sp. and *Fusobacterium* sp. | - | Azo reductase | - | - | Reduction (Azo reduction) | [53] |
|  |  | *Proteus* | Rat | Azo reductase | - | - | Reduction (Azo reduction) | [58, 59] |
| Methyl red | Azo dyes | *Enterococcus faecalis ATCC 19433* | Human | Azo reductase | - | - | Reduction (Azo reduction) | [56] |
|  |  | *Clostridium perfringens ATCC 3626* | - | Azo reductase | - | - | Reduction (Azo reduction) | [61] |
| Naphthalene fast orange 2G | Azo dyes | *Proteus* | Rat | Azo reductase | - | - | Reduction (Azo reduction) | [58, 59] |
| Orange G | Azo dyes | *Enterococcus faecalis ATCC 19433* | Human | Azo reductase | - | - | Reduction (Azo reduction) | [56] |
| Orange II | Azo dyes | *Enterococcus faecalis ATCC 19433* | Human | Azo reductase | - | - | Reduction (Azo reduction) | [56] |
|  |  | *Bifidobacterium infantis, Acidaminococcus fermentans, Coprococcus catus, Eubacterium biforme, Peptostreptococcus productus, Bacteroides thetaitaomcron Citrobacter* sp. and *Fusobacterium* sp. | - | Azo reductase | - | - | Reduction (Azo reduction) | [53] |
| Para Red | Azo dyes | *Bacteroides vulgatus, Bacteroides ovatus, Bacteroides uniformis, Bacteroides distasonis, Bacteroides fragilis, Bacteroides thetaiotaomicron, Bacteroides caccae, Bifidobacterium longum, Bifidobacterium infantis, Bifidobacterium angulatum, Clostridium perfringens, Clostridium butyricum, Clostridium ramosum, Clostridium difficile, Clostridium indolis, Clostridium leptum, Clostridium clostridioforme, Eubacterium aerofaciens, Eubacterium limosum, Enterococcus faecalis, Enterococcus faecium, Fusobacterium russii, Fusobacterium nucleatum, Lacticaseibacillus paracasei, Limosilactobacillus reuteri, Lactobacillus rhamnosus, Ligilactobacillus ruminis, Ruminococcus obeum* and *Ruminococcus gnavus* | Human | Azo reductase | - | - | Reduction (Azo reduction) | [63] |
| Poly S | Azo dyes | *Bifidobacterium adolescentis, Acidaminococcus fermentans, Clostridium paraputrificum, Clostridium ramosum, Clostridium sporogenes, Eubacterium aerofaciens, Lactobacillus catenaforme, Peptococcus prevotii, Ruminococcus bromii, Streptococcus faecium, Veillonella parvula, Klebsiella pneumoniae, Proteus vulgaris, Pseudomonas aeruginosa* and *Salmonella typhimurium* | - | Azo reductase | - | - | Reduction (Azo reduction) | [64] |
| Poly T | Azo dyes | *Bifidobacterium adolescentis, Acidaminococcus fermentans, Clostridium paraputrificum, Clostridium ramosum, Clostridium sporogenes, Eubacterium aerofaciens, Lactobacillus catenaforme, Peptococcus prevotii, Ruminococcus bromii, Streptococcus faecium, Veillonella parvula, Klebsiella pneumoniae, Proteus vulgaris, Pseudomonas aeruginosa* and *Salmonella typhimurium* | - | Azo reductase | - | - | Reduction (Azo reduction) | [64] |
| Poly Y-607 | Azo dyes | *Bifidobacterium adolescentis, Acidaminococcus fermentans, Clostridium paraputrificum, Clostridium ramosum, Clostridium sporogenes, Eubacterium aerofaciens, Lactobacillus catenaforme, Peptococcus prevotii, Ruminococcus bromii, Streptococcus faecium, Veillonella parvula, Klebsiella pneumoniae, Proteus vulgaris, Pseudomonas aeruginosa* and *Salmonella typhimurium* | - | Azo reductase | - | - | Reduction (Azo reduction) | [64] |
| Ponceau BS | Azo dyes | *Enterococcus faecalis ATCC 19433* | Human | Azo reductase | - | - | Reduction (Azo reduction) | [56] |
| Ponceau S | Azo dyes | *Enterococcus faecalis ATCC 19433* | Human | Azo reductase | - | - | Reduction (Azo reduction) | [56] |
| Ponceau SX | Azo dyes | *Bifidobacterium infantis, Acidaminococcus fermentans, Coprococcus catus, Eubacterium biforme, Peptostreptococcus productus, Bacteroides thetaitaomcron Citrobacter* sp. and *Fusobacterium* sp. | - | Azo reductase | - | - | Reduction (Azo reduction) | [53] |
|  |  | *Proteus* | Rat | Azo reductase | - | - | Reduction (Azo reduction) | [58, 59] |
| Sudan I | Azo dyes | *Bacteroides ovatus, Bacteroides distasonis, Bacteroides thetaiotaomicron, Bacteroides caccae, Bifidobacterium infantis, Clostridium indolis, Enterococcus faecalis, Enterococcus faecium, Fusobacterium russii, Lacticaseibacillus paracasei, Lactobacillus rhamnosus* and *Ruminococcus obeum* | Human | Azo reductase | Aniline, 2,4-dimethylaniline, o-toluidine and 4-nitroaniline | Increase carcinogenicity | Reduction (Azo reduction) | [63, 65] |
| Sudan III |  | *Bacteroides ovatus, Bacteroides distasonis, Bacteroides thetaiotaomicron, Bacteroides caccae, Bifidobacterium infantis, Clostridium indolis, Enterococcus faecalis, Enterococcus faecium, Fusobacterium russii, Lacticaseibacillus paracasei, Lactobacillus rhamnosus* and *Ruminococcus obeum* | Human | Azo reductase | Aniline, 2,4-dimethylaniline, o-toluidine and 4-nitroaniline | Increase carcinogenicity | Reduction (Azo reduction) | [63, 65] |
| Sudan IV |  | *Bacteroides ovatus, Bacteroides distasonis, Bacteroides thetaiotaomicron, Bacteroides caccae, Bifidobacterium infantis, Clostridium indolis, Enterococcus faecalis, Enterococcus faecium, Fusobacterium russii, Lacticaseibacillus paracasei, Lactobacillus rhamnosus* and *Ruminococcus obeum* | Human | Azo reductase | Aniline, 2,4-dimethylaniline, o-toluidine and 4-nitroaniline | Increase carcinogenicity | Reduction (Azo reduction) | [63, 65] |
| Sunset yellow (FD&C Yellow 6) | Azo dyes | *Bifidobacterium infantis, Acidaminococcus fermentans, Coprococcus catus, Eubacterium biforme, Peptostreptococcus productus, Bacteroides thetaitaomcron Citrobacter* sp. and *Fusobacterium* sp. | - | Azo reductase | - | - | Reduction (Azo reduction) | [53] |
|  |  | *Bifidobacterium adolescentis, Acidaminococcus fermentans, Clostridium paraputrificum, Clostridium ramosum, Clostridium sporogenes, Eubacterium aerofaciens, Lactobacillus catenaforme, Peptococcus prevotii, Ruminococcus bromii, Streptococcus faecium, Veillonella parvula, Klebsiella pneumoniae, Proteus vulgaris, Pseudomonas aeruginosa* and *Salmonella typhimurium* | - | Azo reductase | - | - | Reduction (Azo reduction) | [64] |
|  |  | *Proteus* | Rat | Azo reductase | - | - | Reduction (Azo reduction) | [58, 59] |
|  |  | *Bacteroides ovatus* and *Enterococcus faecalis* | Rat | Azo reductase | 1-amino-2-naphthol-6-sulfonate sodium salt (ANSA-Na) | - | Reduction (Azo reduction) | [54] |
| Tartrazine (FD&C Yellow 5) | Azo dyes | *Clostridium perfringens ATCC 3626* | - | Azo reductase | - | - | Reduction (Azo reduction) | [61] |
|  |  | *Bifidobacterium infantis, Acidaminococcus fermentans, Coprococcus catus, Eubacterium biforme, Peptostreptococcus productus, Bacteroides thetaitaomcron Citrobacter* sp. and *Fusobacterium* sp. | - | Azo reductase | - | - | Reduction (Azo reduction) | [53] |
|  |  | *Bifidobacterium adolescentis, Acidaminococcus fermentans, Clostridium paraputrificum, Clostridium ramosum, Clostridium sporogenes, Eubacterium aerofaciens, Lactobacillus catenaforme, Peptococcus prevotii, Ruminococcus bromii, Streptococcus faecium, Veillonella parvula, Klebsiella pneumoniae, Proteus vulgaris, Pseudomonas aeruginosa* and *Salmonella typhimurium* | - | Azo reductase | - | - | Reduction (Azo reduction) | [64] |
|  |  | *Proteus* | - | Azo reductase | - | - | Reduction (Azo reduction) | [58, 59] |
| Trypan Blue | Azo dyes | *Clostridium perfringens ATCC 3626* | - | Azo reductase | - | - | Reduction (Azo reduction) | [61] |
| Yellow 2G | Azo dyes | *Proteus* | Rat | Azo reductase | - | - | Reduction (Azo reduction) | [58, 59] |
| 3,4-Dimethoxyacetanilide | Raw chemical | - | Rat | Deacetylase | - | - | Hydrolysis (Deacylation) | [66] |
| 3,4-Dimethoxycinnamic acid | Raw chemical | - | Rat | Deacetylase | - | - | Hydrolysis (Deacylation) | [66] |
| 3,5-Dimethoxyphenylpropionic acid | Raw chemical | - | Rat | Deacetylase | - | - | Hydrolysis (Deacylation) | [66] |
| Diphenyl sulfoxide | Raw chemical | *Escherichia coli* | Rat | Sulfatase | - | - | Hydrolysis | [67] |
| Formanilide | Raw chemical | *-* | Rat | - | Aniline | - | Hydrolysis (Deacylation) | [66] |
| Glycolanilide | Raw chemical | - | Rat | - | Aniline | - | Hydrolysis (Deacylation) | [66] |
| Melamine | Raw chemical | *Klebsiella* | Rat | - | Ammonia and cyanuric acid | Increase toxicity | Reduction | [68] |
|  |  | *Klebsiella terragena* | Human | - | Cyanuric acid | Increase toxicity | Reduction | [69, 70] |

**Table S2.** Summary of transformation of metals by the gut microbiota from human and fauna.

| **Name** | **Classification** | **Functional microbes** | **Hosts** | **Enzymes** | **Metabolites** | **Potential**  **consequences** | **Mechanisms** | **References** |
| --- | --- | --- | --- | --- | --- | --- | --- | --- |
| As | Inorganic As (Arsenate, iAs^V^) | *Desulfovibrio* | Rat | Arsenic methyltransferase and arsenate reductase | iAs^III^, monomethylarsonic acid (MMA^V^) and dimethylarsinic acid (DMA^V^) | Decrease toxicity and increase bioavailability | Reduction and methylation | [71-73] |
|  |  | - | Rat | Arsenic methyltransferase | Monomethylarsonic acid (MMA^V^), dimethylarsinic acid (DMA^V^), monothio-iAs (As^V^S_1_), dithio-iAs (As^V^S_2_), trithio-iAs (As^V^S_3_), monomethylmonothioarsonic acid (MMMTA^V^), monomethyldithioarsonic acid (MMDTA^V^), dimethylmonothioarsonic acid (DMMTA^V^), dimethyldithioarsonic acid (DMDTA^V^), and monomethyltrithioarsonic acid (MMTTA^V^) | Decrease toxicity and increase bioavailability | Methylation, thiolation and methylthiolatio | [74, 75] |
|  |  | *Methanosphaera stadtmanae* and *Methanobrevibacter smithii* | Human | Arsenic methyltransferase | Monomethylarsonic acid (MMA^V^), dimethylarsinic acid (DMA^V^) and monomethylmonothioarsonic acid (MMMTA^V^), dimethylmonothioarsonic acid (DMMTA^V^) and monomethyldithioarsonic acid (MMDTA^V^) | Decrease toxicity and increase bioavailability | Methylation and methylthiolation | [76-78] |
|  |  | - | Human | Arsenate reductase | iAs^III^ | Increase toxicity | Reduction | [79] |
|  | Inorganic As (Arsenate, iAs^III^) | - | Rat | Arsenic methyltransferase | Monomethylarsonic acid (MMA^V^) and dimethylarsinic acid (DMA^V^) | Increase bioavailability | Reduction and methylation | [80] |
|  |  | *Bacteroides*, *Clostridium*, *Alistipes*, and *Bilophila* | Human | Arsenite oxidase and arsenic methyltransferase | Monomethylarsonic acid (MMA^V^) and dimethylarsinic acid (DMA^V^) | Decrease toxicity and increase bioavailability | Methylation and oxidation | [81] |
|  | Monomethylarsonic acid (MMA^V^) | *Desulfovibrio desulfuricans* | Human | - | Monomethylmonothioarsonic acid (MMMTA^V^) and inorganic As (iAs ^IV^) | Increase toxicity | Thiolation and demethylation | [82, 83] |
|  | Dimethylarsinic acid (DMA^V^) | *Escherichia coli* | Rat | - | Unknown arsenic compounds | Increase toxicity and increase bioavailability | - | [84] |
|  | Arsenosugar 329  (3-[5′-deoxy-5-(dimethylarsinoyl)-β-ribofuranosyloxy]-2-hydroxypropanesulfonic acid) | - | Rat | - | Sulfur analog of AsS (329-S) | Decrease bioavailability | - | [85] |
|  | Arsenosugar phospholipids | *Escherichia-Shigella* | Rat | - | Small molecule organic arsenic | Increase bioavailability | - | [86] |
|  | Phosphate arsenosugar | *Parabacteroides* | Rat | - | Small molecule organic arsenic | Increase bioavailability | - | [86] |
|  | Sulfonate arsenosugar | *Parabacteroides* | Rat | - | Small molecule organic arsenic | Increase bioavailability | - | [86] |
|  | Arsenocholine | *Alistipes, Odoribacter, Oscillibacter, Rikenella, Desulfovibrio, Lachnospiraceae, Oscillospiraceae, Gastranaerophilales,* and *Enterococcus* | Rat | - | Small molecule organic arsenic | Increase bioavailability | - | [86] |
| As | Inorganic As (Arsenate, iAs^V^) | *Escherichia coli* | Worm (*Caenorhabditis elegans*) | Arsenic methyltransferase (AS3MT and arsM) and arsenate reductase | iAs^III^ and monomethylarsonic acid (MMA^V^) | Decrease toxicity and increase bioavailability | Reduction and methylation | [87] |
|  |  | - | Earthworm (*Eisenia foetida*) | Arsenate reductase | iAs^III^ | Increase toxicity | Reduction | [88] |
|  |  | *Stenotrophomonas maltophilia* | Fish (*Oreochromis mossambicus* or *Danio rerio*) | Arsenic methyltransferase (AS3MT) and arsenate reductase | iAs^III^, monomethylarsonic acid (MMA^V^) and dimethylarsinic acid (DMA^V^), and arsenobetaine (AsB) | Decrease toxicity and increase bioavailability | Reduction and methylation | [89, 90] |
| Bi | Bismuth subcitrate (Bi^III^) | *-* | Rat | - | Trimethylbismuth (MeBi^III^) | Increase toxicity and increase bioavailability | Methylation | [91] |
|  |  |  | Human | - | Trimethylbismuth (MeBi^III^) | Increase toxicity and increase bioavailability | Methylation | [91] |
|  | iBi^III^ | *Desulfovibrio piger,* Eubacterium eligens, Lactobacillus acidophilus, *Methanosphaera stadtmanae* and *Methanobrevibacter smithii* | Human | - | Trimethylbismuth (MeBi^III^) | Increase toxicity and increase bioavailability | Methylation | [78] |
| Cd | iCd^II^ | *Carnobacterium divergens, Enterococcus faecium, Lactobacillus rhamnosus, Leuconostoc mesenteroides* and *Pediococcus* sp. | Human | - | Complexes formed between heavy metal and bacterial cells | Decrease toxicity | - | [92, 93] |
| Cu | iCu^II^ | *Bacillus licheniformia* | Earthworm (*Metaphire posthuma*) | - | Complexes formed between heavy metal and bacterial cells | Decrease toxicity | - | [94] |
| Hg | iHg^II^ | *Desulfovibrio piger* | Human | - | Methylmercury (MeHg^I^) | Increase toxicity | Methylation | [95-97] |
|  |  | *Desulfotomaculum ruminis* and *Desulfovibrio giganteus* | Sheep | - | Methylmercury (MeHg^I^) | Increase toxicity | Methylation | [95] |
|  |  | *Desulfovibrio* sp. | Cattle | - | Methylmercury (MeHg^I^) | Increase toxicity | Methylation | [95] |
|  |  | *-* | Rat | - | Methylmercury (MeHg^I^) | Increase toxicity | Methylation | [98] |
|  | Methylmercury (CH_3_Hg^I^) | *Escherichia coli* and *Lactobacillus* | Rat | - | Hg(^0^) | Decrease toxicity | Demethylation and reduction | [99-103] |
|  |  | *Bacteroides, Bifidobacteria* and *Escherichia coli.* | Human | - | Hg(^0^) | Decrease toxicity | Demethylation and reduction | [99] |
| Hg | iHg^II^ | *Desulfovibrio* | Earthworm (*Eisenia foetida*) | - | Methylmercury (MeHg^I^) | Increase toxicity | Methylation | [95] |
|  |  | *Desulfovibrio termitidis* | Termite (*Heterotermes indicola*) | - | Methylmercury (MeHg^I^) | Increase toxicity | Methylation | [95] |
|  |  | *-* | Fish | - | Methylmercury (MeHg^I^) | Increase toxicity | Methylation | [104-106] |
|  | Methylmercury (CH_3_Hg^I^) | *Pseudomonas* sp. and *Enterobacter* sp. | Fish | - | iHg^II^ | Decrease toxicity | Demethylation | [105-107] |
|  | *Desulfovibrio desulfuricans, Selenomonas ruminantium,* and *Megasphaera elsdenii* | Cattle | - | Hg(^0^) | Decrease toxicity | Demethylation and reduction | [108] |  |
| Pb | iPb^II^ | *Enterococcus faecium, Enterobacter* sp., *Escherichia coli, Hafnia alvei*, *Klebsiella* sp. and *Serratia marcescens* | Human | - | Complexes formed between heavy metal and bacterial cells | Decrease toxicity | - | [92, 93] |
| Sb | iSb^III^ | *Bacteroides vulgatus, Methanosphaera stadtmanae* and *Methanobrevibacter smithii* | Human | - | Methylantimony (MeSb^III^) | Increase toxicity and increase bioavailability | Methylation | [78] |
| Se | iSe^IV^ | *Bacteroides coprocola, Bacteroides vulgatus, Bifidobacterium bifidum, Clostridium aceticum, Collinsella intestinalis,* *Eubacterium biforme,* Eubacterium eligens, Lactobacillus acidophilus, *Methanosphaera stadtmanae*, *Methanobrevibacter smithii* and *Ruminococcus hansenii* | Human | - | Dimethylselenium (MeSe^II^) and dimethyl thioselenide (MeSe^II^S) | Increase toxicity and increase bioavailability | Methylation and methylthiolation | [78] |
| Te | iTe^II^ | *Bacteroides coprocola, Bacteroides vulgatus, Clostridium aceticum,* *Eubacterium biforme,* Eubacterium eligens, Lactobacillus acidophilus, *Methanosphaera stadtmanae* and *Methanobrevibacter smithii* | Human | - | Methytellurium (MeTe^II^) | Increase toxicity and increase bioavailability | Methylation | [78] |
| Zn | iZn^II^ | *Bacillus licheniformia* | Earthworm (*Metaphire posthuma*) | - | Complexes formed between heavy metal and bacterial cells | Decrease toxicity | - | [94] |

**Table S3.** Summary of transformation of pharmaceutical and personal care products (PPCPs) by the gut microbiota from human and fauna.

| **Name** | **Classification** | **Functional microbes** | **Hosts** | **Enzymes** | **Metabolites** | **Potential**  **consequences** | **Mechanisms** | **References** |
| --- | --- | --- | --- | --- | --- | --- | --- | --- |
| 5-Aminosalicylate (Hepatic metabolite of Sulfasalazine) | Digestive-related drug | *Citrobacter amalonaticus, Citrobacter farmer,*  *Citrobacter freundii* and *Citrobacter koseri*  *Klebsiella oxytoca, Klebsiella rhinoscleromatis*  *Morganella morganii*  *Serratia marcescens*  *Escherichia coli*  *Salmonella enterica*  *Pseudomonas aeruginosa*  *Helicobacter pylori* and  *Bacteroides* sp. | Human | Acetyl transferase | AC-5-aminosalicylate | Decrease efficacy | Functional group removal (Acetylation) | [109] |
| 5-Fluorocytosine | Antifungal agent | *Escherichia coli* | Human | - | 5-Fluorouracil | Increase toxicity | Functional group removal (Transamination) | [110] |
|  |  | - | Human | - | 5-Fluorouracil | Increase toxicity | Functional group removal (Transamination) | [111] |
| 5-Fluorouracil | Antitumor drug | *Escherichia coli* | Human | Dehydrogenase | - | - | Oxidation | [112] |
|  |  | *-* | Rat | - | Cyanuric acid | - | Oxidation | [113] |
| Acetaminophen | Nonsteroidal antiinflammatory drug | - | Rat | N-deacetylase | Aminophenol | Increase efficacy and toxicity | Hydrolysis  (Deacylation) | [66] |
| Acetaminophen-Glth, acetaminophen-Sul and acetaminophen-Glu (Hepatic metabolite of acetaminophen) | Nonsteroidal antiinflammatory drug | - | Rat | β-Glucuronidase | Acetaminophen | Increase efficacy and enterohepatic circulation | Hydrolysis  (Deconjugation and other deconjugation reactions) | [114-116] |
| Acetanilide | Analgesic | - | Rat | N-deacetylase | Aniline | - | Hydrolysis  (Deacylation) | [66] |
| Anagrelide | Endocrine drug | Bacteroides dorei | Human | - | - | - | - | [117] |
| Artemisinin | Natural products (Natural drug) | Akkermansia muciniphila, Alistipes indistinctus, Anaerotruncus colihominis, Anaerococcus hydrogenalis, Anaerostipes sp., Bacteroides caccae, Bacteroides cellulosilyticus, Bacteroides coprophilus, Bacteroides dorei, Bacteroides eggerthii, Bacteroides fragilis, Bacteroides finegoldii, Bacteroides intestinalis, Bacteroides ovatus, Bacteroides pectinophilus, Bacteroides stercoris, Bacteroides uniformis, Bacteroides vulgatus, Bacteroides xylanisolvens, Blautia hansenii, Blautia luti, Bifidobacterium adolescentis, Bifidobacterium breve, Bifidobacterium longum, Bifidobacterium ruminatum, Bacteroides thetaiotaomicron, Bryantia formatexigens, Clostridium asparagiforme, Clostridium bolteae, Clostridium difficile, Clostridium hathewayi, Clostridium scindens, Clostridium sp., Clostridium spiroforme, Clostridium sporogenes, Clostridium ramosum, Clostridium symbiosum, Collinsella aerofaciens, Collinsella intestinalis, Coprococcus comes, Dorea formicigenerans, Edwardsiella tarda, Eggerthella lenta, Enterobacter cancerogenus, Enterococcus faecalism, Escherichia coli, Eubacterium biforme, Eubacterium hallii, Eubacterium rectale, Eubacterium ventriosum, Limosilactobacillus reuteri, Odoribacter splanchnius, Parabacteroides distasonis, Parabacteroides johnsonii, Parabacteroides merdae, Pretovella copri, Proteus penneri, Providencia alcalifaciens, Providencia rettgeri, Providencia stuartii, Roseburia intestinalis, Ruminococcus gnavus, Ruminococcus lactaris, Ruminococcus torques, Salmonella Typhimurium, Subdoligranulum variabile and Victivallis vadensis | Human | - | - | - | - | [117] |
| Azatadine maleate | Antihistamine agent | Bacteroides eggerthii and Bacteroides vulgatus | Human | - | - | - | - | [117] |
| Azetirelin | Endocrine drug | - | Rat, dog, and human | - | - | Decrease efficacy | Hydrolysis  (Proteolysis) | [118] |
|  |  | - | Rat | β-Glucuronidase | Baicalein and oroxylin A | Increase efficacy and decrease toxicity | Hydrolysis  (Deconjugation) | [119-121] |
| Balsalazide | Digestive-related drug | - | Human | Azo reductase | 5-Aminosalicylic acid | Decrease mutagenicity | Reduction  (Azo reduction) | [122] |
|  |  | - | Rat | Azo reductase | 5-Aminosalicylic acid | Decrease mutagenicity | Reduction  (Azo reduction) | [122, 123] |
| Benazepril | Endocrine drug | Bacteroides coprophilus, Bacteroides dorei, Bacteroides fragilis, Bacteroides ovatus, Bacteroides stercoris, Bacteroides thetaiotaomicron, Bacteroides vulgatus, Blautia hansenii, Parabacteroides distasonis and Pretovella copri | Human | - | - | - | - | [117] |
| Benzbromarone | Endocrine drug | Bifidobacterium breve | Human | - | - | - | - | [117] |
| Benzthiazide | Endocrine drug | Bacteroides fragilis, Blautia hansenii and Clostridium sp. | Human | - | - | - | - | [117] |
| Benzylpenicillin | Antibiotic | *Enterobacter cloacae* and *Enterobacter aerogenes* | Human | β-Lactamase | - | Decrease efficacy | Hydrolysis  (Deacylation) | [124] |
| Berberine | Plant polyphenols (Isoquinolines) | - | Rat | Nitro reductase | Dihydroberberine | Increase efficacy and increase bioavailability | Reduction (Nitro reduction) | [125] |
| Betamethasone acetate | Endocrine drug | Bacteroides dorei, Bacteroides fragilis, Blautia hansenii, Parabacteroides johnsonii, Bacteroides stercoris and Odoribacter splanchnius | Human | - | - | - | - | [117] |
| Betamethasone valerate | Endocrine drug | Bacteroides dorei, Bacteroides eggerthii, Bacteroides fragilis, Bacteroides thetaiotaomicron, Bacteroides uniformis, Bacteroides vulgatus, Blautia hansenii, Clostridium sp., Eggerthella lenta, Escherichia coli, Parabacteroides johnsonii, Odoribacter splanchnius and Victivallis vadensis | Human | - | - | - | - | [117] |
| Bezafibrate | Endocrine drug | Bacteroides fragilis, Bacteroides vulgatus and Odoribacter splanchnius | Human | - | - | - | - | [117] |
| Bicalutamide | Endocrine drug | Bacteroides fragilis | Human | - | - | - | - | [117] |
| BILR-355 | Reverse transcriptases | - | Human | - | BILR 402 | - | Reduction | [126] |
| Biperiden | Nervous system drug | Bacteroides dorei, Bacteroides vulgatus and Blautia hansenii | Human | - | - | - | - | [117] |
| Bisacodyl | Digestive-related drug | Akkermansia muciniphila, Alistipes indistinctus, Anaerotruncus colihominis, Anaerococcus hydrogenalis, Anaerostipes sp., Bacteroides caccae, Bacteroides cellulosilyticus, Bacteroides coprophilus, Bacteroides dorei, Bacteroides eggerthii, Bacteroides fragilis, Bacteroides finegoldii, Bacteroides intestinalis, Bacteroides ovatus, Bacteroides pectinophilus, Bacteroides stercoris, Bacteroides uniformis, Bacteroides vulgatus, Bacteroides xylanisolvens, Blautia hansenii, Blautia luti, Bifidobacterium adolescentis, Bifidobacterium breve, Bifidobacterium longum, Bifidobacterium ruminatum, Bacteroides thetaiotaomicron, Bryantia formatexigens, Clostridium asparagiforme, Clostridium bolteae, Clostridium difficile, Clostridium hathewayi, Clostridium scindens, Clostridium sp., Clostridium spiroforme, Clostridium sporogenes, Clostridium ramosum, Clostridium symbiosum, Collinsella aerofaciens, Collinsella intestinalis, Coprococcus comes, Dorea formicigenerans, Edwardsiella tarda, Eggerthella lenta, Enterobacter cancerogenus, Enterococcus faecalism, Escherichia coli, Eubacterium biforme, Eubacterium hallii, Eubacterium rectale, Eubacterium ventriosum, Limosilactobacillus reuteri, Odoribacter splanchnius, Parabacteroides distasonis, Parabacteroides johnsonii, Parabacteroides merdae, Pretovella copri, Proteus penneri, Providencia alcalifaciens, Providencia rettgeri, Providencia stuartii, Roseburia intestinalis, Ruminococcus gnavus, Ruminococcus lactaris, Ruminococcus torques, Salmonella Typhimurium, Subdoligranulum variabile and Victivallis vadensis | Human | - | - | - | - | [117] |
| Brivudine | Antiviral drug | Bacteroides thetaiotaomicron and Bacteroides ovatus | Human and rat | Nitro reductase | Bromovinyluracil (BVU) | - | Reduction  (Nitro reduction) | [127] |
| Bromocriptine mesylate | Endocrine drug | Akkermansia muciniphila, Alistipes indistinctus, Anaerotruncus colihominis, Anaerococcus hydrogenalis, Anaerostipes sp., Bacteroides caccae, Bacteroides cellulosilyticus, Bacteroides coprophilus, Bacteroides dorei, Bacteroides eggerthii, Bacteroides fragilis, Bacteroides finegoldii, Bacteroides intestinalis, Bacteroides ovatus, Bacteroides pectinophilus, Bacteroides uniformis, Bacteroides vulgatus, Bacteroides xylanisolvens, Blautia hansenii, Blautia luti, Bifidobacterium adolescentis, Bifidobacterium breve, Bifidobacterium longum, Bifidobacterium ruminatum, Bacteroides thetaiotaomicron, Clostridium asparagiforme, Clostridium bolteae, Clostridium difficile, Clostridium hathewayi, Clostridium scindens, Clostridium sp., Clostridium spiroforme, Clostridium ramosum, Clostridium symbiosum, Collinsella aerofaciens, Collinsella intestinalis, Coprococcus comes, Dorea formicigenerans, Edwardsiella tarda, Eggerthella lenta, Enterobacter cancerogenus, Enterococcus faecalism, Eubacterium biforme, Eubacterium hallii, Eubacterium rectale, Eubacterium ventriosum, Limosilactobacillus reuteri, Odoribacter splanchnius, Parabacteroides distasonis, Parabacteroides johnsonii, Parabacteroides merdae, Pretovella copri, Proteus penneri, Providencia rettgeri, Providencia stuartii, Roseburia intestinalis, Ruminococcus gnavus, Ruminococcus lactaris, Ruminococcus torques, Salmonella Typhimurium, Subdoligranulum variabile and Victivallis vadensis | Human | - | - | - | - | [117] |
| Bucetin | Nonsteroidal antiinflammatory drug | - | Rat | N-deacetylase | p-Phenetidine | - | Hydrolysis  (Deacylation) | [66] |
| Budesonide | Endocrine drug | Bacteroides dorei, Bacteroides fragilis, Bacteroides vulgatus, Blautia hansenii, Clostridium sp. and Eubacterium biforme | Human | - | - | - | - | [117] |
| Bupropion | Nervous system drug | Bifidobacterium ruminatum and Escherichia coli | Human | - | - | - | - | [117] |
| Calcitonin | Endocrine drug | - | Human | Protease | - | Decrease efficacy | Hydrolysis  (Proteolysis) | [128] |
|  |  | - | Rat | Protease | - | Decrease efficacy | Hydrolysis  (Proteolysis) | [129] |
| Capecitabine | Antitumor drug | Clostridium sp. | Human | - | - | - | - | [117] |
|  |  | Escherichia coli and Parabacteroides distasonis | Human | β-Glucuronidase | Deglycocapecitabine | - | Hydrolysis  (Deconjugation) | [130] |
| Carbenoxolone | Digestive-related drug | - | Rat | β-Glucuronidase | Succinate and  18β-Glycyrrhetic acid | Enterohepatic circulation | Hydrolysis  (Deconjugation) | [131] |
| Carbetapentane citrate | Nervous system drug | Bacteroides uniformis, Clostridium sp. and Victivallis vadensis | Human | - | - | - | Hydrolysis  (Dealkylation) | [117] |
| Carbinoxamine maleate | Antihistamine agent | Bacteroides uniformis | Human | - | - | - | - | [117] |
| Carisoprodol | Nervous system drug | Bacteroides cellulosilyticus, Bacteroides dorei, Bacteroides fragilis, Bacteroides stercoris, Bacteroides thetaiotaomicron, Bacteroides thetaiotaomicron, Bacteroides uniformis, Bacteroides vulgatus, Bacteroides xylanisolven, Blautia hansenii, Clostridium difficil and Clostridium sp. | Human | - | - | - | - | [117] |
| Carvedilol | Endocrine drug | Bacteroides coprophilus, Bacteroides dorei, Bacteroides fragilis, Bacteroides thetaiotaomicron, Bacteroides thetaiotaomicron, Bacteroides uniformis, Bacteroides vulgatus, Bacteroides xylanisolvens, Blautia hansenii, Clostridium sp., Clostridium sporogenes, Eubacterium biforme, Eubacterium rectale, Roseburia intestinalis and Subdoligranulum variabile | Human | - | - | - | - | [117] |
| Celecoxib | Nonsteroidal antiinflammatory drug | Clostridium sporogenes and Eubacterium biforme | Human | - | - | - | - | [117] |
| Cetirizine | Antihistamine agent | Bacteroides cellulosilyticus, Bacteroides dorei, Bacteroides eggerthii, Bacteroides fragilis, Bacteroides pectinophilus, Bacteroides stercoris, Bacteroides thetaiotaomicron, Bacteroides uniformis, Bacteroides vulgatus, Bacteroides xylanisolvens, Blautia hansenii, Eubacterium biforme, Parabacteroides distasonis and Pretovella copri | Human | - | - | - | - | [117] |
| Chloramphenicol | Antibiotic | *Escherichia coli* | Human | Amino hydrolase | - | Decrease efficacy | Hydrolysis  (Deacylation) | [132] |
| Chlormezanone | Nervous system drug | Blautia hansenii, Bifidobacterium ruminatum and Clostridium sp. | Human | - | - | - | - | [117] |
| Cimetidine | Digestive-related drug | Bacteroides dorei, Bacteroides fragilis and Bacteroides thetaiotaomicron | Human | - | - | - | - | [117] |
| Clemastine fumarate | Antihistamine agent | Bacteroides cellulosilyticus, Bacteroides coprophilus, Bacteroides dorei, Bacteroides eggerthii, Bacteroides fragilis, Bacteroides stercoris, Bacteroides uniformis, Bacteroides vulgatus, Bacteroides xylanisolvens, Blautia hansenii, Clostridium sp., Escherichia coli, Eubacterium biforme, Odoribacter splanchnius, Parabacteroides distasonis, Pretovella copri and Roseburia intestinalis | Human | - | - | - | - | [117] |
| Clemizole | Antihistamine agent | Blautia hansenii | Human | - | - | - | - | [117] |
| Clonazepam | Nervous system drug | *Escherichia* *coli* | Rat | Nitro reductase | - | - | Reduction  (Nitro reduction) | [133] |
|  |  | - | Rat | Nitro reductase | 7-Aminonitrazepam | - | Reduction  (Nitro reduction) | [134] |
|  |  | - | Human | Nitro reductase | 7-Aminonitrazepam | - | Reduction  (Nitro reduction) | [130] |
| Clonidine | Endocrine drug | Bacteroides dorei, Bacteroides fragilis, Bacteroides fragilis, Bacteroides ovatus, Bacteroides stercoris, Bacteroides thetaiotaomicron and Bacteroides uniformis | Human | - | - | - | - | [117] |
| Clopidogrel sulfate | Endocrine drug | Bacteroides dorei, Bacteroides eggerthii, Bacteroides fragilis, Bacteroides fragilis, Bacteroides stercoris, Bacteroides thetaiotaomicron, Bacteroides uniformis, Bacteroides vulgatus, Bacteroides xylanisolvens, Blautia hansenii, Clostridium sp., Odoribacter splanchnius and Parabacteroides johnsonii | Human | Sulfatase | - | - | - | [117] |
| Colchicine | Nervous system drug | Bacteroides dorei, Bacteroides fragilis, Bacteroides uniformis, Bacteroides vulgatus, Blautia hansenii and Clostridium sp. | Human | - | - | - | - | [117] |
| Cyclobenzaprine | Nervous system drug | Bacteroides fragilis, Blautia hanseni and Clostridium sp. | Human | - | - | - | - | [117] |
| Cyclophosphamide | Antitumor drug | Blautia hansenii | Human | - | - | - | - | [117] |
| Cyproterone acetate | Endocrine drug | Bacteroides dorei, Bacteroides eggerthii, Bacteroides fragilis, Bacteroides thetaiotaomicron, Bacteroides uniformis, Bacteroides vulgatus, Blautia hansenii, Clostridium sp. and Parabacteroides distasonis | Human | - | - | - | - | [117] |
| Dabigatran etexilate mesylate | Anticoagulant drug | Bacteroides dorei and Bacteroides fragilis | Human | - | - | - | - | [117] |
| Danazol | Endocrine drug | Akkermansia muciniphila, Alistipes indistinctus, Anaerotruncus colihominis, Anaerococcus hydrogenalis, Anaerostipes sp., Bacteroides caccae, Bacteroides cellulosilyticus, Bacteroides dorei, Bacteroides eggerthii, Bacteroides fragilis, Bacteroides finegoldii, Bacteroides intestinalis, Bacteroides pectinophilus, Bacteroides stercoris, Bacteroides uniformis, Bacteroides vulgatus, Bacteroides xylanisolvens, Blautia hansenii, Blautia luti, Bifidobacterium adolescentis, Bifidobacterium breve, Bifidobacterium longum, Bifidobacterium ruminatum, Bacteroides thetaiotaomicron, Bryantia formatexigens, Clostridium asparagiforme, Clostridium bolteae, Clostridium difficile, Clostridium hathewayi, Clostridium scindens, Clostridium sp., Clostridium spiroforme, Clostridium sporogenes, Clostridium ramosum, Clostridium symbiosum, Collinsella aerofaciens, Collinsella intestinalis, Coprococcus comes, Dorea formicigenerans, Edwardsiella tarda, Eggerthella lenta, Enterobacter cancerogenus, Enterococcus faecalism, Escherichia coli, Eubacterium biforme, Eubacterium hallii, Eubacterium rectale, Eubacterium ventriosum, Odoribacter splanchnius, Parabacteroides distasonis, Parabacteroides johnsonii, Parabacteroides merdae, Pretovella copri, Proteus penneri, Providencia alcalifaciens, Providencia rettgeri, Providencia stuartii, Roseburia intestinalis, Ruminococcus gnavus, Ruminococcus lactaris, Ruminococcus torques, Salmonella Typhimurium, Subdoligranulum variabile and Victivallis vadensis | Human | - | - | - | - | [117] |
| Dasatinib | Antitumor drug | Bacteroides fragilis, Blautia hansenii, Clostridium bolteae, Clostridium sp. and Limosilactobacillus reuteri | Human | - | - | - | - | [117] |
| Deflazacort | Digestive-related drug | Alistipes indistinctus, Bacteroides caccae, Bacteroides cellulosilyticus, Bacteroides dorei, Bacteroides eggerthii, Bacteroides fragilis, Bacteroides intestinali, Bacteroides ovatus, Bacteroides thetaiotaomicron, Bacteroides uniformis, Bacteroides vulgatus, Bacteroides xylanisolvens, Blautia hansenii, Clostridium sp., Odoribacter splanchnius, Parabacteroides distasonis, Parabacteroides johnsonii, Parabacteroides merdae and Victivallis vadensis | Human | - | - | - | - | [117] |
| Deleobuvir | Antiviral drug | - | Rat | Dehydrogenase | CD 6168 | Decrease efficacy | Reduction  (Alkene reduction) | [135] |
| Dexamethasone | Endocrine drug | Bacteroides dorei, Bacteroides fragilis and Clostridium scindens | Human | - | - | - | - | [117] |
| Diacetamate | Nonsteroidal antiinflammatory drug | Akkermansia muciniphila, Alistipes indistinctus, Anaerotruncus colihominis, Anaerococcus hydrogenalis, Anaerostipes sp., Bacteroides caccae, Bacteroides cellulosilyticus, Bacteroides coprophilus, Bacteroides dorei, Bacteroides eggerthii, Bacteroides fragilis, Bacteroides finegoldii, Bacteroides intestinalis, Bacteroides ovatus, Bacteroides pectinophilus, Bacteroides stercoris, Bacteroides uniformis, Bacteroides vulgatus, Bacteroides xylanisolvens, Blautia luti, Bifidobacterium adolescentis, Bifidobacterium breve, Bifidobacterium longum, Bifidobacterium ruminatum, Bacteroides thetaiotaomicron, Bryantia formatexigens, Clostridium asparagiforme, Clostridium bolteae, Clostridium difficile, Clostridium hathewayi, Clostridium scindens, Clostridium spiroforme, Clostridium sporogenes, Clostridium ramosum, Clostridium symbiosum, Collinsella aerofaciens, Collinsella intestinalis, Coprococcus comes, Dorea formicigenerans, Edwardsiella tarda, Eggerthella lenta, Enterococcus faecalism, Eubacterium biforme, Eubacterium hallii, Eubacterium rectale, Eubacterium ventriosum, Limosilactobacillus reuteri, Odoribacter splanchnius, Parabacteroides distasonis, Parabacteroides johnsonii, Parabacteroides merdae, Pretovella copri, Proteus penneri, Providencia alcalifaciens, Providencia rettgeri, Providencia stuartii, Roseburia intestinalis, Ruminococcus gnavus, Ruminococcus lactaris, Ruminococcus torques, Salmonella Typhimurium, Subdoligranulum variabile and Victivallis vadensis | Human | - | - | - | Hydrolysis  (Deacylation) | [117] |
| Diclofenac-Glu (Hepatic metabolite of diclofenac) | Nonsteroidal antiinflammatory drug | *Escherichia* *coli* | Rat | β-Glucuronidase | Diclofenac | Decrease toxicity | Hydrolysis  (Deconjugation) | [136, 137] |
| Dicyclomine | Digestive-related drug | Bacteroides dorei, Bacteroides vulgatus, Bacteroides xylanisolvens, Eggerthella lenta, Limosilactobacillus reuteri, Odoribacter splanchnius and Ruminococcus torques | Human | - | - | - | - | [117] |
| Diflorasone diacetate | Endocrine drug | Alistipes indistinctus, Bacteroides cellulosilyticus, Bacteroides dorei, Bacteroides ovatus, Bacteroides thetaiotaomicron, Bacteroides thetaiotaomicron, Bacteroides thetaiotaomicron, Bacteroides uniformis, Bacteroides vulgatus, Bacteroides xylanisolvens, Blautia hansenii, Clostridium asparagiforme, Clostridium sp., Odoribacter splanchnius, Parabacteroides distasonis, Parabacteroides johnsonii, Parabacteroides merdae, Pretovella copri and Victivallis vadensis | Human | - | - | - | - | [117] |
| Digitoxin | Endocrine drug | Bacteroides dorei, Bacteroides fragilis, Bacteroides stercoris, Bacteroides thetaiotaomicron, Bacteroides uniformis, Bacteroides vulgatus, Bacteroides xylanisolvens, Blautia hansenii, Clostridium sp., Odoribacter splanchnius, and Parabacteroides johnsonii  Parabacteroides merdae | Human | - | - | - | - | [117] |
| Digoxin | Endocrine drug | *Bacteroides dorei, Bacteroides fragilis, Bacteroides thetaiotaomicron, Bacteroides uniformis, Bacteroides vulgatus, Bacteroides xylanisolvens, Blautia hansenii, Clostridium* sp.*, Odoribacter splanchnius* and *Parabacteroides johnsonii* | Human | Dehydrogenase | - | - | Reduction  (Alkene reduction) | [117] |
|  |  | *Eggerthella lenta* | Human | Ene reductase | Dihydrodigoxin | Decrease efficacy | Reduction  (Alkene reduction) | [138, 139] |
|  |  | *Eubacterium lentum* | Human | Ene reductase | Dihydrodigoxin | Decrease efficacy | Reduction  (Alkene reduction) | [138] |
|  |  | - | Human | Ene reductase | Dihydrodigoxin and dihydrodigoxicenin | Decrease efficacy | Reduction (Alkene reduction) and hydrolysis | [130, 140, 141] |
| Dihydrosinapic acid | Antimalarial drug | - | Rat | N-Deacetylase | - | - | Hydrolysis  (Decarboxylation) | [66] |
| Diltiazem | Endocrine drug | *Bifidobacterium thetaiotaomicron* | Human | - | Desacetyldiltiazem | Increase efficacy | Hydrolysis  (Deacylation) | [117] |
|  |  | *Bacteroides dorei, Bacteroides fragilis, Bacteroides ovatus, Bacteroides thetaiotaomicron, Bacteroides uniformis, Bacteroides xylanisolvens, Blautia hansenii, Clostridium* sp.*, Parabacteroides distasonis, Parabacteroides johnsonii* and *Victivallis vadensis* | Human | - | Desacetyldiltiazem | Increase efficacy | Hydrolysis  (Deacylation) | [117] |
| Dipyridamole | Endocrine drug | Bacteroides dorei, Blautia hansenii and Clostridium sp. | Human | - | - | - | - | [117] |
| Doxazosin mesylate | Endocrine drug | Bacteroides dorei, Blautia hansenii and Clostridium sp. | Human | - | - | - | - | [117] |
| Drospirenone | Endocrine drug | Bacteroides xylanisolvens, Odoribacter splanchnius, Parabacteroides johnsonii, Bacteroides uniformis, Bacteroides fragilis, Bacteroides dorei, Bacteroides vulgatus and Eubacterium biforme | Human | - | - | - | - | [117] |
| Duloxetine | Nervous system drug | Bacteroides dorei, Clostridium sp., Escherichia coli, Blautia hansenii and Bacteroides coprophilus | Human | - | - | - | - | [117] |
| Eltrombopag | Endocrine drug | - | Human and rat | Hydrazone reductases | SB 61855 | - | Reduction  (Hydrazine cleavage) | [142] |
| Enalapril maleate | Endocrine drug | Blautia hansenii | Human | - | - | - | - | [117] |
| Entacapone | Nervous system drug | Akkermansia muciniphila, Alistipes indistinctus, Anaerotruncus colihominis, Anaerococcus hydrogenalis, Anaerostipes sp., Bacteroides caccae, Bacteroides cellulosilyticus, Bacteroides coprophilus, Bacteroides dorei, Bacteroides eggerthii, Bacteroides fragilis, Bacteroides finegoldii, Bacteroides intestinalis, Bacteroides ovatus, Bacteroides pectinophilus, Bacteroides stercoris, Bacteroides uniformis, Bacteroides vulgatus, Bacteroides xylanisolvens, Blautia hansenii, Blautia luti, Bifidobacterium adolescentis, Bifidobacterium breve, Bifidobacterium longum, Bifidobacterium ruminatum, Bacteroides thetaiotaomicron, Bryantia formatexigens, Clostridium asparagiforme, Clostridium bolteae, Clostridium difficile, Clostridium hathewayi, Clostridium scindens, Clostridium sp., Clostridium spiroforme, Clostridium sporogenes, Clostridium ramosum, Clostridium symbiosum, Collinsella aerofaciens, Collinsella intestinalis, Coprococcus comes, Dorea formicigenerans, Edwardsiella tarda, Enterobacter cancerogenus, Enterococcus faecalism, Eubacterium biforme, Eubacterium hallii, Eubacterium rectale, Eubacterium ventriosum, Odoribacter splanchnius, Parabacteroides distasonis, Parabacteroides johnsonii, Parabacteroides merdae, Pretovella copri, Proteus penneri, Providencia stuartii, Roseburia intestinalis, Ruminococcus gnavus, Ruminococcus lactaris, Ruminococcus torques, Salmonella Typhimurium, Subdoligranulum variabile and Victivallis vadensis | Human | - | - | - | - | [117] |
| Ergonovine maleate | Nervous system drug | Bacteroides dorei, Bacteroides cellulosilyticus, Limosilactobacillus reuteri, Bacteroides fragilis and Bacteroides coprophilus | Human | - | - | - | - | [117] |
| Ergotamine tartrate | Nervous system drug | Bacteroides coprophilus, Bacteroides dorei, Bacteroides fragilis, Bacteroides thetaiotaomicron and Bacteroides uniformis | Human | - | - | - | - | [117] |
| Eszopiclone | Nervous system drug | Bacteroides coprophilus, Bryantia formatexigens, Clostridium sp. and Eggerthella lenta | Human | - | - | - | - | [117] |
| Ethopropazine | Nervous system drug | Bacteroides dorei, Bacteroides vulgatus, Blautia hansenii and Clostridium sp. | Human | - | - | - | - | [117] |
| Ethoxzolamide | Antibiotic | Eubacterium rectale | Human | - | - | - | - | [117] |
| Ethynodiol diacetate | Endocrine drug | Ruminococcus torques | Human | - | - | - | - | [117] |
| Etodolac | Nonsteroidal antiinflammatory drug | Bacteroides fragilis, Bacteroides thetaiotaomicron, Bacteroides uniformis, Bacteroides xylanisolvens, Blautia hansenii and Odoribacter splanchnius | Human | - | - | - | - | [117] |
| Ezetimibe | Endocrine drug | Bacteroides fragilis, Bacteroides thetaiotaomicron, Bacteroides uniformis, Bacteroides xylanisolvens, Clostridium sp. and Odoribacter splanchnius | Human | - | - | - | - | [117] |
| Famciclovir | Endocrine drug | Bacteroides caccae, Bacteroides cellulosilyticus, Bacteroides dorei, Bacteroides eggerthii, Bacteroides fragilis, Bacteroides stercoris, Bacteroides thetaiotaomicron, Bacteroides uniformis, Bacteroides vulgatus, Bacteroides xylanisolvens, Clostridium sp., Odoribacter splanchnius, Parabacteroides johnsonii, Parabacteroides merdae and Victivallis vadensisq | Human | - | - | - | Hydrolysis  (Deacylation) | [117] |
| Famprofazone | Antiviral drug | Bacteroides coprophilus, Bacteroides dorei, Bacteroides fragilis, Bacteroides stercoris, Bacteroides thetaiotaomicron, Bacteroides uniformi, Bacteroides vulgatus, Bacteroides xylanisolvens, Blautia hansenii, Odoribacter splanchnius and Parabacteroides distasonis | Human | - | - | - | - | [117] |
| Febuxostat | Nonsteroidal antiinflammatory drug | Bacteroides dorei, Bacteroides fragilis, Bacteroides thetaiotaomicron, Bacteroides uniformis, Bacteroides vulgatus, Bacteroides xylanisolvens, Blautia hansenii, Clostridium sp. and Odoribacter splanchnius | Human | - | - | - | - | [117] |
| Fenofibrate | Endocrine drug | Bacteroides eggerthii, Bacteroides fragilis, Bacteroides xylanisolvens, Bifidobacterium breve, Bifidobacterium longum, Clostridium asparagiforme, Clostridium difficile, Coprococcus comes, Pretovella copri and Ruminococcus torques | Human | - | - | - | - | [117] |
| Fenspiride | Endocrine drug | Bacteroides coprophilus, Bacteroides fragilis, Bacteroides ovatus, Bacteroides thetaiotaomicron and Bacteroides uniformis | Human | - | - | - | - | [117] |
| Finasteride | Nonsteroidal antiinflammatory drug | Akkermansia muciniphila, Bacteroides dorei, Bacteroides fragilis, Bacteroides uniformis, Bacteroides vulgatus, Bacteroides xylanisolvens and Odoribacter splanchnius | Human | - | - | - | - | [117] |
| Fluconazole | Antifungal agent | Bacteroides dorei, Bacteroides fragilis, Bacteroides stercoris, Bacteroides thetaiotaomicron, Bacteroides uniformis, Bacteroides xylanisolvens, Blautia hansenii, Clostridium sp. and Odoribacter splanchnius | Human | - | - | - | - | [117] |
| Fluoxetine | Endocrine drug | Akkermansia muciniphila, Alistipes indistinctus, Bacteroides caccae, Bacteroides dorei, Bacteroides eggerthii, Bacteroides fragilis, Bacteroides intestinalis, Bacteroides ovatus, Bacteroides pectinophilus, Bacteroides stercoris, Bacteroides uniformis, Bacteroides vulgatus, Bacteroides xylanisolvens, Blautia hansenii, Bifidobacterium adolescentis, Bifidobacterium breve, Bifidobacterium longum, Bifidobacterium ruminatum, Bacteroides thetaiotaomicron, Clostridium asparagiforme, Clostridium bolteae, Clostridium sp., Clostridium spiroforme, Clostridium sporogenes, Clostridium ramosum, Collinsella aerofaciens, Coprococcus comes, Dorea formicigenerans, Edwardsiella tarda, Enterobacter cancerogenus, Escherichia coli, Eubacterium hallii, Eubacterium rectale, Eubacterium ventriosum, Limosilactobacillus reuteri, Odoribacter splanchnius, Parabacteroides distasonis, Parabacteroides johnsonii, Parabacteroides merdae, Pretovella copri, Proteus penneri, Providencia stuartii, Roseburia intestinalis, Ruminococcus lactaris, Salmonella Typhimurium and Subdoligranulum variabile | Human | - | - | - | - | [117] |
| Fluphenazine | Nervous system drug | Anaerostipes sp., Bacteroides dorei, Bacteroides pectinophilus, Bacteroides uniformis, Bacteroides vulgatus, Blautia hansenii, Clostridium asparagiforme, Clostridium hathewayi, Escherichia coli, Eubacterium ventriosum, Odoribacter splanchnius, Parabacteroides distasonis, Pretovella copri and Roseburia intestinalis | Human | - | - | - | - | [117] |
| Fluvoxamine maleate | Nervous system drug | Blautia hansenii and Clostridium sp. | Human | - | - | - | - | [117] |
| Galantamine | Nervous system drug | Bacteroides coprophilus, Bacteroides fragilis, Bacteroides ovatus, Bacteroides thetaiotaomicron and Bacteroides uniformis | Human | - | - | - | - | [117] |
| Gliclazide | Endocrine drug | Bacteroides dorei, Bacteroides fragilis, Bacteroides thetaiotaomicron and Bacteroides vulgatus | Human | - | - | - | - | [117] |
|  |  | Bacteroides dorei, Bacteroides fragilis, Bacteroides thetaiotaomicron, Bacteroides vulgatus, Eubacterium biforme and Odoribacter splanchnius | Human | - | - | - | - | [117] |
| Glucagon | Endocrine drug | - | Human | Protease | - | Decrease efficacy | Hydrolysis  (Proteolysis) | [128] |
|  |  | - | Rat | β-Glucuronidase | 18-β-Glycyrrhetic acid and 3-epi-18β-Glycyrrhetic acid | Increase efficacy | Hydrolysis  (Deconjugation) | [143] |
| Griseofulvin | Antifungal agent | Bacteroides dorei, Bacteroides fragilis, Bacteroides uniformis, Bacteroides vulgatus, Bacteroides xylanisolvens and Odoribacter splanchnius | Human | - | - | - | - | [117] |
| Hydrocortisone | Endocrine drug | *Bifidobacterium adolescentis* and *Clostridium scindens,* | Human | Reductase | 20β-Dihydrocortisone | - | Reduction  (Keto-reduction) | [130] |
| Idebenone | Nervous system drug | Alistipes indistinctus, Bacteroides fragilis, Bacteroides thetaiotaomicron, Bacteroides uniformis, Bacteroides vulgatus, Bacteroides xylanisolvens, Blautia luti, Clostridium bolteae, Clostridium sp., Odoribacter splanchnius and Ruminococcus lactaris | Human | - | - | - | - | [117] |
| Imatinib | Antitumor drug | Bacteroides vulgatus | Human | - | - | - | - | [117] |
| Indapamide | Endocrine drug | Eubacterium biforme | Human | - | - | - | - | [117] |
| Indomethacin | Nonsteroidal antiinflammatory drug | Bacteroides fragilis, Bacteroides stercoris, Bacteroides thetaiotaomicron, Bacteroides uniformis, Bacteroides xylanisolvens and Odoribacter splanchnius | Human | - | - | - | - | [117] |
| Indomethacin-Glu (Hepatic metabolite of Indomethacin) | Nonsteroidal antiinflammatory drug | - | Rat | β-Glucuronidase | Indomethacin | Decrease toxicity | Hydrolysis  (Deconjugation) | [144] |
| Insulin | Endocrine drug | - | Human | Protease | - | Decrease efficacy | Hydrolysis  (Proteolysis) | [128] |
|  |  | - | Rat | Protease | - | Decrease efficacy | Hydrolysis  (Proteolysis) | [129] |
| Ipsalazide | Digestive-related drug | - | Human | Azo reductase | 5-Aminosalicylic acid | Decrease mutagenicity | Reduction  (Azo reduction) | [122] |
|  |  | - | Rat | Azo reductase | 5-Aminosalicylic acid | Decrease mutagenicity | Reduction  (Azo reduction) | [122] |
| Irbesartan | Endocrine drug | Bacteroides fragilis, Bacteroides thetaiotaomicron, Bacteroides uniformis, Bacteroides vulgatus, Bacteroides xylanisolvens, Odoribacter splanchnius and Parabacteroides johnsonii | Human | - | - | - | - | [117] |
| Irinotecan | Endocrine drug | - | Human | Carboxylesterase | SN38-G | Increase efficacy | Hydrolysis  (Deacylation) | [145] |
| Irsogladine maleate | Digestive-related drug | Bifidobacterium ruminatum | Human | - | - | - | - | [117] |
| Isradipine | Endocrine drug | Bacteroides fragilis, Bacteroides xylanisolvens, Blautia hansenii, Clostridium sp., Odoribacter splanchnius and Parabacteroides merdae | Human | - | - | - | - | [117] |
| Itraconazole | Antifungal agent | Akkermansia muciniphila, Bacteroides uniformis, Blautia hansenii, Blautia luti, Clostridium sp., Clostridium sporogenes, Odoribacter splanchnius and Roseburia intestinalis | Human | - | - | - | - | [117] |
| Ketoprofen-Glu (Hepatic metabolite of ketoprofen) | Nonsteroidal antiinflammatory drug | - | Rat | β-Glucuronidase | Ketoprofen | Decrease toxicity | Hydrolysis  (Deconjugation) | [144] |
| Ketorolac tromethamine | Nonsteroidal antiinflammatory drug | Bacteroides dorei, Bacteroides fragilis, Bacteroides uniformis, Bacteroides vulgatus, Bacteroides xylanisolvens, Clostridium sp. and Parabacteroides distasonis | Human | - | - | - | - | [117] |
|  |  | - | Rat | - | *m*-Hydroxyphenylacetic acid | - | Reduction (Dehydroxylation) and Hydrolysis (Dealkylation) | [146] |
|  |  | - | Human | - | *m*-Hydroxyphenylacetic acid | - | Reduction (Dehydroxylation) and Hydrolysis (Dealkylation) | [147, 148] |
|  |  | - | Human | Deearboxylase | - | - | Reduction (Dehydroxylation) and Hydrolysis (Dealkylation) | [149, 150] |
| L-Dopa | Natural products (Endogenous metabolite) | *Helicobacter pylori* | Human | - | m-Tyramine and m-hydroxyphenyl-acetic acid | - | Reduction (Dehydroxylation) and Hydrolysis (Dealkylation) | [151] |
|  |  | - | Rat | - | *m*-Hydroxyphenylacetic acid | - | Reduction (Dehydroxylation) and Hydrolysis (Dealkylation) | [146] |
|  |  | - | Human | - | *m*-Hydroxyphenylacetic acid | - | Reduction (Dehydroxylation) and Hydrolysis (Dealkylation) | [147, 148] |
|  |  | - | Human | Deearboxylase | - | - | Reduction (Dehydroxylation) and Hydrolysis (Dealkylation) | [149, 150] |
| Levamisole | Digestive-related drug | *Bacteroides distasonis,* *Bacteroides fragilis, Bacteroides ovatus, Bacteroides thetuiotaomicron, Bacteroides uniformis, Bacteroides erulgatus Clostridium barkeri,* *Clostridium radaveris Clostridium dostridiforme, Clostridium dificile, Clostridium innocuum, Clostridium oroticum, Clostridium sordellii, Clostridium sphenoides, Clostridium septicum, Escherichia moniliforme* and *Peptostreptococcus productus* | Human | - | levametabol-I, levametabol-II and levametabol-III | Decrease efficacy | Hydrolysis  (Thiazole ring-opening) | [152] |
|  |  | Anaerococcus hydrogenalis, Anaerotruncus colihominis, Bacteroides dorei, Bacteroides fragilis, Bacteroides stercoris, Bacteroides thetaiotaomicron, Bacteroides uniformis, Clostridium sporogenes, Escherichia coli and Parabacteroides johnsonii | Human | - | - | - | - | [117] |
| Levonorgestrel | Endocrine drug | Bacteroides caccae, Bacteroides cellulosilyticus, Bacteroides dorei, Bacteroides fragilis, Bacteroides thetaiotaomicron, Bacteroides uniformis, Bacteroides vulgatus, Bacteroides xylanisolvens, Clostridium hathewayi, Clostridium sp., Eubacterium biforme, Odoribacter splanchnius, Parabacteroides johnsonii, Parabacteroides merdae and  Ruminococcus gnavus | Human | - | - | - | - | [117] |
| Linagliptin | Endocrine drug | Bacteroides caccae, Bacteroides coprophilus ,  Bacteroides dorei, Bacteroides eggerthii, Bacteroides finegoldii, Bacteroides fragilis, Bacteroides stercoris, Bacteroides thetaiotaomicron, Bacteroides uniformis, Bacteroides xylanisolvens, Odoribacter splanchnius, Parabacteroides distasonis, Parabacteroides johnsonii and Pretovella copri | Human | - | - | - | - | [117] |
| Loperamide N-oxide | Digestive-related drug | Bacteroides coprophilus, Bacteroides dorei, Bacteroides fragilis, Bacteroides stercoris, Bacteroides uniformis, Bacteroides vulgatus, Bacteroides xylanisolvens, Blautia hansenii, Clostridium sp., Clostridium sporogenes, Escherichia coli, Odoribacter splanchnius  and Roseburia intestinalis | Human | - | - | - | Reduction  (N-oxide cleavage) | [117] |
|  |  | - | Human, rat and dog | N-oxide reductase | Loperamide | Increase efficacy | Reduction  (N-oxide cleavage) | [153, 154] |
| [Losartan](http://www.baidu.com/link?url=hAnD8yXtfaDqv6CdmPKoIbQbtdRUEl_4cNrSoqyOB3LbjgG8Jp4xAIidvtdFxs_aHSatKTsE7R8G2O_c-0Wpya) | Endocrine drug | Bacteroides dorei, Bacteroides fragilis, Bacteroides uniformis, Bacteroides vulgatus, Bacteroides xylanisolvens and Odoribacter splanchnius | Human | - | - | - | - | [117] |
| Lovastatin | Endocrine drug | Bacteroides cellulosilyticus DSM14838, Bacteroides dorei, Bacteroides fragilis, Bacteroides thetaiotaomicron, Bacteroides uniformis, Bacteroides vulgatus, Bacteroides xylanisolvens, Bifidobacterium adolescentis, Bifidobacterium breve, Blautia hansenii, Clostridium asparagiforme, Clostridium sp., Eubacterium biforme, Odoribacter splanchnius, Parabacteroides distasonis and Parabacteroides merdae | Human | - | - | - | - | [117] |
|  |  | - | Rat | - | Demethylbutyryl metabolite, hydroxylated metabolite, hydroxy acid metabolite and hydroxylated metabolite | Decrease efficacy | Hydrolysis (Dealkylation) | [155] |
| Mebendazole | Digestive-related drug | Bacteroides cellulosilyticus,  Bacteroides dorei, Bacteroides eggerthii, Bacteroides fragilis Bacteroides stercoris, Bacteroides thetaiotaomicron, Bacteroides uniformis, Bacteroides vulgatus, Bacteroides xylanisolvens, Bifidobacterium ruminatum, Blautia hansenii, Clostridium sp., Odoribacter splanchnius and Parabacteroides distasonis | Human | - | - | - | - | [117] |
| Mefloquine | Digestive-related drug | Eggerthella lenta, Escherichia coli, Providencia alcalifaciens and Roseburia intestinalis | Human | - | - | - | - | [117] |
| Megestrol acetate | Endocrine drug | Bacteroides dorei, Bacteroides fragilis, Bacteroides thetaiotaomicron, Bacteroides uniformis, Bacteroides vulgatus, Bacteroides xylanisolvens, Blautia hansenii, Clostridium hathewayi, Clostridium sp. and  Odoribacter splanchnius | Human | - | - | - | - | [117] |
| Melphalan | Antitumor drug | Akkermansia muciniphila, Alistipes indistinctus, Anaerotruncus colihominis, Anaerococcus hydrogenalis, Anaerostipes sp., Bacteroides caccae, Bacteroides cellulosilyticus, Bacteroides coprophilus, Bacteroides dorei, Bacteroides eggerthii, Bacteroides fragilis, Bacteroides finegoldii, Bacteroides intestinalis, Bacteroides ovatus, Bacteroides pectinophilus, Bacteroides stercoris, Bacteroides uniformis, Bacteroides vulgatus, Bacteroides xylanisolvens, Blautia hansenii, Blautia luti, Bifidobacterium adolescentis, Bifidobacterium breve, Bifidobacterium longum, Bifidobacterium ruminatum, Bacteroides thetaiotaomicron, Bryantia formatexigens, Clostridium asparagiforme, Clostridium bolteae, Clostridium difficile, Clostridium hathewayi, Clostridium scindens, Clostridium sp., Clostridium spiroforme, Clostridium sporogenes, Clostridium ramosum, Clostridium symbiosum, Collinsella aerofaciens, Collinsella intestinalis, Coprococcus comes, Dorea formicigenerans, Edwardsiella tarda, Eggerthella lenta, Enterobacter cancerogenus, Enterococcus faecalism, Escherichia coli, Eubacterium biforme, Eubacterium hallii, Eubacterium rectale, Eubacterium ventriosum, Limosilactobacillus reuteri, Odoribacter splanchnius, Parabacteroides distasonis, Parabacteroides johnsonii, Parabacteroides merdae, Pretovella copri, Proteus penneri, Providencia alcalifaciens, Providencia rettgeri, Providencia stuartii, Roseburia intestinalis, Ruminococcus gnavus, Ruminococcus lactaris, Ruminococcus torques, Salmonella Typhimurium, Subdoligranulum variabile and Victivallis vadensis | Human | - | - | - | - | [117] |
| Memantine | Nervous system drug | Bifidobacterium ruminatum | Human | - | - | - | - | [117] |
| Metaxalone | Nonsteroidal antiinflammatory drug | Bacteroides dorei, Bacteroides fragilis, Bacteroides pectinophilus, Bacteroides uniformis, Bacteroides vulgatus, Bacteroides xylanisolvens, Blautia hansenii, Clostridium sp., Parabacteroides distasonis and Pretovella copri | Human | - | - | - | - | [117] |
| Methamphetamine | Nervous system drug | *Enterobacteria, Enterococci, Lactobacilli, Clostridia, Bacteriodes* and *Bifidobacteria* | Pig | - | Amphetamine, 4'-hydroxyamphetamine and 4'-hydroxymethamphetamine | Decrease efficacy | Functional group removal (N-dealkylation) | [156] |
| Methiothepin maleate | Nervous system drug | Bacteroides uniformis | Human | - | - | - | - | [117] |
| Methotrexate | Antitumor drug | - | Rat | - | 4-Amino-4-deoxy-*N*^10^-methylpteroic acid | Decrease toxicity | Functional group removal (N-dealkylation) | [157] |
| Methoxsalen | Nonsteroidal antiinflammatory drug | Bacteroides caccae, Bacteroides dorei, Bacteroides fragilis, Bacteroides uniformis, Bacteroides vulgatus, Bacteroides xylanisolvens, Blautia hansenii, Clostridium ramosum, Clostridium sporogenes, Odoribacter splanchnius and Parabacteroides distasonis | Human | - | - | - | - | [117] |
| Methsuximide | Nervous system drug | Clostridium sp. | Human | - | - | - | - | [117] |
| Methylphenidate | Nervous system drug | Clostridium sp. and Eggerthella lenta | Human | - | - | - | - | [117] |
| Metronidazole | Antibiotic | *Clostridium perfringens* | Rat | Nitro reductase | Acetamide | Increase teratogenicity | Reduction  (Nitro reduction) | [158] |
|  |  | *Enterococcus gallinarum* and  *Enterococcus casseliflavus* | Human | Nitro reductase | - | Increase teratogenicity | Reduction  (Nitro reduction) | [159] |
| Mevastatin | Endocrine drug | Bacteroides cellulosilyticus, Bacteroides dorei, Bacteroides fragilis, Bacteroides intestinalis, Bacteroides pectinophilus, Bacteroides thetaiotaomicron, Bacteroides uniformis, Bacteroides vulgatus, Bifidobacterium adolescentis, Bifidobacterium breve, Limosilactobacillus reuteri, Odoribacter splanchnius, Parabacteroides distasonis and Parabacteroides merdae | Human | - | - | - | - | [117] |
| Mifepristone | Endocrine drug | Bacteroides dorei, Bacteroides fragilis and Bacteroides vulgatus | Human | - | - | - | - | [117] |
| Misonidazole | Antihistamine agent | *Clostridium perfringens* | Rat | Nitro reductase | 1(2-Aminoimidazol 1-yl) 3-methoxypropan 2-ol | - | Reduction  (Nitro reduction) | [160] |
| Misoprostol | Digestive-related drug | - | Human | Methyl hydrolase | Misoprostol acid | Increase efficacy | Hydrolysis (Dealkylation) | [130] |
| Morphine-Glu (Hepatic metabolite of morphine) | Nervous system drug | - | Rat | β-Glucuronidase | Morphine | Enterohepatic circulation | Hydrolysis  (Deconjugation) | [161] |
| Mycophenolate mofetil | Immunosuppressant | - | Human | - | Mycophenolic acid | Increase gastrointestinal toxicity | Hydrolysis (Dealkylation) | [130] |
|  |  | Akkermansia muciniphila, Anaerotruncus colihominis, Anaerococcus hydrogenalis, Anaerostipes sp., Bacteroides coprophilus, Bacteroides dorei, Bacteroides eggerthii, Bacteroides fragilis, Bacteroides intestinalis, Bacteroides ovatus, Bacteroides pectinophilus, Bacteroides stercoris, Bacteroides uniformis, Bacteroides vulgatus, Bacteroides xylanisolvens, Blautia hansenii, Blautia luti, Bifidobacterium adolescentis, Bifidobacterium breve, Bifidobacterium longum, Bifidobacterium ruminatum, Bacteroides thetaiotaomicron, Bryantia formatexigens, Clostridium asparagiforme, Clostridium bolteae, Clostridium difficile, Clostridium hathewayi, Clostridium scindens, Clostridium sp., Clostridium spiroforme, Clostridium sporogenes, Clostridium ramosum, Collinsella intestinalis, Coprococcus comes, Eggerthella lenta, Enterobacter cancerogenus, Enterococcus faecalism, Eubacterium biforme, Eubacterium hallii, Eubacterium rectale, Limosilactobacillus reuteri, Odoribacter splanchnius, Parabacteroides distasonis, Parabacteroides johnsonii, Parabacteroides merdae, Pretovella copri, Providencia alcalifaciens, Providencia rettgeri, Providencia stuartii, Roseburia intestinalis, Ruminococcus torques, Salmonella Typhimurium, Subdoligranulum variabile and Victivallis vadensis | Human | - | - | - | - | [117] |
| N-Acetyl-aminophenol | Nonsteroidal antiinflammatory drug | - | Rat | N-deacetylase | Aminophenol | - | Functional group removal (N-dealkylation) | [66] |
| N-hydroxy-N-2-fluorenylacetamide | Antitumor drug | *Escherichia coli* | Rat | - | N-2-fluorenylacetamide | Decrease carcinogenicity | Functional group removal (N-dealkylation) | [162] |
| Nafronyl oxalate | Nervous system drug | Bacteroides uniformis and Bacteroides vulgatus | Human | - | - | - | - | [117] |
| Naftopidil | Endocrine drug | Bacteroides fragilis | Human | - | - | - | - | [117] |
| Naloxone | Nervous system drug | Bacteroides coprophilus, Bacteroides fragilis, Bacteroides ovatus, Bacteroides thetaiotaomicron and Bacteroides uniformis | Human | - | - | - | - | [117] |
| Naproxen | Nonsteroidal antiinflammatory drug | Bacteroides fragilis, Bacteroides uniformis, Bacteroides vulgatus and Odoribacter splanchnius | Human | - | - | - | - | [117] |
| Naringin | Natural drug | *Clostridium orbiscindens* and  *Eubacterium ramulus* | Human | β-Glucuronidase | 3-(4-Hydroxyphenyl) propionic acid and phlorogucinol | Increase efficacy and increase toxicity | Hydrolysis  (Deconjugation) | [163-165] |
| Nateglinide | Endocrine drug | Bacteroides fragilis, Bacteroides thetaiotaomicron, Bacteroides uniformis, Bacteroides vulgatus, Blautia hansenii, Odoribacter splanchnius and Parabacteroides distasonis | Human | - | - | - | - | [117] |
| Nefazodone | Nervous system drug | Clostridium asparagiforme | Human | - | - | - | - | [117] |
| Neoprontosil | Antibiotic | *Proteus* | Rat | Azo reductase | - | - | Reduction  (Azo reduction) | [58, 59] |
|  |  | - | Rat | Azo reductase | Sulfanilamide | Increase efficacy | Reduction  (Azo reduction) | [166] |
| Neostigmine bromide | Nervous system drug | Bacteroides coprophilus, Bacteroides dorei, Bacteroides fragilis, Bacteroides ovatus, Bacteroides thetaiotaomicron, Bacteroides uniformis and Parabacteroides merdae | Human | - | - | - | - | [117] |
| Nevirapine | Antiviral drug | Blautia hansenii and Clostridium sp. | Human | - | - | - | - | [117] |
| Nicardipine | Endocrine drug | - | Human | Nitro reductase | Aminonlicardipine | - | Reduction  (Nitro reduction) | [130] |
| Nicergoline | Nervous system drug | Bacteroides uniformis, Clostridium difficile, Clostridium sp., Escherichia coli, Parabacteroides distasonis and Salmonella Typhimurium | Human | - | - | - | - | [117] |
| Nicotine-1′-N-oxide | Natural drug | - | Human | N-oxide reductase | N-oxide, cotinine and nicotine | Decrease efficacy | Reduction  (N-oxide cleavage) | [167] |
| Nitrazepam | Nervous system drug | *Clostridium leptum* | Human | Nitro reductase | 7-Aminonitrazepam | Increase teratogenicity | Reduction  (Nitro reduction) | [168] |
|  |  | - | Rat | Nitro reductases | 7-Aminonitrazepam | Increase teratogenicity | Reduction  (Nitro reduction) | [169, 170] |
| Nitrendipine | Endocrine drug | Alistipes indistinctus, Anaerotruncus colihominis, Anaerococcus hydrogenalis, Anaerostipes sp., Bacteroides caccae, Bacteroides cellulosilyticus, Bacteroides dorei, Bacteroides eggerthii, Bacteroides fragilis, Bacteroides finegoldii, Bacteroides intestinalis, Bacteroides stercoris, Bacteroides uniformis, Bacteroides vulgatus, Bacteroides xylanisolvens, Blautia hansenii, Blautia luti, Bifidobacterium adolescentis, Bifidobacterium longum, Bifidobacterium ruminatum, Bacteroides thetaiotaomicron, Bryantia formatexigens, Clostridium asparagiforme, Clostridium bolteae, Clostridium difficile, Clostridium hathewayi, Clostridium scindens, Clostridium sp., Clostridium spiroforme, Clostridium sporogenes, Clostridium ramosum, Clostridium symbiosum, Collinsella aerofaciens, Collinsella intestinalis, Coprococcus comes, Dorea formicigenerans, Eggerthella lenta, Eubacterium biforme, Eubacterium hallii, Eubacterium rectale, Eubacterium ventriosum, Odoribacter splanchnius, Parabacteroides distasonis, Parabacteroides johnsonii, Parabacteroides merdae, Pretovella copri, Roseburia intestinalis, Ruminococcus gnavus, Ruminococcus lactaris, Subdoligranulum variabile and Victivallis vadensis | Human | - | - | - | - | [117] |
| Nitrofurantoin | Antibiotic | *Clostridium perfringens ATCC 3626, Clostridium paraputrificum NR1, Clostridium* sp. *strain NR8, Clostridium* sp. *strain NR9* and  *Clostridium leptum NP15* | Human | Nitro reductase | - | Increase efficacy | Reduction  (Nitro reduction) | [171] |
| Nizatidine | Digestive-related drug | *Bacteroides dorei, Bacteroides fragilis, Bacteroides thetaiotaomicron, Bacteroides uniformis, Bifidobacterium breve* and *Parabacteroides johnsonii* | Human | - | - | - | Reduction  (N-oxide cleavage) | [117] |
|  |  | - | Human | N-oxide reductase | - | Decrease efficacy | Reduction  (N-oxide cleavage) | [172] |
| Norethindrone acetate | Endocrine drug | Alistipes indistinctus, Bacteroides caccae, Bacteroides cellulosilyticus, Bacteroides coprophilu, Bacteroides dorei, Bacteroides eggerthii, Bacteroides fragilis, Bacteroides fragilis, Bacteroides intestinalis, Bacteroides ovatus, Bacteroides stercoris, Bacteroides thetaiotaomicron, Bacteroides uniformis, Bacteroides vulgatus, Bacteroides xylanisolvens, Blautia hansenii, Clostridium sp., Eubacterium biforme, Odoribacter splanchnius, Parabacteroides johnsonii, Parabacteroides merdae, Ruminococcus gnavus and  Ruminococcus torques | Human | - | - | - | Hydrolysis  (Deacylation) | [117] |
| Norgestimate | Endocrine drug | Bacteroides fragilis, Blautia hansenii, Clostridium sp., Eggerthella lenta, Escherichia coli, Eubacterium biforme, Eubacterium rectale  and Ruminococcus torques | Human | - | - | - | - | [117] |
| Noscapine | Nervous system drug | Bryantia formatexigens, Eggerthella lenta  and Eubacterium biforme | Human | - | - | - | - | [117] |
| Olanzapine | Nervous system drug | Bacteroides coprophilus, Bacteroides fragilis  and Odoribacter splanchnius | Human | - | - | - | - | [117] |
|  |  | - | Rat | - | - | - | - | [173] |
| Olmesartan medoxomil | Endocrine drug | Anaerotruncus colihominis, Anaerococcus hydrogenalis, Anaerostipes sp., Bacteroides caccae, Bacteroides cellulosilyticus, Bacteroides coprophilus, Bacteroides dorei, Bacteroides fragilis, Bacteroides finegoldii, Bacteroides intestinalis, Bacteroides stercoris, Bacteroides uniformis, Bacteroides vulgatus, Bacteroides xylanisolvens, Blautia hansenii, Blautia luti, Bifidobacterium breve, Bifidobacterium longum, Bryantia formatexigens, Clostridium asparagiforme, Clostridium bolteae, Clostridium difficile, Clostridium hathewayi, Clostridium scindens, Clostridium spiroforme, Clostridium sporogenes, Clostridium symbiosum, Collinsella aerofaciens, Coprococcus comes, Dorea formicigenerans, Edwardsiella tarda, Eggerthella lenta, Enterobacter cancerogenus, Escherichia coli, Eubacterium biforme, Eubacterium hallii, Eubacterium rectale, Eubacterium ventriosum, Limosilactobacillus reuteri, Odoribacter splanchnius, Parabacteroides distasonis, Parabacteroides johnsonii, Pretovella copri, Proteus penneri, Providencia stuartii, Ruminococcus lactaris, Salmonella Typhimurium, Subdoligranulum variabile and Victivallis vadensis | Human | - | - | - | - | [117] |
| Olsalazine | Digestive-related drug | - | Human | Azo reductase | 5-Aminosalicylic acid (mesalazine) | Increase efficacy | Reduction  (Azo reduction) | [123, 130, 174] |
|  |  | - | Horse | Azo reductase | 5-Aminosalicylic acid (mesalazine) | Increase efficacy | Reduction  (Azo reduction) | [175] |
| Omeprazole | Digestive-related drug | Akkermansia muciniphila, Alistipes indistinctus, Anaerotruncus colihominis, Anaerococcus hydrogenalis, Anaerostipes sp., Bacteroides caccae, Bacteroides cellulosilyticus, Bacteroides coprophilus, Bacteroides dorei, Bacteroides eggerthii, Bacteroides fragilis, Bacteroides finegoldii, Bacteroides intestinalis, Bacteroides ovatus, Bacteroides pectinophilus, Bacteroides stercoris, Bacteroides uniformis, Bacteroides vulgatus, Bacteroides xylanisolvens, Blautia hansenii, Blautia luti, Bifidobacterium adolescentis, Bifidobacterium breve, Bifidobacterium longum, Bifidobacterium ruminatum, Bacteroides thetaiotaomicron, Bryantia formatexigens, Clostridium asparagiforme, Clostridium bolteae, Clostridium difficile, Clostridium hathewayi, Clostridium scindens, Clostridium sp., Clostridium spiroforme, Clostridium sporogenes, Clostridium ramosum, Clostridium symbiosum, Collinsella aerofaciens, Collinsella intestinalis, Coprococcus comes, Dorea formicigenerans, Edwardsiella tarda, Eggerthella lenta, Enterobacter cancerogenus, Enterococcus faecalism, Escherichia coli, Eubacterium biforme, Eubacterium hallii, Eubacterium rectale, Eubacterium ventriosum, Limosilactobacillus reuteri, Odoribacter splanchnius, Parabacteroides distasonis, Parabacteroides johnsonii, Parabacteroides merdae, Pretovella copri, Proteus penneri, Providencia alcalifaciens, Providencia rettgeri, Providencia stuartii, Roseburia intestinalis, Ruminococcus gnavus, Ruminococcus lactaris, Ruminococcus torques, Salmonella Typhimurium, Subdoligranulum variabile and Victivallis vadensis | Human | - | - | - | Reduction  (Sulfoxide reduction) | [117] |
|  |  | *Bacteroides* | Rat | Sulfoxide reductase | - | - | Reduction  (Sulfoxide reduction) | [176] |
| Oxaprozine | Nonsteroidal antiinflammatory drug | Bacteroides dorei, Bacteroides fragilis, Bacteroides stercoris, Bacteroides thetaiotaomicron, Bacteroides uniformis, Bacteroides vulgatus, Bacteroides xylanisolvens, Clostridium sp. and Odoribacter splanchnius | Human | - | - | - | - | [117] |
| Oxybutynin chloride | Nervous system drug | Bacteroides dorei, Bacteroides fragilis, Bacteroides uniformis, Bacteroides vulgatus  and Clostridium sp. | Human | - | - | - | - | [117] |
| Paclitaxel | Antitumor drug | Bacteroides fragilis, Bacteroides thetaiotaomicron, Bacteroides uniformis, Bacteroides vulgatus, Bacteroides xylanisolvens, Clostridium sp., Eggerthella lenta, Escherichia coli and Odoribacter splanchnius | Human | - | - | - | - | [117] |
| Paliperidone | Nervous system drug | Alistipes indistinctus, Anaerococcus hydrogenalis, Anaerotruncus colihominis, Bacteroides caccae, Bacteroides fragilis, Bifidobacterium breve, Blautia luti, Clostridium asparagiforme, Clostridium scindens, Clostridium sp., Clostridium sporogenes, Clostridium symbiosum, Collinsella intestinalis, Coprococcus comes, Enterobacter cancerogenus, Eubacterium hallii, Eubacterium rectale, Odoribacter splanchnius, Roseburia intestinalis, Ruminococcus lactaris and Subdoligranulum variabile | Human | - | - | - | - | [117] |
| Pantoprazole | Digestive-related drug | Akkermansia muciniphila, Alistipes indistinctus, Anaerotruncus colihominis, Anaerococcus hydrogenalis, Anaerostipes sp., Bacteroides caccae, Bacteroides cellulosilyticus, Bacteroides coprophilus, Bacteroides dorei, Bacteroides eggerthii, Bacteroides fragilis, Bacteroides finegoldii, Bacteroides intestinalis, Bacteroides ovatus, Bacteroides pectinophilus, Bacteroides stercoris, Bacteroides uniformis, Bacteroides vulgatus, Bacteroides xylanisolvens, Blautia hansenii, Blautia luti, Bifidobacterium adolescentis, Bifidobacterium breve, Bifidobacterium longum, Bifidobacterium ruminatum, Bacteroides thetaiotaomicron, Bryantia formatexigens, Clostridium asparagiforme, Clostridium bolteae, Clostridium difficile, Clostridium hathewayi, Clostridium scindens, Clostridium sp., Clostridium spiroforme, Clostridium sporogenes, Clostridium ramosum, Clostridium symbiosum, Collinsella aerofaciens, Collinsella intestinalis, Coprococcus comes, Dorea formicigenerans, Edwardsiella tarda, Eggerthella lenta, Enterobacter cancerogenus, Enterococcus faecalism, Escherichia coli, Eubacterium biforme, Eubacterium hallii, Eubacterium rectale, Eubacterium ventriosum, Limosilactobacillus reuteri, Odoribacter splanchnius, Parabacteroides distasonis, Parabacteroides johnsonii, Parabacteroides merdae, Pretovella copri, Proteus penneri, Providencia alcalifaciens, Providencia rettgeri, Providencia stuartii, Roseburia intestinalis, Ruminococcus gnavus, Ruminococcus lactaris, Ruminococcus torques, Salmonella Typhimurium, Subdoligranulum variabile and Victivallis vadensis | Human | - | - | - | - | [117] |
| Penbutolol sulfate | Nervous system drug | Bacteroides uniformis, Bacteroides vulgatus  and Clostridium sp. | Human | - | - | - | - | [117] |
| Periciazine | Nervous system drug | Alistipes indistinctus, Bacteroides caccae, Bacteroides dorei, Bacteroides fragilis, Bacteroides thetaiotaomicron, Bacteroides vulgatus, Parabacteroides distasonis  and Parabacteroides johnsonii | Human | - | - | - | - | [117] |
| p-Ethoxyglycolanilide | Nonsteroidal antiinflammatory drug | - | Rat | N-deacetylase | p-Phenetidine | - | Hydrolysis  (Deacylation) | [66] |
| Phenacetin | Nervous system drug | - | Rat | N-deacetylase | p-Phenetidine | - | Hydrolysis  (Deacylation) | [66] |
| Phenazopyridine | Nervous system drug | Alistipes indistinctus, Anaerotruncus colihominis, Anaerococcus hydrogenalis, Anaerostipes sp., Bacteroides cellulosilyticus, Bacteroides dorei, Bacteroides fragilis, Bacteroides finegoldii, Bacteroides intestinalis, Bacteroides ovatus, Bacteroides uniformis, Bacteroides vulgatus, Bacteroides xylanisolvens, Blautia hansenii, Blautia luti, Bifidobacterium breve, Bifidobacterium ruminatum, Bacteroides thetaiotaomicron, Bryantia formatexigens, Clostridium bolteae, Clostridium difficile, Clostridium hathewayi, Clostridium scindens, Clostridium sp., Clostridium spiroforme, Clostridium sporogenes, Clostridium ramosum, Clostridium symbiosum, Collinsella aerofaciens, Collinsella intestinalis, Coprococcus comes, Dorea formicigenerans, Eggerthella lenta, Enterobacter cancerogenus, Enterococcus faecalism, Eubacterium biforme, Eubacterium hallii, Eubacterium rectale, Eubacterium ventriosum, Odoribacter splanchnius, Parabacteroides distasonis, Parabacteroides johnsonii, Parabacteroides merdae, Proteus penneri, Providencia alcalifaciens, Roseburia intestinalis, Ruminococcus gnavus, Ruminococcus lactaris, Ruminococcus torques, Subdoligranulum variabile  and Victivallis vadensis | Human | - | - | - | - | [117] |
| Phenytoin sodium | Nervous system drug | Bacteroides dorei, Bacteroides uniformis  and Bacteroides vulgatus | Human | - | - | - | - | [117] |
| Pidotimod | Immunosuppressant | Bacteroides coprophilus, Bacteroides dorei, Bacteroides ovatus, Bacteroides stercoris, Bacteroides thetaiotaomicron, Bacteroides uniformis, Blautia hansenii and Clostridium sp. | Human | - | - | - | - | [117] |
| Pimozide | Nervous system drug | Akkermansia muciniphila, Clostridium bolteae, Clostridium sp. and Eggerthella lenta | Human | - | - | - | - | [117] |
| p-methoxyacetanilide,  p-methoxyglycolanilide and  p-methoxyoxanilic acid | Nonsteroidal antiinflammatory drug | - | Rat | N-deacetylase | p-Anisidine | - | Hydrolysis  (Deacylation) | [66] |
| Potassium oxonate | Antitumor drug | - | Rat | - | Cyanuric acid | - | Reduction | [113] |
| Pranoprofen | Nonsteroidal antiinflammatory drug | Alistipes indistinctus, Bacteroides coprophilus, Bacteroides dorei, Bacteroides fragilis, Bacteroides ovatus, Bacteroides pectinophilus, Bacteroides stercoris, Bacteroides thetaiotaomicron, Bacteroides uniformis, Bacteroides vulgatus, Bacteroides xylanisolvens, Clostridium difficile, Clostridium sp., Eubacterium biforme, Odoribacter splanchnius, Parabacteroides distasonis and Pretovella copri | Human | - | - | - | - | [117] |
| Prednisone | Endocrine drug | Anaerostipes sp., Bacteroides coprophilus, Bacteroides dorei, Bacteroides fragilis, Bacteroides stercoris, Bacteroides uniformis, Bacteroides vulgatus, Bifidobacterium ruminatum, Dorea formicigenerans, Eggerthella lenta, Enterobacter cancerogenus, Eubacterium biforme, Parabacteroides johnsonii, Providencia stuartii, Ruminococcus gnavus, Ruminococcus lactaris  and Salmonella Typhimurium | Human | - | - | - | Functional group removal  (N-dealkylation) | [117] |
| Primaquine phosphate | Antimalarial drug | Bacteroides pectinophilus, Clostridium bolteae, Clostridium symbiosum and  Parabacteroides distasonis | Human | - | - | - | - | [117] |
| Procarbazine | Antitumor drug | Bacteroides dorei, Bacteroides fragilis, Bacteroides stercoris, Bacteroides uniformis, Bacteroides vulgatus, Bacteroides xylanisolvens, Clostridium sp. and Odoribacter splanchnius | Human | - | - | - | - | [117] |
| Prontosil | Antibiotic | - | Rat | Azo reductase | Sulfanilamide | Increase efficacy | Reduction  (Azo reduction) | [166] |
| Quercetin-3-glucoside | - | *Eubacterium ramulus* and *Enterococcus casseliflavus* | Rat | β-glucuronidase | Quercetin, 3,4-dihydroxyphenylacetic acid and isorhamnetin | Increase efficacy | Hydrolysis  (Deconjugation) | [177] |
|  | - | *-* | Human | β-glucuronidase | Quercetin | Increase efficacy | Hydrolysis  (Deconjugation) | [178, 179] |
| Quinacrine | Antimalarial drug | Bacteroides fragilis | Human | - | - | - | - | [117] |
| Quinapril | Endocrine drug | Bacteroides caccae, Bacteroides cellulosilyticus, Bacteroides dorei, Bacteroides fragilis, Bacteroides intestinalis, Bacteroides pectinophilus, Bacteroides stercoris, Bacteroides thetaiotaomicron, Bacteroides uniformis, Bacteroides vulgatus, Bacteroides xylanisolvens, Bifidobacterium ruminatum, Blautia hansenii, Clostridium difficile, Clostridium sp., Eubacterium biforme, Odoribacter splanchnius, Parabacteroides distasonis and Pretovella copri | Human | - | - | - | - | [117] |
| Racecadotril | Digestive-related drug | Akkermansia muciniphila, Bacteroides caccae, Bacteroides cellulosilyticus, Bacteroides coprophilus, Bacteroides dorei, Bacteroides finegoldii, Bacteroides fragilis, Bacteroides intestinalis, Bacteroides ovatus, Bacteroides pectinophilus, Bacteroides stercoris, Bacteroides thetaiotaomicron, Bacteroides uniformis, Bacteroides vulgatus, Bacteroides xylanisolvens, Bifidobacterium adolescentis, Bifidobacterium breve, Bifidobacterium longum, Blautia hansenii, Clostridium asparagiforme, Clostridium difficile, Clostridium hathewayi, Clostridium sp., Clostridium sporogenes, Eubacterium rectale, Eubacterium ventriosum, Odoribacter splanchnius, Parabacteroides distasonis, Parabacteroides johnsonii, Parabacteroides merdae, Pretovella copri, Ruminococcus lactaris, Subdoligranulum variabile and Victivallis vadensis | Human | - | - | - | - | [117] |
| Ramelteon | Nervous system drug | Bacteroides dorei, Bacteroides fragilis, Bacteroides thetaiotaomicron, Bacteroides uniformis, Bacteroides vulgatus, Bacteroides xylanisolvens, Blautia hansenii, Odoribacter splanchnius and Parabacteroides distasonis | Human | - | - | - | - | [117] |
| Ramipril | Endocrine drug | Bacteroides fragilis, Bacteroides xylanisolvens, Bacteroides vulgatus and Bacteroides uniformis | Human | - | - | - | - | [117] |
| Ranitidine | Antiviral drug | *Bacteroides fragilis* and *Bifidobacterium breve* | Human | - | - | - | Reduction  (N-oxide cleavage) | [117] |
|  |  | - | Human | N-oxide reductase | - | Decrease efficacy | Reduction  (N-oxide cleavage) | [180] |
| Rebamipide | Digestive-related drug | Bacteroides dorei, Bacteroides fragilis, Bacteroides ovatus, Bacteroides stercoris, Bacteroides thetaiotaomicron, Bacteroides uniformis, Bacteroides vulgatus, Bifidobacterium ruminatum, Parabacteroides distasonis  and Salmonella Typhimurium | Human | - | - | - | - | [117] |
| Repaglinide | Endocrine drug | Bacteroides thetaiotaomicron, Bacteroides uniformis and Bacteroides xylanisolvens | Human | - | - | - | - | [117] |
| Reserpine | Endocrine drug | Anaerotruncus colihominis, Bacteroides dorei, Bacteroides vulgatus, Clostridium bolteae, Clostridium hathewayi and Clostridium sp. | Human | - | - | - | - | [117] |
| Riluzole | Nervous system drug | Akkermansia muciniphila, Bacteroides caccae, Bacteroides dorei, Bacteroides eggerthii, Bacteroides fragilis, Bacteroides stercoris, Bacteroides thetaiotaomicron, Bacteroides uniformis, Bacteroides vulgatus, Bacteroides xylanisolvens, Bifidobacterium ruminatum, Blautia hansenii, Clostridium sp., Clostridium sporogenes, Odoribacter splanchnius, Parabacteroides distasonis, Pretovella copri and  Roseburia intestinalis | Human | - | - | - | - | [117] |
| Risperidone | Nervous system drug | *Alistipes indistinctus, Anaerococcus hydrogenalis, Anaerotruncus colihominis, Bacteroides caccae, Bacteroides fragilis, Bifidobacterium breve, Blautia hansenii, Blautia luti, Bryantia formatexigens, Clostridium difficile, Clostridium hathewayi, Clostridium scindens, Clostridium* sp.*, Clostridium sporogenes, Clostridium symbiosum, Collinsella intestinalis, Coprococcus comes, Eggerthella lenta, Enterobacter cancerogenus, Enterococcus faecalis, Eubacterium biforme, Eubacterium hallii, Odoribacter splanchnius , Roseburia intestinalis, Ruminococcus gnavus, Ruminococcus lactaris* and  *Subdoligranulum variabile* | Human | - | - | - | Reduction  (Benzisoxazole ring reduction) | [117] |
| Ritonavir | Antiviral drug | Bacteroides dorei, Bacteroides eggerthii, Bacteroides fragilis, Bacteroides uniformis, Bacteroides vulgatus, Bacteroides xylanisolvens, Blautia hansenii, Escherichia coli and  Odoribacter splanchnius | Human | - | - | - | - | [117] |
| Rizatriptan benzoate | Nervous system drug | Bacteroides coprophilus | Human | - | - | - | - | [117] |
| Rosuvastatin calcium | Endocrine drug | Bacteroides fragilis | Human | - | - | - | - | [117] |
| Roxatidine acetate | Digestive-related drug | Alistipes indistinctus, Bacteroides caccae, Bacteroides cellulosilyticus, Bacteroides dorei  Bacteroides eggerthii, Bacteroides fragilis, Bacteroides intestinalis, Bacteroides ovatus, Bacteroides pectinophilus, Bacteroides stercoris, Bacteroides thetaiotaomicron, Bacteroides uniformis, Bacteroides vulgatus, Bacteroides xylanisolvens, Escherichia coli, Eubacterium rectale, Odoribacter splanchnius, Parabacteroides distasonis, Parabacteroides johnsonii, Parabacteroides merdae, Pretovella copri, Salmonella Typhimurium, Subdoligranulum variabile and Victivallis vadensis | Human | - | - | - | - | [117] |
| Secretin | Endocrine drug | - | Human | Protease | - | Decrease efficacy | Hydrolysis  (Proteolysis) | [128] |
| Sildenafil citrate | Nervous system drug | Blautia hansenii and Clostridium sp. | Human | - | - | - | - | [117] |
| Simvastatin | Digestive-related drug | - | Human | - | 2-Hydroxyisovaleric acid, 3-hydroxybutanoic acid, lactic acid and cyclohexanecarboxylic acid | - | Functional group removal  (N-dealkylation) | [181] |
| SN38-G (Hepatic metabolite of Irinotecan) | Antitumor drug | *Escherichia coli,* *Staphylococcus* sp.  and *Clostridium* sp. | Human | β-Glucuronidase | SN38 | Increase toxicity and enterohepatic circulation | Hydrolysis  (Deconjugation) | [145, 154, 182] |
|  |  | *Escherichia coli,* *Staphylococcus* sp. and *Clostridium* sp. | Rat | β-Glucuronidase | SN38 | Increase toxicity and enterohepatic circulation | Hydrolysis  (Deconjugation) | [183] |
| Sodium picosulfate | Digestive-related drug | - | Human | Sulfatase | 4,4'-Dihydroxydiphenyl-(2-pyridyl) methane | Increase efficacy | Hydrolysis  (Desulfation) | [184] |
| Somatostatin | Endocrine drug | - | Human | Protease | - | Decrease efficacy | Hydrolysis  (Proteolysis) | [128] |
| Sorivudine | Digestive-related drug | *Bacteroides* | Rat | Dihydropyrimidine dehydrogenase | (E)-5-(2-bromovinyl) uracil | Increase toxicity | Functional group removal  (N-dealkylation) | [185] |
|  |  | *Bacteroides vulgatus, Bacteroides thetaiotaomicron, Bacteroides fragilis, Bacteroides uniformis* and *Bacteroides eggerthii* | Rat | - | (E)-5-(2-bromovinyl) uracil | Increase toxicity | Functional group removal  (N-dealkylation) | [186] |
| Sotalol | Endocrine drug | Bacteroides fragilis, Bacteroides ovatus, Bacteroides thetaiotaomicron, Bacteroides thetaiotaomicron and Bacteroides uniformis | Human | - | - | - | - | [117] |
| Spironolactone | Endocrine drug | - | Human | Thioesterase | 7α-Thiospironolactone | Increase efficacy | Hydrolysis  (Deacylation) | [130] |
| Succinyl sulfathiazole | Antibiotic | - | Rat | - | Sulfathiazole | - | Functional group removal  (N-dealkylation) | [187] |
| Sulfapyridine and 5-aminosalicylate  (Hepatic metabolite of Sulfasalazine) | Digestive-related drug | - | Human | Acetyl transferase | AC-5- aminosalicylate | Decrease efficacy | Functional group removal (Acetylation) | [188] |
|  |  | - | Rat, dog, pig and human | Acetyl transferase | AC-sulfapyridine and AC-5- aminosalicylate | Decrease efficacy | Functional group removal (Acetylation) | [188] |
| Sulfasalazine | Digestive-related drug | Alistipes indistinctus, Anaerotruncus colihominis, Anaerococcus hydrogenalis, Anaerostipes sp., Bacteroides caccae, Bacteroides cellulosilyticus, Bacteroides dorei, Bacteroides eggerthii, Bacteroides fragilis, Bacteroides finegoldii, Bacteroides intestinalis, Bacteroides pectinophilus, Bacteroides uniformis, Bacteroides vulgatus, Bacteroides xylanisolvens, Blautia hansenii, Blautia luti, Bifidobacterium adolescentis, Bifidobacterium breve, Bifidobacterium longum, Bifidobacterium ruminatum, Bacteroides thetaiotaomicron, Clostridium asparagiforme, Clostridium bolteae, Clostridium difficile, Clostridium hathewayi, Clostridium scindens, Clostridium sp., Clostridium spiroforme, Clostridium sporogenes, Clostridium ramosum, Clostridium symbiosum, Collinsella aerofaciens, Collinsella intestinalis, Coprococcus comes, Dorea formicigenerans, Eggerthella lenta, Enterobacter cancerogenus, Enterococcus faecalism, Escherichia coli, Eubacterium biforme, Eubacterium hallii, Eubacterium rectale, Eubacterium ventriosum, Limosilactobacillus reuteri, Odoribacter splanchnius, Parabacteroides distasonis, Parabacteroides johnsonii, Parabacteroides merdae, Proteus penneri, Providencia stuartii, Roseburia intestinalis, Ruminococcus gnavus, Ruminococcus lactaris, Ruminococcus torques, Salmonella Typhimurium, Subdoligranulum variabile and Victivallis vadensis | Human | Azo reductase | - | - | Reduction  (Azo reduction) | [117] |
|  |  | - | Human | Azo reductase | 5-Aminosalicylic acid (mesalazine) and sulfapyridine | Increase efficacy | Reduction  (Azo reduction) | [123, 130] |
|  |  | - | Rat | Azo reductase | 5-Aminosalicylate, sulfapyridine and their metabolites | Increase efficacy | Reduction  (Azo reduction) | [189] |
|  | Antibiotic | *Escherichia coli, Bacteroldes species, Streptococcus faecalis, Klebsiella aerogenes, Pseudomonas pyrocyanea, Staphylococcus aureus, Pneumococcus, Streptococcus haemolyticus B*  and *Proteus vulgaris* | - | Azo reductase | - | - | Reduction  (Azo reduction) | [190] |
| Sulfinpyrazone | Nonsteroidal antiinflammatory drug | *Bacteroides fragilis, Bacteroides thetaiotaomicron, Bacteroides xylanisolvens, Eggerthella lenta, Escherichia coli* and *Odoribacter splanchnius* | Human | - | - | Increase efficacy | Reduction  (Sulfoxide reduction) | [117] |
|  |  | *Escherichia coli* | Rat | Sulfatase | - | - | Reduction  (Sulfoxide reduction) | [67] |
|  |  | *Escherichia coli, Enterobacier, Proteur* sp.*, Providencia, Klebsiella* sp.*, Citrobacter, Psadomonas* sp.*, Enterococci, Eubacteria, Bacteroides* sp.*, Fusobacteria* and  *Clostridia, Bifidobacteria* | Human | Sulfatase | Sulfide and sulfinpyrazone sulfide | Increase efficacy | Reduction  (Sulfoxide reduction) | [191, 192] |
|  |  | *Escherichia coli, Enterobacier, Proteur* sp.*, Providencia, Klebsiella* sp.*, Citrobacter, Psadomonas* sp.*, Enterococci, Eubacteria, Bacteroides* sp.*, Fusobacteria* and  *Clostridia, Bifidobacteria* | Rabbit | Sulfatase | Sulfide and sulfinpyrazone sulfide | Increase efficacy | Reduction  (Sulfoxide reduction) | [191] |
|  |  | - | Rat | Sulfatase | Sulfide and sulfinpyrazone sulfide | Increase efficacy | Reduction  (Sulfoxide reduction) | [193] |
| Sulindac | Nonsteroidal antiinflammatory drug | *Bacteroides fragilis* | Human | - | - | - | Reduction  (Sulfoxide reduction) | [117] |
|  |  | *Escherichia coli* | Rat | Sulfatase | - | - | Reduction  (Sulfoxide reduction) | [67] |
|  |  | *Escherichia coli, Enterobacier, Proteur* sp.*, Providencia, Klebsiella* sp.*, Citrobacter, Psadomonas* sp.*, Enterococci, Eubacteria, Bacteroides* sp.*, Fusobacteria* and  *Clostridia, Bifidobacteria* | Human | Sulfatase | Sulfide | Increase efficacy | Reduction  (Sulfoxide reduction) | [191, 194] |
|  |  | *Escherichia coli, Enterobacier, Proteur* sp.*, Providencia, Klebsiella* sp.*, Citrobacter, Psadomonas* sp.*, Enterococci, Eubacteria, Bacteroides* sp.*, Fusobacteria* and  *Clostridia, Bifidobacteria* | Rabbit | Sulfatase | Sulfide | Increase efficacy | Reduction  (Sulfoxide reduction) | [191] |
|  |  | - | Human | Sulfatase | - | - | Reduction  (Sulfoxide reduction) | [130] |
| Sumatriptan succinate | Nonsteroidal antiinflammatory drug | Bacteroides fragilis and Bacteroides uniformis | Human | - | - | - | - | [117] |
| Tacrolimus | Immunosuppressant | Akkermansia muciniphila, Alistipes indistinctus, Bacteroides eggerthii, Bacteroides fragilis, Bacteroides uniformis, Bacteroides xylanisolvens, Bifidobacterium adolescentis, Bifidobacterium ruminatum, Blautia hansenii, Blautia luti, Clostridium difficile, Clostridium ramosum, Clostridium sp., Clostridium sporogenes, Eggerthella lenta, Escherichia coli, Eubacterium biforme, Odoribacter splanchnius, Providencia alcalifaciens, Roseburia intestinalis, Ruminococcus gnavus and Victivallis vadensis | Human | - | - | - | Reduction  (Keto reduction) | [117] |
| Tadalafil | Endocrine drug | Bacteroides fragilis and Bacteroides thetaiotaomicron | Human | - | - | - | - | [117] |
| Telmisartan | Endocrine drug | Bacteroides cellulosilyticus, Bacteroides dorei, Bacteroides fragilis, Bacteroides stercoris, Bacteroides thetaiotaomicron, Bacteroides uniformis, Bacteroides vulgatus, Bacteroides xylanisolvens and Parabacteroides distasonis | Human | - | - | - | - | [117] |
| Tenatoprazole | Digestive-related drug | Akkermansia muciniphila, Alistipes indistinctus, Anaerotruncus colihominis, Anaerococcus hydrogenalis, Anaerostipes sp., Bacteroides caccae, Bacteroides cellulosilyticus, Bacteroides coprophilus, Bacteroides dorei, Bacteroides eggerthii, Bacteroides fragilis, Bacteroides finegoldii, Bacteroides intestinalis, Bacteroides ovatus, Bacteroides pectinophilus, Bacteroides stercoris, Bacteroides uniformis, Bacteroides vulgatus, Bacteroides xylanisolvens, Blautia hansenii, Blautia luti, Bifidobacterium adolescentis, Bifidobacterium breve, Bifidobacterium longum, Bifidobacterium ruminatum, Bacteroides thetaiotaomicron, Bryantia formatexigens, Clostridium asparagiforme, Clostridium bolteae, Clostridium difficile, Clostridium hathewayi, Clostridium scindens, Clostridium sp., Clostridium spiroforme, Clostridium sporogenes, Clostridium ramosum, Clostridium symbiosum, Collinsella aerofaciens, Collinsella intestinalis, Coprococcus comes, Dorea formicigenerans, Edwardsiella tarda, Eggerthella lenta, Enterobacter cancerogenus, Enterococcus faecalism, Escherichia coli, Eubacterium biforme, Eubacterium hallii, Eubacterium rectale, Eubacterium ventriosum, Limosilactobacillus reuteri, Odoribacter splanchnius, Parabacteroides distasonis, Parabacteroides johnsonii, Parabacteroides merdae, Pretovella copri, Proteus penneri, Providencia alcalifaciens, Providencia rettgeri, Providencia stuartii, Roseburia intestinalis, Ruminococcus gnavus, Ruminococcus lactaris, Ruminococcus torques, Salmonella Typhimurium, Subdoligranulum variabile and Victivallis vadensis | Human | - | - | - | - | [117] |
| Terbinafine | Antifungal agent | Anaerococcus hydrogenalis, Anaerotruncus colihominis, Bacteroides cellulosilyticus, Bacteroides coprophilus, Bacteroides dorei, Bacteroides intestinalis, Bacteroides pectinophilus, Bacteroides vulgatus, Bifidobacterium breve, Bifidobacterium longum, Blautia hansenii, Clostridium asparagiforme, Clostridium bolteae, Clostridium hathewayi, Clostridium scindens, Clostridium sp., Clostridium spiroforme, Clostridium symbiosum, Collinsella aerofaciens, Coprococcus comes, Dorea formicigenerans, Edwardsiella tarda, Enterobacter cancerogenus, Eubacterium hallii, Eubacterium ventriosum, Odoribacter splanchnius, Parabacteroides johnsonii, Pretovella copri, Proteus penneri, Providencia stuartii, Roseburia intestinalis, Ruminococcus gnavus, Ruminococcus lactaris  and Ruminococcus torques | Human | - | - | - | - | [117] |
| Tinidazole | Antibiotic | Akkermansia muciniphila, Alistipes indistinctus, Anaerotruncus colihominis, Anaerococcus hydrogenalis, Anaerostipes sp., Bacteroides caccae, Bacteroides cellulosilyticus, Bacteroides dorei, Bacteroides eggerthii, Bacteroides fragilis, Bacteroides finegoldii, Bacteroides intestinalis, Bacteroides ovatus, Bacteroides stercoris, Bacteroides uniformis, Bacteroides vulgatus, Bacteroides xylanisolvens, Blautia hansenii, Blautia luti, Bifidobacterium adolescentis, Bifidobacterium breve, Bifidobacterium longum, Bacteroides thetaiotaomicron, Bryantia formatexigens, Clostridium asparagiforme, Clostridium bolteae, Clostridium difficile, Clostridium hathewayi, Clostridium scindens, Clostridium sp., Clostridium spiroforme, Clostridium sporogenes, Clostridium ramosum, Clostridium symbiosum, Collinsella aerofaciens, Collinsella intestinalis, Coprococcus comes, Dorea formicigenerans, Edwardsiella tarda, Enterococcus faecalism, Eubacterium biforme, Eubacterium hallii, Eubacterium rectale, Eubacterium ventriosum, Odoribacter splanchnius, Parabacteroides distasonis, Parabacteroides johnsonii, Parabacteroides merdae, Pretovella copri, Roseburia intestinalis, Ruminococcus gnavus, Ruminococcus lactaris, Ruminococcus torques, Subdoligranulum variabile and  Victivallis vadensis | Human | - | - | - | - | [117] |
| Tolazamide | Endocrine drug | Bacteroides coprophilus, Bacteroides dorei, Bacteroides eggerthii, Bacteroides fragilis, Bacteroides ovatus, Bacteroides stercoris, Bacteroides thetaiotaomicron, Bacteroides uniformis, Bacteroides vulgatus, Blautia hansenii, Clostridium sp., Eubacterium biforme, Odoribacter splanchnius, Parabacteroides distasonis and Parabacteroides johnsonii | Human | - | - | - | - | [117] |
| Tolcapone | Nervous system drug | - | Human | Nitro reductase and Acetyl transferase | - | - | Reduction (Nitro reduction) and Functional group removal (Acetylation) | [130] |
| Trandolapril | Endocrine drug | Bacteroides cellulosilyticus, Bacteroides dorei, Bacteroides fragilis, Bacteroides thetaiotaomicron, Bacteroides vulgatus, Bacteroides xylanisolvens, Eubacterium biforme, Odoribacter splanchnius and Pretovella copri | Human | - | - | - | - | [117] |
| Tranilast | Antihistamine agent | Bacteroides fragilis and Bacteroides vulgatus | Human | - | - | - | - | [117] |
| Trihexyphenidyl | Nervous system drug | Blautia hansenii and Clostridium sp. | Human | - | - | - | - | [117] |
| Trimebutine Maleate | Digestive-related drug | Alistipes indistinctus, Bacteroides dorei, Bifidobacterium ruminatum, Blautia hansenii, Bryantia formatexigens, Clostridium asparagiforme, Clostridium hathewayi, Clostridium sp., Eggerthella lenta, Eubacterium biforme, Eubacterium hallii, Odoribacter splanchnius, Ruminococcus torques, Salmonella Typhimurium and Victivallis vadensis | Human | - | - | - | - | [117] |
| Valsartan | Endocrine drug | Bacteroides dorei, Bacteroides fragilis, Bacteroides thetaiotaomicron, Bacteroides uniformis, Bacteroides vulgatus, Bacteroides xylanisolvens and Parabacteroides distasonis | Human | - | - | - | - | [117] |
| Venlafaxine | Nervous system drug | Bacteroides dorei, Bacteroides vulgatus  and Bacteroides uniformis | Human | - | - | - | - | [117] |
| Vilazodone | Nervous system drug | Bacteroides cellulosilyticus, Bacteroides coprophilus, Bacteroides dorei, Bacteroides eggerthii, Bacteroides fragilis, Bacteroides intestinalis, Bacteroides ovatus, Bacteroides stercoris, Bacteroides thetaiotaomicron, Bacteroides uniformis, Bacteroides vulgatus, Bacteroides xylanisolvens, Clostridium spiroforme, Clostridium sporogenes, Parabacteroides distasonis, Parabacteroides johnsonii, Parabacteroides merdae and Pretovella copri | Human | - | - | - | - | [117] |
| Voriconazole | Antifungal agent | Bacteroides dorei, Bacteroides fragilis, Bacteroides thetaiotaomicron, Bacteroides uniformis, Bacteroides vulgatus and  Bacteroides xylanisolvens | Human | - | - | - | - | [117] |
| Warfarin | Anticoagulant drug | Bacteroides fragilis and Bacteroides vulgatus | Human | - | - | - | - | [117] |
| Zaleplon | Nervous system drug | Bacteroides fragilis,Bacteroides vulgatus  and Bacteroides xylanisolvens | Human | - | - | - | - | [117] |
| Ziprasidone mesilate | Nervous system drug | Bacteroides vulgatus | Human | - | - | - | - | [117] |
| Zonisamide | Nervous system drug | *Clostridium sporogenes, Bifidobacterium bifidum, Bacteroides vulgatus, Escherichia coli, Salmonella typhimurium, Pseudomom jluorescens, Lactobacillus rharnnosus* and  *Streptococcus faecalis* | Mammal | Benzisoxazole ring reductases | 2-Sulfamoylacetyl phenol | Decrease efficacy | Reduction  (Benzisoxazole ring reduction) | [195] |
| Butylparaben | Personal care products | - | Human | - | p-Hydroxybenzoic acid | Decrease toxicity | Hydrolysis (Dealkylation) | [196] |
| Triclocarban-glucopyranoside (TCC-Glu) and Triclocarban-sulfate  (TCC-Sulfate) | Personal care products | - | Human and rat | β-Glucuronidase and sulfatase | Hydroxylated trichlorocarbon (OH-TCC) | Increase bioavailability | Hydrolysis (Deconjugation) | [197] |
| Triclosan | Personal care products | - | Fish (Larval zebrafish) | - | Triclosan sulfate | - | Functional group removal (Sulfonation; Not distinguished the role of hosts and gut microbiota) | [198] |

**Table S4.** Summary of transformation of mycotoxins by the gut microbiota human and fauna.

| **Name** | **Functional microbes** | **Hosts** | **Enzymes** | **Metabolites** | **Potential**  **consequences** | **Mechanisms** | **References** |
| --- | --- | --- | --- | --- | --- | --- | --- |
| Deoxynivalenol (DON) | - | Human | Deoxygenase | Deepoxy-deoxynivalenol | Decrease toxicity | Reduction (De-epoxidation) | [199] |
| Deoxynivalenol-3-β-glucoside (DON-Glu) | *Enterococcus durans, Enterococcus mundtii, Lactiplantibacillus plantarum,*  *Butyrivibrio fibrisolvens, Roseburia intestinalis, Eubacterium rectale, Bifidobacterium adolescentis* and *Lactiplantibacillus plantarum* | Human | β-Glucuronidase | Deoxynivalenol | Increase bioavailability | Hydrolysis (Deconjugation) | [199-203] |
| Diacetoxyscirpenol (DAS) | *Prevotella copri* and *Bifidobacterium fibrisolvens* | Human | - | Deoxynivalenol | Increase bioavailability | Hydrolysis (Deacylation) | [201] |
| HT-2-β-glucoside (HT-2-Glu) | *Butyrivibrio fibrisolvens, Roseburia intestinalis* and *Eubacterium rectale* | Human | β-Glucuronidase | Deoxynivalenol | Increase bioavailability | Hydrolysis (Deconjugation) | [201] |
| Nivalenol-3-β-glucoside (NIV-Glu) | *Butyrivibrio fibrisolvens, Roseburia intestinalis* and *Eubacterium rectale* | Human | β-Glucuronidase | Deoxynivalenol | Increase bioavailability | Hydrolysis (Deconjugation) | [201, 202] |
| Ochratoxin A (OTA) | - | Rat | Deamidase | Ochratoxin α and phenylalanine | Decrease toxicity | Hydrolysis (Deamidation) | [204] |
| T-2 toxin | *Prevotella copri* and *Bifidobacterium fibrisolvens* | Human | - | Deoxynivalenol | Increase bioavailability | Hydrolysis (Deacylation) | [201, 202] |
| Zearalenone-β-glucoside (ZEN-Glu) | - | Human | β-Glucuronidase | Zearalenone | Increase bioavailability | Hydrolysis (Deconjugation) | [203] |
| 15-Acetyldeoxynivalenol (15-ADON) | - | Chicken | - | Deoxynivalenol | Increase bioavailability | Hydrolysis (Deacylation) | [205] |
| 3-Acetyl-deoxynivalenol (3-ADON) | - | Pig | - | Deoxynivalenol | Increase bioavailability | Hydrolysis (Deacylation) | [206] |
| Aflatoxin B1 | *Bacillus subtilis* | Fish | - | - | Decrease toxicity | - | [207] |
|  | *Escherichia coli* | Chicken | - | - | Decrease toxicity | - | [208] |
| Deoxynivalenol (DON) | *Anaerofilum, Coriobacterium* sp. and *Bacillus arbutinivorans* | Chicken | Deoxygenase | Deepoxy-deoxynivalenol | Decrease toxicity | Reduction (De-epoxidation) | [205, 209] |
|  | - | Fish | Deoxygenase | Deepoxy-deoxynivalenol | Decrease toxicity | Reduction (De-epoxidation) | [210] |
|  | - | Pig | Deoxygenase | Deepoxy-deoxynivalenol | Decrease toxicity | Reduction (De-epoxidation) | [211] |
| Diacetylated trichothecenes diacetoxyscirpenol | - | Chicken | - | Deoxynivalenol | Increase bioavailability | Hydrolysis (Deacylation) | [205] |
| Fusarenon X (FX) | - | Chicken | - | Deepoxy-fusarenon | Increase bioavailability | Hydrolysis (Deacylation) | [205] |
| HT-2 | - | Chicken | Deoxygenase | - | Increase bioavailability | Reduction (De-epoxidation) | [205] |
| HT-2-β-glucoside (HT-2-Glu) | - | Pig | β-Glucuronidase | Deoxynivalenol | Increase bioavailability | Hydrolysis (Deconjugation) | [212] |
| Monoacetyl trichothecenes 3-acetyldeoxynivale-nol | - | Chicken | - | - | - | Hydrolysis (Deacylation) | [205] |
| Nivalenol (NIV) | - | Pig | Deoxygenase | Deepoxy-nivalenol | - | Reduction (De-epoxidation) | [206] |
| Non-acylated trichothecenes 4-deoxynivalenol | - | Chicken | Deoxygenase | - | - | Reduction (De-epoxidation) | [205] |
| Ochratoxin A (OTA) | - | Cow | Deamidase | Ochratoxin α and phenylalanine | Decrease toxicity | Hydrolysis (Deamidation) | [213, 214] |
| T-2 toxin | - | Chicken | - | Neosolaniol | - | Hydrolysis (Deacylation) | [205] |
| T-2-β-glucoside (T-2-Glu) | - | Pig | β-Glucuronidase | Deoxynivalenol | Increase bioavailability | Hydrolysis (Deconjugation) | [212] |
| Verrucarol | - | Chicken | Deoxygenase | Deepoxy-verrucarol | - | Reduction (De-epoxidation) | [205] |
| Zearalenone (ZEN) | - | Pig | - | Zearalenol | Increase toxicity | Reduction (Keto-reduction) | [211] |
|  | *Pseudomonas otitidis* | Cow | - | - | - | - | [215] |
|  | *Lysinibacillus* sp. | Chicken | - | - | - | - | [216] |

**Table S5.** Summary of transformation of plastic additives by gut microbiota from human and fauna.

| **Environmental pollutants** | **Microbiota** | **Hosts** | **Enzymes** | **Metabolites** | **Potential consequences** | **Mechanisms** | **References** |
| --- | --- | --- | --- | --- | --- | --- | --- |
| Tetrabromobisphenol A (TBBPA) | *Clostridium manihotivorum* | Human | Rhamnosyltransferase (CmRT) | Rhamnosylated TBBPA (TBBPA-Rha) and TBBPA-DiRha·HCOOH | Decrease bioavailability and toxicity | Functional group removal (Glycosylation) | [217] |
| Tetrabromobisphenol A mono-β-D-glucopyranoside (TBBPA-G) | - | Human | β-Glucuronidase | Tetrabromobisphenol A (TBBPA) | Increase bioavailability | Hydrolysis (Deconjugation) | [218] |
| Tetrabromobisphenol S (TBBPS) | *Clostridium manihotivorum* | Human | Rhamnosyltransferase (CmRT) | Rhamnosylated TBBPS (TBBPS-Rha) and TrBPS-Rha | Decrease bioavailability and toxicity | Functional group removal (Glycosylation) | [217] |
| Tetrachlorobisphenol A (TCBPA) | *Clostridium manihotivorum* | Human | Rhamnosyltransferase (CmRT) | Rhamnosylated TCBPS (TCBPS-Rha) and TCBPA-DiRha·HCOOH | Decrease bioavailability and toxicity | Functional group removal (Glycosylation) | [217] |
| TBBPA mono (allyl ether) (TBBPA-MAE) | *Clostridium manihotivorum* | Human | Rhamnosyltransferase (CmRT) | TBBPA, TBBPA-Rha·HCOOH and TrBBPA-MAE | Decrease bioavailability and toxicity | Reduction (Dehalogenation) and Functional group removal (Glycosylation) | [217] |
| TBBPA mono (2,3-dibromopropyl ether) (TBBPA-MDBPE) | *Clostridium manihotivorum* | Human | Rhamnosyltransferase (CmRT) | TBBPA-MAE, TBBPA, TBBPA-Rha·HCOOH and TrBBPA-MAE | Decrease bioavailability and toxicity | Hydrolysis (Dealkylation), Reduction (Dehalogenation), and Functional group removal (Glycosylation) | [217] |
| Chloroacetonitrile (CAN) | *-* | Human | - | - | Decrease toxicity | - | [219] |
| Dibromoacetic acid (DBAA) | *-* | Human | - | - | Decrease toxicity | - | [219] |
| Tetrabromopyrrole (FBPy) | *-* | Human | - | - | Decrease toxicity | - | [219] |
| Bisphenol A | *Microbacterium* and *Alcaligenes* | Human | - | - | Increase estrogenicity | - | [220] |
| Bisphenol A | *Citrobacter* and *Klebsiella* | Fish (*Carassius auratus* and *Epinephelus coioides*) | - | Hydroquinone and  4-hydroxybenzaldehyde | Decrease estrogenicity | Oxidation (Alkyl cleavage) | [221, 222] |
| Bisphenol A β-glucopyranoside (BPA-G) | *-* | Fish (*Epinephelus coioides*) | β-Glucuronidase | BPA | Increase bioavailability | Hydrolysis (Deconjugation) | [222] |
|  | *-* | Mussel (*Perna viridis*) | β-Glucuronidase | BPA | Increase bioavailability | Hydrolysis (Deconjugation) | [222] |
|  | *-* | Prawn (*Penaeus monodon*) | β-Glucuronidase | BPA | Increase bioavailability | Hydrolysis (Deconjugation) | [222] |
| Bisphenol AP | *Citrobacter* and *Klebsiella* | Fish (*Carassius auratus*) | - | Hydroquinone and  4-hydroxybenzaldehyde | Increase estrogenicity | Oxidation (Alkyl cleavage) | [221] |
| Bisphenol F | *Citrobacter* and *Klebsiella* | Fish (*Carassius auratus*) | - | 4-Hydroxybenzaldehyde | Decrease estrogenicity | Oxidation (Alkyl cleavage) | [221] |
| Di(2-ethylhexyl) phthalate | Clostridiaceae, Oceanobacillus, Acidobacteria, Serratia marcescens and Acinetobacter | Earthworms | - | - | - | - | [223] |
| Phenol | *Acinetobacter t*and*oii* | Termite | - | Catechol, *cis*-*cis* muconic acid, acetyl-CoA and succinate | - | Oxidation (Hydroxylation and alkyl cleavage) | [224] |
| Tri(2-butoxyethyl) phosphate | Rhodococcus, Flavobacterium and Pseudomonas | Earthworms | - | - | - | - | [225] |
| Triphenyl phosphate | *Citrobacter* and *Klebsiella* | Fish (*Carassius auratus*) | - | Diphenyl phosphate | Decrease estrogenicity | Hydrolysis (Dealkylation) | [221] |

**Table S6.** Summary of transformation of emerging organic pollutants by gut microbiota from fauna.

| **Environmental pollutants** | **Microbiota** | **Hosts** | **Enzymes** | **Metabolites** | **Potential consequences** | **Mechanisms** | **References** |
| --- | --- | --- | --- | --- | --- | --- | --- |
| Graphene oxide | - | Rat | - | Organic butyrate | Decrease toxicity | Oxidation (oxidative depolymerization) | [226] |
| Polyethylene  (289 kDa of molecular weight) | *Acinetobacter* sp. and *Bacillus* sp.*, Serratia* sp. | Larvae of greater wax moth (*Galleria mellonella*) | Alcohol dehydrogenase | - | - | Oxidation (oxidative depolymerization) | [227-230] |
|  | - | Superworm (*Zophobas atratus* larvae) | - | - | - | Oxidation (oxidative depolymerization) | [229, 231] |
| Polypropylene (5–6 cm in diameter) | - | Mealworm (*Tenebrio molitor*) | - | - | - | Oxidation (oxidative depolymerization) | [232] |
|  | *Enterobacter asburiae* YT1 and *Bacillus* sp. YP1 | Mealworm (Larvae of *Plodia interpunctella*) | - | - | - | Oxidation (oxidative depolymerization) | [233, 234] |
|  | - | Superworm (*Zophobas atratus*) | - | - | - | Oxidation (oxidative depolymerization) | [232] |
| Low-density polyethylene  (<150 μm) | Microbacterium awajiense, Rhodococcus jostii, Mycobacterium vanbaalenii, Streptomyces fulvissimus, Bacillus simplex and Bacillus sp. | Earthworm (*Lumbricus terrestris*) | - | Octadecane, eicosane, docosane and tricosane | - | Oxidation (oxidative depolymerization) | [235] |
| Polystyrene (2–3 cm in diameter) | *Citrobacter* sp.*, Kosakonia* sp.*, Bacillus* sp. and  *Serratia* sp. | Mealworm (Larvae of *Tenebrio molitor*) | - | - | - | Oxidation (oxidative depolymerization) | [230, 236] |
|  | - | Superworm (*Zophobas atratus* larvae) | Reactive oxygen species and laccase | Soluble monomers | - | Oxidation (oxidative depolymerization) | [237] |
| Polystyrene (3 mm in diameter) | *Exiguobacteria* sp. | Mealworm (Larvae of *Tenebrio molitor*) | - | Debris and CO_2_ | - | Oxidation (oxidative depolymerization) and mineralization | [229, 236, 238] |
| Polystyrene  (6.7-1346kDa of molecular weight) | *Pseudomonas aeruginosa, Citrobacter freundii, Serratia marcescens* and  *Klebsiella aerogenes* | Superworm (*Zophobas atratus* larvae) | Serine hydrolase | Debris and CO_2_ | - | Oxidation (oxidative depolymerization) and mineralization | [229, 231, 239-242] |
| Polystyrene (film) | *Bacillus anthracis Ames* PSI-1 and *Enterobacter cloacae* PSI-2 | Mealworm (Larvae of *Tenebrio molitor Linnaeus*) | - | - | - | - | [243] |
| Polyvinyl chloride (film) | *Klebsiella* sp.*,* | Larvae of *Spodoptera frugiperda* | Atalase peroxidase, dehalogenases, enolase, aldehyde dehydrogenase and oxygenase | - | - | Oxidation (oxidative depolymerization) | [244] |
| Polylactic acid | *-* | Rat | - | Organic butyrate | Decrease toxicity | Oxidation (oxidative depolymerization) | [245] |
| Single-walled carbon nanotubes | - | Rat | - | Organic butyrate | Decrease toxicity | Oxidation (oxidative depolymerization) | [226] |

**Table S7.** Composition of the clusters obtained by the multidimensional scaling (MDS) analysis using online platform ChemMine Tools.

| **Cluster 1** | **Cluster 2** | **Cluster 3** | **Cluster 4** | **Cluster 5** | **Cluster 6** | **Cluster 7** | **Cluster 8** |
| --- | --- | --- | --- | --- | --- | --- | --- |
| Naphthalene | Deoxynivalenol | 2,4,5-Trichlorobiphenyl | Phenol | 2-Amino-3-methylimidazo(4,5-f) quinoline | 5-Aminosalicylate | 8-Prenylnaringenin | Neoprontosil |
| Phenanthrene | Nivalenol | Tetrachlorobiphenyl | 4-Nitrobenzoic acid | 3,4-Dimethoxyacetanilide | 5-Fluorocytosine | Acetanilide | Acid yellow |
| Azobenzene | 3-Acetyl-deoxynivalenol | Hexachlorobiphenyl | Nitrobenzene | 3,4-Dimethoxycinnamic acid | 5-Fluorouracil | Acetaminophen | Amaranth |
| Benzo[a]pyrene | Verrucarol |  | 1,3-Dinitrobenzene | Dihydrosinapic acid |  | Phenacetin | Orange G |
| Clonazepam | 15-Acetyldeoxynivalenol |  | 2,4-Dinitrotoluene | 3,5-Dimethoxyphenylpropionic acid |  | Formanilide | Para Red |
| Nitrazepam |  |  | 2,4,6-Trinitrotoluene |  |  | Bucetin | Allura Red AC |
| N-hydroxy-N-2-fluorenylacetamide |  |  |  |  |  | p-Ethoxyglycolanilide | Ponceau SX |
| Bisphenol A |  |  |  |  |  | Acetaminophen-Glu | Orange II |
| 2-Nitrofluorene |  |  |  |  |  | Acetamiprid | Buffalo Black NBR |
| Bisphenol F |  |  |  |  |  |  |  |
| 1-Nitropyrene |  |  |  |  |  |  |  |
| 6-Nitrochrysene |  |  |  |  |  |  |  |
| Pyrene |  |  |  |  |  |  |  |
| 6-Nitrobenzo[a]pyrene |  |  |  |  |  |  |  |
| 3-Nitrobenzo(a)pyrene |  |  |  |  |  |  |  |
| 1,3-Dinitropyrene |  |  |  |  |  |  |  |
| Bisphenol AP |  |  |  |  |  |  |  |
| Tolcapone |  |  |  |  |  |  |  |
| **Cluster 9** | **Cluster 10** | **Cluster 11** | **Cluster 12** | **Cluster 13** | **Cluster 14** (The sum of other 50 clusters) | | |
| Ketoprofen-Glu | Sulfasalazine | Butylparaben | Trypan blue | Morphine-Glu | Diltiazem | Methotrexate | Omeprazole |
| Diclofenac-Glu | Balsalazide | Capecitabine | Cibacron Brilliant Red 3B-A | Deoxynivalenol-Glu | Eltrombopag | Methoxychlor | Propachlor |
| Indomethacin-Glu | Ipsalazide | Carbetapentane citrate | Congo red | HT-2-β-glucoside | Endosulfan | Methyl red | Sennoside |
|  | Benzylpenicillin | Chloramphenicol |  | T-2-Glu | Famciclovir | Prontosil | SN38-G |
|  | Berberine | Chlorpyrifos |  |  | Hydrocortisone | Methyl orange | Succinyl sulfathiazole |
|  | Brivudine |  |  |  | Prednisone | Metronidazole | Tacrolimus |
|  |  |  |  |  | Irinotecan | Misonidazole | Yellow 2G |
|  |  |  |  |  | Janus green | Misoprostol | Tartrazine |
|  |  |  |  |  | Ketorolac tromethamine | Mycophenolate mofetil | Tetrabromobisphenol A-Glu |
|  |  |  |  |  | Levamisole | Nicardipine | Trichlorfon |
|  |  |  |  |  | Levodopa | Nicotine-1'-N-oxide | Triphenyl phosphate |
|  |  |  |  |  | Lindane | Nitrofurantoin | Zearalenone |
|  |  |  |  |  | Loperamide N-oxide | Nizatidine | ZEN-Glu |
|  |  |  |  |  | Lovastatin | Norethindrone Acetate | Zonisamide |
|  |  |  |  |  | Simvastatin | Ochratoxin A | Azetirelin |
|  |  |  |  |  | Methamphetamine | Olanzapine |  |

**Table S8.** Summary of xenobiotic-metabolizing enzymes those produced by gut microbes from human and fauna.

| **Group** | **Host** | **Position** | **Enzyme** | **Taxonomic information** | | **References** |
| --- | --- | --- | --- | --- | --- | --- |
|  |  |  |  | **Species** | **Phylum** |  |
| Unknown host | Unknown host | - | Azo reductase | *Bifidobacterium infantis* | *Actinomycetota* | [53] |
|  |  |  | Azo reductase | *Acidaminococcus fermentans* | *Bacillota* | [53] |
|  |  |  | Azo reductase | *Coprococcus catus* | *Bacillota* | [53] |
|  |  |  | Azo reductase | *Eubacterium biforme* | *Bacillota* | [53] |
|  |  |  | Azo reductase | *Peptostreptococcus productus* | *Bacillota* | [53] |
|  |  |  | Azo reductase | *Bacteroides* *thetaitaomcron* | *Bacteroidota* | [53] |
|  |  |  | Azo reductase | *Citrobacter* sp. | *Pseudomonadota* | [53] |
|  |  |  | Azo reductase | *Fusobacterium* sp. | *Pseudomonadota* | [53] |
|  |  |  | Azo reductase | *Bifidobacterium* *adolescentis* | *Actinomycetota* | [64] |
|  |  |  | Azo reductase | *Acidaminococcus* *fermentans* | *Bacillota* | [64] |
|  |  |  | Azo reductase | *Clostridium* *paraputrificum, Clostridium* *ramosum* and *Clostridium* *sporogenes* | *Bacillota* | [64] |
|  |  |  | Azo reductase | *Eubacterium* *aerofaciens* | *Bacillota* | [64] |
|  |  |  | Azo reductase | *Lactobacillus* *catenaforme* | *Bacillota* | [64] |
|  |  |  | Azo reductase | *Peptococcus* *prevotii* | *Bacillota* | [64] |
|  |  |  | Azo reductase | *Ruminococcus* *bromii* | *Bacillota* | [64] |
|  |  |  | Azo reductase | *Streptococcus* *faecium* | *Bacillota* | [64] |
|  |  |  | Azo reductase | *Veillonella* *parvula* | *Bacillota* | [64] |
|  |  |  | Azo reductase | *Klebsiella pneumoniae* | *Pseudomonadota* | [64] |
|  |  |  | Azo reductase | *Proteus vulgaris* | *Pseudomonadota* | [64] |
|  |  |  | Azo reductase | *Pseudomonas aeruginosa* | *Pseudomonadota* | [64] |
|  |  |  | Azo reductase | *Salmonella typhimurium* | *Pseudomonadota* | [64] |
|  |  |  | Azo reductase | *Bacillus* sp. | *Bacillota* | [246] |
|  |  |  | Azo reductase | *Streptococcus faecalis* | *Bacillota* | [246] |
|  |  |  | Azo reductase | *Bacteroides* sp. | *Bacteroidota* | [246] |
|  |  |  | Azo reductase | *Lactobacillus* sp. | *Bacillota* | [246] |
|  |  |  | Azo reductase | *Escherichia coli* | *Pseudomonadota* | [246] |
|  |  |  | Azo reductase | *Pseudomonas aeruginosa* | *Pseudomonadota* | [246] |
|  |  |  | Azo reductase | *Proteus vulgaris* | *Pseudomonadota* | [246] |
|  |  |  | Azo reductase | *Staphylococcus aureus* | *Bacillota* | [190] |
|  |  |  | Azo reductase | *Streptococcus faecalis* | *Bacillota* | [190] |
|  |  |  | Azo reductase | *Bacteroides* sp. | *Bacteroidota* | [190] |
|  |  |  | Azo reductase | *Pseudomonas pyrocyanea* | *Pseudomonadota* | [190] |
|  |  |  | Azo reductase | *Escherichia coli* | *Pseudomonadota* | [190] |
|  |  |  | Azo reductase | *Klebsiella aerogenes* | *Pseudomonadota* | [190] |
|  |  |  | Azo reductase | *Proteus* *vulgaris* | *Pseudomonadota* | [190] |
|  |  |  | Azo reductase | *Clostridium perfringens* | *Bacillota* | [61] |
|  |  |  | Azo reductase | *Xenophilus azovorans* | *Pseudomonadota* | [247] |
|  |  |  | Azo reductase | *Geobacillus stearothermophilus* | *Bacillota* | [248] |
|  |  |  | CYP450 | *Nocardia farcinica* | *Actinomycetota* | [249] |
|  |  |  | CYP450 | *Bacillus* *megaterium* | *Bacillota* | [250] |
|  |  |  | Monoamine oxidase | *Klebsiella* *pneumoniae* | *Pseudomonadota* | [251] |
|  |  |  | Monoamine oxidase | *Enterobacter* *aerogenes* | *Pseudomonadota* | [251] |
|  |  |  | Monoamine oxidase | *Escherichia coli* | *Pseudomonadota* | [251] |
|  |  |  | Monoamine oxidase | *Salmonella* *typhimurium* | *Pseudomonadota* | [251] |
|  |  |  | Monoamine oxidase | *Serratia* *marcescens* | *Pseudomonadota* | [251] |
|  |  |  | Monoamine oxidase | *Proteus* *inconstans* | *Pseudomonadota* | [251] |
|  |  |  | Monoamine oxidase | *Pseudomonas aeruginosa* | *Pseudomonadota* | [251] |
|  |  |  | Monoamine oxidase | *Micrococcus luteus* | *Actinomycetota* | [251] |
|  |  |  | Monoamine oxidase | *Brevibacterium ammoniagenes* | *Actinomycetota* | [251] |
|  |  |  | N-acetyl transferase | *Citrobacter amalonaticus* and *Citrobacter farmer,*  *Citrobacter freundii* and *Citrobacter koseri* | *Pseudomonadota* | [109] |
|  |  |  | N-acetyl transferase | *Klebsiella oxytoca* and *Klebsiella rhinoscleromatis* | *Pseudomonadota* | [109] |
|  |  |  | N-acetyl transferase | *Morganella morganii* | *Pseudomonadota* | [109] |
|  |  |  | N-acetyl transferase | *Serratia marcescens* | *Pseudomonadota* | [109] |
|  |  |  | N-acetyl transferase | *Escherichia coli* | *Pseudomonadota* | [109] |
|  |  |  | N-acetyl transferase | *Salmonella enterica* | *Pseudomonadota* | [109] |
|  |  |  | N-acetyl transferase | *Pseudomonas aeruginosa* | *Pseudomonadota* | [109] |
|  |  |  | N-acetyl transferase | *Helicobacter pylori* | *Pseudomonadota* | [109] |
|  |  |  | N-acetyl transferase | *Bacteroides* sp. | *Bacteroidota* | [109] |
|  |  |  | Nitro reductase | *Escherichia coli* | *Pseudomonadota* | [252] |
|  |  |  | Nitro reductase | *Bacteroides fragilis* | *Bacteroidota* | [33] |
|  |  |  | Nitro reductase | *Escherichia coli* | *Pseudomonadota* | [253, 254] |
|  |  |  | Nitro reductase | *Salmonella typhimurium* | *Pseudomonadota* | [255] |
|  |  |  | Nitro reductase | *Salmonella typhimurium* | *Pseudomonadota* | [256] |
|  |  |  | Nitro reductase | *Enterobacter cloacae* | *Pseudomonadota* | [257, 258] |
|  |  |  | Nitro reductase | *Klebsiella* sp. | *Pseudomonadota* | [38] |
|  |  |  | Nitro reductase | *Rhodobacter* | *Pseudomonadota* | [259] |
|  |  |  | β-Glucuronidases | *Clostridium* *perfringens* | *Bacillota* | [260] |
|  |  |  | β-Glucuronidases | *Streptococcus* *agalactiae* | *Bacillota* | [260] |
|  |  |  | β-Glucuronidases | *Escherichia coli* | *Pseudomonadota* | [261] |
|  |  |  | β-Glucuronidases | *Eubacterium eligens* | *Bacillota* | [261] |
|  |  |  | β-Glucuronidases | *Streptococcus* *agalactiae* | *Bacillota* | [261] |
|  |  |  | β-Glucuronidases | *Clostridium perfringens* | *Bacillota* | [261] |
|  |  |  | β-Glucuronidases | *Faecalibacterium prausnitzii* | *Bacillota* | [261] |
|  |  |  | β-Glucuronidases | *Lactobacillus rhamnosus* | *Bacillota* | [261] |
|  |  |  | β-Glucuronidases | *Ruminococcus gnavus* | *Bacillota* | [261] |
|  |  |  | β-Glucuronidases | *Bacteroides fragilis* | *Bacteroidota* | [261] |
|  |  |  | β-Glucuronidases | *Bacteroides uniformis* | *Bacteroidota* | [261] |
|  |  |  | β-Glucuronidases | *Parabacteroides merdae* | *Bacteroidota* | [261] |
|  |  |  | β-Glucuronidases | Bacteroides ovatus | *Bacteroidota* | [261] |
| Invertebrates | Worm | The whole gut | Dehydrochlorinase | *Rhodococcus* sp. | *Actinomycetota* | [262] |
|  |  |  | Dehydrochlorinase | *Bacillus* sp. | *Bacillota* | [262] |
| Fish | Freshwater fish  (Angelfish and oscar) | The whole gut | Esterase | *Clostridium subterminale* and *Clostridium sporogenes* | *Bacillota* | [263] |
|  |  |  | Esterase | *Fusobacterium* sp. | *Pseudomonadota* | [263] |
|  |  |  | Esterase and β-Glucoronidase | *Porphyromonas* sp. | *Bacteroidota* | [263] |
|  |  |  | Esterase and β-Glucoronidase | *Bacteroides distasonis, Bacteroides thetaiotamicron* and *Bacteroides fragilis* | *Bacteroidota* | [263] |
| Non-human mammals | Rat | Intestine | Azo reductase | *Streptococcus* *faecalis* | *Bacillota* | [52] |
|  |  | Intestine | Azo reductase | *Proteus* sp. | *Pseudomonadota* | [58, 59] |
|  |  |  | Azo reductase | *Streptococcus* sp. | *Bacillota* | [58, 59] |
|  |  | Feces | Nitro reductase | *Clostridium perfringens* | *Bacillota* | [158] |
|  |  | Intestine | Nitro reductase | *Escherichia* *coli* | *Pseudomonadota* | [133] |
|  |  | Feces | Nitro reductase | Bacteroides thetaiotaomicron and Bacteroides ovatus | *Bacteroidota* | [127] |
|  |  | Cecal and large intestine | Phosphohydrolase | *Bacteroides vulgatus* | *Bacteroidota* | [186] |
|  |  |  | Phosphohydrolase | *Bacteroides thetaiotaomicron* | *Bacteroidota* | [186] |
|  |  |  | Phosphohydrolase | *Bacteroides fragilis, Bacteroides uniformis* and *Bacteroides eggerthii* | *Bacteroidota* | [186] |
|  |  | - | Sulfotransferase | *Eubacterium rectale* | *Bacillota* | [264] |
|  |  | Feces | β-Glucoronidase | *Eubacterium ramulus* | *Bacillota* | [177] |
|  |  |  | β-Glucoronidase | *Enterococcus casseliflavus* | *Bacillota* | [177] |
|  |  | - | β-Glucoronidase | *Enterococcus* sp. | *Bacillota* | [265] |
|  |  |  | β-Glucoronidase | Bacillus sp. | *Bacillota* | [265] |
|  |  | - | β-Glucoronidase | *Escherichia* *coli* | *Pseudomonadota* | [137] |
|  |  | - | Sulfatase | *Escherichia* *coli* | *Pseudomonadota* | [67] |
|  |  | - | Sulfatase | *Klebsiella* sp. and *Psadomonas* sp. | *Pseudomonadota* | [191, 192] |
|  |  |  | Sulfatase | *Bacteroides* sp. | *Bacteroidota* | [191, 192] |
|  | Rabbit | - | Sulfatase | *Klebsiella* sp. and *Psadomonas* sp. | *Pseudomonadota* | [191, 192] |
|  |  |  | Sulfatase | *Bacteroides* sp. | *Bacteroidota* | [191, 192] |
| Human |  |  | Alcohol dehydrogenase, Glutathione-S-transferase, Monoamine oxidase, N-acetyl transferase, Thiopurine methyltransferase and CYP450* | *Acholeplasma laidlawii* | *Mycoplasmatota* | [266] |
|  |  |  | Alcohol dehydrogenase, Glutathione-S-transferase, Thiopurine methyltransferase and CYP450* | *Providencia stuartii* | *Pseudomonadota* | [266] |
|  |  |  | Aldehyde dehydrogenase, Alcohol dehydrogenase, Epoxide hydrolase, Monoamine oxidase, Glutathione-S-transferase, Thiopurine methyltransferase, N-acetyl transferase and CYP450* | *Achromobacter arsenitoxydans, Achromobacter piechaudii, Achromobacter* sp. and *Achromobacter xylosoxidans* | *Pseudomonadota* | [266] |
|  |  |  | Aldehyde dehydrogenase, Alcohol dehydrogenase, Epoxide hydrolase, Glutathione-S-transferase, Thiopurine methyltransferase, N-acetyl transferase and CYP450* | *Acidaminococcus fermentans* | *Bacillota* | [266] |
|  |  |  | Aldehyde dehydrogenase, Alcohol dehydrogenase, Epoxide hydrolase, Monoamine oxidase, Glutathione-S-transferase, Thiopurine methyltransferase, N-acetyl transferase and CYP450* | *Acidiphilium cryptum, Acidiphilium multivorum* and *Acidiphilium* sp. | *Pseudomonadota* | [266] |
|  |  |  | Aldehyde dehydrogenase, Alcohol dehydrogenase, Epoxide hydrolase, Monoamine oxidase, Glutathione-S-transferase, Thiopurine methyltransferase, N-acetyl transferase and CYP450* | *Acidobacterium capsulatum* | *Acidobacteriota* | [266] |
|  |  |  | Aldehyde dehydrogenase, Alcohol dehydrogenase, Epoxide hydrolase, Monoamine oxidase, Glutathione-S-transferase, Thiopurine methyltransferase, N-acetyl transferase and CYP450* | *Acidothermus cellulolyticus* | *Actinomycetota* | [266] |
|  |  |  | Aldehyde dehydrogenase, Alcohol dehydrogenase, Epoxide hydrolase, Monoamine oxidase, Glutathione-S-transferase, Thiopurine methyltransferase, N-acetyl transferase and CYP450* | *Acidovorax avenae, Acidovorax citrulli, Acidovorax delafieldii, Acidovorax ebreus,* and *Acidovorax* sp. | *Pseudomonadota* | [266] |
|  |  |  | Aldehyde dehydrogenase, Alcohol dehydrogenase, Epoxide hydrolase, Monoamine oxidase, Glutathione-S-transferase, Thiopurine methyltransferase, N-acetyl transferase and CYP450* | *Acinetobacter calcoaceticus, Acinetobacter nosocomialis, Acinetobacter oleivorans, Acinetobacter pittii* and *Acinetobacter* sp. | *Pseudomonadota* | [266] |
|  |  |  | Aldehyde dehydrogenase, Alcohol dehydrogenase, Epoxide hydrolase, Monoamine oxidase, Glutathione-S-transferase, Thiopurine methyltransferase, N-acetyl transferase and CYP450* | *Actinosynnema mirum* | *Actinomycetota* | [266] |
|  |  |  | Aldehyde dehydrogenase, Alcohol dehydrogenase, Epoxide hydrolase, Monoamine oxidase, Glutathione-S-transferase, Thiopurine methyltransferase, N-acetyl transferase and CYP450* | *Aeromonas aquariorum,* *Aeromonas caviae,* *Aeromonas hydrophila, Aeromonas media, Aeromonas salmonicida, Aeromonas* sp. and *Aeromonas veronii* | *Pseudomonadota* | [266] |
|  |  |  | Aldehyde dehydrogenase, Alcohol dehydrogenase, Epoxide hydrolase, Monoamine oxidase, Glutathione-S-transferase, Thiopurine methyltransferase, N-acetyl transferase and CYP450* | *Afipia* | *Pseudomonadota* | [266] |
|  |  |  | Aldehyde dehydrogenase, Alcohol dehydrogenase, Epoxide hydrolase, Glutathione-S-transferase, Thiopurine methyltransferase, N-acetyl transferase and CYP450* | *Aggregatibacter* sp. and *Aggregatibacter aphrophilus* | *Pseudomonadota* | [266] |
|  |  |  | Aldehyde dehydrogenase, Alcohol dehydrogenase, Epoxide hydrolase, Monoamine oxidase, Glutathione-S-transferase, Thiopurine methyltransferase, N-acetyl transferase and CYP450* | *Agrobacterium fabrum, Agrobacterium radiobacter, Agrobacterium* sp.*, Agrobacterium tumefaciens* and *Agrobacterium vitis* | *Pseudomonadota* | [266] |
|  |  |  | Aldehyde dehydrogenase, Alcohol dehydrogenase, Epoxide hydrolase, Monoamine oxidase, Glutathione-S-transferase, Thiopurine methyltransferase, N-acetyl transferase and CYP450* | *Alcanivorax borkumensis* and *Alcanivorax* sp. | *Pseudomonadota* | [266] |
|  |  |  | Aldehyde dehydrogenase, Alcohol dehydrogenase, Epoxide hydrolase, Monoamine oxidase, Glutathione-S-transferase, Thiopurine methyltransferase, N-acetyl transferase and CYP450* | *Amycolatopsis mediterranei* | *Actinomycetota* | [266] |
|  |  |  | Aldehyde dehydrogenase, Alcohol dehydrogenase, Epoxide hydrolase, Monoamine oxidase, Glutathione-S-transferase, Thiopurine methyltransferase, N-acetyl transferase and CYP450* | *Arcobacter butzleri* and *Arcobacter nitrofigilis* | *Pseudomonadota* | [266] |
|  |  |  | Aldehyde dehydrogenase, Alcohol dehydrogenase, Epoxide hydrolase, Monoamine oxidase, Glutathione-S-transferase, Thiopurine methyltransferase, N-acetyl transferase and CYP450* | *Arthrobacter arilaitensis,* *Arthrobacter chlorophenolicus,* *Arthrobacter phenanthrenivorans* and *Arthrobacter* sp. | *Actinomycetota* | [266] |
|  |  |  | Aldehyde dehydrogenase, Alcohol dehydrogenase, Epoxide hydrolase, Monoamine oxidase, Glutathione-S-transferase, Thiopurine methyltransferase, N-acetyl transferase and CYP450* | *Azospirillum lipoferum* and *Azospirillum* sp. | *Pseudomonadota* | [266] |
|  |  |  | Aldehyde dehydrogenase, Alcohol dehydrogenase, Epoxide hydrolase, Monoamine oxidase, Glutathione-S-transferase, Thiopurine methyltransferase, N-acetyl transferase and CYP450* | *Azotobacter vinel*and*ii* | *Pseudomonadota* | [266] |
|  |  |  | Aldehyde dehydrogenase, Alcohol dehydrogenase, Epoxide hydrolase, Monoamine oxidase, Glutathione-S-transferase, Thiopurine methyltransferase, N-acetyl transferase and CYP450* | *Bacillus amyloliquefaciens,* *Bacillus cereus,* *Bacillus cytotoxicus,* *Bacillus halodurans,* *Bacillus licheniformis,* *Bacillus megaterium,* *Bacillus* sp. and *Bacillus thuringiensis* | *Bacillota* | [266] |
|  |  |  | Aldehyde dehydrogenase, Alcohol dehydrogenase, Epoxide hydrolase, Monoamine oxidase, Glutathione-S-transferase, Thiopurine methyltransferase, N-acetyl transferase and CYP450* | *Bifidobacterium adolescentis,* *Bifidobacterium angulatum,* *Bifidobacterium bifidum,* *Bifidobacterium breve,* *Bifidobacterium dentium,* *Bifidobacterium longum* and *Bifidobacterium pseudocatenulatum* | *Actinomycetota* | [266] |
|  |  |  | Aldehyde dehydrogenase, Alcohol dehydrogenase, Epoxide hydrolase, Monoamine oxidase, Glutathione-S-transferase, Thiopurine methyltransferase, N-acetyl transferase and CYP450* | *Bordetella bronchiseptica* and *Bordetella petrii* | *Pseudomonadota* | [266] |
|  |  |  | Aldehyde dehydrogenase, Alcohol dehydrogenase, Epoxide hydrolase, Monoamine oxidase, Glutathione-S-transferase, Thiopurine methyltransferase, N-acetyl transferase and CYP450* | *Bradyrhizobium* sp. | *Pseudomonadota* | [266] |
|  |  |  | Aldehyde dehydrogenase, Alcohol dehydrogenase, Epoxide hydrolase, Monoamine oxidase, Glutathione-S-transferase, Thiopurine methyltransferase, N-acetyl transferase and CYP450* | *Brucella abortus, Brucella* sp. and *Brucella suis* | *Pseudomonadota* | [266] |
|  |  |  | Aldehyde dehydrogenase, Alcohol dehydrogenase, Epoxide hydrolase, Monoamine oxidase, Glutathione-S-transferase, Thiopurine methyltransferase, N-acetyl transferase and CYP450* | *Burkholderia cenocepacia, Burkholderia lata* and *Burkholderia* sp. | *Pseudomonadota* | [266] |
|  |  |  | Aldehyde dehydrogenase, Alcohol dehydrogenase, Epoxide hydrolase, Monoamine oxidase, Glutathione-S-transferase, Thiopurine methyltransferase, N-acetyl transferase and CYP450* | *Butyrivibrio proteoclasticus* | *Bacillota* | [266] |
|  |  |  | Aldehyde dehydrogenase, Alcohol dehydrogenase, Epoxide hydrolase, Monoamine oxidase, Glutathione-S-transferase, Thiopurine methyltransferase, N-acetyl transferase and CYP450* | *Caulobacter* sp. | *Pseudomonadota* | [266] |
|  |  |  | Aldehyde dehydrogenase, Alcohol dehydrogenase, Epoxide hydrolase, Monoamine oxidase, Glutathione-S-transferase, Thiopurine methyltransferase, N-acetyl transferase and CYP450* | *Chlorobium phaeobacteroides* | *Chlorobiota* | [266] |
|  |  |  | Aldehyde dehydrogenase, Alcohol dehydrogenase, Epoxide hydrolase, Monoamine oxidase, Glutathione-S-transferase, Thiopurine methyltransferase, N-acetyl transferase and CYP450* | *Citrobacter amalonaticus,* *Citrobacter freundii,* *Citrobacter koseri* and *Citrobacter* sp. | *Pseudomonadota* | [266] |
|  |  |  | Aldehyde dehydrogenase, Alcohol dehydrogenase, Epoxide hydrolase, Monoamine oxidase, Glutathione-S-transferase, Thiopurine methyltransferase, N-acetyl transferase and CYP450* | *Clostridium beijerinckii,* *Clostridium botulinum,* *Clostridium difficile,* *Clostridium perfringens,* *Clostridium saccharolyticum,* *Clostridium* sp. and  *Clostridium sporogenes* | *Bacillota* | [266] |
|  |  |  | Aldehyde dehydrogenase, Alcohol dehydrogenase, Epoxide hydrolase, Monoamine oxidase, Glutathione-S-transferase, Thiopurine methyltransferase, N-acetyl transferase and CYP450* | *Coprococcus catus* | *Bacillota* | [266] |
|  |  |  | Aldehyde dehydrogenase, Alcohol dehydrogenase, Epoxide hydrolase, Monoamine oxidase, Glutathione-S-transferase, Thiopurine methyltransferase, N-acetyl transferase and CYP450* | *Corynebacterium efficiens* and *Corynebacterium glutamicum* | *Actinomycetota* | [266] |
|  |  |  | Aldehyde dehydrogenase, Alcohol dehydrogenase, Epoxide hydrolase, Monoamine oxidase, Glutathione-S-transferase, Thiopurine methyltransferase, N-acetyl transferase and CYP450* | *Cupriavidus necator,* *Cupriavidus pinatubonensis* and *Cupriavidus* sp. | *Pseudomonadota* | [266] |
|  |  |  | Aldehyde dehydrogenase, Alcohol dehydrogenase, Epoxide hydrolase, Glutathione-S-transferase, Thiopurine methyltransferase, N-acetyl transferase and CYP450* | *Dehalococcoides mccartyi* | *Chloroflexota* | [266] |
|  |  |  | Aldehyde dehydrogenase, Alcohol dehydrogenase, Epoxide hydrolase, Monoamine oxidase, Glutathione-S-transferase, Thiopurine methyltransferase, N-acetyl transferase and CYP450* | *Deinococcus deserti* and *Deinococcus geothermalis* | *Deinococcota* | [266] |
|  |  |  | Aldehyde dehydrogenase, Alcohol dehydrogenase, Epoxide hydrolase, Monoamine oxidase, Glutathione-S-transferase, Thiopurine methyltransferase, N-acetyl transferase and CYP450* | *Delftia acidovorans* | *Pseudomonadota* | [266] |
|  |  |  | Aldehyde dehydrogenase, Alcohol dehydrogenase, Epoxide hydrolase, Monoamine oxidase, Glutathione-S-transferase, Thiopurine methyltransferase, N-acetyl transferase and CYP450* | *Desulfitobacterium hafniense* | *Bacillota* | [266] |
|  |  |  | Aldehyde dehydrogenase, Alcohol dehydrogenase, Epoxide hydrolase, Monoamine oxidase, Glutathione-S-transferase, Thiopurine methyltransferase, N-acetyl transferase and CYP450* | *Desulfovibrio desulfuricans* and *Desulfovibrio magneticus* | *Pseudomonadota* | [266] |
|  |  |  | Aldehyde dehydrogenase, Alcohol dehydrogenase, Epoxide hydrolase, Monoamine oxidase, Glutathione-S-transferase, Thiopurine methyltransferase, N-acetyl transferase and CYP450* | *Enterobacter aerogenes,* *Enterobacter cloacae* and *Enterobacter* sp. | *Pseudomonadota* | [266] |
|  |  |  | Aldehyde dehydrogenase, Alcohol dehydrogenase, Epoxide hydrolase, Monoamine oxidase, Glutathione-S-transferase, Thiopurine methyltransferase, N-acetyl transferase and CYP450* | *Enterococcus casseliflavus,* *Enterococcus faecalis,* *Enterococcus faecium* and *Enterococcus gallinarum* | *Bacillota* | [266] |
|  |  |  | Aldehyde dehydrogenase, Alcohol dehydrogenase, Epoxide hydrolase, Monoamine oxidase, Glutathione-S-transferase, Thiopurine methyltransferase, N-acetyl transferase and CYP450* | *Erwinia billingiae* and *Erwinia* sp. | *Pseudomonadota* | [266] |
|  |  |  | Aldehyde dehydrogenase, Alcohol dehydrogenase, Epoxide hydrolase, Monoamine oxidase, Glutathione-S-transferase, Thiopurine methyltransferase, N-acetyl transferase and CYP450* | *Escherichia coli,* *Escherichia fergusonii* and *Escherichia* sp. | *Pseudomonadota* | [266] |
|  |  |  | Aldehyde dehydrogenase, Alcohol dehydrogenase, Epoxide hydrolase, Monoamine oxidase, Glutathione-S-transferase, Thiopurine methyltransferase, N-acetyl transferase and CYP450* | *Eubacterium eligens,* *Eubacterium limosum* and *Eubacterium siraeum* | *Bacillota* | [266] |
|  |  |  | Aldehyde dehydrogenase, Alcohol dehydrogenase, Epoxide hydrolase, Monoamine oxidase, Glutathione-S-transferase, Thiopurine methyltransferase, N-acetyl transferase and CYP450 | *Flavobacterium johnsoniae* | *Bacteroidota* | [266] |
|  |  |  | Aldehyde dehydrogenase, Alcohol dehydrogenase, Epoxide hydrolase, Monoamine oxidase, Glutathione-S-transferase, Thiopurine methyltransferase, N-acetyl transferase and CYP450* | *Geobacillus* sp. | *Bacillota* | [266] |
|  |  |  | Aldehyde dehydrogenase, Alcohol dehydrogenase, Epoxide hydrolase, Monoamine oxidase, Glutathione-S-transferase, Thiopurine methyltransferase, N-acetyl transferase and CYP450* | *Geobacter daltonii* | *Pseudomonadota* | [266] |
|  |  |  | Aldehyde dehydrogenase, Alcohol dehydrogenase, Epoxide hydrolase, Monoamine oxidase, Glutathione-S-transferase, Thiopurine methyltransferase, N-acetyl transferase and CYP450* | *Glaciecola mesophile* and *Glaciecola* sp. | *Pseudomonadota* | [266] |
|  |  |  | Aldehyde dehydrogenase, Alcohol dehydrogenase, Epoxide hydrolase, Monoamine oxidase, Glutathione-S-transferase, Thiopurine methyltransferase, N-acetyl transferase and CYP450* | *Gluconacetobacter diazotrophicus* | *Pseudomonadota* | [266] |
|  |  |  | Aldehyde dehydrogenase, Alcohol dehydrogenase, Epoxide hydrolase, Monoamine oxidase, Glutathione-S-transferase, Thiopurine methyltransferase, N-acetyl transferase and CYP450* | *Gordonia bronchialis* | *Actinomycetota* | [266] |
|  |  |  | Aldehyde dehydrogenase, Alcohol dehydrogenase, Epoxide hydrolase, Monoamine oxidase, Glutathione-S-transferase, Thiopurine methyltransferase, N-acetyl transferase and CYP450* | *Halomonas elongata* | *Pseudomonadota* | [266] |
|  |  |  | Aldehyde dehydrogenase, Alcohol dehydrogenase, Epoxide hydrolase, Monoamine oxidase, Glutathione-S-transferase, Thiopurine methyltransferase, N-acetyl transferase and CYP450* | *Herbaspirillum seropedicae* | *Pseudomonadota* | [266] |
|  |  |  | Aldehyde dehydrogenase, Alcohol dehydrogenase, Epoxide hydrolase, Monoamine oxidase, Glutathione-S-transferase, Thiopurine methyltransferase, N-acetyl transferase and CYP450* | *Klebsiella oxytoca, Klebsiella pneumoniae, Klebsiella* sp. and *Klebsiella variicola* | *Pseudomonadota* | [266] |
|  |  |  | Aldehyde dehydrogenase, Alcohol dehydrogenase, Epoxide hydrolase, Glutathione-S-transferase, Thiopurine methyltransferase, N-acetyl transferase and CYP450* | *Lactobacillus acidophilus,* *Lactobacillus casei,* *Lacticaseibacillus paracasei* and *Lactobacillus rhamnosus* | *Bacillota* | [266] |
|  |  |  | Aldehyde dehydrogenase, Alcohol dehydrogenase, Epoxide hydrolase, Monoamine oxidase, Glutathione-S-transferase, Thiopurine methyltransferase, N-acetyl transferase and CYP450* | *Legionella pneumophila* | *Pseudomonadota* | [266] |
|  |  |  | Aldehyde dehydrogenase, Alcohol dehydrogenase, Epoxide hydrolase, Monoamine oxidase, Glutathione-S-transferase, Thiopurine methyltransferase, N-acetyl transferase and CYP450* | *Leptospira interrogans* | *Spirochaetota* | [266] |
|  |  |  | Aldehyde dehydrogenase, Alcohol dehydrogenase, Epoxide hydrolase, Glutathione-S-transferase, Thiopurine methyltransferase, N-acetyl transferase and CYP450* | *Lysinibacillus sphaericus* | *Bacillota* | [266] |
|  |  |  | Aldehyde dehydrogenase, Alcohol dehydrogenase, Epoxide hydrolase, Monoamine oxidase, Glutathione-S-transferase, Thiopurine methyltransferase, N-acetyl transferase and CYP450* | *Marinobacter adhaerens* and *Marinobacter hydrocarbonoclasticus* | *Pseudomonadota* | [266] |
|  |  |  | Aldehyde dehydrogenase, Alcohol dehydrogenase, Epoxide hydrolase, Monoamine oxidase, Glutathione-S-transferase, Thiopurine methyltransferase, N-acetyl transferase and CYP450* | *Mesorhizobium loti* and *Mesorhizobium ciceri* | *Pseudomonadota* | [266] |
|  |  |  | Aldehyde dehydrogenase, Alcohol dehydrogenase, Epoxide hydrolase, Monoamine oxidase, Glutathione-S-transferase, Thiopurine methyltransferase, N-acetyl transferase and CYP450 | *Methylobacterium nodulans,* *Methylobacterium radiotolerans* and *Methylobacterium* sp. | *Pseudomonadota* | [266] |
|  |  |  | Aldehyde dehydrogenase, Alcohol dehydrogenase, Epoxide hydrolase, Monoamine oxidase, Glutathione-S-transferase, Thiopurine methyltransferase, N-acetyl transferase and CYP450* | *Mycobacterium gilvum,* *Mycobacterium smegmatis,* *Mycobacterium* sp. and *Mycobacterium vanbaalenii* | *Actinomycetota* | [266] |
|  |  |  | Aldehyde dehydrogenase, Alcohol dehydrogenase, Epoxide hydrolase, Monoamine oxidase, Glutathione-S-transferase, Thiopurine methyltransferase, N-acetyl transferase and CYP450* | *Myxococcus xanthus* | *Pseudomonadota* | [266] |
|  |  |  | Aldehyde dehydrogenase, Alcohol dehydrogenase, Epoxide hydrolase, Monoamine oxidase, Glutathione-S-transferase, Thiopurine methyltransferase, N-acetyl transferase and CYP450* | *Nocardia farcinica* | *Actinomycetota* | [266] |
|  |  |  | Aldehyde dehydrogenase, Alcohol dehydrogenase, Epoxide hydrolase, Monoamine oxidase, Glutathione-S-transferase, Thiopurine methyltransferase, N-acetyl transferase and CYP450* | *Nocardiopsis dassonvillei* | *Actinomycetota* | [266] |
|  |  |  | Aldehyde dehydrogenase, Alcohol dehydrogenase, Epoxide hydrolase, Monoamine oxidase, Glutathione-S-transferase, Thiopurine methyltransferase, N-acetyl transferase and CYP450* | *Novosphingobium aromaticivorans* | *Pseudomonadota* | [266] |
|  |  |  | Aldehyde dehydrogenase, Alcohol dehydrogenase, Epoxide hydrolase, Monoamine oxidase, Glutathione-S-transferase, Thiopurine methyltransferase, N-acetyl transferase and CYP450* | *Ochrobactrum anthropi* | *Pseudomonadota* | [266] |
|  |  |  | Aldehyde dehydrogenase, Alcohol dehydrogenase, Epoxide hydrolase, Monoamine oxidase, Glutathione-S-transferase, Thiopurine methyltransferase, N-acetyl transferase and CYP450* | *Paenibacillus* sp. and *Paenibacillus polymyxa* | *Bacillota* | [266] |
|  |  |  | Aldehyde dehydrogenase, Alcohol dehydrogenase, Epoxide hydrolase, Monoamine oxidase, Glutathione-S-transferase, Thiopurine methyltransferase, N-acetyl transferase and CYP450* | *Pantoea* *ananatis* and *Pantoea* sp. | *Pseudomonadota* | [266] |
|  |  |  | Aldehyde dehydrogenase, Alcohol dehydrogenase, Epoxide hydrolase, Monoamine oxidase, Glutathione-S-transferase, Thiopurine methyltransferase, N-acetyl transferase and CYP450* | *Paracoccus denitrificans* | *Pseudomonadota* | [266] |
|  |  |  | Aldehyde dehydrogenase, Alcohol dehydrogenase, Epoxide hydrolase, Monoamine oxidase, Glutathione-S-transferase, Thiopurine methyltransferase, N-acetyl transferase and CYP450* | *Pasteurella multocida* | *Pseudomonadota* | [266] |
|  |  |  | Aldehyde dehydrogenase, Alcohol dehydrogenase, Epoxide hydrolase, Thiopurine methyltransferase, N-acetyl transferase and CYP450* | *Pediococcus pentosaceus* | *Bacillota* | [266] |
|  |  |  | Aldehyde dehydrogenase, Alcohol dehydrogenase, Epoxide hydrolase, Monoamine oxidase, Glutathione-S-transferase, Thiopurine methyltransferase, N-acetyl transferase and CYP450* | *Photobacterium profundum* | *Pseudomonadota* | [266] |
|  |  |  | Aldehyde dehydrogenase, Alcohol dehydrogenase, Epoxide hydrolase, Monoamine oxidase, Glutathione-S-transferase, Thiopurine methyltransferase, N-acetyl transferase and CYP450* | *Pseudoalteromonas atlantica* | *Pseudomonadota* | [266] |
|  |  |  | Aldehyde dehydrogenase, Alcohol dehydrogenase, Epoxide hydrolase, Monoamine oxidase, Glutathione-S-transferase, Thiopurine methyltransferase, N-acetyl transferase and CYP450* | *Pseudomonas aeruginosa,* *Pseudomonas putida* and *Pseudomonas* sp. | *Pseudomonadota* | [266] |
|  |  |  | Aldehyde dehydrogenase, Alcohol dehydrogenase, Epoxide hydrolase, Monoamine oxidase, Glutathione-S-transferase, Thiopurine methyltransferase, N-acetyl transferase and CYP450* | *Psychrobacter* sp. | *Pseudomonadota* | [266] |
|  |  |  | Aldehyde dehydrogenase, Alcohol dehydrogenase, Epoxide hydrolase, Monoamine oxidase, Glutathione-S-transferase, Thiopurine methyltransferase, N-acetyl transferase and CYP450* | *Ralstonia eutropha* and *Ralstonia* sp. | *Pseudomonadota* | [266] |
|  |  |  | Aldehyde dehydrogenase, Alcohol dehydrogenase, Epoxide hydrolase, Monoamine oxidase, Glutathione-S-transferase, Thiopurine methyltransferase, N-acetyl transferase and CYP450* | *Rhizobium leguminosarum* and *Rhizobium* sp. | *Pseudomonadota* | [266] |
|  |  |  | Aldehyde dehydrogenase, Alcohol dehydrogenase, Epoxide hydrolase, Monoamine oxidase, Glutathione-S-transferase, Thiopurine methyltransferase, N-acetyl transferase and CYP450* | *Rhodococcus jostii* and *Rhodococcus* sp. | *Actinomycetota* | [266] |
|  |  |  | Aldehyde dehydrogenase, Alcohol dehydrogenase, Epoxide hydrolase, Monoamine oxidase, Glutathione-S-transferase, Thiopurine methyltransferase, N-acetyl transferase and CYP450* | *Ruminococcus bromii* | *Bacillota* | [266] |
|  |  |  | Aldehyde dehydrogenase, Alcohol dehydrogenase, Epoxide hydrolase, Monoamine oxidase, Glutathione-S-transferase, Thiopurine methyltransferase, N-acetyl transferase and CYP450* | *Saccharomonospora viridis* | *Actinomycetota* | [266] |
|  |  |  | Aldehyde dehydrogenase, Alcohol dehydrogenase, Epoxide hydrolase, Monoamine oxidase, Glutathione-S-transferase, Thiopurine methyltransferase, N-acetyl transferase and CYP450* | *Serratia* sp. and *Serratia marcescens* | *Pseudomonadota* | [266] |
|  |  |  | Aldehyde dehydrogenase, Alcohol dehydrogenase, Epoxide hydrolase, Monoamine oxidase, Glutathione-S-transferase, Thiopurine methyltransferase, N-acetyl transferase and CYP450* | *Shewanella halifaxensis* and *Shewanella pealeana* | *Pseudomonadota* | [266] |
|  |  |  | Aldehyde dehydrogenase, Alcohol dehydrogenase, Epoxide hydrolase, Monoamine oxidase, Glutathione-S-transferase, Thiopurine methyltransferase, N-acetyl transferase and CYP450* | *Sphingobium japonicum* and *Sphingobium* sp. | *Pseudomonadota* | [266] |
|  |  |  | Aldehyde dehydrogenase, Alcohol dehydrogenase, Epoxide hydrolase, Monoamine oxidase, Glutathione-S-transferase, Thiopurine methyltransferase, N-acetyl transferase and CYP450* | *Sphingomonas wittichii* | *Pseudomonadota* | [266] |
|  |  |  | Aldehyde dehydrogenase, Alcohol dehydrogenase, Epoxide hydrolase, Monoamine oxidase, Glutathione-S-transferase, Thiopurine methyltransferase, N-acetyl transferase and CYP450* | *Streptomyces bingchenggensis,* *Streptomyces coelicolor,* *Streptomyces scabiei* and *Streptomyces* sp. | *Actinomycetota* | [266] |
|  |  |  | Aldehyde dehydrogenase, Alcohol dehydrogenase, Epoxide hydrolase, Monoamine oxidase, Glutathione-S-transferase, Thiopurine methyltransferase, N-acetyl transferase and CYP450* | *Thermoanaerobacter tengcongensis* | *Bacillota* | [266] |
|  |  |  | Aldehyde dehydrogenase, Alcohol dehydrogenase, Epoxide hydrolase, Monoamine oxidase, Glutathione-S-transferase, Thiopurine methyltransferase, N-acetyl transferase and CYP450* | *Thermotoga lettingae* | *Thermotogota* | [266] |
|  |  |  | Aldehyde dehydrogenase, Alcohol dehydrogenase, Epoxide hydrolase, Monoamine oxidase, Glutathione-S-transferase, Thiopurine methyltransferase, N-acetyl transferase and CYP450* | *Thermus thermophilus* | *Deinococcota* | [266] |
|  |  |  | Aldehyde dehydrogenase, Alcohol dehydrogenase, Epoxide hydrolase, Monoamine oxidase, Glutathione-S-transferase, Thiopurine methyltransferase, N-acetyl transferase and CYP450* | *Treponema denticola* | *Spirochaetota* | [266] |
|  |  |  | Aldehyde dehydrogenase, Alcohol dehydrogenase, Epoxide hydrolase, Monoamine oxidase, Glutathione-S-transferase, Thiopurine methyltransferase, N-acetyl transferase and CYP450* | *Vibrio cholerae, Vibrio furnissii, Vibrio parahaemolyticus* and *Vibrio* sp. | *Pseudomonadota* | [266] |
|  |  |  | Aldehyde dehydrogenase, Alcohol dehydrogenase, Epoxide hydrolase, Monoamine oxidase, Glutathione-S-transferase, Thiopurine methyltransferase, N-acetyl transferase and CYP450 | *Xanthomonas axonopodis,* *Xanthomonas campestris* and *Xanthomonas citri* | *Pseudomonadota* | [266] |
|  |  |  | Aldehyde dehydrogenase, Alcohol dehydrogenase, Epoxide hydrolase, Monoamine oxidase, Glutathione-S-transferase, Thiopurine methyltransferase, N-acetyl transferase and CYP450* | *Yersinia enterocolitica* | *Pseudomonadota* | [266] |
|  |  |  | Aldehyde dehydrogenase, Alcohol dehydrogenase, Glutathione-S-transferase, Thiopurine methyltransferase, N-acetyl transferase and CYP450* | *Bacteroides eggerthii,* *Bacteroides fragilis, Bacteroides* sp.*,* *Bacteroides thetaiotaomicron,* *Bacteroides uniformis* and *Bacteroides vulgatus* | *Bacteroidota* | [266] |
|  |  |  | Aldehyde dehydrogenase, Alcohol dehydrogenase, Glutathione-S-transferase, Thiopurine methyltransferase, N-acetyl transferase and CYP450* | *Caldicellulosiruptor saccharolyticus* | *Bacillota* | [266] |
|  |  |  | Aldehyde dehydrogenase, Alcohol dehydrogenase, Glutathione-S-transferase, Thiopurine methyltransferase, N-acetyl transferase and CYP450* | *Campylobacter jejuni* and *Campylobacter* sp. | *Pseudomonadota* | [266] |
|  |  |  | Aldehyde dehydrogenase, Alcohol dehydrogenase, Glutathione-S-transferase, Thiopurine methyltransferase, N-acetyl transferase and CYP450* | *Haemophilus parainfluenzae* and *Haemophilus somnus* | *Pseudomonadota* | [266] |
|  |  |  | Aldehyde dehydrogenase, Alcohol dehydrogenase, Glutathione-S-transferase, Thiopurine methyltransferase, N-acetyl transferase and CYP450* | *Helicobacter pylori* | *Pseudomonadota* | [266] |
|  |  |  | Aldehyde dehydrogenase, Alcohol dehydrogenase, Glutathione-S-transferase, Thiopurine methyltransferase, N-acetyl transferase and CYP450* | *Listeria monocytogenes* | *Bacillota* | [266] |
|  |  |  | Aldehyde dehydrogenase, Alcohol dehydrogenase, Glutathione-S-transferase, Thiopurine methyltransferase, N-acetyl transferase and CYP450* | *Neisseria meningitidis* | *Pseudomonadota* | [266] |
|  |  |  | Aldehyde dehydrogenase, Alcohol dehydrogenase, Glutathione-S-transferase, Thiopurine methyltransferase, N-acetyl transferase and CYP450* | *Streptococcus agalactiae,* *Streptococcus dysgalactiae,* *Streptococcus pyogenes* and *Streptococcus* sp. | *Bacillota* | [266] |
|  |  |  | Aldehyde dehydrogenase, Alcohol dehydrogenase, Monoamine oxidase, Glutathione-S-transferase, Thiopurine methyltransferase, N-acetyl transferase and CYP450* | *Acidimicrobium ferrooxidans* | *Actinomycetota* | [266] |
|  |  |  | Aldehyde dehydrogenase, Alcohol dehydrogenase, Monoamine oxidase, Glutathione-S-transferase, Thiopurine methyltransferase, N-acetyl transferase and CYP450* | *Acidithiobacillus ferrivorans, Acidithiobacillus ferrooxidans* and *Acidithiobacillus* sp. | *Pseudomonadota* | [266] |
|  |  |  | Aldehyde dehydrogenase, Alcohol dehydrogenase, Monoamine oxidase, Glutathione-S-transferase, Thiopurine methyltransferase, N-acetyl transferase and CYP450* | *Actinobacillus pleuropneumoniae* and  *Actinobacillus succinogenes* | *Pseudomonadota* | [266] |
|  |  |  | Aldehyde dehydrogenase, Alcohol dehydrogenase, Monoamine oxidase, Glutathione-S-transferase, Thiopurine methyltransferase, N-acetyl transferase and CYP450* | *Aliivibrio fischeri* | *Pseudomonadota* | [266] |
|  |  |  | Aldehyde dehydrogenase, Alcohol dehydrogenase, Monoamine oxidase, Glutathione-S-transferase, Thiopurine methyltransferase, N-acetyl transferase and CYP450* | *Anoxybacillus flavithermus* and *Anoxybacillus* sp. | *Bacillota* | [266] |
|  |  |  | Aldehyde dehydrogenase, Alcohol dehydrogenase, Monoamine oxidase, Glutathione-S-transferase, Thiopurine methyltransferase, N-acetyl transferase and CYP450* | *Bartonella bacilliformis,* *Bartonella birtlesii,* *Bartonella elizabethae,* *Bartonella grahamii* and *Bartonella tribocorum* | *Pseudomonadota* | [266] |
|  |  |  | Aldehyde dehydrogenase, Alcohol dehydrogenase, Monoamine oxidase, Glutathione-S-transferase, Thiopurine methyltransferase, N-acetyl transferase and CYP450* | *Brachyspira pilosicoli* | *Spirochaetota* | [266] |
|  |  |  | Aldehyde dehydrogenase, Alcohol dehydrogenase, Monoamine oxidase, Glutathione-S-transferase, Thiopurine methyltransferase, N-acetyl transferase and CYP450* | *Cronobacter sakazakii* | *Pseudomonadota* | [266] |
|  |  |  | Aldehyde dehydrogenase, Alcohol dehydrogenase, Monoamine oxidase, Glutathione-S-transferase, Thiopurine methyltransferase, N-acetyl transferase and CYP450* | *Leuconostoc gasicomitatum* | *Bacillota* | [266] |
|  |  |  | Aldehyde dehydrogenase, Alcohol dehydrogenase, Monoamine oxidase, Thiopurine methyltransferase, N-acetyl transferase and CYP450* | *Mycoplasma penetrans* | *Mycoplasmatota* | [266] |
|  |  |  | Aldehyde dehydrogenase, Alcohol dehydrogenase, Monoamine oxidase, Glutathione-S-transferase, Thiopurine methyltransferase, N-acetyl transferase and CYP450* | *Propionibacterium acnes* and *Propionibacterium* sp. | *Actinomycetota* | [266] |
|  |  |  | Aldehyde dehydrogenase, Alcohol dehydrogenase, Monoamine oxidase, Glutathione-S-transferase, Thiopurine methyltransferase, N-acetyl transferase and CYP450* | *Proteus mirabilis* | *Pseudomonadota* | [266] |
|  |  |  | Aldehyde dehydrogenase, Alcohol dehydrogenase, Monoamine oxidase, Glutathione-S-transferase, Thiopurine methyltransferase, N-acetyl transferase and CYP450* | *Psychromonas ingrahamii* | *Pseudomonadota* | [266] |
|  |  |  | Aldehyde dehydrogenase, Alcohol dehydrogenase, Monoamine oxidase, Glutathione-S-transferase, Thiopurine methyltransferase, N-acetyl transferase and CYP450* | *Shigella* sp. | *Pseudomonadota* | [266] |
|  |  |  | Aldehyde dehydrogenase, Alcohol dehydrogenase, Monoamine oxidase, Glutathione-S-transferase, Thiopurine methyltransferase, N-acetyl transferase and CYP450* | *Staphylococcus aureus,* *Staphylococcus carnosus* and *Staphylococcus saprophyticus* | *Bacillota* | [266] |
|  |  |  | Aldehyde dehydrogenase, Alcohol dehydrogenase, Monoamine oxidase, Glutathione-S-transferase, Thiopurine methyltransferase, N-acetyl transferase and CYP450* | *Thauera* sp. | *Pseudomonadota* | [266] |
|  |  |  | Aldehyde dehydrogenase, Alcohol dehydrogenase, Thiopurine methyltransferase, N-acetyl transferase and CYP450* | *Desulfotomaculum acetoxidans* and  *Desulfotomaculum reducens* | *Bacillota* | [266] |
|  |  |  | Aldehyde dehydrogenase, Epoxide hydrolase, Monoamine oxidase and N-acetyl transferase* | *Actinoplanes friuliensis* and *Actinoplanes* sp. | *Actinomycetota* | [266] |
|  |  |  | Aldehyde dehydrogenase, Epoxide hydrolase, Monoamine oxidase, Thiopurine methyltransferase, N-acetyl transferase and CYP450* | *Anaerostipes hadrus* | *Bacillota* | [266] |
|  |  |  | Aldehyde dehydrogenase, Glutathione-S-transferase, Thiopurine methyltransferase, N-acetyl transferase and CYP450* | *Capnocytophaga ochracea* | *Bacteroidota* | [266] |
|  |  |  | Aldehyde dehydrogenase, Glutathione-S-transferase, Thiopurine methyltransferase, N-acetyl transferase and CYP450* | *Francisella cf.* | *Pseudomonadota* | [266] |
|  |  |  | Aldehyde dehydrogenase, Glutathione-S-transferase, Thiopurine methyltransferase, N-acetyl transferase and CYP450* | *Parabacteroides distasonis* | *Bacteroidota* | [266] |
|  |  |  | Aldehyde dehydrogenase, Glutathione-S-transferase, Thiopurine methyltransferase, N-acetyl transferase and CYP450* | *Pectobacterium atrosepticum* | *Pseudomonadota* | [266] |
|  |  |  | Aldehyde dehydrogenase, Glutathione-S-transferase, Thiopurine methyltransferase, N-acetyl transferase and CYP450* | *Prevotella ruminicola* | *Bacteroidota* | [266] |
|  |  |  | Aldehyde dehydrogenase, Monoamine oxidase, Glutathione-S-transferase, Thiopurine methyltransferase, N-acetyl transferase and CYP450* | *Aerococcus urinae* | *Bacillota* | [266] |
|  |  |  | Aldehyde dehydrogenase, Monoamine oxidase, Glutathione-S-transferase, Thiopurine methyltransferase, N-acetyl transferase and CYP450* | *Alistipes shahii* | *Bacteroidota* | [266] |
|  |  | - | Aldehyde dehydrogenase, Alcohol dehydrogenase, Monoamine oxidase, Glutathione-S-transferase, Thiopurine methyltransferase, N-acetyl transferase and CYP450* | *Acetobacter aceti, Acetobacter lovaniensis, Acetobacter pasteurianus* and *Acetobacter pomorum* | *Pseudomonadota* | [266] |
|  |  |  | Epoxide hydrolase, Monoamine oxidase, Glutathione-S-transferase, Thiopurine methyltransferase, N-acetyl transferase and CYP450* | *Rhodopseudomonas palustris* | *Pseudomonadota* | [266] |
|  |  |  | Glutathione-S-transferase, Thiopurine methyltransferase, N-acetyl transferase and CYP450* | *Chlamydia pneumoniae* | *Chlamydiota* | [266] |
|  |  |  | Thiopurine methyltransferase, N-acetyl transferase and CYP450* | *Chlamydophila felis* | *Chlamydiota* | [266] |
|  |  | Colon | Aldehyde dehydrogenase | *Escherichia* *coli* | *Pseudomonadota* | [267] |
|  |  |  | Aldehyde dehydrogenase | *Klebsiella pneumoniae* and *Klebsiella oxytoca* | *Pseudomonadota* | [267] |
|  |  |  | Aldehyde dehydrogenase | *Hafnia alvei* | *Pseudomonadota* | [267] |
|  |  |  | Aldehyde dehydrogenase | *Pseudomonas aeruginosa* | *Pseudomonadota* | [267] |
|  |  | Colon | β-Glucosidase | *Butyrivibrio fibrisolvens* | *Bacillota* | [268] |
|  |  |  | β-Glucosidase | *Coprococcus eutactus* and *Coprococcus* sp. | *Bacillota* | [268] |
|  |  |  | β-Glucosidase | *Eubacterium rectale* and *Eubacterium siraeum* | *Bacillota* | [268] |
|  |  |  | β-Glucosidase | *Ruminococcus* sp. | *Bacillota* | [268] |
|  |  |  | β-Glucosidase | *Bifidobacterium adolescentis, Bifidobacterium angulatum, Bifidobacterium breve* and  *Bifidobacterium pseudocatenulatum* | *Actinomycetota* | [268] |
|  |  |  | β-Glucosidase | *Bacteroides thetaiotaomicron* | *Bacteroidota* | [268] |
|  |  |  | β-Glucosidase | *Roseburia faecis* and *Roseburia inulinivorans* | *Bacillota* | [268] |
|  |  |  | β-Glucuronidase | *Faecalibacterium prausnitzii* | *Bacillota* | [268] |
|  |  |  | β-Glucuronidase and β-Glucosidase | *Roseburia hominis* and *Roseburia intestinalis* | *Bacillota* | [268] |
|  |  | Feces | Azo reductase | *Enterococcus faecalis* and *Enterococcus faecium* | *Bacillota* | [57] |
|  |  |  | Azo reductase | *Bacillus cereus* | *Bacillota* | [57] |
|  |  |  | Azo reductase | *Escherichia coli* | *Pseudomonadota* | [57] |
|  |  | - | Azo reductase | *Enterococcus faecalis* | *Bacillota* | [56] |
|  |  | Feces | Azo reductase | *Butyrivibrio* sp. | *Bacillota* | [62] |
|  |  |  | Azo reductase | *Clostridium clostridiiforme, Clostridium paraputrificum, Clostridium nexile* and *Clostridium* sp. | *Bacillota* | [62] |
|  |  |  | Azo reductase | *Eubacterium* sp. and *Eubacterium hadrum* | *Bacillota* | [62] |
|  |  |  | Azo reductase | *Bacteroides* sp. | *Bacteroidota* | [62] |
|  |  | Feces | Azo reductase, nitro reductase, β-Glucuronidase and  β-Glucosidase | *Clostridium clostridiiforme, Clostridium innocuum, Clostridium paraputrificum, Clostridium perfringens* and *Clostridium ramosum* | *Bacillota* | [269] |
|  |  |  | Azo reductase, nitro reductase, β-Glucuronidase and  β-Glucosidase | *Bacteroides distasonis, Bacteroides fragilis, Bacteroides thetaiotaomicron, Bacteroides uniformis* and  *Bacteroides vulgatus* | *Bacillota* | [269] |
|  |  | Feces | Amino hydrolase | *Escherichia coli* | *Pseudomonadota* | [132] |
|  |  | Feces | Ene reductase | *Eubacterium lentum* | *Bacillota* | [138, 139] |
|  |  | Feces | Ene reductase | *Eubacterium ramulus* | *Bacillota* | [270] |
|  |  | Feces | β-Glucanase* | *Eubacterium eligens* | *Bacillota* | [271] |
|  |  |  | β-Glucanase* | *Bacteroides thetaiotaomicron* | *Bacteroidota* | [271] |
|  |  | Feces | β-Glucuronidase | *Clostridium sphenoides* | *Bacillota* | [272] |
|  |  |  | β-Glucuronidase | *Eubacterium rectale* | *Bacillota* | [272] |
|  |  | Feces | β-Glucuronidase | *Escherichia coli* | *Pseudomonadota* | [130] |
|  |  |  | β-Glucuronidase | Parabacteroides distasonis | *Bacteroidota* | [130] |
|  |  | Feces | β-Glucanase | *Pseudomonas aeruginosa* | *Pseudomonadota* | [273] |
|  |  |  | β-Glucuronidase and β-Glucanase | *Bacteroides thetaiotaomicron* | *Bacteroidota* | [273] |
|  |  |  | β-Glucuronidase and β-Glucanase | *Bifidobacterium longum* | *Actinomycetota* | [273] |
|  |  |  | β-Glucuronidase and β-Glucanase | *Clostridium perfringens* | *Bacillota* | [273] |
|  |  |  | β-Glucuronidase and β-Glucanase | *Escherichia coli* | *Pseudomonadota* | [273] |
|  |  |  | β-Glucuronidase and β-Glucanase | *Streptomyces coelicolor* | *Actinomycetota* | [273] |
|  |  | Feces | β-Glucuronidase and β-Glucanase* | *Streptococcus* sp. | *Bacillota* | [274] |
|  |  |  | β-Glucuronidase and β-Glucanase* | *Peptostreptococcus* sp. | *Bacillota* | [274] |
|  |  |  | β-Glucuronidase and β-Glucanase* | *Ruminococcus* sp. | *Bacillota* | [274] |
|  |  |  | β-Glucuronidase and β-Glucanase* | *Clostridium* sp. | *Bacillota* | [274] |
|  |  |  | β-Glucuronidase and β-Glucanase* | *Alistipes* sp. | *Bacteroidota* | [274] |
|  |  | Feces | Dihydropyrimidine dehydrogenase | *Escherichia coli* | *Pseudomonadota* | [112] |
|  |  | Intestine | Azo reductase and nitro reductase | *Clostridium perfringens, Clostridium* *leptum, Clostridium paraputrificum* and *Clostridium* sp. | *Bacillota* | [32, 46] |
|  |  |  | Azo reductase and nitro reductase | *Eubacterium* sp. | *Bacillota* | [46] |
|  |  | Intestine | Nitro reductase | *Clostridium perfringens, Clostridium paraputrificum, Clostridium* sp. and *Clostridium leptum* | *Bacillota* | [32, 171] |
|  |  | Intestine | Nitro reductase | *Enterococcus gallinarum* and *Enterococcus casseliflavus* | *Bacillota* | [159] |
|  |  | Intestine | Nitro reductase | *Enterobacter cloacae* | *Pseudomonadota* | [257] |
|  |  | Intestine | Nitro reductase | *Clostridium leptum* | *Bacillota* | [31, 168] |
|  |  | Intestine | Nitro reductase | *Eubacterium* sp. | *Bacillota* | [31] |
|  |  |  | Nitro reductase | *Clostridium clostridiiforme* | *Bacillota* | [31] |
|  |  |  | Nitro reductase | *Clostridium paraputrificum* | *Bacillota* | [31] |
|  |  | Intestine | β-Glucuronidase* | *Bacteroides capillosus* and *Bacteroides ovatus* | *Bacteroidota* | [275] |
|  |  |  | β-Glucuronidase* | *Parabacteroides johnsonii* and *Parabacteroides merdae* | *Bacteroidota* | [275] |
|  |  |  | β-Glucuronidase* | *Bryantella formatexigens* | *Bacillota* | [275] |
|  |  |  | β-Glucuronidase* | *Clostridium bartlettii,* | *Bacillota* | [275] |
|  |  |  | β-Glucuronidase* | *Faecalibacterium prausnitzii* | *Bacillota* | [275] |
|  |  |  | β-Glucuronidase* | *Roseburia inulinivorans* | *Bacillota* | [275] |
|  |  |  | β-Glucuronidase* | *Ruminococcus gnavus* | *Bacillota* | [275] |
|  |  |  | β-Glucuronidase* | *Subdoligranulum variabil* | *Bacillota* | [275] |
|  |  | Intestine | β-Glucuronidase | *Eubacterium* sp. | *Bacillota* | [276] |
|  |  |  | β-Glucuronidase | *Streptococcus* sp. | *Bacillota* | [276] |
|  |  | - | β-Glucuronidase | *Escherichia coli* | *Pseudomonadota* | [154] |
|  |  | Intestine | β-Glucuronidase* | *Bacteroides* | *Bacteroidota* | [277] |
|  |  | Intestine | β-Glucosidase, nitro reductase and azo reductase | *Clostridium septicum* and *Clostridium sporogenes* | *Bacillota* | [278] |
|  |  |  | β-Glucuronidase and β-Glucosidase | *Bifidobacterium angulatum* and *Bifidobacterium breve* | *Actinomycetota* | [278] |
|  |  |  | β-Glucuronidase, β-Glucosidase, arylsulfatase and azo reductase | *Bacteroides fragilis* | *Bacteroidota* | [278] |
|  |  |  | β-Glucuronidase, β-Glucosidase, arylsulfatase and azo reductase | *Enterococcus faecalis* | *Bacillota* | [278] |
|  |  |  | β-Glucuronidase, β-Glucosidase, arylsulfatase and azo reductase | *Bifidobacterium pseudolongun* | *Actinomycetota* | [278] |
|  |  |  | β-Glucuronidase, β-Glucosidase, nitro reductase and arylsulfatase | *Bifidobacterium bifidum* | *Actinomycetota* | [278] |
|  |  |  | β-Glucuronidase, β-Glucosidase, nitro reductase, arylsulfatase and azo reductase | *Clostridium perfringens* | *Bacillota* | [278] |
|  |  |  | β-Glucuronidase, β-Glucosidase, nitro reductase, arylsulfatase and azo reductase | *Bacteroides ovatus* and *Bacteroides vulgatus* | *Bacteroidota* | [278] |
|  |  |  | β-Glucuronidase, β-Glucosidase, nitro reductase, arylsulfatase and azo reductase | *Escherichia coli* | *Pseudomonadota* | [278] |
|  |  |  | β-Glucuronidase, β-Glucosidase, nitro reductase, arylsulfatase and azo reductase | *Enterococcus faecium* | *Bacillota* | [278] |
|  |  |  | β-Glucuronidase, β-Glucosidase, nitro reductase and azo reductase | *Clostridium bifermentans* and *Clostridium butyricum* | *Bacillota* | [278] |
|  |  |  | β-Glucuronidase, β-Glucosidase, nitro reductase and azo reductase | *Lactobacillus acidophilus* | *Bacillota* | [278] |
|  |  |  | β-Glucuronidase, β-Glucosidase, nitro reductase and azo reductase | *Bifidobacterium longum* | *Actinomycetota* | [278] |
|  |  | - | β-Lactamase | *Enterobacter cloacae* | *Pseudomonadota* | [124] |
|  |  |  | β-Lactamase | *Enterobacter aerogenes* | *Pseudomonadota* | [124] |
|  |  | - | Sulfatase | *Hungatella hathewayi* | *Bacillota* | [279] |
|  |  |  | Sulfatase | *Escherichia coli* | *Pseudomonadota* | [279] |
|  |  |  | Sulfatase | *Alistipes obesi* | *Bacteroidota* | [279] |
|  |  |  | Sulfatase | *Culturomica massiliensis* | *Bacteroidota* | [279] |
|  |  |  | Sulfatase | *Bacteroides fragilis* | *Bacteroidota* | [279] |
|  |  | Large intestine | β-Glucuronidase | *Ruminococcus* | *Bacillota* | [280] |
|  |  | - | Phosphohydrolase | *Lactobacillus delbrueckii, Limosilactobacillus fermentum,* and *Lactobacillus plantarum* | *Bacillota* | [16] |
|  |  |  | Phosphohydrolase | *Enterococcus faecalis* and *Escherichia coli* | *Pseudomonadota* | [16] |
|  |  | Stomach | N-acetyl transferase | *Helicobacter pylori* | *Pseudomonadota* | [281] |

**Table S9.** Summary of digestive enzymes that produced by gut microbes from human and fauna.

| **Group** | **Host** | **Position** | **Enzyme** | **Taxonomic information** | | **References** |
| --- | --- | --- | --- | --- | --- | --- |
|  |  |  |  | **Genus** | **Phylum** |  |
| Invertebrate | Freshwater crustacean (penaeid shrimp) | - | Amylase, cellulase and gelatinase | *Cytophaga* | *Bacteroidota* | [282] |
|  |  |  | Lipase | *Photobacterium* | *Pseudomonadota* | [282] |
|  |  |  | Amylase, gelatinase and lipase | *Pseudomonas* | *Pseudomonadota* | [282] |
|  |  |  | Amylase, gelatinase and lipase | *Caulobacter* | *Pseudomonadota* | [282] |
|  |  |  | Amylase, gelatinase and lipase | *Xanthomonas* | *Pseudomonadota* | [282] |
|  |  |  | Amylase, gelatinase and lipase | *Alteromonas* | *Pseudomonadota* | [282] |
|  |  |  | Amylase, gelatinase and lipase | *Chromobacterium* | *Pseudomonadota* | [282] |
|  |  |  | Amylase, gelatinase and lipase | *Aeromonas* | *Pseudomonadota* | [282] |
|  |  |  | Amylase, gelatinase and lipase | *Vibrio* | *Fusobacteriota* | [282] |
|  |  |  | Amylase, cellulase, gelatinase and lipase | *Flavobacterium* | *Bacteroidota* | [282] |
|  |  |  | Amylase, cellulase, gelatinase and lipase | *Alcaligenes* | *Pseudomonadota* | [282] |
|  | Marine amphipod (*Boeckosimus affinis*) | Midgut, anal plates and faece | Chitinase, lipase and protease | *Vibrio* | *Fusobacteriota* | [283] |
|  | Marine annelid (*Grania*) | - | Cellulase | *Enterobacter* | *Pseudomonadota* | [284] |
|  |  |  | Cellulase | *Rhodotorula* | *Dikarya* | [284] |
|  |  |  | Cellulase | *Cystobasidium* | *Dikarya* | [284] |
|  |  |  | Amylase, cellulase, lipase and protease | *Pseudomonas* | *Pseudomonadota* | [284] |
|  |  |  | Amylase, cellulase, lipase and protease | *Psychrobacter* | *Pseudomonadota* | [284] |
|  |  |  | Agarase, amylase, cellulase, lipase and protease | *Flavobacterium* | *Bacteroidota* | [284] |
|  | Marine copepods | - | Chitinase, lipase and protease | *Vibrio* | *Fusobacteriota* | [285] |
|  | Marine crustacean (Pacific white shrimp) | - | Amylase, chitinase and lipase | *Pseudoalteromonas* | *Pseudomonadota* | [286] |
|  |  |  | Amylase, chitinase and lipase | *Vibrio* | *Fusobacteriota* | [286] |
|  | Marine crustacean (spiny lobster) | - | Cellulase | *Flavobacterium* | *Bacteroidota* | [287] |
|  | Marine crustacean (thalassinid prawns) | - | Chitinase, lipase, lysozyme and protease | *Vibrio* | *Fusobacteriota* | [288] |
|  |  |  | Chitinase, lipase, lysozyme and protease | *Pseudomonas* | *Pseudomonadota* | [288] |
|  | Marine crustacean  (tiger shrimp) | - | Amylase, cellulase, lipase and protease | *Bacillus* | *Bacillota* | [289] |
|  | Marine echinoderm (sea cucumber) | - | Protease | *Virgibacillus* | *Bacillota* | [290] |
|  |  |  | Amylase, cellulase and protease | *Bacillus* | *Bacillota* | [290] |
| Fish | Freshwater fish | - | Cellulase | *Clostridium* | *Bacillota* | [291] |
|  |  |  | Cellulase | *Bacillus* | *Bacillota* | [291] |
|  |  |  | Cellulase | *Paenibacillus* | *Bacillota* | [291] |
|  |  |  | Cellulase | *Ruminococcus* | *Bacillota* | [291] |
|  |  |  | Cellulase | *Streptococcus* | *Bacillota* | [291] |
|  |  |  | Cellulase | *Citrobacter* | *Pseudomonadota* | [291] |
|  |  |  | Cellulase | *Erwinia* | *Pseudomonadota* | [291] |
|  |  |  | Cellulase | *Pseudomonas* | *Pseudomonadota* | [291] |
|  |  |  | Cellulase | *Brevundimonas* | *Pseudomonadota* | [291] |
|  |  |  | Cellulase | *Pseudoxanthomonas* | *Pseudomonadota* | [291] |
|  |  |  | Cellulase | *Methylobacterium* | *Pseudomonadota* | [291] |
|  |  |  | Cellulase | *Leptotrichia* | *Fusobacteriota* | [291] |
|  |  |  | Cellulase | *Vibrio* | *Fusobacteriota* | [291] |
|  |  |  | Cellulase | *Actinomyces* | *Actinomycetota* | [291] |
|  |  |  | Protease | *Cetobacterium* | *Fusobacteriota* | [291] |
|  |  |  | Protease | *Halomonas* | *Pseudomonadota* | [291] |
|  | Freshwater fish (angelfish and oscars) | - | Lipase | *Clostridium* | *Bacillota* | [263] |
|  |  |  | Lipase | *Fusobacterium* | *Pseudomonadota* | [263] |
|  |  |  | Lipase | *Porphyromonas* | *Bacteroidota* | [263] |
|  |  |  | Lipase | *Bacteroides* | *Bacteroidota* | [263] |
|  | Freshwater fish (ayu, carp, channel catfish, Japanese eel and tilapia) | - | Amylase | *Clostridium* | *Bacillota* | [292] |
|  |  |  | Amylase | *Pseudomonas* | *Pseudomonadota* | [292] |
|  |  |  | Amylase | *Aeromonas* | *Pseudomonadota* | [292] |
|  | Freshwater fish (carps) | - | Amylase and protease | *Bacillus* | *Bacillota* | [293] |
|  |  |  | Amylase and protease | *Solibacillus* | *Bacillota* | [293] |
|  | Freshwater fish (carps) | - | Phytase | *Rhodococcus* | *Actinomycetota* | [294] |
|  | Freshwater fish (carps and nile tilapia) | - | Chitinase | *Bacillus* | *Bacillota* | [295] |
|  |  |  | Chitinase, lipase, lipase, phytase, protease and xylanase | *Pichia* | *Ascomycota* | [296] |
|  |  |  | Amylase, cellulase, chitinase, lipase, lipase, phytase, protease and xylanase | *C*and*ida* | *Ascomycota* | [296] |
|  | Freshwater fish (catla, rohu and mrigal) | - | Amylase, cellulase and protease | *Citrobacter* | *Pseudomonadota* | [297] |
|  |  |  | Amylase, cellulase and protease | *Enterobacter* | *Pseudomonadota* | [297] |
|  |  |  | Amylase, cellulase and protease | *Bacillus* | *Bacillota* | [297] |
|  | Freshwater fish (catla, silver carp, rohu, grass carp, mrigal, common carp, bata, kalbasu, climbing perch and nile tilapia) | - | Phytase | *Bacillus* | *Bacillota* | [298] |
|  | Freshwater fish (catla, silver carp, rohu, grass carp, mrigal, common carp, bata, kalbasu, tilapia and nile tilapia) | - | Tannase | *Enterobacter* | *Pseudomonadota* | [299] |
|  |  |  | Tannase | *Pichia* | *Ascomycota* | [299] |
|  |  |  | Tannase | *C*and*ida* | *Ascomycota* | [299] |
|  |  |  | Tannase | *Capillus* | *Cyanobacteriota* | [299] |
|  | Freshwater fish (*Ctenopharyngodon idellus*) | - | Cellulase | *Aeromonas* | *Pseudomonadota* | [300] |
|  | Freshwater fish (*Ctenopharyngodon idellus*) | - | Cellulase, lipase and protease | *Aeromonas* | *Pseudomonadota* | [301] |
|  | Freshwater fish (*Ctenopharyngodon idellus*) | - | Cellulase, lipase and protease | *Aeromonas* | *Pseudomonadota* | [301] |
|  | Freshwater fish (*Ctenopharyngodon idellus*) | - | Cellulase | *Klebsiella* | *Pseudomonadota* | [302] |
|  |  |  | Cellulase | *Pseudomonas* | *Pseudomonadota* | [302] |
|  |  |  | Cellulase | *Unclassified* | *Pseudomonadota* | [302] |
|  |  |  | Cellulase | *Brevibacillus* | *Bacillota* | [302] |
|  |  |  | Cellulase | *Hydrotalea* | *Bacteroidota* | [302] |
|  |  |  | Cellulase | *Raoultella* | *Pseudomonadota* | [302, 303] |
|  |  |  | Cellulase | *Enterococcus* | *Bacillota* | [302, 303] |
|  |  |  | Cellulase | *Aeromonas* | *Pseudomonadota* | [302, 303] |
|  |  |  | Cellulase | *Enterobacter* | *Pseudomonadota* | [302, 303] |
|  |  |  | Cellulase | *Citrobacter* | *Pseudomonadota* | [302] |
|  |  |  | Cellulase | *Bacillus* | *Bacillota* | [302, 303] |
|  | Freshwater fish (common carp, crucian carp and gray mullet) | - | Chitinase | *Aeromonas* | *Pseudomonadota* | [304] |
|  | Freshwater fish (*Dicentrarchus labrax*) | - | Lipase | *Acinetobacter* | *Pseudomonadota* | [305] |
|  |  |  | Lipase | *Enterobacter* | *Pseudomonadota* | [305] |
|  |  |  | Lipase | *Pseudomonas* | *Pseudomonadota* | [305] |
|  |  |  | Amylase, lipase and protease | *Vibrio* | *Fusobacteriota* | [305] |
|  | Freshwater fish (gray mullet) | - | Protease | *Pseudomonas* | *Pseudomonadota* | [306] |
|  |  |  | Lipase and protease | *Aeromonas* | *Pseudomonadota* | [306] |
|  |  |  | Amylase, chitinase and protease | *Acinetobacter* | *Pseudomonadota* | [306] |
|  |  |  | Amylase, chitinase, lipase and protease | *Enterobacter* | *Pseudomonadota* | [306] |
|  |  |  | Amylase, chitinase, lipase and protease | *Vibrio* | *Fusobacteriota* | [306] |
|  | Freshwater fish (*Labeo bata*) | - | Amylase, cellulase and protease | *Bacillus* | *Bacillota* | [307] |
|  | Freshwater fish (*Labeo rohita*) | - | Amylase, cellulase and protease | *Bacillus* | *Bacillota* | [308] |
|  | Freshwater fish (murrel, stinging catfish) | - | Cellulase | *Bacillus* | *Bacillota* | [309] |
|  | Freshwater fish (mrigal) | - | Amylase, cellulase, lipase, phytase, protease and xylanase | *Bacillus* | *Bacillota* | [310] |
|  | Freshwater fish (nile tilapia) | - | Protease and xylanase | *Rummeliibacillus* | *Bacillota* | [311] |
|  | Freshwater fish (pacu and piau-com-pinta) | - | Cellulase | *Bacillus* | *Bacillota* | [312] |
|  | Freshwater fish (*Panaque nigrolineatus*) | - | Cellulase | *Azospirillum* | *Pseudomonadota* | [313] |
|  |  |  | Cellulase | *Bosea* | *Pseudomonadota* | [313] |
|  |  |  | Cellulase | *Achromobacter* | *Pseudomonadota* | [313] |
|  |  |  | Cellulase | *Pseudoxanthomonas* | *Pseudomonadota* | [313] |
|  |  |  | Cellulase | *Bacillus* | *Bacillota* | [313] |
|  |  |  | Cellulase | *Clostridium* | *Bacillota* | [313] |
|  |  |  | Cellulase | *Sporomusa* | *Bacillota* | [313] |
|  |  |  | Cellulase | *Curtobacterium* | *Actinomycetota* | [313] |
|  |  |  | Cellulase | *Flavobacterium* | *Bacteroidota* | [313] |
|  | Freshwater fish (*Rutilus* *rut​​ilus*) | - | Amylase and protease | *Aeromonas* | *Pseudomonadota* | [314] |
|  |  |  | Amylase and protease | *Pseudomonas* | *Pseudomonadota* | [314] |
|  |  |  | Amylase and protease | *Flavobacterium* | *Bacteroidota* | [314] |
|  |  |  | Amylase and protease | *Enterobacter* | *Pseudomonadota* | [314] |
|  |  |  | Amylase and protease | *Micrococcus* | *Actinomycetota* | [314] |
|  | Freshwater fish (snow trout and anjak) | - | Amylase and protease | *Microbacterium* | *Actinomycetota* | [315] |
|  |  |  | Amylase and protease | *Arthrobacter* | *Actinomycetota* | [315] |
|  |  |  | Amylase and protease | *Streptomyces* | *Actinomycetota* | [315] |
|  |  |  | Amylase and protease | *Kocuria* | *Actinomycetota* | [315] |
|  |  |  | Amylase and protease | *Saccharomonospora* | *Actinomycetota* | [315] |
|  |  |  | Amylase and protease | *Micromonospora* | *Actinomycetota* | [315] |
|  | Freshwater fish (tilapia) | - | Amylase, chitinase, lipase and protease | *Aeromonas* | *Pseudomonadota* | [316, 317] |
|  |  |  | Amylase, chitinase, lipase and protease | *Vibrio* | *Fusobacteriota* | [316, 317] |
|  |  |  | Amylase, chitinase, lipase and protease | *Plesiomonas* | *Pseudomonadota* | [316, 317] |
|  | Freshwater fish (tilapia and grass carp) | - | Amylase, cellulase and protease | *Bacillus* | *Bacillota* | [318] |
|  | Freshwater fish (walking catfish) | - | Amylase, lipase and protease | *Bacillus* | *Bacillota* | [319] |
|  | Marine fish  (dover sole) | - | Chitinase | *Acinetobacter* | *Pseudomonadota* | [320] |
|  |  |  | Protease | *Alcaligenes* | *Pseudomonadota* | [320] |
|  |  |  | Protease | *Staphylococcus* | *Bacillota* | [320] |
|  |  |  | Protease | *Micrococcus* | *Actinomycetota* | [320] |
|  |  |  | Chitinase and protease | *Photobacterium* | *Pseudomonadota* | [320] |
|  |  |  | Chitinase and protease | *Enterobacter* | *Pseudomonadota* | [320] |
|  |  |  | Chitinase and protease | *Vibrio* | *Fusobacteriota* | [320] |
|  |  |  | Chitinase and protease | *Flavobacterium* | *Bacteroidota* | [320] |
|  | Marine fish (arabesque greenling, pacific cod and sand flounder) | - | Protease | *Pseudomonas* | *Pseudomonadota* | [321] |
|  | Marine fish (Arctic charr) | - | Lipase | *Vibrio* | *Fusobacteriota* | [322] |
|  | Marine fish (Atlantic cod) | - | Amylase and chitinase | *Jeotgalibacillus* | *Bacillota* | [323] |
|  |  |  | Amylase, lipase and protease | *Pseudomonas* | *Pseudomonadota* | [323] |
|  |  |  | Amylase, phytase and protease | *Staphylococcus* | *Bacillota* | [323] |
|  |  |  | Amylase, phytase and protease | *Carnobacterium* | *Bacillota* | [323] |
|  |  |  | Amylase, cellulase, chitinase, phytase and protease | *Psychrobacter* | *Pseudomonadota* | [323] |
|  |  |  | Amylase, cellulase, chitinase, lipase, phytase and protease | *Brochothrix* | *Bacillota* | [323] |
|  | Marine fish (Atlantic salmon) | - | Amylase, cellulase, chitinase and phytase | *Agrococcus* | *Actinomycetota* | [324] |
|  |  |  | Cellulase and protease | *Carnobacterium* | *Bacillota* | [324] |
|  |  |  | Amylase, cellulase and protease | *Pseudomonas* | *Pseudomonadota* | [324] |
|  |  |  | Amylase, cellulase, phytase and protease | *Staphylococcus* | *Bacillota* | [324] |
|  |  |  | Amylase, cellulase, chitinase, lipase, phytase and protease | *Acinetobacter* | *Pseudomonadota* | [324] |
|  |  |  | Amylase, cellulase, chitinase, lipase, phytase and protease | *Bacillus* | *Bacillota* | [324] |
|  | Marine fish (Atlantic salmon) | - | Amylase, cellulase, chitinase, lipase and protease | *Pseudomonas* | *Pseudomonadota* | [325] |
|  |  |  | Chitinase and protease | *Psychrobacter* | *Pseudomonadota* | [325] |
|  | Marine fish (bombay duck) | - | Lipase | *Corynebacterium* | *Actinomycetota* | [326] |
|  |  |  | Lipase and pectinase | *Microbacterium* | *Actinomycetota* | [326] |
|  |  |  | Lipase and protease | *Micrococcus* | *Actinomycetota* | [326] |
|  |  |  | Lipase and protease | *Kocuria* | *Actinomycetota* | [326] |
|  |  |  | Lipase, pectinase and protease | *Staphylococcus* | *Bacillota* | [326] |
|  |  |  | Amylase, lipase, pectinase and protease | *Exiguobacterium* | *Bacillota* | [326] |
|  | Marine fish (*Carangoides praeustus, Filimanus similis, Sardinella longiceps* and *illago sihama*) | - | Cellulase | *Pseudomonas* | *Pseudomonadota* | [327] |
|  |  |  | Cellulase | *Klebsiella* | *Pseudomonadota* | [327] |
|  |  |  | Cellulase | *Bacillus* | *Bacillota* | [327] |
|  |  |  | Cellulase | *Vibrio* | *Fusobacteriota* | [327] |
|  | Marine fish (Japanese flounder) | - | Cellulase | *Vibrio* | *Fusobacteriota* | [328] |
|  | Marine fish (mangrove red snapper) | - | Amylase, cellulase, chitinase, lipase, pectinase and protease | *Aeromonas* | *Pseudomonadota* | [329] |
|  |  |  | Amylase, cellulase, chitinase, lipase, pectinase and protease | *Vibrio* | *Fusobacteriota* | [329] |
|  |  |  | Amylase, cellulase, chitinase, lipase, pectinase and protease | *Bacillus* | *Bacillota* | [329] |
|  | Marine fish (milkfish) | - | Amylase, cellulase and protease | *Vibrio* | *Fusobacteriota* | [330] |
|  |  |  | Amylase, cellulase and protease | *Bacillus* | *Bacillota* | [330] |
|  | Marine fish (*Mugil cephalus*) | - | Protease | *Bacillus* | *Bacillota* | [331] |
|  | Marine fish (rabbitfish) | - | Amylase, cellulase and protease | *Bacillus* | *Bacillota* | [332] |
|  | Marine fish (*Rastrelliger kanagurta* and *Sillago sihama*) | - | Amylase | *Bacillus* | *Bacillota* | [333] |
|  | Marine fish (salmon) | - | Protease | *Flavobacterium* | *Bacteroidota* | [334] |
|  | Marine fish (salmon) | - | Lipase | *Agrobacterium* | *Pseudomonadota* | [335] |
|  |  |  | Lipase | *Pseudomonas* | *Pseudomonadota* | [335] |
|  |  |  | Lipase | *Staphylococcus* | *Bacillota* | [335] |
|  |  |  | Lipase | *Brevibacterium* | *Actinomycetota* | [335] |
|  |  |  | Lipase | *Microbacterium* | *Actinomycetota* | [335] |
|  | Marine fish (Scatophagus argus, Terapon jarbua, Mystus gulio and Etroplus suratensis) | - | Amylase, cellulase, lipase and protease | *Bacillus* | *Bacillota* | [336] |
|  |  |  | Amylase, cellulase, lipase and protease | *Brevibacillus* | *Bacillota* | [336] |
|  | Marine fish (Sea cucumber and *Hexagrammos* *otakii*) | - | Phytase | *Kodamaea* | *Ascomycota* | [337] |
|  | Marine fish (southern flounder) | - | Lipase | *Clostridium* | *Bacillota* | [263] |
|  | Marine fish (tilapia) | - | Amylase, cellulase, lipase and protease | *Aeromonas* | *Pseudomonadota* | [316] |
|  |  |  | Amylase, cellulase, lipase and protease | *Vibrio* | *Fusobacteriota* | [316] |
|  | Marine fish (16 species) | - | Chitinase | *Marinobacter* | *Pseudomonadota* | [338] |
|  |  |  | Chitinase | *Ferrimonas* | *Pseudomonadota* | [338] |
|  |  |  | Chitinase | *Pseudoalteromonas* | *Pseudomonadota* | [338] |
|  |  |  | Chitinase | *Grimontia* | *Pseudomonadota* | [338] |
|  |  |  | Chitinase | *Photobacterium* | *Pseudomonadota* | [338] |
|  |  |  | Chitinase | *Enterovibrio* | *Pseudomonadota* | [338] |
|  |  |  | Chitinase | *Vibrio* | *Fusobacteriota* | [338] |
| Non-human mammals | Cattle | Rumen | Cellulase | *Methanobrevibacter* | *Euryarchaeota* | [339] |
|  | Cattle | Rumen | Cellulase | *Methanobrevibacter* | *Euryarchaeota* | [340] |
|  | Cattle | Rumen | Cellulase | *Fibrobacter* | *Fibrobacterota* | [341] |
|  |  |  | Cellulase | *Ruminococcus* | *Bacillota* | [341] |
|  | Cattle | Rumen | Cellulase | *Fibrobacter* | *Fibrobacterota* | [342] |
|  |  |  | Cellulase | *Ruminococcus* | *Bacillota* | [342] |
|  | Cattle | - | Cellulase | *Anaerosporobacter* | *Bacillota* | [343] |
|  |  |  | Cellulase | *Anaerovorax* | *Bacillota* | [343] |
|  |  |  | Cellulase | *Eubacterium* | *Bacillota* | [343] |
|  |  |  | Cellulase | *Paenibacillus* | *Bacillota* | [343] |
|  |  |  | Cellulase | *Sedimentibacter* | *Bacillota* | [343] |
|  |  |  | Cellulase | Sporanaerobacter | *Bacillota* | [343] |
|  |  |  | Cellulase | *Staphylococcus* | *Bacillota* | [343] |
|  |  |  | Cellulase | *Tissierella* | *Bacillota* | [343] |
|  |  |  | Cellulase | *Proteiniphilum* | *Bacteroidota* | [343] |
|  |  |  | Cellulase | *Citrobacter* | *Pseudomonadota* | [343] |
|  |  |  | Cellulase | *Morganella* | *Pseudomonadota* | [343] |
|  |  |  | Cellulase | *Paraeggerthella* | *Actinomycetota* | [343] |
|  |  |  | Cellulase, pectinase and xylanase | *Anaerofilum* | *Bacillota* | [343] |
|  |  |  | Cellulase, pectinase and xylanase | *Blautia* | *Bacillota* | [343] |
|  |  |  | Cellulase, pectinase and xylanase | *Clostridium* | *Bacillota* | [343] |
|  |  |  | Cellulase, pectinase and xylanase | *Enterococcus* | *Bacillota* | [343] |
|  |  |  | Cellulase, pectinase and xylanase | *Lactonifactor* | *Bacillota* | [343] |
|  |  |  | Cellulase, pectinase and xylanase | *Oscillibacter* | *Bacillota* | [343] |
|  |  |  | Cellulase, pectinase and xylanase | *Ruminococcus* | *Bacillota* | [343] |
|  |  |  | Cellulase, pectinase and xylanase | *Streptococcus* | *Bacillota* | [343] |
|  |  |  | Cellulase, pectinase and xylanase | *Bacteroides* | *Bacteroidota* | [343] |
|  |  |  | Cellulase, pectinase and xylanase | *Dysgonomonas* | *Bacteroidota* | [343] |
|  |  |  | Cellulase, pectinase and xylanase | *Parabacteroides* | *Bacteroidota* | [343] |
|  |  |  | Cellulase, pectinase and xylanase | *Aeromonas* | *Pseudomonadota* | [343] |
|  |  |  | Cellulase, pectinase and xylanase | *Desulfovibrio* | *Pseudomonadota* | [343] |
|  |  |  | Cellulase, pectinase and xylanase | *Proteus* | *Pseudomonadota* | [343] |
|  |  |  | Cellulase, pectinase and xylanase | *Cloacibacillus* | *Synergistota* | [343] |
|  |  |  | Cellulase, pectinase and xylanase | *Fusobacterium* | *Fusobacteriota* | [343] |
|  |  |  | Pectinase and xylanase | *Anaerostipes* | *Bacillota* | [343] |
|  |  |  | Pectinase and xylanase | *Butyricicoccus* | *Bacillota* | [343] |
|  |  |  | Pectinase and xylanase | *Flavonifractor* | *Bacillota* | [343] |
|  |  |  | Pectinase and xylanase | *Megasphaera* | *Bacillota* | [343] |
|  |  |  | Pectinase and xylanase | *Peptostreptococcus* | *Bacillota* | [343] |
|  |  |  | Pectinase and xylanase | *Escherichia* | *Pseudomonadota* | [343] |
|  |  |  | Pectinase and xylanase | *Bifidobacterium* | *Actinomycetota* | [343] |
|  |  |  | Pectinase and xylanase | *Olsenella* | *Actinomycetota* | [343] |
|  |  |  | Pectinase and xylanase | *Propionibacterium* | *Actinomycetota* | [343] |
|  | Donkey | Rumen | Cellulase | *Ruminococcus* | *Bacillota* | [344] |
|  | Pig | Rumen and cecum | Cellulase | *Fibrobacter* | *Fibrobacterota* | [345] |
|  |  |  | Cellulase | *Ruminococcus* | *Bacillota* | [345] |
|  |  |  | Cellulase | *Butyrivibrio* | *Bacillota* | [345] |
|  |  |  | Cellulase | *Prevotella* | *Bacteroidota* | [345] |
|  | Pony | Rumen | Cellulase | *Fibrobacter* | *Fibrobacterota* | [344] |
|  | Ruminants | Rumen | Cellulase | *Butyrivibrio* | *Bacillota* | [346] |
|  | Ruminants | Rumen | Cellulase | *Bacteroides* | *Bacteroidota* | [347] |
|  |  |  | Cellulase | *Ruminococcus* | *Bacillota* | [347] |
|  |  |  | Cellulase | *Butyrivibrio* | *Bacillota* | [347] |
|  | Ruminants | Rumen | Cellulase | *Fibrobacter* | *Fibrobacterota* | [348] |
|  |  |  | Cellulase | *Bacteroides* | *Bacteroidota* | [348] |
|  |  |  | Cellulase | *Lachnospira* | *Bacillota* | [348] |
|  | Sheep | Rumen | Cellulase | *Ruminococcus* | *Bacillota* | [349] |
|  | Sheep | Rumen | Cellulase | *Fibrobacter* | *Fibrobacterota* | [350] |
|  |  |  | Cellulase | *Ruminococcus* | *Bacillota* | [350] |
|  | Sheep | Rumen and cecum | Cellulase | *Fibrobacter* | *Fibrobacterota* | [351] |
|  |  |  | Cellulase | *Ruminococcus* | *Bacillota* | [351] |
| Human |  | Colon | Amylase | *Ruminococcus* | *Bacillota* | [352] |
|  |  | Colon | Amylase | *Bacteroides* | *Bacteroidota* | [353] |
|  |  |  | Amylase | *Bifidobacterium* | *Actinomycetota* | [353] |
|  |  |  | Amylase | *Clostridium* | *Bacillota* | [353] |
|  |  |  | Amylase | *Eubacterium* | *Bacillota* | [353] |
|  |  |  | Amylase | *Peptostreptococcus* | *Bacillota* | [353] |
|  |  |  | Amylase | *Lactobacillus* | *Bacillota* | [353] |
|  |  | Colon | Amylase | *Bacteroides* | *Bacteroidota* | [354] |
|  |  | Colon | Amylase | *Prevotella* | *Bacteroidota* | [355] |
|  |  |  | Amylase | *Clostridium* | *Bacillota* | [355] |
|  |  |  | Amylase | *Butyrivibrio* | *Bacillota* | [355] |
|  |  |  | Amylase | *Eubacterium* | *Bacillota* | [355] |
|  |  |  | Amylase | *Ruminococcus* | *Bacillota* | [355] |
|  |  |  | Amylase | *Roseburia* | *Bacillota* | [355] |
|  |  |  | Amylase | *Akkermansi* | *Verrucomicrobiota* | [355] |
|  |  | Colon | Amylase and pectinase | *Eubacterium* | *Bacillota* | [356, 357] |
|  |  |  | Amylase and pectinase | *Peptostreptococcus* | *Bacillota* | [356, 357] |
|  |  |  | Amylase, pectinase and protease | *Bifidobacterium* | *Actinomycetota* | [356, 357] |
|  |  |  | Amylase, pectinase and protease | *Ruminococcus* | *Bacillota* | [356, 357] |
|  |  |  | Amylase, pectinase and protease | *Bacteroides* | *Bacteroidota* | [356, 357] |
|  |  | Colon | Amylase and xylanase | *Clostridium* | *Bacillota* | [273] |
|  |  |  | Amylase and xylanase | *Escherichia* | *Pseudomonadota* | [273] |
|  |  |  | Amylase, arabinanase, chitinase and xylanase | *Bacteroides* | *Bacteroidota* | [273] |
|  |  |  | Amylase, arabinanase, chitinase and xylanase | *Streptomyces* | *Actinomycetota* | [273] |
|  |  |  | Chitinase | *Pseudomonas* | *Pseudomonadota* | [273] |
|  |  |  | Xylanase | *Bifidobacterium* | *Actinomycetota* | [273] |
|  |  | Colon | Protease | *Bacteroides* | *Bacteroidota* | [358] |
|  |  |  | Protease | *Clostridium* | *Bacillota* | [358] |
|  |  |  | Protease | *Enterobacter* | *Pseudomonadota* | [358] |
|  |  | Colon | Protease | *Lactobacillus* | *Bacillota* | [359] |
|  |  |  | Protease | *Streptococcus* | *Bacillota* | [359] |
|  |  |  | Protease | *Staphylococcus* | *Bacillota* | [359] |
|  |  |  | Protease | *Clostridium* | *Bacillota* | [359] |
|  |  |  | Protease | *Bifidobacterium* | *Actinomycetota* | [359] |
|  |  | Feces | Amylase | *Eubacterium* | *Bacillota* | [271] |
|  |  |  | Amylase, xylanase and galactanase | *Bacteroides* | *Bacteroidota* | [271] |
|  |  |  | Xylanase and galactanase | *Bifidobacterium* | *Actinomycetota* | [271] |
|  |  | Intestine | Protease | *Actinomyces* | *Actinomycetota* | [360] |
|  |  |  | Protease | *Bacillus* | *Bacillota* | [360] |
|  |  |  | Protease | *Lactobacillus* | *Bacillota* | [360] |
|  |  |  | Protease | *Prevotella* | *Bacteroidota* | [360] |
|  |  |  | Protease | *Pseudomonas* | *Pseudomonadota* | [360] |
|  |  |  | Protease | Stenotrophomonas | *Pseudomonadota* | [360] |
|  |  | - | Amylase | *Desulfovibrio* | *Pseudomonadota* | [361] |
|  |  |  | Amylase | *Fusobacterium* | *Pseudomonadota* | [361] |
|  |  |  | Amylase | *Finegoldia* | *Bacillota* | [361] |
|  |  |  | Amylase | *Anaerobaculum* | *Synergistota* | [361] |
|  |  |  | Amylase and cellulase | *Elusimicrobium* | *Elusimicrobiota* | [361] |
|  |  |  | Amylase and cellulase | *Pediococcus* | *Bacteroidota* | [361] |
|  |  |  | Amylase and cellulase | *Porphyromonas* | *Bacteroidota* | [361] |
|  |  |  | Amylase and cellulase | *Bifidobacterium* | *Actinomycetota* | [361] |
|  |  |  | Amylase and cellulase | *Slackia* | *Actinomycetota* | [361] |
|  |  |  | Amylase and cellulase | *Brachyspira* | *Spirochaetota* | [361] |
|  |  |  | Amylase and cellulase | *Odoribacter* | *Bacteroidota* | [361] |
|  |  |  | Amylase and cellulase | *Listeria* | *Bacillota* | [361] |
|  |  |  | Amylase and cellulase | *Megamonas* | *Bacillota* | [361] |
|  |  |  | Amylase and cellulase | *Mitsuokella* | *Bacillota* | [361] |
|  |  |  | Amylase and cellulase | *Streptococcus* | *Bacillota* | [361] |
|  |  |  | Amylase and cellulase | *Veillonella* | *Bacillota* | [361] |
|  |  |  | Amylase and cellulase | *Escherichia* | *Pseudomonadota* | [361] |
|  |  |  | Amylase and cellulase | *Oxalobacter* | *Pseudomonadota* | [361] |
|  |  |  | Amylase and cellulase | *Providencia* | *Pseudomonadota* | [361] |
|  |  |  | Amylase and cellulase | *Xenorhabdus* | *Pseudomonadota* | [361] |
|  |  |  | Amylase and cellulase | *Acidaminococcus* | *Bacillota* | [361] |
|  |  |  | Amylase and cellulase | *Anaerostipes* | *Bacillota* | [361] |
|  |  |  | Amylase and cellulase | *Anaerotruncus* | *Bacillota* | [361] |
|  |  |  | Amylase and cellulase | *Bacillus* | *Bacillota* | [361] |
|  |  |  | Amylase and cellulase | *Blautia* | *Bacillota* | [361] |
|  |  |  | Amylase and cellulase | *Coprococcus* | *Bacillota* | [361] |
|  |  |  | Amylase and cellulase | *Dorea* | *Bacillota* | [361] |
|  |  |  | Amylase, cellulase, hyaluronate and lyase | *Catenibacterium* | *Bacillota* | [361] |
|  |  |  | Amylase, cellulase and pectinase | *Actinobacillus* | *Pseudomonadota* | [361] |
|  |  |  | Amylase, cellulase and pectinase | *Citrobacter* | *Pseudomonadota* | [361] |
|  |  |  | Amylase, cellulase and pectinase | *Enterobacter* | *Pseudomonadota* | [361] |
|  |  |  | Amylase, cellulase and pectinase | *Bryantella* | *Bacillota* | [361] |
|  |  |  | Amylase, cellulase and pectinase | *Eubacterium* | *Bacillota* | [361] |
|  |  |  | Amylase, cellulase and pectinase | *Faecalibacterium* | *Bacillota* | [361] |
|  |  |  | Amylase, cellulase and pectinase | *Subdoligranulum* | *Bacillota* | [361] |
|  |  |  | Amylase, cellulase and pectinase | *Collinsella* | *Actinomycetota* | [361] |
|  |  |  | Amylase, cellulase and pectinase | *Sebaldella* | *Fusobacteriota* | [361] |
|  |  |  | Amylase, cellulase and pectinase | *Akkermansia* | *Verrucomicrobiota* | [361] |
|  |  |  | Amylase, cellulase and pectinase | *Butyrivibrio* | *Bacillota* | [361] |
|  |  |  | Amylase, cellulase and pectinase | *Petrotoga* | *Thermotogota* | [361] |
|  |  |  | Amylase, cellulase, heparin lyase and pectinase | *Vibrio* | *Pseudomonadota* | [361] |
|  |  |  | Amylase, cellulase, heparin lyase and pectinase | *Yersinia* | *Pseudomonadota* | [361] |
|  |  |  | Amylase, cellulase, hyaluronate lyase and pectinase | *Edwardsiella* | *Pseudomonadota* | [361] |
|  |  |  | Amylase, cellulase, hyaluronate lyase and pectinase | *Klebsiella* | *Pseudomonadota* | [361] |
|  |  |  | Amylase, cellulase, hyaluronate lyase and pectinase | *Clostridium* | *Bacillota* | [361] |
|  |  |  | Amylase, cellulase, hyaluronate lyase and pectinase | *Enterococcus* | *Bacillota* | [361] |
|  |  |  | Amylase, cellulase, hyaluronate lyase and pectinase | *Lactobacillus* | *Bacillota* | [361] |
|  |  |  | Amylase, cellulase, hyaluronate lyase and pectinase | *Alistipes* | *Bacteroidota* | [361] |
|  |  |  | Amylase, cellulase, pectinase and xylanase | *Roseburia* | *Bacillota* | [361] |
|  |  |  | Amylase, cellulase, pectinase and xylanase | *Ruminococcus* | *Bacillota* | [361] |
|  |  |  | Amylase, cellulase, pectinase and xylanase | *Paraprevotella* | *Bacteroidota* | [361] |
|  |  |  | Amylase, cellulase, hyaluronate lyase, pectinase and xylanase | *Parabacteroides* | *Bacteroidota* | [361] |
|  |  |  | Amylase, cellulase, hyaluronate lyase, hyaluronate lyase, pectinase and xylanase | *Victivallis* | *Lentisphaerota* | [361] |
|  |  |  | Amylase, cellulase, hyaluronate lyase, hyaluronate lyase, pectinase and xylanase | *Bacteroides* | *Bacteroidota* | [361] |
|  |  |  | Cellulase | *Acinetobacter* | *Pseudomonadota* | [361] |
|  |  |  | Cellulase | *Laribacter* | *Pseudomonadota* | [361] |
|  |  |  | Hyaluronate lyase | *Helicobacter* | *Pseudomonadota* | [361] |
|  |  | - | Amylase and cellulase | *Eubacterium* | *Bacillota* | [362] |
|  |  |  | Amylase and cellulase | *Ruminococcus* | *Bacillota* | [362] |
|  |  |  | Amylase and cellulase | *Bifidobacterium* | *Actinomycetota* | [362] |
|  |  |  | Cellulase | *Clostridium* | *Bacillota* | [362] |
|  |  |  | Cellulase | *Roseburia* | *Bacillota* | [362] |
|  |  |  | Cellulase | *Bacteroides* | *Bacteroidota* | [362] |
|  |  | - | Protease | *Akkermansia* | *Verrucomicrobiota* | [363] |
|  |  | - | Protease | *Ruminococcus* | *Bacillota* | [364] |
|  |  |  | Protease | *Bifidobacterium* | *Actinomycetota* | [364] |
|  |  | - | Amylase | *Bifidobacterium* | *Actinomycetota* | [365] |
|  |  |  | Amylase | *Propionibacterium* | *Bacteroidota* | [365] |
|  |  |  | Amylase | *Fusobacterium* | *Pseudomonadota* | [365] |
|  |  |  | Amylase | *Eubacterium* | *Bacillota* | [365] |
|  |  |  | Amylase | *Clostridium* | *Bacillota* | [365] |
|  |  |  | Amylase | *Streptococcus* | *Bacillota* | [365] |
|  |  |  | Amylase | *Bacteroides* | *Bacteroidota* | [365] |
|  |  | - | Amylase | *Bifidobacterium* | *Actinomycetota* | [366] |
|  |  |  | Amylase | *Roseburia* | *Bacillota* | [366] |
|  |  |  | Amylase | *Ruminococcus* | *Bacillota* | [366] |
|  |  |  | Amylase | *Coprococcus* | *Bacillota* | [366] |
|  |  |  | Amylase | *Dorea* | *Bacillota* | [366] |
|  |  |  | Amylase and protease | *Bacteroides* | *Bacteroidota* | [366] |
|  |  |  | Amylase and protease | *Parabacteroides* | *Bacteroidota* | [366] |
|  |  |  | Amylase and protease | *Prevotella* | *Bacteroidota* | [366] |
|  |  |  | Amylase and protease | *Alistipes* | *Bacteroidota* | [366] |
|  |  |  | Amylase and protease | *Ruminiclostridium* | *Bacillota* | [366] |
|  |  |  | Protease | *Clostridium* | *Bacillota* | [366] |
|  |  |  | Protease | *Eubacterium* | *Bacillota* | [366] |
|  |  |  | Protease | *Erysipelatoclostridium* | *Bacillota* | [366] |
|  |  |  | Protease | *Blautia* | *Bacillota* | [366] |
|  |  |  | Protease | *Lachnoclostridium* | *Bacillota* | [366] |
|  |  |  | Protease | *Streptococcus* | *Bacillota* | [366] |
|  |  |  | Protease | *Veillonella* | *Bacillota* | [366] |
|  |  |  | Protease | *Escherichia* | *Pseudomonadota* | [366] |
|  |  | - | Amylase and protease | *Bifidobacterium* | *Actinomycetota* | [367] |
|  |  | - | Amylase, cellulase, pectinase, heparin lyase and xylanase | *Bacteroides* | *Bacteroidota* | [368] |

**Table S10.** List of the chemicals those involved in the review.

The details of the molecular information are provided in the xlsx S1 spreadsheet.

**References:**

1. Backman TWH, Cao Y, Girke T. Chemmine tools: An online service for analyzing and clustering small molecules. *Nucleic Acids Res*. 2011;**39**:W486-W91 <https://doi.org/10.1093/nar/gkr320>

2. Chen X, Reynolds CH. Performance of similarity measures in 2d fragment-based similarity searching:  Comparison of structural descriptors and similarity coefficients. *J Chem Inf Comput Sci*. 2002;**42**:1407-14 <https://doi.org/10.1021/ci025531g>

3. Bakke JE, Bergman ÅL, Larsen GL. Metabolism of 2,4′,5-trichlorobiphenyl by the mercapturic acid pathway. *Science*. 1982;**217**:645-47 <https://doi.org/10.1126/science.6806905>

4. Brandt I, Klasson-Wehler E, Rafter J *et al.* Metabolism of 2,4′,5-trichlorobiphenyl: Tissue concentrations of methylsulfonyl-2,4′,5-trichlorobiphenyl in germfree and conventional mice. *Toxicol Lett*. 1982;**12**:273-80 <https://doi.org/10.1016/0378-4274(82)90251-X>

5. Van de Wiele T, Vanhaecke L, Boeckaert C *et al.* Human colon microbiota transform polycyclic aromatic hydrocarbons to estrogenic metabolites. *Environ Health Perspect*. 2005;**113**:6-10 <https://doi.org/10.1289/ehp.7259>

6. Barker PS, Morrison FO, Whitaker RS. Conversion of ddt to ddd by proteus vulgaris, a bacterium isolated from the intestinal flora of a mouse. *Nature*. 1965;**205**:621-22 <https://doi.org/10.1038/205621b0>

7. Mendel JL, Walton MS. Conversion of p,p′-ddt to p,p′-ddd by intestinal flora of the rat. *Science*. 1966;**151**:1527-28 <https://doi.org/10.1126/science.151.3717.1527>

8. De S, Ghosh S, Dutta SKJIjom. Congener specific polychlorinated biphenyl metabolism by human intestinal microbe clostridium species: Comparison with human liver cell line-hepg2. 2006;**46**:199

9. Stein K, Portig J, Fuhrmann H *et al.* Steric factors in the pharmacokinetics of lindane and α-hexachlorocyclohexane in rats. *Xenobiotica*. 1980;**10**:65-77 <https://doi.org/10.3109/00498258009033732>

10. Yim Y-J, Seo J, Kang S-I *et al.* Reductive dechlorination of methoxychlor and ddt by human intestinal bacterium eubacterium limosum under anaerobic conditions. *Archives of Environmental Contamination* and *Toxicology*. 2008;**54**:406-11 <https://doi.org/10.1007/s00244-007-9044-y>

11. Bakke J, Struble C, Gustafsson JA *et al.* Catabolism of premercapturic acid pathway metabolites of naphthalene to naphthols and methylthio-containing metabolites in rats. *Proc Natl Acad Sci*. 1985;**82**:668-71 <https://doi.org/10.1073/pnas.82.3.668>

12. Mudziwapasi R, Mlambo SS, Chigu NL *et al.* Isolation and molecular characterization of bacteria from the gut of eisenia fetida for biodegradation of 4,4 ddt. *Journal of Applied Biology & Biotechnology*. 2016;**4**:41-47 <https://doi.org/10.7324/JABB.2016.40507>

13. Boush MG, Matsumura F. Insecticidal degradation by pseudomonas melophthora, the bacterial symbiote of the apple maggot1. *J Econ Entomol*. 1967;**60**:918-20 <https://doi.org/10.1093/jee/60.4.918>

14. Verma K, Agrawal N, Farooq M *et al.* Endosulfan degradation by a rhodococcus strain isolated from earthworm gut. *Ecotoxicology* and *Environmental Safety*. 2006;**64**:377-81 <https://doi.org/https://doi.org/10.1016/j.ecoenv.2005.05.014>

15. Verma A, Ali D, Farooq M *et al.* Expression and inducibility of endosulfan metabolizing gene in rhodococcus strain isolated from earthworm gut microflora for its application in bioremediation. *Bioresour Technol*. 2011;**102**:2979-84 <https://doi.org/10.1016/j.biortech.2010.10.005>

16. Harishankar MK, Sasikala C, Ramya M. Efficiency of the intestinal bacteria in the degradation of the toxic pesticide, chlorpyrifos. *3 Biotech*. 2013;**3**:137-42 <https://doi.org/10.1007/s13205-012-0078-0>

17. Hillenweck A, Cravedi J-P, Debrauwer L *et al.* Chlorothalonil biotransformation by gastrointestinal microflora:In vitrocomparative approach in rat, dog, and human. *Pestic Biochem Physiol*. 1997;**58**:34-48 <https://doi.org/10.1006/pest.1997.2282>

18. Bakke J, Gustafsson J-Å. Mercapturic acid pathway metabolites of xenobiotics: Generation of potentially toxic metabolites during enterohepatic circulation. *Trends Pharmacol Sci*. 1984;**5**:517-21 <https://doi.org/10.1016/0165-6147(84)90532-7>

19. Rafter JJ, Gustafsson JÅ, Bakke JE *et al.* Studies on the re-establishment of the intestinal microflora in germ-free rats with special reference to the metabolism of n-isopropyl-α-chloroacetanilide (propachlor). *Xenobiotica*. 1983;**13**:171-78 <https://doi.org/10.3109/00498258309052251>

20. Bakke JE, Gustafsson J-Å, Gustafsson BE. Metabolism of propachlor by the germfree rat. *Science*. 1980;**210**:433-35 <https://doi.org/10.1126/science.7433983>

21. Larsen GL, Bakke JE. Metabolism of mercapturic acid-pathway metabolites of 2-chloro-n-isopropylacetanilide (propachlor) by gastrointestinal bacteria. *Xenobiotica*. 1983;**13**:115-26 <https://doi.org/10.3109/00498258309052245>

22. Larsen GL, Bakke JE. Enterohepatic circulation in formation of propachlor (2-chloro-n-isopropylacetanilide) metabolites in the rat. *Xenobiotica*. 1981;**11**:473-80 <https://doi.org/10.3109/00498258109045857>

23. Bakke JE, Gustafsson Jå. Role of intestinal flora in metabolism of agrochemicals conjugated with glutathione. *Xenobiotica*. 1986;**16**:1047-56 <https://doi.org/10.3109/00498258609038982>

24. Elango D, Siddharthan N, Alaqeel SI *et al.* Biodegradation of neonicotinoid insecticide acetamiprid by earthworm gut bacteria brucella intermedium pdb13 and its ecotoxicity. *Microbiol Res*. 2023;**268**:127278 <https://doi.org/10.1016/j.micres.2022.127278>

25. Chen B, Zhang N, Xie S *et al.* Gut bacteria of the silkworm bombyx mori facilitate host resistance against the toxic effects of organophosphate insecticides. *Environ Int*. 2020;**143**:105886 <https://doi.org/10.1016/j.envint.2020.105886>

26. Wang D, Ren J, Tan Z *et al.* Gut microbial profiles in nereis succinea and their contribution to the degradation of organic pollutants. *Environ Sci Technol*. 2020;**54**:6235-43 <https://doi.org/10.1021/acs.est.9b07854>

27. Almeida LGd, Moraes LABd, Trigo JR *et al.* The gut microbiota of insecticide-resistant insects houses insecticide-degrading bacteria: A potential source for biotechnological exploitation. *PLoS One*. 2017;**12**:e0174754 <https://doi.org/10.1371/journal.pone.0174754>

28. Han L, Fang K, You X *et al.* Earthworms synergize with indigenous soil functional microorganisms to accelerate the preferential degradation of the highly toxic s-enantiomer of the fungicide imazalil in soil. *J Hazard Mater*. 2023;**457**:131778 <https://doi.org/10.1016/j.jhazmat.2023.131778>

29. Cheng D, Guo Z, Riegler M *et al.* Gut symbiont enhances insecticide resistance in a significant pest, the oriental fruit fly bactrocera dorsalis (hendel). *Microbiome*. 2017;**5**:13 <https://doi.org/10.1186/s40168-017-0236-z>

30. Philbert MA, Gray AJ, Connors TA. Preliminary investigations into the involvement of the intestinal microflora in cns toxicity induced by 1,3-dinitrobenzene in male f-344 rats. *Toxicol Lett*. 1987;**38**:307-14 <https://doi.org/10.1016/0378-4274(87)90013-0>

31. Rafil F, Franklin W, Heflich RH *et al.* Reduction of nitroaromatic compounds by anaerobic bacteria isolated from the human gastrointestinal tract. *Applied* and *Environmental Microbiology*. 1991;**57**:962-68 <https://doi.org/10.1128/aem.57.4.962-968.1991>

32. Rafii F, Cerniglia CE. Comparison of the azoreductase and nitroreductase from clostridium perfringens. *Applied* and *Environmental Microbiology*. 1993;**59**:1731-34 <https://doi.org/10.1128/aem.59.6.1731-1734.1993>

33. Kinouchi T, Ohnishi Y. Purification and characterization of 1-nitropyrene nitroreductases from bacteroides fragilis. *Applied* and *Environmental Microbiology*. 1983;**46**:596-604 <https://doi.org/10.1128/aem.46.3.596-604.1983>

34. Cerniglia CE, Howard PC, Fu PP *et al.* Metabolism of nitropolycyclic aromatic hydrocarbons by human intestinal microflora. *Biochemical* and *Biophysical Research Communications*. 1984;**123**:262-70 <https://doi.org/10.1016/0006-291X(84)90407-8>

35. Howard PC, Beland FA, Cerniglia CE. Reduction of the carcinogen 1-nitropyrene to 1-aminopyrene by rat intestinal bacteria. *Carcinogenesis*. 1983;**4**:985-90 <https://doi.org/10.1093/carcin/4.8.985>

36. El-Bayoumy K, Sharma C, Louis YM *et al.* The role of intestinal microflora in the metabolic reduction of 1-nitropyrene to 1-aminopyrene in conventional and germfree rats and in humans. *Cancer Lett*. 1983;**19**:311-16 <https://doi.org/10.1016/0304-3835(83)90100-3>

37. Ball LM, Rafter JJ, Gustafsson JÅ *et al.* Formation of mutagenic urinary metabolites from 1-nitropyrene in germ-free and conventional rats: Role of the gut flora. *Carcinogenesis*. 1991;**12**:1-5 <https://doi.org/10.1093/carcin/12.1.1>

38. Kim H-Y, Song H-G. Purification and characterization of nad(p)h-dependent nitroreductase i from klebsiella sp. C1 and enzymatic transformation of 2,4,6-trinitrotoluene. *Applied Microbiology* and *Biotechnology*. 2005;**68**:766-73 <https://doi.org/10.1007/s00253-005-1950-1>

39. Rickert DE, Butterworth BE, Popp JA *et al.* Dinitrotoluene: Acute toxicity, oncogenicity, genotoxicity, and metabolism. *CRC Crit Rev Toxicol*. 1984;**13**:217-34 <https://doi.org/10.3109/10408448409003373>

40. Rickert DE, Long RM, Krakowka S *et al.* Metabolism and excretion of 2,4-[14c]dinitrotoluene in conventional and axenic fischer-344 rats. *Toxicology* and *applied pharmacology*. 1981;**59**:574-79 <https://doi.org/10.1016/0041-008X(81)90312-4>

41. Guest D, Schnell SR, Rickert DE *et al.* Metabolism of 2,4-dinitrotoluene by intestinal microorganisms from rat, mouse, and man. *Toxicology* and *applied pharmacology*. 1982;**64**:160-68 <https://doi.org/10.1016/0041-008X(82)90335-0>

42. Humblot C, Combourieu B, Väisänen M-L *et al.* 1h nuclear magnetic resonance spectroscopy-based studies of the metabolism of food-borne carcinogen 2-amino-3-methylimidazo[4,5-f]quinoline by human intestinal microbiota. *Applied* and *Environmental Microbiology*. 2005;**71**:5116-23 <https://doi.org/10.1128/AEM.71.9.5116-5123.2005>

43. Hirayama K, Baranczewski P, Åkerlund J-E *et al.* Effects of human intestinal flora on mutagenicity of and DNA adduct formation from food and environmental mutagens. *Carcinogenesis*. 2000;**21**:2105-11 <https://doi.org/10.1093/carcin/21.11.2105>

44. Möller L, Corrie M, Midtvedt T *et al.* The role of the intestinal microflora in the formation of mutagenic metabolites from the carcinogenic air pollutant 2-nitrofluorene. *Carcinogenesis*. 1988;**9**:823-30 <https://doi.org/10.1093/carcin/9.5.823>

45. Richardson KE, Fu PP, Cerniglia CE. Metabolism of 1‐, 3‐, and 6‐nitrobenzo[a]pyrene by intestinal microflora. *J Toxicol Environ Health*. 1988;**23**:527-37 <https://doi.org/10.1080/15287398809531134>

46. Rafii F, Cerniglia CE. Reduction of azo dyes and nitroaromatic compounds by bacterial enzymes from the human intestinal tract. *Environ Health Perspect*. 1995;**103**:17-19 <https://doi.org/10.1289/ehp.95103s417>

47. Fu PP, Cerniglia CE, Richardson KE *et al.* Nitroreduction of 6-nitrobenzo[a]pyrene: A potential activation pathway in humans. *Mutation Research Letters*. 1988;**209**:123-29 <https://doi.org/https://doi.org/10.1016/0165-7992(88)90028-0>

48. Manning BW, Campbell WL, Franklin W *et al.* Metabolism of 6-nitrochrysene by intestinal microflora. *Applied* and *Environmental Microbiology*. 1988;**54**:197-203 <https://doi.org/10.1128/aem.54.1.197-203.1988>

49. Kinouchi T, Kataoka K, Miyanishi K *et al.* Role of intestinal microflora in metabolism of glutathione conjugates of 1-nitropyrene 4, 5-oxide and 1-nitropyrene 9, 10-oxide. *The Tohoku Journal of Experimental Medicine*. 1992;**168**:119-22 <https://doi.org/10.1620/tjem.168.119>

50. Kinouchi T, Kataoka K, Miyanishi K *et al.* Biological activities of the intestinal microflora in mice treated with antibiotics or untreated and the effects of the microflora on absorption and metabolic activation of orally administered glutathione conjugates of k-region epoxides of 1-nitropyrene. *Carcinogenesis*. 1993;**14**:869-74 <https://doi.org/10.1093/carcin/14.5.869>

51. Levin AA, Dent JG. Comparison of the metabolism of nitrobenzene by hepatic microsomes and cecal microflora from fischer-344 rats in vitro and the relative importance of each in vivo. *Drug Metab Disposition*. 1982;**10**:450 <https://doi.org/10.1016/S0090-9556(25)07912-7>

52. Scheline RR, Nygaard RT, Longberg B. Enzymatic reduction of the azo dye, acid yellow, by extracts of streptococcus faecalis isolated from rat intestine. *Food Cosmet Toxicol*. 1970;**8**:55-8 <https://doi.org/10.1016/s0015-6264(70)80223-1>

53. Chung KT, Fulk GE, Egan M. Reduction of azo dyes by intestinal anaerobes. *Clin Microbiol Rev*. 1978;**35**:588-62 <https://doi.org/10.1128/cmr.00039-07>

54. He Z, Chen L, Catalan-Dibene J *et al.* Food colorants metabolized by commensal bacteria promote colitis in mice with dysregulated expression of interleukin-23. *Cell Metabolism*. 2021;**33**:1358-71 <https://doi.org/10.1016/j.cmet.2021.04.015>

55. Dillon D, Combes R, Zeiger E. Activation by caecal reduction of the azo dye d &amp; c red no. 9 to a bacterial mutagen. *Mutagenesis*. 1994;**9**:295-99 <https://doi.org/10.1093/mutage/9.4.295>

56. Chen H, Wang R-F, Cerniglia CE. Molecular cloning, overexpression, purification, and characterization of an aerobic fmn-dependent azoreductase from enterococcus faecalis. *Protein Expression* and *Purification*. 2004;**34**:302-10 <https://doi.org/10.1016/j.pep.2003.12.016>

57. Zahran SA, Ali-Tammam M, Hashem AM *et al.* Azoreductase activity of dye-decolorizing bacteria isolated from the human gut microbiota. *Sci Rep*. 2019;**9**:5508 <https://doi.org/10.1038/s41598-019-41894-8>

58. Roxon JJ, Ryan AJ, Wright SE. Reduction of water-soluble azo dyes by intestinal bacteria. *Food Cosmet Toxicol*. 1967;**5**:367-9 <https://doi.org/10.1016/s0015-6264(67)83064-5>

59. Roxon JJ, Ryan AJ, Wright SE. Reduction of tartrazine by a proteus species isolated from rats. *Food Cosmet Toxicol*. 1966;**4**:419-26 <https://doi.org/10.1016/s0015-6264(66)80583-7>

60. Macholz R, Kujawa M, Schulze J *et al.* The metabolism of some xenobiotics in germ-free and conventional rats. *Arch Toxicol Suppl*. 1985;**8**:373-6 <https://doi.org/10.1007/978-3-642-69928-3_77>

61. Morrison JM, Wright CM, John GH. Identification, isolation and characterization of a novel azoreductase from clostridium perfringens. *Anaerobe*. 2012;**18**:229-34 <https://doi.org/10.1016/j.anaerobe.2011.12.006>

62. Rafii F, Franklin W, Cerniglia CE. Azoreductase activity of anaerobic bacteria isolated from human intestinal microflora. *Appl Environ Microbiol*. 1990;**56**:2146-51 <https://doi.org/10.1128/aem.56.7.2146-2151.1990>

63. Xu H, Heinze TM, Paine DD *et al.* Sudan azo dyes and para red degradation by prevalent bacteria of the human gastrointestinal tract. *Anaerobe*. 2010;**16**:114-19 <https://doi.org/10.1016/j.anaerobe.2009.06.007>

64. Brown JP. Reduction of polymeric azo and nitro dyes by intestinal bacteria. *Applied* and *Environmental Microbiology*. 1981;**41**:1283-86 <https://doi.org/10.1128/cmr.00039-07>

65. Xu H, Heinze Thomas M, Chen S *et al.* Anaerobic metabolism of 1-amino-2-naphthol-based azo dyes (sudan dyes) by human intestinal microflora. *Applied* and *Environmental Microbiology*. 2007;**73**:7759-62 <https://doi.org/10.1128/AEM.01410-07>

66. Smith GE, Griffiths LA. Metabolism of n-acylated and o-alkylated drugs by the intestinal microflora during anaerobic incubation in vitro. *Xenobiotica*. 1974;**4**:477-87 <https://doi.org/10.3109/00498257409052100>

67. Lee SC, Renwick AG. Sulfoxide reduction by rat intestinal flora and by escherichia coli in vitro. *Biochem Pharmacol*. 1995;**49**:1567-76 <https://doi.org/10.1016/0006-2952(95)00093-F>

68. Zheng X, Zhao A, Xie G *et al.* Melamine-induced renal toxicity is mediated by the gut microbiota. *Science Translational Medicine*. 2013;**5**:172ra22-72ra22 <https://doi.org/10.1126/scitranslmed.3005114>

69. Shelton DR, Karns JS, McCarty GW *et al.* Metabolism of melamine by klebsiella terragena. *Applied* and *Environmental Microbiology*. 1997;**63**: 2832-35 <https://doi.org/10.1128/cmr.00039-07>

70. Allison SJ. Gut microbes: A role in melamine-induced renal toxicity? *Nature Reviews Nephrology*. 2013;**9**:186-86 <https://doi.org/10.1038/nrneph.2013.28>

71. Hall LL, George SE, Kohan MJ *et al.* In vitro methylation of inorganic arsenic in mouse intestinal cecum. *Toxicology* and *applied pharmacology*. 1997;**147**:101-09 <https://doi.org/10.1006/taap.1997.8269>

72. Rowland IR, Davies MJ. In vitro metabolism of inorganic arsenic by the gastro-intestinal microflora of the rat. *J Appl Toxicol*. 1981;**1**:278-83 <https://doi.org/10.1002/jat.2550010508>

73. Wang H-Y, Chen S, Xue R-Y *et al.* Arsenic ingested early in life is more readily absorbed: Mechanistic insights from gut microbiota, gut metabolites, and intestinal morphology and functions. *Environ Sci Technol*. 2023;**57**:1017-27 <https://doi.org/10.1021/acs.est.2c04584>

74. Pinyayev TS, Kohan MJ, Herbin-Davis K *et al.* Preabsorptive metabolism of sodium arsenate by anaerobic microbiota of mouse cecum forms a variety of methylated and thiolated arsenicals. *Chem Res Toxicol*. 2011;**24**:475-77 <https://doi.org/10.1021/tx200040w>

75. Bu N, Wang HY, Hao WH *et al.* Generation of thioarsenicals is dependent on the enterohepatic circulation in rats. *Metallomics*. 2011;**3**:1064-73 <https://doi.org/10.1039/c1mt00036e>

76. Van de Wiele T, Gallawa Christina M, Kubachk Kevin M *et al.* Arsenic metabolism by human gut microbiota upon in vitro digestion of contaminated soils. *Environ Health Perspect*. 2010;**118**:1004-09 <https://doi.org/10.1289/ehp.0901794>

77. Diaz-Bone RA, Van de Wiele TR. Biovolatilization of metal(loid)s by intestinal microorganisms in the simulator of the human intestinal microbial ecosystem. *Environ Sci Technol*. 2009;**43**:5249-56 <https://doi.org/10.1021/es900544c>

78. Meyer J, Michalke K, Kouril T *et al.* Volatilisation of metals and metalloids: An inherent feature of methanoarchaea? *Systematic* and *Applied Microbiology*. 2008;**31**:81-87 <https://doi.org/10.1016/j.syapm.2008.02.001>

79. Yin N, Cai X, Zheng L *et al.* In vitro assessment of arsenic release and transformation from as(v)-sorbed goethite and jarosite: The influence of human gut microbiota. *Environ Sci Technol*. 2020;**54**:4432-42 <https://doi.org/10.1021/acs.est.9b07235>

80. Chi L, Xue J, Tu P *et al.* Gut microbiome disruption altered the biotransformation and liver toxicity of arsenic in mice. *Arch Toxicol*. 2019;**93**:25-35 <https://doi.org/10.1007/s00204-018-2332-7>

81. Yu H, Wu B, Zhang X-X *et al.* Arsenic metabolism and toxicity influenced by ferric iron in simulated gastrointestinal tract and the roles of gut microbiota. *Environ Sci Technol*. 2016;**50**:7189-97 <https://doi.org/10.1021/acs.est.6b01533>

82. Rubin SSCDC, Alava P, Zekker I *et al.* Arsenic thiolation and the role of sulfate-reducing bacteria from the human intestinal tract. *Environ Health Perspect*. 2014;**122**:817-22 <https://doi.org/10.1289/ehp.1307759>

83. Potera C. Fire in the belly? Sulfur-reducing gut microbes fuel arsenic thiolation. *Environ Health Perspect*. 2014;**122**:A222-A22 <https://doi.org/10.1289/ehp.122-A222>

84. Kuroda K, Yoshida K, Yoshimura M *et al.* Microbial metabolite of dimethylarsinic acid is highly toxic and genotoxic. *Toxicology* and *applied pharmacology*. 2004;**198**:345-53 <https://doi.org/10.1016/j.taap.2003.10.014>

85. Conklin SD, Ackerman AH, Fricke MW *et al.* In vitro biotransformation of an arsenosugar by mouse anaerobic cecal microflora and cecal tissue as examined using ic-icp-ms and lc-esi-ms/ms. *Analyst*. 2006;**131**:648-55 <https://doi.org/10.1039/B516275K>

86. Xue X-M, Wang H-Y, Yu X-W *et al.* Gut microbiota control the bioavailability and metabolism of organoarsenicals of seaweeds in mice after oral ingestion. *Environ Sci Technol*. 2023;**57**:8588-97 <https://doi.org/10.1021/acs.est.2c09167>

87. Zhou G-W, Yang X-R, Zheng F *et al.* Arsenic transformation mediated by gut microbiota affects the fecundity of caenorhabditis elegans. *Environ Pollut*. 2020;**260**:113991 <https://doi.org/10.1016/j.envpol.2020.113991>

88. Wang H-T, Zhu D, Li G *et al.* Effects of arsenic on gut microbiota and its biotransformation genes in earthworm metaphire sieboldi. *Environ Sci Technol*. 2019;**53**:3841-49 <https://doi.org/10.1021/acs.est.8b06695>

89. Song D, Chen L, Zhu S *et al.* Gut microbiota promote biotransformation and bioaccumulation of arsenic in tilapia. *Environ Pollut*. 2022;**305**:119321 <https://doi.org/10.1016/j.envpol.2022.119321>

90. Zhong X, Zhang G, Huang J *et al.* Effects of intestinal microbiota on the biological transformation of arsenic in zebrafish: Contribution and mechanism. *Environ Sci Technol*. 2024;**58**:2247-59 <https://doi.org/10.1021/acs.est.3c08010>

91. Michalke K, Schmidt A, Huber B *et al.* Role of intestinal microbiota in transformation of bismuth and other metals and metalloids into volatile methyl and hydride derivatives in humans and mice. *Applied* and *Environmental Microbiology*. 2008;**74**:3069-75 <https://doi.org/10.1128/AEM.02933-07>

92. Topcu A, Bulat T. Removal of cadmium and lead from aqueous solution by enterococcus faecium strains. *J Food Sci*. 2010;**75**:T13-T17 <https://doi.org/10.1111/j.1750-3841.2009.01429.x>

93. George F, Mahieux S, Daniel C *et al.* Assessment of pb(ii), cd(ii), and al(iii) removal capacity of bacteria from food and gut ecological niches: Insights into biodiversity to limit intestinal biodisponibility of toxic metals. *Microorganisms*.

94. Biswas JK, Banerjee A, Rai MK *et al.* Exploring potential applications of a novel extracellular polymeric substance synthesizing bacterium (bacillus licheniformis) isolated from gut contents of earthworm (metaphire posthuma) in environmental remediation. *Biodegradation*. 2018;**29**:323-37 <https://doi.org/10.1007/s10532-018-9835-z>

95. Kaschak E, Knopf B, Petersen JH *et al.* Biotic methylation of mercury by intestinal and sulfate-reducing bacteria and their potential role in mercury accumulation in the tissue of the soil-living eisenia foetida. *Soil Biol Biochem*. 2014;**69**:202-11 <https://doi.org/10.1016/j.soilbio.2013.11.004>

96. Rowland IR, Grasso P, Davies MJ. The methylation of mercuric chloride by human intestinal bacteria. *Experientia*. 1975;**31**:1064-65 <https://doi.org/10.1007/BF02326961>

97. Edwards T, McBride BC. Biosynthesis and degradation of methylmercury in human faeces. *Nature*. 1975;**253**:462-64 <https://doi.org/10.1038/253462a0>

98. Ludwicki JK. Studies on the role of gastrointestinal tract contents in the methylation of inorganic mercury compounds. *Bulletin of environmental contamination* and *toxicology*. 1989;**42**:283-88 <https://doi.org/10.1007/BF01699412>

99. Rowland IR, Davies MJ, Grasso P. Metabolism of methylmercuric chloride by the gastro-intestinal flora of the rat. *Xenobiotica*. 1978;**8**:37-43 <https://doi.org/10.3109/00498257809060381>

100. Rowland IR, Davies MJ, Evans JG. Tissue content of mercury in rats given methylmercuric chloride orally: Influence of intestinal flora. *Archives of Environmental Health: An International Journal*. 1980;**35**:155-60 <https://doi.org/10.1080/00039896.1980.10667485>

101. Nakamura I, Hosokawa K, Tamura H *et al.* Reduced mercury excretion with feces in germfree mice after oral administration of methyl mercury chloride. *Bulletin of environmental contamination* and *toxicology*. 1977;**17**:528-33 <https://doi.org/10.1007/BF01685974>

102. Seko Y, Miura T, Takahashi M *et al.* Methyl mercury decomposition in mice treated with antibiotics. *Acta Pharmacol Toxicol (Copenh)*. 1981;**49**:259-65 <https://doi.org/10.1111/j.1600-0773.1981.tb00903.x>

103. Ludwicki JK. In vitro methylation and demethylation of mercury compounds by the intestinal contents. *Bull Environ Contam Toxicol*. 1990;**44**:357-62 <https://doi.org/10.1007/bf01701215>

104. Rudd JW, Furutani A, Turner MA. Mercury methylation by fish intestinal contents. *Applied* and *Environmental Microbiology*. 1980;**40**:777-82 <https://doi.org/10.1128/aem.40.4.777-782.1980>

105. Yang T-T, Liu Y, Tan S *et al.* The role of intestinal microbiota of the marine fish (acanthopagrus latus) in mercury biotransformation. *Environ Pollut*. 2021;**277**:116768 <https://doi.org/10.1016/j.envpol.2021.116768>

106. Pan-Hou HSK, Imura N. Biotransformation of mercurials by intestinal microorganisms isolated from yellowfin tuna. *Bull Environ Contam Toxicol*. 1981;**26**:359-63 <https://doi.org/10.1007/bf01622102>

107. Wang X, Wu F, Wang W-X. In vivo mercury demethylation in a marine fish (acanthopagrus schlegeli). *Environ Sci Technol*. 2017;**51**:6441-51 <https://doi.org/10.1021/acs.est.7b00923>

108. Kozak S, Forsberg CW. Transformation of mercuric chloride and methylmercury by the rumen microflora. *Applied* and *Environmental Microbiology*. 1979;**38**:626-36 <https://doi.org/10.1128/aem.38.4.626-636.1979>

109. Deloménie C, Fouix S, Longuemaux S *et al.* Identification and functional characterization of arylamine n-acetyltransferases in eubacteria: Evidence for highly selective acetylation of 5-aminosalicylic acid. *J Bacteriol*. 2001;**183**:3417-27 <https://doi.org/10.1128/jb.183.11.3417-3427.2001>

110. Vermes A, Kuijper EJ, Guchelaar H-J *et al.* An in vitro study on the active conversion of flucytosine to fluorouracil by microorganisms in the human intestinal microflora. *Chemotherapy*. 2003;**49**:17-23 <https://doi.org/10.1159/000069784>

111. Harris BE, Manning BW, Federle TW *et al.* Conversion of 5-fluorocytosine to 5-fluorouracil by human intestinal microflora. *Antimicrobial Agents* and *Chemotherapy*. 1986;**29**:44-48 <https://doi.org/10.1128/aac.29.1.44>

112. Hidese R, Mihara H, Kurihara T *et al.* Escherichia coli dihydropyrimidine dehydrogenase is a novel nad-dependent heterotetramer essential for the production of 5,6-dihydrouracil. *J Bacteriol*. 2011;**193**:989-93 <https://doi.org/10.1128/cmr.00039-07>

113. Kunihiro Y, Hirotoshi M, Eiji M *et al.* Tissue distribution and biotransformation of potassium oxonate after oral administration of a novel antitumor agent (drug combination of tegafur, 5-chloro-2,4-dihydroxypyridine, and potassium oxonate) to rats. *Drug Metabolism* and *Disposition*. 2000;**28**:1162

114. Lee SH, An JH, Lee HJ *et al.* Evaluation of pharmacokinetic differences of acetaminophen in pseudo germ-free rats. *Biopharm Drug Disposition*. 2012;**33**:292-303 <https://doi.org/10.1002/bdd.1799>

115. Mikov M, Caldwell J, Dolphin CT *et al.* The role of intestinal microflora in the formation of the methylthio adduct metabolites of paracetamol: Studies in neomycin-pretreated and germ-free mice. *Biochem Pharmacol*. 1988;**37**:1445-49 <https://doi.org/10.1016/0006-2952(88)90005-6>

116. Clayton TA, Baker D, Lindon JC *et al.* Pharmacometabonomic identification of a significant host-microbiome metabolic interaction affecting human drug metabolism. *Proc Natl Acad Sci*. 2009;**106**:14728-33 <https://doi.org/10.1073/pnas.0904489106>

117. Zimmermann M, Zimmermann-Kogadeeva M, Wegmann R *et al.* Mapping human microbiome drug metabolism by gut bacteria and their genes. *Nature*. 2019;**570**:462-67 <https://doi.org/10.1038/s41586-019-1291-3>

118. Sasaki I, Tamura T, Shibakawa T *et al.* Metabolism of azetirelin, a new thyrotropin-releasing hormone (trh) analogue, by intestinal microorganisms. *Pharm Res*. 1997;**14**:1004-07 <https://doi.org/10.1023/A:1012141025938>

119. Liu T, Jiang X. Investigation of the absorption mechanisms of baicalin and baicalein in rats. *Journal of Pharmaceutical Sciences*. 2006;**95**:1326-33 <https://doi.org/https://doi.org/10.1002/jps.20593>

120. Jung M-A, Jang S-E, Hong S-W *et al.* The role of intestinal microflora in anti-inflammatory effect of baicalin in mice. *Biomol Ther (Seoul)*. 2012;**20**:36-42 <https://doi.org/10.4062/biomolther.2012.20.1.036>

121. Zhang L, Lin G, Zuo Z. Involvement of udp-glucuronosyltransferases in the extensive liver and intestinal first-pass metabolism of flavonoid baicalein. *Pharm Res*. 2007;**24**:81-89 <https://doi.org/10.1007/s11095-006-9126-y>

122. Chan RP, Pope DJ, Gilbert AP *et al.* Studies of two novel sulfasalazine analogs, ipsalazide and balsalazide. *Digestive Diseases* and *Sciences*. 1983;**28**:609-15 <https://doi.org/10.1007/BF01299921>

123. Sousa T, Yadav V, Zann V *et al.* On the colonic bacterial metabolism of azo-bonded prodrugsof 5-aminosalicylic acid. *Journal of Pharmaceutical Sciences*. 2014;**103**:3171-75 <https://doi.org/10.1002/jps.24103>

124. Hennessey TD. Inducible β-lactamase in enterobacter. *Microbiology*. 1967;**49**:277-85 <https://doi.org/10.1099/00221287-49-2-277>

125. Feng R, Shou J-W, Zhao Z-X *et al.* Transforming berberine into its intestine-absorbable form by the gut microbiota. *Sci Rep*. 2015;**5**:12155 <https://doi.org/10.1038/srep12155>

126. Yongmei L, Jun X, Lai WG *et al.* Metabolic switching of bilr 355 in the presence of ritonavir. Ii. Uncovering novel contributions by gut bacteria and aldehyde oxidase. *Drug Metab Disposition*. 2012;**40**:1130 <https://doi.org/10.1124/dmd.111.044362>

127. Zimmermann M, Zimmermann-Kogadeeva M, Wegmann R *et al.* Separating host and microbiome contributions to drug pharmacokinetics and toxicity. *Science*. 2019;**363**:eaat9931 <https://doi.org/10.1126/science.aat9931>

128. Wang J, Yadav V, Smart AL *et al.* Stability of peptide drugs in the colon. *Eur J Pharm Sci*. 2015;**78**:31-36 <https://doi.org/10.1016/j.ejps.2015.06.018>

129. Tozaki H, Emi Y, Horisaka ERI *et al.* Degradation of insulin and calcitonin and their protection by various protease inhibitors in rat caecal contents: Implications in peptide delivery to the colon. *Journal of Pharmacy* and *Pharmacology*. 1997;**49**:164-68 <https://doi.org/10.1111/j.2042-7158.1997.tb06773.x>

130. Javdan B, Lopez JG, Chankhamjon P *et al.* Personalized mapping of drug metabolism by the human gut microbiome. *Cell*. 2020;**181**:1661-79.e22 <https://doi.org/10.1016/j.cell.2020.05.001>

131. Iveson P, Lindup WE, Parke DV *et al.* The metabolism of carbenoxolone in the rat. *Xenobiotica*. 1971;**1**:79-95 <https://doi.org/10.3109/00498257109044381>

132. Holt R. The bacterial degradation of chloramphenicol. *The Lancet*. 1967;**289**:1259-60 <https://doi.org/10.1016/S0140-6736(67)92720-1>

133. LinWu S-W, Syu C-J, Chen Y-L *et al.* Characterization of escherichia coli nitroreductase nfsb in the metabolism of nitrobenzodiazepines. *Biochem Pharmacol*. 2009;**78**:96-103 <https://doi.org/10.1016/j.bcp.2009.03.019>

134. Elmer GW, Remmel RP. Role of the intestinal microflora in clonazepam metabolism in the rat. *Xenobiotica*. 1984;**14**:829-40 <https://doi.org/10.3109/00498258409151481>

135. Michelle M, Rucha SS, Monica K-L *et al.* Defining the role of gut bacteria in the metabolism of deleobuvir: In vitro and in vivo studies. *Drug Metabolism* and *Disposition*. 2015;**43**:1612 <https://doi.org/10.1124/dmd.115.064477>

136. Amanda L, Bret DW, Lauren B *et al.* Pharmacologic targeting of bacterial β-glucuronidase alleviates nonsteroidal anti-inflammatory drug-induced enteropathy in mice. *Journal of Pharmacology* and *Experimental Therapeutics*. 2012;**341**:447 <https://doi.org/10.1124/jpet.111.191122>

137. LoGuidice A, Wallace BD, Bendel L *et al.* Pharmacologic targeting of bacterial β-glucuronidase alleviates nonsteroidal anti-inflammatory drug-induced enteropathy in mice. *Journal of Pharmacology* and *Experimental Therapeutics*. 2012;**341**:447-54 <https://doi.org/10.1124/jpet.111.191122>

138. Dobkin JF, Saha JR, Butler VP *et al.* Digoxin-inactivating bacteria: Identification in human gut flora. *Science*. 1983;**220**:325-27 <https://doi.org/10.1126/science.6836275>

139. Haiser HJ, Gootenberg DB, Chatman K *et al.* Predicting and manipulating cardiac drug inactivation by the human gut bacterium eggerthella lenta. *Science*. 2013;**341**:295-98 <https://doi.org/10.1126/science.1235872>

140. Lindenbaum J, Rund DG, Butler VP *et al.* Inactivation of digoxin by the gut flora: Reversal by antibiotic therapy. *New Engl*and *Journal of Medicine*. 1981;**305**:789-94 <https://doi.org/10.1056/NEJM198110013051403>

141. Mathan VI, Wiederman J, Dobkin JF *et al.* Geographic differences in digoxin inactivation, a metabolic activity of the human anaerobic gut flora. *Gut*. 1989;**30**:971 <https://doi.org/10.1136/gut.30.7.971>

142. Yanli D, Martin R, Caroline S *et al.* Investigations of hydrazine cleavage of eltrombopag in humans. *Drug Metab Disposition*. 2011;**39**:1747 <https://doi.org/10.1124/dmd.111.040188>

143. Hattori M, Sakamoto T, Kobashi K *et al.* Metabolism of glycyrrhizin by human intestinal flora. *Planta Med*. 1983;**48**:38-42 <https://doi.org/10.1055/s-2007-969875>

144. Saitta KS, Zhang C, Lee KK *et al.* Bacterial β-glucuronidase inhibition protects mice against enteropathy induced by indomethacin, ketoprofen or diclofenac: Mode of action and pharmacokinetics. *Xenobiotica*. 2014;**44**:28-35 <https://doi.org/10.3109/00498254.2013.811314>

145. Tobin PJ, Dodds HM, Clarke S *et al.* The relative contributions of carboxylesterase and β-glucuronidase in the formation of sn-38 in human colorectal tumours. *Oncol Rep*. 2003;**10**:1977-79 <https://doi.org/10.3892/or.10.6.1977>

146. Goldin BR, Peppercorn MA, Goldman P. Contributions of host and intesitnal microflora in the metabolism of l-dopa by the rat. *Journal of Pharmacology* and *Experimental Therapeutics*. 1973;**186**:160

147. Sandler M, Goodwin BL, Ruthven CRJ *et al.* Therapeutic implications in parkinsonism of m-tyramine formation from l-dopa in man. *Nature*. 1971;**229**:414-16 <https://doi.org/10.1038/229414a0>

148. Sandler M, Karoum F, Ruthven CRJ *et al.* M-hydroxyphenylacetic acid formation from l-dopa in man: Suppression by neomycin. *Science*. 1969;**166**:1417-18 <https://doi.org/10.1126/science.166.3911.1417>

149. Bergmark J, Carlsson A, Granerus A-K *et al.* Decarboxylation of orally administered l-dopa in the human digestive tract. *Naunyn-Schmiedeberg's Arch Pharmacol*. 1972;**272**:437-40 <https://doi.org/10.1007/BF00501249>

150. Calne DB, Reid JL, Vakil SD *et al.* Idiopathic parkinsonism treated with an extracerebral decarboxylase inhibitor in combination with levodopa. *Br Med J*. 1971;**3**:729-32 <https://doi.org/10.1136/bmj.3.5777.729>

151. Hashim H, Azmin S, Razlan H *et al.* Eradication of helicobacter pylori infection improves levodopa action, clinical symptoms and quality of life in patients with parkinson's disease. *PLoS One*. 2014;**9**:e112330 <https://doi.org/10.1371/journal.pone.0112330>

152. Shu YZ, Kingston DGI, Van Tassell RL *et al.* Metabolism of levamisole, an anti-colon cancer drug, by human intestinal bacteria. *Xenobiotica*. 1991;**21**:737-50 <https://doi.org/10.3109/00498259109039513>

153. Lavrijsen K, Van Dyck D, Van Houdt J *et al.* Reduction of the prodrug loperamide oxide to its active drug loperamide in the gut of rats, dogs, and humans. *Drug Metab Disposition*. 1995;**23**:354-62 <https://doi.org/10.1002/ddr.430340309>

154. Wallace BD, Wang H, Lane KT *et al.* Alleviating cancer drug toxicity by inhibiting a bacterial enzyme. *Science*. 2010;**330**:831-35 <https://doi.org/10.1126/science.1191175>

155. Dae-Hyoung Y, In Sook K, Thi Kim Van L *et al.* Gut microbiota-mediated drug interactions between lovastatin and antibiotics. *Drug Metab Disposition*. 2014;**42**:1508 <https://doi.org/10.1124/dmd.114.058354>

156. Caldwell J, Hawksworth GM. The demethylation of methamphetamine by intestinal microflora. *Journal of Pharmacy* and *Pharmacology*. 1973;**25**:422-24 <https://doi.org/10.1111/j.2042-7158.1973.tb10043.x>

157. Valerino DM, Johns DG, Zaharko DS *et al.* Studies of the metabolism of methotrexate by intestinal flora—i: Identification and study of biological properties of the metabolite 4-amino-4-deoxy-n10-methylpteroic acid. *Biochem Pharmacol*. 1972;**21**:821-31 <https://doi.org/10.1016/0006-2952(72)90125-6>

158. Koch RL, Chrystal EJT, Beaulieu BB *et al.* Acetamide—a metabolite of metronidazole formed by the intestinal flora. *Biochem Pharmacol*. 1979;**28**:3611-15 <https://doi.org/10.1016/0006-2952(79)90407-6>

159. Rafii F, Wynne R, Heinze TM *et al.* Mechanism of metronidazole-resistance by isolates of nitroreductase-producing enterococcus gallinarum and enterococcus casseliflavus from the human intestinal tract. *FEMS Microbiol Lett*. 2003;**225**:195-200 <https://doi.org/10.1016/S0378-1097(03)00513-5>

160. Koch RL, Beaulieu BB, Goldman P. Role of the intestinal flora in the metabolism of misonidazole. *Biochem Pharmacol*. 1980;**29**:3281-84 <https://doi.org/10.1016/0006-2952(80)90304-4>

161. Walsh CT, Levine RR. Studies of the enterohepatic circulation of morphine in the rat. *Journal of Pharmacology* and *Experimental Therapeutics*. 1975;**195**:303-10 <https://doi.org/10.1016/S0022-3565(25)30342-3>

162. Williams JR, Grantham PH, Marsh HH *et al.* Participation of liver fractions and of intestinal bacteria in the metabolism of n-hydroxy-n-2-fluorenylacetamide in the rat. *Biochem Pharmacol*. 1970;**19**:173-88 <https://doi.org/10.1016/0006-2952(70)90338-2>

163. Selma MV, Espín JC, Tomás-Barberán FA. Interaction between phenolics and gut microbiota: Role in human health. *Journal of Agricultural* and *Food Chemistry*. 2009;**57**:6485-501 <https://doi.org/10.1021/jf902107d>

164. Schoefer L, Mohan R, Schwiertz A *et al.* Anaerobic degradation of flavonoids by clostridium orbiscindens. *Applied* and *Environmental Microbiology*. 2003;**69**:5849-54 <https://doi.org/10.1128/AEM.69.10.5849-5854.2003>

165. Schneider H, Blaut M. Anaerobic degradation of flavonoids by eubacterium ramulus. *Arch Microbiol*. 2000;**173**:71-75 <https://doi.org/10.1007/s002030050010>

166. Gingell R, Bridges JW, Williams RT. The role of the gut flora in the metabolism of prontosil and neoprontosil in the rat. *Xenobiotica*. 1971;**1**:143-56 <https://doi.org/10.3109/00498257109044386>

167. Beckett AH, Gorrod JW, Jenner P. Absorption of (−)-nicotine-1′-n-oxide in man and its reduction in the gastrointestinal tract. *Journal of Pharmacy* and *Pharmacology*. 1970;**22**:722-23 <https://doi.org/10.1111/j.2042-7158.1970.tb12767.x>

168. Rafii F, Sutherland J, Hansen Jr E *et al.* Reduction of nitrazepam by clostridium leptum, a nitroreductase-producing bacterium isolated from the human intestinal tract. *Clinical Infectious Diseases-Supplements*. 1997;**25**:S121-S22 <https://doi.org/10.1086/516204>

169. Takeno S, Hirano Y, Kitamura A *et al.* Comparative developmental toxicity and metabolism of nitrazepam in rats and mice. *Toxicology* and *applied pharmacology*. 1993;**121**:233-38 <https://doi.org/10.1006/taap.1993.1150>

170. Takeno S, Sakai T. Involvement of the intestinal microflora in nitrazepam-induced teratogenicity in rats and its relationship to nitroreduction. *Teratology*. 1991;**44**:209-14 <https://doi.org/10.1002/tera.1420440209>

171. Rafii F, Hansen Eugene B. Isolation of nitrofurantoin-resistant mutants of nitroreductase-producing clostridium sp. Strains from the human intestinal tract. *Antimicrobial Agents* and *Chemotherapy*. 1998;**42**:1121-26 <https://doi.org/10.1128/aac.42.5.1121>

172. Basit AW, Newton JM, Lacey LF. Susceptibility of the h2-receptor antagonists cimetidine, famotidine and nizatidine, to metabolism by the gastrointestinal microflora. *Int J Pharm*. 2002;**237**:23-33 <https://doi.org/10.1016/S0378-5173(02)00018-2>

173. Ye W, Xing J, Yu Z *et al.* Mechanism and treatments of antipsychotic-induced weight gain. *International Journal of Obesity*. 2023;**47**:423-33 <https://doi.org/10.1038/s41366-023-01291-8>

174. Wadworth AN, Fitton A. Olsalazine. *Drugs*. 1991;**41**:647-64 <https://doi.org/10.2165/00003495-199141040-00009>

175. Knoll U, Strauhs P, Schusser G *et al.* Study of the plasma pharmacokinetics and faecal excretion of the prodrug olsalazine and its metabolites after oral administration to horses. *J Vet Pharmacol Ther*. 2002;**25**:135-43 <https://doi.org/10.1046/j.1365-2885.2002.00395.x>

176. Watanabe K, Yamashita S, Furuno K *et al.* Metabolism of omeprazole by gut flora in rats. *J Pharm Sci*. 1995;**84**:516-17 <https://doi.org/10.1002/jps.2600840425>

177. Schneider H, Simmering R, Hartmann L *et al.* Degradation of quercetin-3-glucoside in gnotobiotic rats associated with human intestinal bacteria. *J Appl Microbiol*. 2000;**89**:1027-37 <https://doi.org/10.1046/j.1365-2672.2000.01209.x>

178. Xue C, Jiang S, Guo J *et al.* Screening for in vitro metabolites of abelmoschus manihot extract in intestinal bacteria by ultra-performance liquid chromatography/quadrupole time-of-flight mass spectrometry. *J Chromatogr B*. 2011;**879**:3901-08 <https://doi.org/10.1016/j.jchromb.2011.10.043>

179. Day AJ, DuPont MS, Ridley S *et al.* Deglycosylation of flavonoid and isoflavonoid glycosides by human small intestine and liver β-glucosidase activity. *FEBS Lett*. 1998;**436**:71-75 <https://doi.org/10.1016/S0014-5793(98)01101-6>

180. Basit AW, Lacey LF. Colonic metabolism of ranitidine: Implications for its delivery and absorption. *Int J Pharm*. 2001;**227**:157-65 <https://doi.org/10.1016/S0378-5173(01)00794-3>

181. Aura A-M, Mattila I, Hyötyläinen T *et al.* Drug metabolome of the simvastatin formed by human intestinal microbiota in vitro. *Molecular BioSystems*. 2011;**7**:437-46 <https://doi.org/10.1039/C0MB00023J>

182. Parvez MM, Basit A, Jariwala PB *et al.* Quantitative investigation of irinotecan metabolism, transport, and gut microbiome activation. *Drug Metab Disposition*. 2021;**49**:683-93 <https://doi.org/10.1124/dmd.121.000476>

183. Stringer AM, Gibson RJ, Logan RM *et al.* Faecal microflora and β-glucuronidase expression are altered in an irinotecan-induced diarrhea model in rats. *Cancer Biology & Therapy*. 2008;**7**:1919-25 <https://doi.org/10.4161/cbt.7.12.6940>

184. Hoy SM, Scott LJ, Wagstaff AJ. Sodium picosulfate/magnesium citrate. *Drugs*. 2009;**69**:123-36 <https://doi.org/10.2165/00003495-200969010-00009>

185. Okuda H, Ogura K, Kato A *et al.* A possible mechanism of eighteen patient deaths caused by interactions of sorivudine, a new antiviral drug, with oral 5-fluorouracil prodrugs. *Journal of Pharmacology* and *Experimental Therapeutics*. 1998;**287**:791

186. Haruyuki N, Takemi K, Keiko K *et al.* Intestinal anaerobic bacteria hydrolyse sorivudine, producing the high blood concentration of 5-(e)-(2-bromovinyl)uracil that increases the level and toxicity of 5-fluorouracil. *Pharmacogenetics*. 1997;**7**:35-43 <https://doi.org/10.1097/00008571-199702000-00005>

187. Scheline RR. The metabolism of drugs and other organic compounds by the intestinal microflora. *Acta Pharmacol Toxicol (Copenh)*. 1968;**26**:332-42 <https://doi.org/10.1111/j.1600-0773.1968.tb00453.x>

188. Dull BJ, Salata K, Goldman P. Role of the intestinal flora in the acetylation of sulfasalazine metabolites. *Biochem Pharmacol*. 1987;**36**:3772-74 <https://doi.org/10.1016/0006-2952(87)90034-7>

189. Peppercorn MA, Goldman P. The role of intestinal bacteria in the metabolism of salicylazosulfapyridine. *Journal of Pharmacology* and *Experimental Therapeutics*. 1972;**181**:555-62 <https://doi.org/10.1016/S0022-3565(25)29238-2>

190. Khan AKA, Guthrie G, Johnston HH *et al.* Tissue and bacterial splitting of sulfasalazine. *Clin Sci*. 1983;**64**:349-54 <https://doi.org/10.1042/cs0640349>

191. Strong HA, Renwick AG, George CF *et al.* The reduction of sulfinpyrazone and sulindac by intestinal bacteria. *Xenobiotica*. 1987;**17**:685-96 <https://doi.org/10.3109/00498258709043976>

192. Strong HA, Oates J, Sembi J *et al.* Role of the gut flora in the reduction of sulfinpyrazone in humans. *Journal of Pharmacology* and *Experimental Therapeutics*. 1984;**230**:726-32 <https://doi.org/10.1016/S0022-3565(25)21746-3>

193. Renwick AG, Evans SP, Sweatman TW *et al.* The role of the gut flora in the reduction of sulfinpyrazone in the rat. *Biochem Pharmacol*. 1982;**31**:2649-56 <https://doi.org/10.1016/0006-2952(82)90713-4>

194. Strong HA, Warner NJ, Renwick AG *et al.* Sulindac metabolism: The importance of an intact colon. *Clin Pharmacol Ther*. 1985;**38**:387-93 <https://doi.org/10.1038/clpt.1985.192>

195. Kitamura S, Sugihara K, Kuwasako M *et al.* The role of mammalian intestinal bacteria in the reductive metabolism of zonisamide. *Journal of Pharmacy* and *Pharmacology*. 1997;**49**:253-56 <https://doi.org/10.1111/j.2042-7158.1997.tb06790.x>

196. Khanal T, Kim HG, Jin SW *et al.* Protective role of metabolism by intestinal microflora in butyl paraben-induced toxicity in hepg2 cell cultures. *Toxicol Lett*. 2012;**213**:174-83 <https://doi.org/10.1016/j.toxlet.2012.07.004>

197. Wang G, Zhang H, Zhang J *et al.* Metabolic fate of environmental chemical triclocarban in colon tissues: Roles of gut microbiota involved. *Sci Total Environ*. 2021;**787**:147677 <https://doi.org/10.1016/j.scitotenv.2021.147677>

198. Weitekamp CA, Phelps D, Swank A *et al.* Triclosan-selected host-associated microbiota perform xenobiotic biotransformations in larval zebrafish. *Toxicol Sci*. 2019;**172**:109-22 <https://doi.org/10.1093/toxsci/kfz166>

199. Gratz Silvia W, Duncan G, Richardson Anthony J. The human fecal microbiota metabolizes deoxynivalenol and deoxynivalenol-3-glucoside and may be responsible for urinary deepoxy-deoxynivalenol. *Applied* and *Environmental Microbiology*. 2013;**79**:1821-25 <https://doi.org/10.1128/AEM.02987-12>

200. Berthiller F, Krska R, Domig KJ *et al.* Hydrolytic fate of deoxynivalenol-3-glucoside during digestion. *Toxicol Lett*. 2011;**206**:264-67 <https://doi.org/10.1016/j.toxlet.2011.08.006>

201. Daud N, Currie V, Duncan G *et al.* Prevalent human gut bacteria hydrolyse and metabolise important food-derived mycotoxins and masked mycotoxins. *Toxins*.

202. Gratz SW, Dinesh R, Yoshinari T *et al.* Masked trichothecene and zearalenone mycotoxins withstand digestion and absorption in the upper gi tract but are efficiently hydrolyzed by human gut microbiota in vitro. *Mol Nutr Food Res*. 2017;**61**:1600680 <https://doi.org/10.1002/mnfr.201600680>

203. Dall’Erta A, Cirlini M, Dall’Asta M *et al.* Masked mycotoxins are efficiently hydrolyzed by human colonic microbiota releasing their aglycones. *Chem Res Toxicol*. 2013;**26**:305-12 <https://doi.org/10.1021/tx300438c>

204. Madhyastha MS, Marquardt RR, Frohlich AA. Hydrolysis of ochratoxin a by the microbial activity of digesta in the gastrointestinal tract of rats. *Archives of Environmental Contamination* and *Toxicology*. 1992;**23**:468-72 <https://doi.org/10.1007/BF00203811>

205. Young JC, Zhou T, Yu H *et al.* Degradation of trichothecene mycotoxins by chicken intestinal microbes. *Food Chem Toxicol*. 2007;**45**:136-43 <https://doi.org/10.1016/j.fct.2006.07.028>

206. Eriksen GS, Pettersson H, Johnsen K *et al.* Transformation of trichothecenes in ileal digesta and faeces from pigs. *Archiv für Tierernaehrung*. 2002;**56**:263-74 <https://doi.org/10.1080/00039420214343>

207. Gao X, Ma Q, Zhao L *et al.* Isolation of bacillus subtilis: Screening for aflatoxins b1, m1, and g1 detoxification. *Eur Food Res Technol*. 2011;**232**:957-62 <https://doi.org/10.1007/s00217-011-1463-3>

208. Wang L, Wu J, Liu Z *et al.* Aflatoxin b1 degradation and detoxification by escherichia coli cg1061 isolated from chicken cecum. *Front Pharmacol*. 2019;**9** <https://doi.org/10.3389/fphar.2018.01548>

209. Yu H, Zhou T, Gong J *et al.* Isolation of deoxynivalenol-transforming bacteria from the chicken intestines using the approach of pcr-dgge guided microbial selection. *BMC Microbiol*. 2010;**10**:182 <https://doi.org/10.1186/1471-2180-10-182>

210. Guan S, He J, Young JC *et al.* Transformation of trichothecene mycotoxins by microorganisms from fish digesta. *Aquaculture*. 2009;**290**:290-95 <https://doi.org/10.1016/j.aquaculture.2009.02.037>

211. Kollarczik B, Gareis M, Hanelt M. In vitro transformation of the fusarium mycotoxins deoxynivalenol and zearalenone by the normal gut microflora of pigs. *Nat Toxins*. 1994;**2**:105-10 <https://doi.org/10.1002/nt.2620020303>

212. Kasimir M, Behrens M, Schulz M *et al.* Intestinal metabolism of α- and β-glucosylated modified mycotoxins t-2 and ht-2 toxin in the pig cecum model. *Journal of Agricultural* and *Food Chemistry*. 2020;**68**:5455-61 <https://doi.org/10.1021/acs.jafc.0c00576>

213. Müller HM, Lerch C, Müller K *et al.* Kinetic profiles of ochratoxin a and ochratoxin α during in vitro incubation in buffered forestomach and abomasal contents from cows. *Nat Toxins*. 1998;**6**:251-58 <https://doi.org/10.1002/(SICI)1522-7189(199811/12)6:6><251::AID-NT35>3.0.CO;2-P

214. Hult K, Teiling A, Gatenbeck S. Degradation of ochratoxin a by a ruminant. *Applied* and *Environmental Microbiology*. 1976;**32**:443-44 <https://doi.org/10.1128/aem.32.3.443-444.1976>

215. Tan H, Zhang Z, Hu Y *et al.* Isolation and characterization of pseudomonas otitidis th-n1 capable of degrading zearalenone. *Food Control*. 2015;**47**:285-90 <https://doi.org/10.1016/j.foodcont.2014.07.013>

216. Wang JQ, Yang F, Yang PL *et al.* Microbial reduction of zearalenone by a new isolated lysinibacillus sp. Zj-2016-1. *World mycotoxin journal*. 2018;**11**:571-78 <https://doi.org/10.3920/WMJ2017.2264>

217. Lv M, Liu Y, Wang M *et al.* Biotransformation of tetrabromobisphenol a and its analogs by selected gut bacteria strains: Implications for human health. *Environ Sci Technol*. 2024;**58**:20894-905 <https://doi.org/10.1021/acs.est.4c10434>

218. Zhang H, Zhang S, Hou X *et al.* Deglycosylation of two typical plant-generated glycoconjugates of tetrabromobisphenol a through in vitro digestion processes. *Environ Sci Technol Lett*. 2022;**9**:955-61 <https://doi.org/10.1021/acs.estlett.2c00757>

219. Yin J, Li D, Zheng T *et al.* Gastrointestinal degradation and toxicity of disinfection byproducts in drinking water using in vitro models and the roles of gut microbiota. *Environ Sci Technol*. 2023;**57**:16219-31 <https://doi.org/10.1021/acs.est.3c04483>

220. Wang Y, Rui M, Nie Y *et al.* Influence of gastrointestinal tract on metabolism of bisphenol a as determined by in vitro simulated system. *J Hazard Mater*. 2018;**355**:111-18 <https://doi.org/10.1016/j.jhazmat.2018.05.011>

221. Hou R, Zhang S, Huang Q *et al.* Role of gastrointestinal microbiota from crucian carp in microbial transformation and estrogenicity modification of novel plastic additives. *Environ Sci Technol*. 2023;**57**:11476-88 <https://doi.org/10.1021/acs.est.3c03595>

222. Zhang S, Hou R, Sun C *et al.* Metabolic activity of gut microbial enrichment cultures from different marine species and their transformation abilities to plastic additives. *Environ Int*. 2024;**190**:108882 <https://doi.org/https://doi.org/10.1016/j.envint.2024.108882>

223. Luo S, Zhen Z, Teng T *et al.* New mechanisms of biochar-assisted vermicomposting by recognizing different active di-(2-ethylhexyl) phthalate (dehp) degraders across pedosphere, charosphere and intestinal sphere. *J Hazard Mater*. 2023;**458**:131990 <https://doi.org/10.1016/j.jhazmat.2023.131990>

224. Van Dexter S, Boopathy R. Biodegradation of phenol by acinetobacter tandoii isolated from the gut of the termite. *Environ Sci Pollut R*. 2019;**26**:34067-72 <https://doi.org/10.1007/s11356-018-3292-4>

225. Wu X, Zhu Y, Yang M *et al.* Earthworms enhance the bioremediation of tris(2-butoxyethyl) phosphate-contaminated soil by releasing degrading microbes. *J Hazard Mater*. 2023;**452**:131303 <https://doi.org/10.1016/j.jhazmat.2023.131303>

226. Cui X, Wang X, Chang X *et al.* A new capacity of gut microbiota: Fermentation of engineered inorganic carbon nanomaterials into endogenous organic metabolites. *Proc Natl Acad Sci*. 2023;**120**:e2218739120 <https://doi.org/10.1073/pnas.2218739120>

227. LeMoine CMR, Grove HC, Smith CM *et al.* A very hungry caterpillar: Polyethylene metabolism and lipid homeostasis in larvae of the greater wax moth (galleria mellonella). *Environ Sci Technol*. 2020;**54**:14706-15 <https://doi.org/10.1021/acs.est.0c04386>

228. Cassone BJ, Grove HC, Elebute O *et al.* Role of the intestinal microbiome in low-density polyethylene degradation by caterpillar larvae of the greater wax moth, galleria mellonella. *Proceedings of the Royal Society B: Biological Sciences*. 2020;**287**:20200112 <https://doi.org/10.1098/rspb.2020.0112>

229. Billen P, Khalifa L, Van Gerven F *et al.* Technological application potential of polyethylene and polystyrene biodegradation by macro-organisms such as mealworms and wax moth larvae. *Sci Total Environ*. 2020;**735**:139521 <https://doi.org/http://neimeng.zssgdsb-85176920tsgjnz.com:80/rwt/17/https/MSYXTLUQPJUB/10.1016/j.scitotenv.2020.139521>

230. Lou Y, Ekaterina P, Yang S-S *et al.* Biodegradation of polyethylene and polystyrene by greater wax moth larvae (galleria mellonella l.) and the effect of co-diet supplementation on the core gut microbiome. *Environ Sci Technol*. 2020;**54**:2821-31 <https://doi.org/10.1021/acs.est.9b07044>

231. Przemieniecki SW, Kosewska A, Ciesielski S *et al.* Changes in the gut microbiome and enzymatic profile of tenebrio molitor larvae biodegrading cellulose, polyethylene and polystyrene waste. *Environ Pollut*. 2020;**256**:113265 <https://doi.org/10.1016/j.envpol.2019.113265>

232. Yang S-S, Ding M-Q, He L *et al.* Biodegradation of polypropylene by yellow mealworms (tenebrio molitor) and superworms (zophobas atratus) via gut-microbe-dependent depolymerization. *Sci Total Environ*. 2021;**756**:144087 <https://doi.org/10.1016/j.scitotenv.2020.144087>

233. Yang Y, Chen J, Wu W-M *et al.* Complete genome sequence of bacillus sp. Yp1, a polyethylene-degrading bacterium from waxworm's gut. *J Biotechnol*. 2015;**200**:77-78 <https://doi.org/10.1016/j.jbiotec.2015.02.034>

234. Yang J, Yang Y, Wu W-M *et al.* Evidence of polyethylene biodegradation by bacterial strains from the guts of plastic-eating waxworms. *Environ Sci Technol*. 2014;**48**:13776-84 <https://doi.org/10.1021/es504038a>

235. Huerta Lwanga E, Thapa B, Yang X *et al.* Decay of low-density polyethylene by bacteria extracted from earthworm's guts: A potential for soil restoration. *Sci Total Environ*. 2018;**624**:753-57 <https://doi.org/10.1016/j.scitotenv.2017.12.144>

236. Brandon AM, Gao S-H, Tian R *et al.* Biodegradation of polyethylene and plastic mixtures in mealworms (larvae of tenebrio molitor) and effects on the gut microbiome. *Environ Sci Technol*. 2018;**52**:6526-33 <https://doi.org/10.1021/acs.est.8b02301>

237. Chen Z, Zhang Y, Xing R *et al.* Reactive oxygen species triggered oxidative degradation of polystyrene in the gut of superworms (zophobas atratus larvae). *Environ Sci Technol*. 2023;**57**:7867-74 <https://doi.org/10.1021/acs.est.3c00591>

238. Yang Y, Yang J, Wu W-M *et al.* Biodegradation and mineralization of polystyrene by plastic-eating mealworms: Part 2. Role of gut microorganisms. *Environ Sci Technol*. 2015;**49**:12087-93 <https://doi.org/10.1021/acs.est.5b02663>

239. Kim HR, Lee HM, Yu HC *et al.* Biodegradation of polystyrene by pseudomonas sp. Isolated from the gut of superworms (larvae of zophobas atratus). *Environ Sci Technol*. 2020;**54**:6987-96 <https://doi.org/10.1021/acs.est.0c01495>

240. Yang Y, Wang J, Xia M. Biodegradation and mineralization of polystyrene by plastic-eating superworms zophobas atratus. *Sci Total Environ*. 2020;**708**:135233 <https://doi.org/10.1016/j.scitotenv.2019.135233>

241. Peng B-Y, Sun Y, Xiao S *et al.* Influence of polymer size on polystyrene biodegradation in mealworms (tenebrio molitor): Responses of depolymerization pattern, gut microbiome, and metabolome to polymers with low to ultrahigh molecular weight. *Environ Sci Technol*. 2022;**56**:17310-20 <https://doi.org/10.1021/acs.est.2c06260>

242. Brandon AM, Garcia AM, Khlystov NA *et al.* Enhanced bioavailability and microbial biodegradation of polystyrene in an enrichment derived from the gut microbiome of tenebrio molitor (mealworm larvae). *Environ Sci Technol*. 2021;**55**:2027-36 <https://doi.org/10.1021/acs.est.0c04952>

243. Kong F, Hong K-J, Xu H *et al.* Evidence of polystyrene biodegradation by gut microbiota of styrofoam-feeding yellow mealworms (larvae of tenebrio molitor linnaeus). *Microbiology China*. 2018;**45**:1438-49

244. Zhang Z, Peng H, Yang D *et al.* Polyvinyl chloride degradation by a bacterium isolated from the gut of insect larvae. *Nature Communications*. 2022;**13**:5360 <https://doi.org/10.1038/s41467-022-32903-y>

245. Bao L, Cui X, Zeng T *et al.* Incorporation of polylactic acid microplastics into the carbon cycle as a carbon source to remodel the endogenous metabolism of the gut. *Proc Natl Acad Sci*. 2025;**122**:e2417104122 <https://doi.org/10.1073/pnas.2417104122>

246. Soleim HA, Scheline RR. Metabolism of xenobiotics by strains of intestinal bacteria. *Acta Pharmacol Toxicol (Copenh)*. 1972;**31**:471-80 <https://doi.org/10.1111/j.1600-0773.1972.tb03610.x>

247. Bürger S, Stolz A. Characterisation of the flavin-free oxygen-tolerant azoreductase from xenophilus azovorans kf46f in comparison to flavin-containing azoreductases. *Applied Microbiology* and *Biotechnology*. 2010;**87**:2067-76 <https://doi.org/10.1007/s00253-010-2669-1>

248. Matsumoto Ki, Mukai Y, Ogata D *et al.* Characterization of thermostable fmn-dependent nadh azoreductase from the moderate thermophile geobacillus stearothermophilus. *Applied Microbiology* and *Biotechnology*. 2010;**86**:1431-38 <https://doi.org/10.1007/s00253-009-2351-7>

249. Bracco P, Janssen DB, Schallmey A. Selective steroid oxyfunctionalisation by cyp154c5, a bacterial cytochrome p450. *Microb Cell Fact*. 2013;**12**:95 <https://doi.org/10.1186/1475-2859-12-95>

250. Narhi LO, Fulco AJ. Characterization of a catalytically self-sufficient 119,000-dalton cytochrome p-450 monooxygenase induced by barbiturates in bacillus megaterium. *J Biol Chem*. 1986;**261**:7160-69 <https://doi.org/10.1016/S0021-9258(17)38369-2>

251. Murooka Y, Doi N, Harada T. Distribution of membrane-bound monoamine oxidase in bacteria. *Applied* and *Environmental Microbiology*. 1979;**38**:565-69 <https://doi.org/10.1128/aem.38.4.565-569.1979>

252. Lehouritis P, Cummins J, Stanton M *et al.* Local bacteria affect the efficacy of chemotherapeutic drugs. *Sci Rep*. 2015;**5**:14554 <https://doi.org/10.1038/srep14554>

253. Zenno S, Kobori T, Tanokura M *et al.* Conversion of nfsa, the major escherichia colinitroreductase, to a flavin reductase with an activity similar to that of frp, a flavin reductase in vibrio harveyi, by a single amino acid substitution. *J Bacteriol*. 1998;**180**:422-25 <https://doi.org/10.1128/jb.180.2.422-425.1998>

254. Zenno S, Koike H, Tanokura M *et al.* Gene cloning, purification, and characterization of nfsb, a minor oxygen-insensitive nitroreductase from escherichia coli, similar in biochemical properties to frase i, the major flavin reductase in vibrio fischeri1. *The Journal of Biochemistry*. 1996;**120**:736-44 <https://doi.org/10.1093/oxfordjournals.jbchem.a021473>

255. Watanabe M, Nishino T, Takio K *et al.* Purification and characterization of wild-type and mutant “classical” nitroreductases of salmonella typhimurium: L33r mutation greatly diminishes binding of fmn to the nitroreductase of s. Typhimurium*. *Journal of Biological Chemistry*. 1998;**273**:23922-28 <https://doi.org/https://doi.org/10.1074/jbc.273.37.23922>

256. Nokhbeh MR, Boroumandi S, Pokorny N *et al.* Identification and characterization of snra, an inducible oxygen-insensitive nitroreductase in salmonella enterica serovar typhimurium ta1535. *Mutation Research/Fundamental* and *Molecular Mechanisms of Mutagenesis*. 2002;**508**:59-70 <https://doi.org/10.1016/S0027-5107(02)00174-4>

257. Bryant C, DeLuca M. Purification and characterization of an oxygen-insensitive nad(p)h nitroreductase from enterobacter cloacae*. *J Biol Chem*. 1991;**266**:4119-25 <https://doi.org/10.1016/S0021-9258(20)64294-6>

258. Koder RL, Miller A-F. Overexpression, isotopic labeling, and spectral characterization of enterobacter cloacae nitroreductase. *Protein Expression* and *Purification*. 1998;**13**:53-60 <https://doi.org/10.1006/prep.1997.0866>

259. Pérez-Reinado E, Blasco R, Castillo F *et al.* Regulation and characterization of two nitroreductase genes, <i>npra</i> and <i>nprb</i>, of <i>rhodobacter capsulatus</i>. *Applied* and *Environmental Microbiology*. 2005;**71**:7643-49 <https://doi.org/10.1128/AEM.71.12.7643-7649.2005>

260. Wallace Bret D, Roberts Adam B, Pollet Rebecca M *et al.* Structure and inhibition of microbiome β-glucuronidases essential to the alleviation of cancer drug toxicity. *Chem Biol*. 2015;**22**:1238-49 <https://doi.org/https://doi.org/10.1016/j.chembiol.2015.08.005>

261. Biernat KA, Pellock SJ, Bhatt AP *et al.* Structure, function, and inhibition of drug reactivating human gut microbial β-glucuronidases. *Sci Rep*. 2019;**9**:825 <https://doi.org/10.1038/s41598-018-36069-w>

262. Mudziwapasi R, Chigu NL, Kuipa PK *et al.* Isolation and molecular characterization of bacteria from the gut of eisenia fetida for biodegradation of 4, 4 ddt. 2016

263. Ramirez RF, Dixon BA. Enzyme production by obligate intestinal anaerobic bacteria isolated from oscars (astronotus ocellatus), angelfish (pterophyllum scalare) and southern flounder (paralichthys lethostigma). *Aquaculture*. 2003;**227**:417-26 <https://doi.org/10.1016/S0044-8486(03)00520-9>

264. Kim D-H, Hyun S-H, Shim S-B *et al.* The role of intestinal bacteria in the transformation of sodium picosulfate. *The Japanese Journal of Pharmacology*. 1992;**59**:1-5 <https://doi.org/10.1254/jjp.59.1>

265. Tao J-h, Duan J-a, Jiang S *et al.* Biotransformation and metabolic profile of buddleoside with human intestinal microflora by ultrahigh-performance liquid chromatography coupled to hybrid linear ion trap/orbitrap mass spectrometer. *J Chromatogr B*. 2016;**1025**:7-15 <https://doi.org/10.1016/j.jchromb.2016.04.055>

266. Das A, Srinivasan M, Ghosh TS *et al.* Xenobiotic metabolism and gut microbiomes. *PLoS One*. 2016;**11**:e0163099 <https://doi.org/10.1371/journal.pone.0163099>

267. Nosova T, Jokelainen K, Kaihovaara P *et al.* Characteristics of aldehyde dehydrogenases of certain aerobic bacteria representing human colonic flora. *Alcohol* and *Alcoholism*. 1998;**33**:273-80 <https://doi.org/10.1093/oxfordjournals.alcalc.a008391>

268. Dabek M, McCrae SI, Stevens VJ *et al.* Distribution of β-glucosidase and β-glucuronidase activity and of β-glucuronidase gene gus in human colonic bacteria. *FEMS Microbiol Ecol*. 2008;**66**:487-95 <https://doi.org/10.1111/j.1574-6941.2008.00520.x>

269. Nakamura J, Kubota Y, Miyaoka M *et al.* Comparison of four microbial enzymes in clostridia and bacteroides isolated from human feces. *Microbiol Immunol*. 2002;**46**:487-90 <https://doi.org/10.1111/j.1348-0421.2002.tb02723.x>

270. Paraiso IL, Plagmann LS, Yang L *et al.* Reductive metabolism of xanthohumol and 8-prenylnaringenin by the intestinal bacterium eubacterium ramulus. *Mol Nutr Food Res*. 2019;**63**:1800923 <https://doi.org/10.1002/mnfr.201800923>

271. Tasse L, Bercovici J, Pizzut-Serin S *et al.* Functional metagenomics to mine the human gut microbiome for dietary fiber catabolic enzymes. *Genome Res*. 2010;**20**:1605-12 <https://doi.org/10.1101/gr.108332.110>

272. Kobashi K, Nishimura T, Kusaka M *et al.* Metabolism of sennosides by human intestinal bacteria. *Planta Med*. 1980;**40**:225-36 <https://doi.org/10.1055/s-2008-1074963>

273. Xu J, Bjursell MK, Himrod J *et al.* A genomic view of the human-bacteroides thetaiotaomicron symbiosis. *Science*. 2003;**299**:2074-76 <https://doi.org/10.1126/science.1080029>

274. Flores R, Shi J, Gail MH *et al.* Association of fecal microbial diversity and taxonomy with selected enzymatic functions. *PLoS One*. 2012;**7**:e39745 <https://doi.org/10.1371/journal.pone.0039745>

275. Gloux K, Berteau O, El oumami H *et al.* A metagenomic β-glucuronidase uncovers a core adaptive function of the human intestinal microbiome. *Proc Natl Acad Sci*. 2011;**108**:4539-46 <https://doi.org/10.1073/pnas.1000066107>

276. Kim D-H, Hong S-W, Kim B-T *et al.* Biotransformation of glycyrrhizin by human intestinal bacteria and its relation to biological activities. *Archives of pharmacal research*. 2000;**23**:172-77 <https://doi.org/10.1007/BF02975509>

277. Elmassry MM, Kim S, Busby B. Predicting drug-metagenome interactions: Variation in the microbial β-glucuronidase level in the human gut metagenomes. *PLoS One*. 2021;**16**:e0244876 <https://doi.org/10.1371/journal.pone.0244876>

278. Mcbain AJ, Macfarlane GT. Ecological and physiological studies on large intestinal bacteria in relation to production of hydrolytic and reductive enzymes involved in formation of genotoxic metabolites. *J Med Microbiol*. 1998;**47**:407-16 <https://doi.org/10.1099/00222615-47-5-407>

279. Ervin SM, Simpson JB, Gibbs ME *et al.* Structural insights into endobiotic reactivation by human gut microbiome-encoded sulfatases. *Biochemistry*. 2020;**59**:3939-50 <https://doi.org/10.1021/acs.biochem.0c00711>

280. Beaud D, Tailliez P, Anba-Mondoloni J. Genetic characterization of the β-glucuronidase enzyme from a human intestinal bacterium, ruminococcus gnavus. *Microbiology*. 2005;**151**:2323-30 <https://doi.org/10.1099/mic.0.27712-0>

281. Chung JG, Hsia TC, Kuo HM *et al.* Inhibitory actions of luteolin on the growth and arylamine n-acetyltransferase activity in strains of helicobacter pylori from ulcer patients. *Toxicol In Vitro*. 2001;**15**:191-98 <https://doi.org/10.1016/S0887-2333(01)00015-7>

282. Dempsey AC, Kitting CL. Characteristics of bacteria isolated from penaeid shrimp. *Crustaceana*. 1987;**52**:90-94

283. Atlas RM, Busdosh M, Krichevsky EJ *et al.* Bacterial populations associated with the arctic amphipod boeckosimus affinis. *Can J Microbiol*. 1982;**28**:92-99 <https://doi.org/10.1139/m82-008>

284. Herrera LM, García-Laviña CX, Marizcurrena JJ *et al.* Hydrolytic enzyme-producing microbes in the antarctic oligochaete grania sp. (annelida). *Polar Biol*. 2017;**40**:947-53 <https://doi.org/10.1007/s00300-016-2012-0>

285. Sochard MR, Wilson DF, Austin B *et al.* Bacteria associated with the surface and gut of marine copepods. *Applied* and *Environmental Microbiology*. 1979;**37**:750-59 <https://doi.org/10.1128/aem.37.4.750-759.1979>

286. Tzuc JT, Escalante DR, Rojas Herrera R *et al.* Microbiota from litopenaeus vannamei: Digestive tract microbial community of pacific white shrimp (litopenaeus vannamei). *SpringerPlus*. 2014;**3**:280 <https://doi.org/10.1186/2193-1801-3-280>

287. Lein EY, Mohamad Lal MT, Venmathi Maran BA *et al.* Gastrointestinal microbiota of spiny lobster: A review. *Fishes*.

288. Harris JM, Seiderer LJ, Lucas MI. Gut microflora of two saltmarsh detritivore thalassinid prawns,upogebia africana andcallianassa kraussi. *Microb Ecol*. 1991;**21**:277-96 <https://doi.org/10.1007/BF02539159>

289. Wang Y, Al Farraj DA, Vijayaraghavan P *et al.* Host associated mixed probiotic bacteria induced digestive enzymes in the gut of tiger shrimp penaeus monodon. *Saudi J Biol Sci*. 2020;**27**:2479-84 <https://doi.org/10.1016/j.sjbs.2020.07.010>

290. Li F, Gao F, Tan J *et al.* Characterization and identification of enzyme-producing microflora isolated from the gut of sea cucumber apostichopus japonicus. *Chin J Oceanol Limnol*. 2016;**34**:153-62 <https://doi.org/10.1007/s00343-015-4149-z>

291. Liu H, Guo X, Gooneratne R *et al.* The gut microbiome and degradation enzyme activity of wild freshwater fishes influenced by their trophic levels. *Scientific Reports*. 2016;**6**:24340 <https://doi.org/10.1038/srep24340>

292. Sugita H, Kawasaki J, Deguchi Y. Production of amylase by the intestinal microflora in cultured freshwater fish. *Lett Appl Microbiol*. 1997;**24**:105-8 <https://doi.org/10.1046/j.1472-765x.1997.00360.x>

293. Mukherjee A, Dutta D, Banerjee S *et al.* Potential probiotics from indian major carp, cirrhinus mrigala. Characterization, pathogen inhibitory activity, partial characterization of bacteriocin and production of exoenzymes. *Res Vet Sci*. 2016;**108**:76-84 <https://doi.org/10.1016/j.rvsc.2016.08.011>

294. Khan A, Mandal S, Samanta D *et al.* Phytase-producing rhodococcus sp. (mtcc 9508) from fish gut: A preliminary study. *Proceedings of the Zoological Society*. 2011;**64**:29-34 <https://doi.org/10.1007/s12595-011-0004-1>

295. Banerjee S, Mukherjee A, Dutta D *et al.* Evaluation of chitinolytic gut microbiota in some carps and optimization of culture conditions for chitinase production by the selected bacteria. *Journal of Microbiology, Biotechnology* and *Food Sciences*. 2015;**5**:12-19 <https://doi.org/10.15414/jmbfs.2015.5.1.12-19>

296. Banerjee S, Ghosh K. Enumeration of gut associated extracellular enzyme-producing yeasts in some freshwater fishes. *J Appl Ichthyol*. 2014;**30**:986-93 <https://doi.org/10.1111/jai.12457>

297. Ray AK, Roy T, Mondal S *et al.* Identification of gut-associated amylase, cellulase and protease-producing bacteria in three species of indian major carps. *Aquacult Res*. 2010;**41**:1462-69 <https://doi.org/10.1111/j.1365-2109.2009.02437.x>

298. Roy T, Mondal S, Ray AK. Phytase-producing bacteria in the digestive tracts of some freshwater fish. *Aquacult Res*. 2009;**40**:344-53 <https://doi.org/10.1111/j.1365-2109.2008.02100.x>

299. Mandal S, Ghosh K. Isolation of tannase-producing microbiota from the gastrointestinal tracts of some freshwater fish. *J Appl Ichthyol*. 2013;**29**:145-53 <https://doi.org/10.1111/j.1439-0426.2012.02054.x>

300. Jiang Y, Xie C, Yang G *et al.* Cellulase-producing bacteria of aeromonas are dominant and indigenous in the gut of ctenopharyngodon idellus (valenciennes). *Aquacult Res*. 2011;**42**:499-505 <https://doi.org/10.1111/j.1365-2109.2010.02645.x>

301. Trust TJ, Bull LM, Currie BR *et al.* Obligate anaerobic bacteria in the gastrointestinal microflora of the grass carp (ctenopharyngodon idella), goldfish (carassius auratus), and rainbow trout (salmo gairdneri). *Journal of the Fisheries Research Board of Canada*. 1979;**36**:1174-79 <https://doi.org/10.1139/f79-169>

302. Li H, Wu S, Wirth S *et al.* Diversity and activity of cellulolytic bacteria, isolated from the gut contents of grass carp (ctenopharyngodon idellus) (valenciennes) fed on sudan grass (sorghum sudanense) or artificial feedstuffs. *Aquacult Res*. 2016;**47**:153-64 <https://doi.org/10.1111/are.12478>

303. Li H, Zheng Z, Cong-xin X *et al.* Isolation of cellulose—producing microbes from the intestine of grass carp (ctenopharyngodon idellus). In: Noakes DLG, Romero A, Zhao Y *et al.* (eds.). *Chinese fishes*, Dordrecht: Springer Netherlands. 131-35. Retreived from <https://doi.org/10.1007/978-90-481-3458-8_19>

304. Sugita H, Yamada S, Konagaya Y *et al.* Production of &beta;-<i>n</i>-acetylglucosaminidase and chitinase by <i>aeromonas</i> species isolated from river fish. *Fish Sci*. 1999;**65**:155-58 <https://doi.org/10.2331/fishsci.65.155>

305. Gatesoupe F-J, Infante J-LZ, Cahu C *et al.* Early weaning of seabass larvae, dicentrarchus labrax: The effect on microbiota, with particular attention to iron supply and exoenzymes. *Aquaculture*. 1997;**158**:117-27 <https://doi.org/10.1016/S0044-8486(97)00179-8>

306. Hamid A, Sakata T, Kakimoto D. Microflora in the alimentary tract of gray mullet-iv

estimation of enzymic activities of the intestinal bacteria. *Nippon Suisan Gakkaishi*. 1979;**45**:99-106 <https://doi.org/10.2331/suisan.45.99>

307. Mondal S, Roy T, Ray AK. Characterization and identification of enzyme-producing bacteria isolated from the digestive tract of bata, labeo bata. *J World Aquacult Soc*. 2010;**41**:369-77 <https://doi.org/10.1111/j.1749-7345.2010.00378.x>

308. Ghosh K, Sen SK, Ray AK. Characterization of bacilli isolated from the gut of rohu, labeo rohita, fingerlings and its significance in digestion. *J Appl Aquacult*. 2002;**12**:33-42 <https://doi.org/10.1300/J028v12n03_04>

309. Banerjee G, Ray AK, Askarian F *et al.* Characterisation and identification of enzyme-producing autochthonous bacteria from the gastrointestinal tract of two indian air-breathing fish. *Beneficial Microbes*. 2013;**4**:277-84 <https://doi.org/10.3920/BM2012.0051>

310. Dutta D, Ghosh K. Screening of extracellular enzyme-producing and pathogen inhibitory gut bacteria as putative probiotics in mrigal, cirrhinus mrigala (hamilton, 1822). *International Journal of Fisheries* and *Aquatic Studies*. 2015;**2**:310-18

311. Tan HY, Chen S-W, Hu S-Y. Improvements in the growth performance, immunity, disease resistance, and gut microbiota by the probiotic rummeliibacillus stabekisii in nile tilapia (oreochromis niloticus). *Fish Shellfish Immunol*. 2019;**92**:265-75 <https://doi.org/10.1016/j.fsi.2019.06.027>

312. Peixoto SB, Cladera-Olivera F, Daroit DJ *et al.* Cellulase-producing bacillus strains isolated from the intestine of amazon basin fish. *Aquacult Res*. 2011;**42**:887-91 <https://doi.org/10.1111/j.1365-2109.2010.02727.x>

313. Watts JEM, McDonald R, Daniel R *et al.* Examination of a culturable microbial population from the gastrointestinal tract of the wood-eating loricariid catfish panaque nigrolineatus. *Diversity*. 641-56.

314. Skrodenytė-Arbaĉiauskienė V. Enzymatic activity of intestinal bacteria in roach rutilus rutilus l. *Fish Sci*. 2007;**73**:964-66 <https://doi.org/10.1111/j.1444-2906.2007.01421.x>

315. Jami M, Ghanbari M, Kneifel W *et al.* Phylogenetic diversity and biological activity of culturable actinobacteria isolated from freshwater fish gut microbiota. *Microbiol Res*. 2015;**175**:6-15 <https://doi.org/10.1016/j.micres.2015.01.009>

316. Sakata T, Okabayashi J, Kakimoto D. Variations in the intestinal microflora of <i>tilapia</i> reared in fresh and sea water. *Nippon Suisan Gakkaishi*. 1980;**46**:313-17 <https://doi.org/10.2331/suisan.46.313>

317. Sakata T, Koreeda Y. A numerical taxonomic study of the dominant bacteria isolated from tilapia intestines. *Nippon Suisan Gakkaishi*. 1986;**52**:1625-34 <https://doi.org/10.2331/suisan.52.1625>

318. Saha S, Roy RN, Sen SK *et al.* Characterization of cellulase-producing bacteria from the digestive tract of tilapia, oreochromis mossambica (peters) and grass carp, ctenopharyngodon idella (valenciennes). *Aquacult Res*. 2006;**37**:380-88 <https://doi.org/10.1111/j.1365-2109.2006.01442.x>

319. Dey A, Ghosh K, Hazra N. Evaluation of extracellular enzyme-producing autochthonous gut bacteria in walking catfish, clarias batrachus (l.). *Journal of Fisheries*. 2016;**4**:345-52 <https://doi.org/10.17017/jfish.v4i1.2016.115>

320. MacDonald NL, Stark JR, Austin B. Bacterial microflora in the gastro-intestinal tract of dover sole (solea solea l.), with emphasis on the possible role of bacteria in the nutrition of the host. *FEMS Microbiology Letters*. 1986;**35**:107-11 <https://doi.org/https://doi.org/10.1111/j.1574-6968.1986.tb01508.x>

321. Hoshino T, Ishizaki K, Sakamoto T *et al.* Isolation of a pseudomonas species from fish intestine that produces a protease active at low temperature. *Lett Appl Microbiol*. 1997;**25**:70-72 <https://doi.org/10.1046/j.1472-765X.1997.00183.x>

322. Henderson RJ, Millar RM. Characterization of lipolytic activity associated with a vibrio species of bacterium isolated from fish intestines. *J Mar Biotechnol*. 1998;**6**:168-73 <https://doi.org/10.1038/sj.jim.2900482>

323. Askarian F, Sperstad S, Merrifield DL *et al.* The effect of different feeding regimes on enzyme activities of gut microbiota in atlantic cod (gadus morhua l.). *Aquacult Res*. 2013;**44**:841-46 <https://doi.org/10.1111/j.1365-2109.2011.03079.x>

324. Askarian F, Zhou Z, Olsen RE *et al.* Culturable autochthonous gut bacteria in atlantic salmon (salmo salar l.) fed diets with or without chitin. Characterization by 16s rrna gene sequencing, ability to produce enzymes and in vitro growth inhibition of four fish pathogens. *Aquaculture*. 2012;**326-329**:1-8 <https://doi.org/10.1016/j.aquaculture.2011.10.016>

325. Lazado CC, Caipang CMA, Kiron V. Enzymes from the gut bacteria of atlantic cod, gadus morhua and their influence on intestinal enzyme activity. *Aquacult Nutr*. 2012;**18**:423-31 <https://doi.org/10.1111/j.1365-2095.2011.00928.x>

326. Hossain TJ, Chowdhury SI, Mozumder HA *et al.* Hydrolytic exoenzymes produced by bacteria isolated and identified from the gastrointestinal tract of bombay duck. *Frontiers in Microbiology*. 2020;**11** <https://doi.org/10.3389/fmicb.2020.02097>

327. Augustine A, Joseph I. Four novel strains of cellulolytic symbiotic bacteria isolated and characterized from gi tract of marine fishes of various feeding habits. *Biocatalysis* and *Agricultural Biotechnology*. 2018;**16**:706-14 <https://doi.org/10.1016/j.bcab.2018.05.009>

328. Sugita H, Ito Y. Identification of intest\inal bacteria from japanese flounder (paralichthys olivaceus) and their ability to digest chitin. *Lett Appl Microbiol*. 2006;**43**:336-42 <https://doi.org/10.1111/j.1472-765X.2006.01943.x>

329. Reshma KJ, Sumithra TG, Nair AV *et al.* An insight into the gut microbiology of wild-caught mangrove red snapper, lutjanus argentimaculatus (forsskal, 1775). *Aquaculture*. 2018;**497**:320-30 <https://doi.org/10.1016/j.aquaculture.2018.08.008>

330. Hortillosa EM, Amar MJA, Nuñal SN *et al.* Effects of putative dietary probiotics from the gut of milkfish (chanos chanos) on the growth performance and intestinal enzymatic activities of juvenile nile tilapia (oreochromis niloticus). *Aquacult Res*. 2022;**53**:98-108 <https://doi.org/10.1111/are.15556>

331. Esakkiraj P, Immanuel G, Sowmya SM *et al.* Evaluation of protease-producing ability of fish gut isolate bacillus cereus for aqua feed. *Food* and *Bioprocess Technology*. 2009;**2**:383-90 <https://doi.org/10.1007/s11947-007-0046-6>

332. Simora RMC, Traifalgar RFM, Legario FS. Characterization of extracellular enzymes from culturable autochthonous gut bacteria in rabbitfish (siganus guttatus). *Extreme Life, Biospeology* and *Astrobiology*. 2015;**7**:67-76

333. Erfanimoghadam MR, Homaei A Identification of new amylolytic enzymes from marine symbiotic bacteria of bacillus species. *Catalysts*.

334. Morita Y, Hasan Q, Sakaguchi T *et al.* Properties of a cold-active protease from psychrotrophic flavobacterium balustinum p104. *Applied Microbiology* and *Biotechnology*. 1998;**50**:669-75 <https://doi.org/10.1007/s002530051349>

335. Ringø E, Strøm E, Tabachek JA. Intestinal microflora of salmonids: A review. *Aquaculture Research*. 1995;**26**:773-89 <https://doi.org/https://doi.org/10.1111/j.1365-2109.1995.tb00870.x>

336. Das P, Mandal S, Khan A *et al.* Distribution of extracellular enzyme-producing bacteria in the digestive tracts of 4 brackish water fish species. *Turkish Journal of Zoology*. 2014;**38**:10 <https://doi.org/10.1023/A:1021355406412>

337. Li X, Chi Z, Liu Z *et al.* Phytase production by a marine yeast kodamea ohmeri bg3. *Applied Biochemistry* and *Biotechnology*. 2008;**149**:183-93 <https://doi.org/10.1007/s12010-007-8099-6>

338. Itoi S, Okamura T, Koyama Y *et al.* Chitinolytic bacteria in the intestinal tract of japanese coastal fishes. *Can J Microbiol*. 2006;**52**:1158-63 <https://doi.org/10.1139/w06-082>

339. Carberry CA, Waters SM, Kenny DA *et al.* Rumen methanogenic genotypes differ in abundance according to host residual feed intake phenotype and diet type. *Applied* and *Environmental Microbiology*. 2014;**80**:03131-13 <https://doi.org/10.1128/cmr.00039-07>

340. Zhou M, Hernandez-Sanabria E, Guan Le L. Characterization of variation in rumen methanogenic communities under different dietary and host feed efficiency conditions, as determined by pcr-denaturing gradient gel electrophoresis analysis. *Applied* and *Environmental Microbiology*. 2010;**76**:3776-86 <https://doi.org/10.1128/AEM.00010-10>

341. Fields MW, Russell JB, Wilson DB. The role of ruminal carboxymethylcellulases in the degradation of β-glucans from cereal grain1proprietary or brand names are necessary to report factually on available data; however, the usda neither guarantees nor warrants the standard of the product, and the use of the name by the usda implies no approval of the product, and exclusion of others that may be suitable.1. *FEMS Microbiol Ecol*. 1998;**27**:261-68 <https://doi.org/10.1016/S0168-6496(98)00072-5>

342. Weimer PJ, Waghorn GC, Odt CL *et al.* Effect of diet on populations of three species of ruminal cellulolytic bacteria in lactating dairy cows1. *J Dairy Sci*. 1999;**82**:122-34 <https://doi.org/10.3168/jds.S0022-0302(99)75216-1>

343. Ziemer Cherie J. Newly cultured bacteria with broad diversity isolated from eight-week continuous culture enrichments of cow feces on complex polysaccharides. *Applied* and *Environmental Microbiology*. 2014;**80**:574-85 <https://doi.org/10.1128/AEM.03016-13>

344. Julliand V, Vaux Ad, Millet L *et al.* Identification of ruminococcus flavefaciens as the predominant cellulolytic bacterial species of the equine cecum. *Applied* and *Environmental Microbiology*. 1999;**65**:3738-41 <https://doi.org/10.1128/AEM.65.8.3738-3741.1999>

345. Varel VH, Yen JT. Microbial perspective on fiber utilization by swine1. *J Anim Sci*. 1997;**75**:2715-22 <https://doi.org/10.2527/1997.75102715x>

346. Hespell RB, Cotta MA. Degradation and utilization by butyrivibrio fibrisolvens h17c of xylans with different chemical and physical properties. *Applied* and *Environmental Microbiology*. 1995;**61**:3042-50 <https://doi.org/10.1128/cmr.00039-07>

347. Dehority BA, Scott HW. Extent of cellulose and hemicellulose digestion in various forages by pure cultures of rumen bacteria1. *J Dairy Sci*. 1967;**50**:1136-41 <https://doi.org/10.3168/jds.S0022-0302(67)87579-9>

348. Osborne JM, Dehority BA. Synergism in degradation and utilization of intact forage cellulose, hemicellulose, and pectin by three pure cultures of ruminal bacteria. *Applied* and *Environmental Microbiology*. 1989;**55**:2247-50 <https://doi.org/10.1128/aem.55.9.2247-2250.1989>

349. Krause DO, Dalrymple BP, Smith WJ *et al.* 16s rdna sequencing of ruminococcus albus and ruminococcus flavefaciens: Design of a signature probe and its application in adult sheep. *Microbiology*. 1999;**145**:1797-807 <https://doi.org/10.1099/13500872-145-7-1797>

350. Dehority BA. Effects of microbial synergism on fibre digestion in the rumen. *Proc Nutr Soc*. 1991;**50**:149-59 <https://doi.org/10.1079/PNS19910026>

351. Michalet-Doreau B, Fernandez I, Fonty G. A comparison of enzymatic and molecular approaches to characterize the cellulolytic microbial ecosystems of the rumen and the cecum1. *J Anim Sci*. 2002;**80**:790-96 <https://doi.org/10.2527/2002.803790x>

352. Ze X, Duncan SH, Louis P *et al.* Ruminococcus bromii is a keystone species for the degradation of resistant starch in the human colon. *The ISME Journal*. 2012;**6**:1535-43 <https://doi.org/10.1038/ismej.2012.4>

353. Sharp R, Macfarlane George T. Chemostat enrichments of human feces with resistant starch are selective for adherent butyrate-producing clostridia at high dilution rates. *Applied* and *Environmental Microbiology*. 2000;**66**:4212-21 <https://doi.org/10.1128/AEM.66.10.4212-4221.2000>

354. Anderson KL, Salyers AA. Biochemical evidence that starch breakdown by bacteroides thetaiotaomicron involves outer membrane starch-binding sites and periplasmic starch-degrading enzymes. *J Bacteriol*. 1989;**171**:3192-98 <https://doi.org/10.1128/cmr.00039-07>

355. Maier TV, Lucio M, Lang HL *et al.* Impact of dietary resistant starch on the human gut microbiome, metaproteome, and metabolome. *mBio*. 2017 **8**:01343-17 <https://doi.org/10.1128/cmr.00039-07>

356. Salyers AA, West SE, Vercellotti JR *et al.* Fermentation of mucins and plant polysaccharides by anaerobic bacteria from the human colon. *Applied* and *Environmental Microbiology*. 1977;**34**:529-33 <https://doi.org/10.1128/aem.34.5.529-533.1977>

357. Salyers AA, Vercellotti JR, West SE *et al.* Fermentation of mucin and plant polysaccharides by strains of bacteroides from the human colon. *Applied* and *Environmental Microbiology*. 1977;**33**:319-22 <https://doi.org/10.1128/aem.33.2.319-322.1977>

358. Macfarlane S, Woodmansey Emma J, Macfarlane George T. Colonization of mucin by human intestinal bacteria and establishment of biofilm communities in a two-stage continuous culture system. *Applied* and *Environmental Microbiology*. 2005;**71**:7483-92 <https://doi.org/10.1128/AEM.71.11.7483-7492.2005>

359. Caminero A, Herrán AR, Nistal E *et al.* Diversity of the cultivable human gut microbiome involved in gluten metabolism: Isolation of microorganisms with potential interest for coeliac disease. *FEMS Microbiol Ecol*. 2014;**88**:309-19 <https://doi.org/10.1111/1574-6941.12295>

360. Herrán AR, Pérez-Andrés J, Caminero A *et al.* Gluten-degrading bacteria are present in the human small intestine of healthy volunteers and celiac patients. *Res Microbiol*. 2017;**168**:673-84 <https://doi.org/10.1016/j.resmic.2017.04.008>

361. Kaoutari AE, Armougom F, Gordon JI *et al.* The abundance and variety of carbohydrate-active enzymes in the human gut microbiota. *Nat Rev Microbiol*. 2013;**11**:497-504 <https://doi.org/10.1038/nrmicro3050>

362. Leitch ECM, Walker AW, Duncan SH *et al.* Selective colonization of insoluble substrates by human faecal bacteria. *Environ Microbiol*. 2007;**9**:667-79 <https://doi.org/10.1111/j.1462-2920.2006.01186.x>

363. Derrien M, Vaughan EE, Plugge CM *et al.* Akkermansia muciniphila gen. Nov., sp. Nov., a human intestinal mucin-degrading bacterium. *Int J Syst Evol Microbiol*. 2004;**54**:1469-76 <https://doi.org/10.1099/ijs.0.02873-0>

364. Hoskins LC. Mucin degradation in the human gastrointestinal tract and its significance to enteric microbial ecology. *Eur J Gastroenterol Hepatol*. 1993;**5**:205-13

365. Wang X, Conway Patricia L, Brown Ian L *et al.* In vitro utilization of amylopectin and high-amylose maize (amylomaize) starch granules by human colonic bacteria. *Applied* and *Environmental Microbiology*. 1999;**65**:4848-54 <https://doi.org/10.1128/AEM.65.11.4848-4854.1999>

366. Oliphant K, Allen-Vercoe E. Macronutrient metabolism by the human gut microbiome: Major fermentation by-products and their impact on host health. *Microbiome*. 2019;**7**:91 <https://doi.org/10.1186/s40168-019-0704-8>

367. Schell MA, Karmirantzou M, Snel B *et al.* The genome sequence of bifidobacterium longum reflects its adaptation to the human gastrointestinal tract. *Proc Natl Acad Sci*. 2002;**99**:14422-27 <https://doi.org/10.1073/pnas.212527599>

368. Koropatkin NM, Cameron EA, Martens EC. How glycan metabolism shapes the human gut microbiota. *Nat Rev Microbiol*. 2012;**10**:323-35 <https://doi.org/10.1038/nrmicro2746>
